# Development and evaluation of a meat mitochondrial metagenomic (3MG) method for composition determination of meat from fifteen mammalian and avian species

**DOI:** 10.1186/s12864-021-08263-0

**Published:** 2022-01-07

**Authors:** Mei Jiang, Shu-Fei Xu, Tai-Shan Tang, Li Miao, Bao-Zheng Luo, Yang Ni, Fan-De Kong, Chang Liu

**Affiliations:** 1 Institute of Medicinal Plant Development, Chinese Academy of Medical Sciences & Peking Union Medical College, 100193 Beijing, PR China; 2 Technology Center of Xiamen Entry-exit Inspection and Quarantine Bureau, Xiamen, Fujian 361026 PR China; 3 Technology Center of Jiangsu Entry-exit Inspection and Quarantine Bureau, Nanjing, Jiangsu 210009 PR China; 4 Technology Center of Henan Entry-exit Inspection and Quarantine Bureau, Zhengzhou, Henan 450003 PR China; 5 Technology Center of Zhuhai Entry-exit Inspection and Quarantine Bureau, Zhuhai, Guangdong 519000 PR China; 6 College of Agriculture, Fujian Agriculture and Forestry University, Fuzhou, Fujian Province 350002 PR China

**Keywords:** Composition determination, Biomass estimation, Meat mix, Mitogenome, Mitochondrial metagenomics, Next-generation sequencing, Analysis pipeline

## Abstract

**Background:**

Bioassessment and biomonitoring of meat products are aimed at identifying and quantifying adulterants and contaminants, such as meat from unexpected sources and microbes. Several methods for determining the biological composition of mixed samples have been used, including metabarcoding, metagenomics and mitochondrial metagenomics. In this study, we aimed to develop a method based on next-generation DNA sequencing to estimate samples that might contain meat from 15 mammalian and avian species that are commonly related to meat bioassessment and biomonitoring.

**Results:**

In this project, we found the meat composition from 15 species could not be identified with the metabarcoding approach because of the lack of universal primers or insufficient discrimination power. Consequently, we developed and evaluated a meat mitochondrial metagenomics (3MG) method. The 3MG method has four steps: (1) extraction of sequencing reads from mitochondrial genomes (mitogenomes); (2) assembly of mitogenomes; (3) mapping of mitochondrial reads to the assembled mitogenomes; and (4) biomass estimation based on the number of uniquely mapped reads. The method was implemented in a python script called 3MG. The analysis of simulated datasets showed that the method can determine contaminant composition at a proportion of 2% and the relative error was < 5%. To evaluate the performance of 3MG, we constructed and analysed mixed samples derived from 15 animal species in equal mass. Then, we constructed and analysed mixed samples derived from two animal species (pork and chicken) in different ratios. DNAs were extracted and used in constructing 21 libraries for next-generation sequencing. The analysis of the 15 species mix with the method showed the successful identification of 12 of the 15 (80%) animal species tested. The analysis of the mixed samples of the two species revealed correlation coefficients of 0.98 for pork and 0.98 for chicken between the number of uniquely mapped reads and the mass proportion.

**Conclusion:**

To the best of our knowledge, this study is the first to demonstrate the potential of the non-targeted 3MG method as a tool for accurately estimating biomass in meat mix samples. The method has potential broad applications in meat product safety.

**Supplementary Information:**

The online version contains supplementary material available at 10.1186/s12864-021-08263-0.

## Background

Meat represents a significant portion of daily human consumption. However, meat adulteration has become a global issue. Valuable and expensive meat, such as beef and mutton, is often detected mixed with cheaper chicken, duck, pork, mink and animal meat [1, 2]. For instance, two of the nine beef samples examined by Erol et al. contained horse and deer meat [3]. Such adulteration harms consumers’ rights and interests [4] and disrupts market order [5]. Therefore, identifying adulterated ingredients in meat and meat products is essential.

Based on next-generation DNA sequencing, many methods for determining the biological composition of mixed samples have been developed, including metabarcoding [6], metagenomics [7, 8] and mitochondrial metagenomics (MMG) [9]. The metabarcoding approach depends on the PCR amplification of a particular marker for species determination. The metagenomics approach consists of two steps for species determination and biomass quantification, namely, shotgun sequencing and mapping of read to whole nuclear genomes. MMG is essentially a metagenomic method using mitochondrial genomes (mitogenome) instead of nuclear genomes as references. The PCR amplification-dependent metabarcoding method is the workhorse for the molecular determination of biological composition.

Numerous markers have been tested on animals, including 18S rRNA genes from the nuclear genome, 16S rRNA gene and cytochrome c oxidase I (COX1, CO1 or COI) gene from the mitogenome [10]. However, these PCR-dependent methods have limitations. Firstly, they require universal primers targeting particular markers, usually lacking across all taxa [11]. Different sets of universal markers and primer pairs complicate data integration when different markers are used, and different primer pairs are used for the same markers. Secondly, even with universal primers, template DNA molecules with different sequences have different melting properties, leading to amplification bias [12]. Consequently, the direct quantification of template DNA molecules with different sequences is difficult.

All-Food-Seq (AFS) is a recently developed metagenomics method [8], in which the non-targeted deep sequencing of total genomic DNA from foodstuff, followed by bioinformatics analysis, can identify species from all kingdoms of life with high accuracy. It facilitates the quantitative measurement of the main ingredients and detection of unanticipated food components. Conceptually, the AFS method has set up a framework for ultimate bio-surveillance.

However, the AFS method has several practical limitations. Firstly, the method is probably extremely complex for bioassessment and biomonitoring because a whole genome has a high degree of complexity. Secondly, although whole-genome databases have expanded rapidly, obtaining high-quality whole-genome sequences for a species requires many years. The effect of genomic diversity on bioassessment and biomonitoring is unknown. Thirdly, this study used simulated data rather than experimental data.

MMG delimits closely related species from mixed samples [13, 14]. This method is desirable because of its advantages. Firstly, a mitogenome and its genes are common phylogenetic, DNA barcoding and metabarcoding markers. Secondly, the structures of mitogenomes are conserved, whereas sequences can be highly diverse. Thirdly, mitogenomes are small and easy to obtain and can be directly reconstructed using bioinformatics methods. Fourthly, large numbers of mitogenomes are available in public databases. More than 10,000 mitogenomes have been included in the GenBank in December 2020. The performance and accuracy of metabarcoding and MMG in biomass estimation in invertebrate community samples have been evaluated [15]. Overall, MMG yields more informative predictions of biomass content from bulk macroinvertebrate communities than metabarcoding. However, despite that MMG has been applied to ecological assessment [9, 16–21], the use of MMG in mammalian and avian meat mixed samples have not been examined to the best of our knowledge.

In this study, we intended to use either metabarcoding or MMG to detect the potential mixing of meat from 15 mammalian and avian species on the basis of a market survey. Preliminary studies suggested that the most commonly used metabarcoding markers, COI and 16S, are unsuitable for simultaneously detecting meat from these 15 species. Thus, we tested MMG in mixed meat samples. This approach, called ‘meat mitochondrial mitogenome (3MG)’, circumvent the problem of marker selection, PCR bias and sequencing bias. Additionally, this approach takes advantage of the availability of mitogenomes for many species. The results showed that it can accurately determine the biological composition of meat mix samples and accurately estimate biomass. The method has a wide range of applications in food and pharmaceutical industries involving animal products.

## Materials and methods

### Meat samples and mock mixed meat samples

We prepared mock samples with meat from the legs of 15 mammalian and avian species: *Anas platyrhynchos* (duck), *Bos taurus* (cattle), *Camelus bactrianus* (camel), *Canis lupus familiaris* (dog), *Equus caballus* (horse), *Gallus gallus* (chicken), *Mus musculus* (mouse), *Mustela putorius voucher* (ferret), *Myocastor coypus* (nutria), *Nyctereutes procyonoides* (raccoon dog), *Oryctolagus cuniculus* (rabbit), *Ovis aries* (sheep), *Rattus norvegicus* (rat), *Sus scrofa domesticus* (pig) and *Vulpes vulpes* (fox). Efforts had been made to extract meat samples with homogenous compositions intraspecificly and interspecificly. We obtained camel, nutria, fox, donkey and deer meat from breeding farms. Nanjing Medical University provided the mouse, rabbit and rat samples. The Entry-exit Inspection and Quarantine Bureau provided other meat samples. The detailed information regarding sample origin, particularly cities and institutions, is provided in Table 1.

**Table 1:** Information for meat samples used in this study

Sample ID	Species		Source (Organisation, City, Province, Country)
	Common name	Latin name	
S01R1^a^, S01R2, S01R3	Duck	*Anas platyrhynchos*	Jiangsu EEIQB^b^, Nanjing City, Jiangsu Province, China
S02R1, S02R2, S02R3	Cattle	*Bos taurus*	Jiangsu EEIQB, Nanjing City, Jiangsu Province, China
S03R1, S03R2, S03R3	Camel	*Camelus bactrianus*	The Breeding Farm of Camel, Alxa League, Inner Mongolia, China
S04R1, S04R2, S04R3	Dog	*Canis lupus familiaris*	Jiangsu EEIQB, Nanjing City, Jiangsu Province, China
S05R1, S05R2, S05R3	Horse	*Equus caballus*	Xinjiang EEIQB, Urumqi City, Xinjiang, China
S06R1, S06R2, S06R3	Chicken	*Gallus gallus*	Jiangsu EEIQB, Nanjing City, Jiangsu Province, China
S07R1, S07R2, S07R3	Mouse	*Mus musculus*	Nanjing Medical University, Nanjing City, Jiangsu Province, China
S08R1, S08R2, S08R3	Ferret	*Mustela putorius voucher*	Jiangsu EEIQB, Nanjing City, Jiangsu Province, China
S09R1, S09R2, S09R3	Nutria	*Myocastor coypus*	The breeding farm of nutria, Chongqing City, China
S10R1, S10R2, S10R3	Raccoon dog	*Nyctereutes procyonoides*	Jiangsu EEIQB, Nanjing City, Jiangsu Province, China
S11R1, S11R2, S11R3	Rabbit	*Oryctolagus cuniculus*	Nanjing Medical University, Nanjing City, Jiangsu Province, China
S12R1, S12R2, S12R3	Sheep	*Ovis aries*	Jiangsu EEIQB, Nanjing City, Jiangsu Province, China
S13R1, S13R2, S13R3	Rat	*Rattus norvegicus*	Nanjing Medical University, Nanjing City, Jiangsu Province, China
S14R1, S14R2, S14R3	Pork	*Sus scrofa domesticus*	Jiangsu EEIQB, Nanjing City, Jiangsu Province, China
S15R1, S15R2, S15R3	Fox	*Vulpes vulpes*	The breeding farm of fox, Suihua City, Heilongjiang Province, China

^a^*S* Species, *R* Replicate

^b^*EEIQB* Entry-exit Inspection and Quarantine Bureau

Two methods were used in mixing the samples. One mix contained meat samples in equal amounts from 15 species. This mix was referred to as the ‘mix containing meat from 15 species’ or ‘M1’. The other mix contained meat from *S. scrofa domesticus* (pig) and *G. gallus* (chicken) in the following proportions: 10:0 (‘sample 1; mix containing two species’ or ‘M2-S1’), 8:2 (M2-S2), 6:4 (M2-S3), 4:6 (M2-S4), 2:8 (M2-S5) and 0:10 (M2-S6). Each M1 or M2 sample has three replicates.

### Loop-mediated isothermal amplification (LAMP)

We performed loop-mediated isothermal amplification (LAMP) experiments to validate the composition of mock samples (M1 and M2). LAMP methods for detecting ingredients that contain cattle, sheep, pig, chickens and duck meat were developed by the Technology center of Xiamen Entry-Exit Inspection and Quarantine Bureau of the People’s Republic of China [22–24]. The probe and primer sequences target *cytB* genes from the corresponding species were provided in Table S1. The PCR reaction mix contained isothermal master mix (15 μL), primer mix (FIP, 2 μL; BIP, 2 μL; F3, 1 μL; B3, 1 μL) and DNA (1 μL). We added RNase-free water to the final reaction of 50 μL. The experimental conditions were as follows: amplification at 60 °C for 90 min and annealing from 98 °C to 80 °C at a rate of 0.05 °C per second.

### DNA extraction, library construction and next-generation sequencing (NGS)

We extracted genomic DNA with a modified sodium dodecyl sulfate (SDS)-based method [25]. The integrity and concentration of the extracted DNA were detected through electrophoresis in 1% (w/v) agarose gel and spectrophotometer (Nanodrop 2000; Thermo Fisher Scientific, USA). The extracted DNA samples (100 ng) were subjected to library construction using NEBNext® Ultra™ II DNA library prep kit for Illumina® (New England BioLabs, USA) according to the manufacturer’s recommendations. Each library had an insert size of 500 bp. The quantity and quality of the libraries were analysed using Agilent 2100 Bioanalyser (Agilent Technologies, USA). We sequenced the libraries using the HiSeq X reagent kits (Illumina, USA) in an Illumina Hiseq X sequencer. We deposited the data generated in this study in GenBank. The accession numbers were SRR9107560 and SRR9140737.

### Construction of mitogenome reference databases

We constructed a database (15MGDB), which had complete mitogenome sequences from the 15 species. The 15 mitogenome sequences were downloaded from GenBank, with the following accession numbers: *A. platyrhynchos* (NC_009684), *B. taurus* (NC_006853), *C. bactrianus* (NC_009628), *C. lupus familiaris* (NC_002008), *E. caballus* (NC_001640), *G. gallus* (NC_001323), *M. musculus* (NC_005089), *M. putorius voucher* (NC_020638), *M. coypus* (NC_035866), *O. cuniculus* (NC_001913), *O. aries* (NC_001941), *N. procyonoides* (NC_013700), *R. norvegicus* (NC_001665), *S. scrofa domesticus* (NC_012095), *V. vulpes* (NC_008434). The sequences in 15MGDB were used in constructing a searchable database with the makeblastdb command from the BLAST+ (v2.7.1) software package [26] and with the option ‘-dbtype nucl -parse_seqids’.

### Development of the 3MG analysis pipeline

The 3MG pipeline was developed using Python 2.7.15 with the following third-party software applications: pandas module in python, BBMap (v35.66; https://sourceforge.net/projects/bbmap/), MITOBim (v1.9.1) [27], Blast+ (v2.7.1) [26], bowtie2 (v2.3.4) [28] and samtools (v1.9) [29]. The source code, sample data and instruction for using the locally installed copy of the 3 mg pipeline and a singularity container for running the 3 mg pipeline can be found using the following link: http://1kmpg.cn/3mg/.

### Determination of 3MG detection errors using simulation

We generated 21 sets of data through simulation. Reads from an M2-S1 sample containing 100% pork was used as the background. Reads were then extracted from the reads of M2-S6 containing only chicken with the seqtk program (v1.3-r106) and with the option ‘seqtk sample -s100’. The reads extracted from M2-S6 were mixed with those from M2-S1 in the following percentages: 0.01, 0.1, 1, 2, 3, 4, 5, 6, 7, 8, 9, 10, 20, 30, 40, 50, 60, 70, 80, 90 and 100%. We prepared five replicates of simulated data for each percentage level and used default seeds. The resulting sample sets were then analysed using the 3MG pipeline. We calculated relative detection errors with the following formula as described previously [8].

$$\mathrm{Relative}\ \mathrm{error}= \mid \left(\mathrm{Number}\ \mathrm{of}\ \mathrm{chicken}\ \mathrm{reads}\ \mathrm{detected}-\mathrm{Number}\ \mathrm{of}\ \mathrm{chicken}\ \mathrm{reads}\ \mathrm{in}\ \mathrm{the}\ \mathrm{sample}\right) \mid /\left(\mathrm{Number}\ \mathrm{of}\ \mathrm{chicken}\ \mathrm{reads}\ \mathrm{in}\ \mathrm{the}\ \mathrm{sample}\right)$$

### Comparison of the reference and assembled mitogenomes

We aligned the assembled sequences with their reference sequences for each species using the CLUSTALW2 (v2.0.12) program [30] with option ‘-type = dna -output = phylip’. We used these aligned sequences in constructing phylogenetic trees with the maximum likelihood (ML) method implemented in RaxML (v8.2.4) [31]. We calculated the intra-specific and inter-specific distances among mitogenomes using the distmat program from EMBOSS (v6.3.1) [28] with the options ‘-nucmethod = 0’. Corrections for multiple substitutions cannot be made through this method. Finally, we calculated the p-distances among mitogenomes with MEGA (v7) [32].

### Detection of other contaminating biological composition

Taxon content in reads unmapped to mitogenomes were analysed using the RDP classifier (v2.12) [33]. The unmapped reads were assigned to COX1 and 16S rRNA database with an assignment confidence cutoff of 0.8. The 16S rRNA database is part of the RDP Classifier package. The COX1 database was downloaded from https://github.com/terrimporter/CO1Classifier/releases/tag/v3.2 [34]. The results were visualised using MEGAN (v6) [35] with the following LCA parameters: ‘minSupportPercent = 0.02, minSupport = 1, minScore = 50.0, maxExpected = 0.01, topPercent = 10.0 and readAssignmentMode = readCount’.

## Results

### Evaluation of the metabarcoding method for the 15 mammalian and avian species

To determine whether the mixture containing 15 species can be identified using metabarcoding, we analysed the availability of universal primers and the ability of their amplified products (if applicable) to distinguish the 15 species. For the COX1 gene, no primer matched the sequences from all the species. For instance, the maximum number of matched species was five when the primer pair I-B1 and COI-C04 was used (Table S2). For the 16S rRNA gene, only one primer set, 16Sbr-H, matched the sequences of all the species, and the amplified products showed high degrees of variations that were sufficient for distinguishing the 15 species (Fig. S1). For the 18S rRNA, only the primer Uni18S was found in the sequences of all the species, but the amplified products were highly conserved and could not be used in distinguishing the 15 species (Fig. S2). Previously, the performance of COI metabarcoding and that of shotgun mitogenome sequencing were compared. Shotgun sequencing can provide highly significant correlations between read number and biomass [17]. As a result, we focused on developing the metagenomic approach for the direct biomass estimation of meat samples from the 15 species.

### Development of 3MG method

**Fig. 1:**
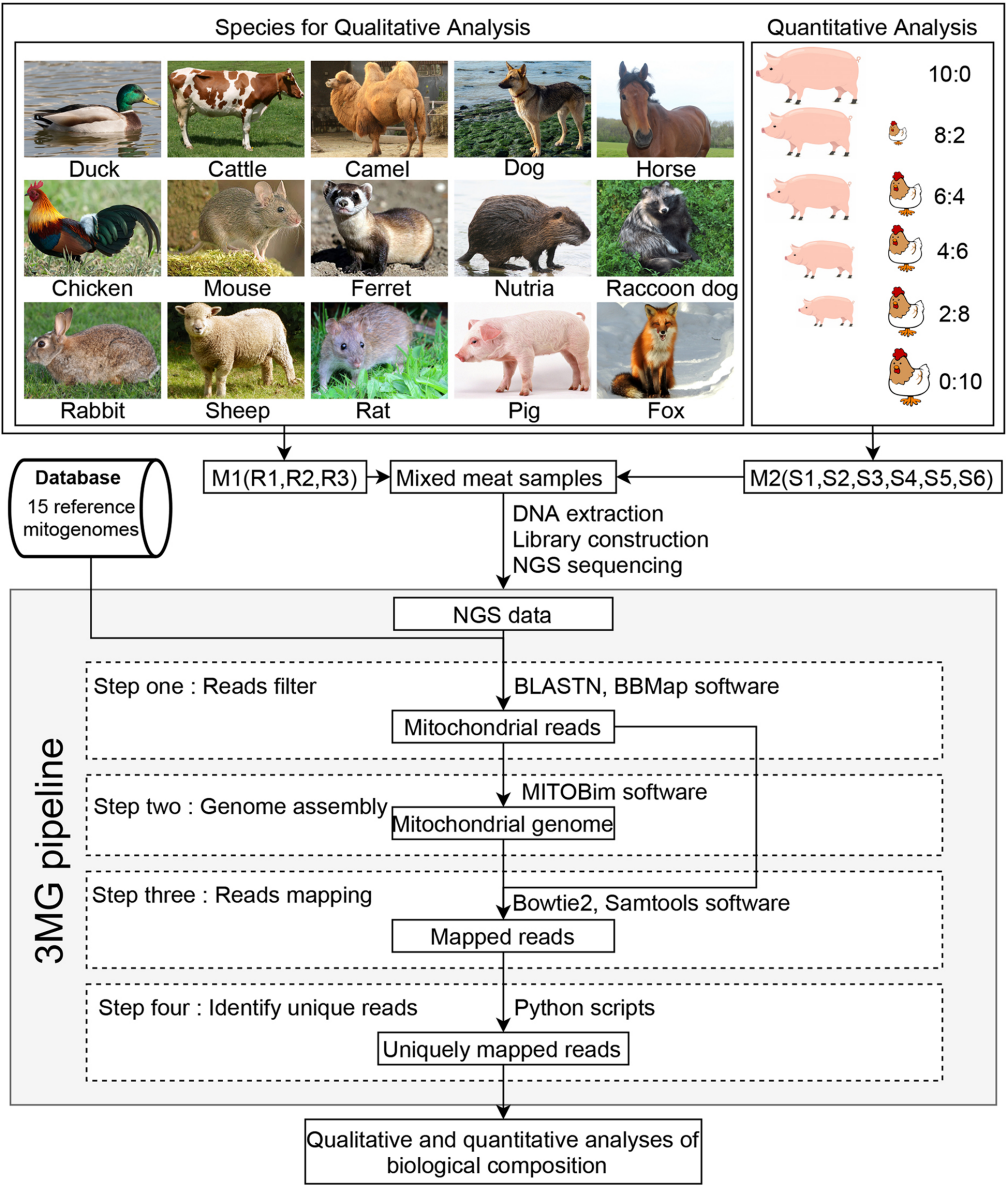
Flow chart of the 3MG pipeline. The 15 species used for the qualitative analyses and the setup of meat from the two species for the quantitative analyses are shown on the top. The four steps are labeled as S1, S2, S3 and S4. The results of each step are shown in the black rectangle. The third-party tools are shown on the right side of the corresponding process

The 3MG pipeline can be divided into four steps (Fig. 1). The first step is ‘extracting mitochondrial reads’. We searched next-generation sequencing (NGS) reads against 15MGDB by using the BLASTN command with the following parameters: -evalue = 1e-5 and –outfmt = 6. We extracted the matched reads using the ‘filterbyname.sh’ command in the BBMap software package (v35.66). The extracted reads were called ‘mitochondrial reads’ and used in the subsequent procedures.

The second step is assembling mitogenomes from mitochondrial reads. The mitogenomes in the public database might have originated from a particular individual or subspecies. Thus, the sequences from the samples might differ from those in the public database because of intra-specific variations. To ensure accurate qualitative and quantitative analyses, we assembled the mitogenomes according to the NGS reads and used MITOBim (v1.9.1) [27] with the default parameters. The mitogenome sequences downloaded from GenBank were used as references. They were called reference mitogenomes in the subsequent text.

The third step is mapping reads to the assembled mitogenomes. We mapped the reads to each assembled sequence of the species with bowtie2 (v2.3.4) [28], using default parameters. We then extracted the mapped reads using samtools (v1.9) [29] with the following command: ‘samtools view -bF 4.’

The fourth step is identifying and counting reads uniquely mapped to the assembled mitogenomes. The mitogenome sequences were highly conserved. Some reads may be mapped to the mitogenomes of multiple species. We calculated the p-distances among these mitogenomes to determine how conserved they were. The p-distances among the 15 mitogenomes ranged from 0.14 to 0.49 (Fig. S3). These numbers indicated a high degree of mitogenome sequence conservation. All the mapped reads may have originated from multiple sources. To overcome this problem, we developed a custom python script to remove non-specific reads. Specifically, we obtained 15 files recording the mapped reads of each species. We compared the mapped reads of the target specie with those of other 14 species and deleted non-specific reads appeared multiple files. The proportions of unique reads mapped to the mitogenome of a particular species in all mitochondrial reads were calculated. When the proportion was greater than 2% (the cutoff of 2% was set according to the results of Determination of detection sensitivity for 3MG methods based on simulated datasets section), the species was called ‘presence’.

### Determination of detection sensitivity for 3MG methods based on simulated datasets

We constructed 21 simulated datasets (Table 2, SD01-SD21) mixed with 30,000 pork (major composition) and chicken mitochondrial reads (minor composition) to determine detection sensitivity. The percentages of chicken mitochondrial reads were 0.01, 0.1, 1, 2, 3, 4, 5, 6, 7, 8, 9, 10, 20,30, 40, 50, 60, 70, 80, 90 and 100% of all the mitochondrial reads in the simulated dataset. We prepared five replicates at each proportion. We analysed the data using the 3MG analysis pipeline. We calculated the relative detection error at each proportion (Table 2). At a high proportion, the quantitative detected results were similar to the simulation results. At 2–100%, we detected the minor composition with a relative error of < 5%. However, the accuracy was significantly reduced at 1, 0.1 and 0.01%. These results indicated that the method can detect a contaminant at a proportion of 2% and has an error rate of < 5%.

**Table 2:** Relative detection errors determined using the simulated dataset

Sample ID	No. of Reads Added in the SD^a^		No. of Unique Reads Detected as Chicken using 3MG method						Relative errors±STD
	Total No. of Reads	No. of Chicken Reads	R1^b^	R2	R3	R4	R5	Average	
SD01	30,000	3 (0.01%)	30	31	29	31	29	30	900.00% ± 29.81%
SD02	30,000	30 (0.1%)	57	58	56	57	55	56.6	88.67% ± 3.40%
SD03	30,000	300 (1%)	323	322	316	322	321	320.8	6.93% ± 0.83%
SD04	30,000	600 (2%)	621	620	610	617	614	616.4	2.73% ± 0.67%
SD05	30,000	900 (3%)	916	918	909	912	909	912.8	1.42% ± 0.41%
SD06	30,000	1200 (4%)	1211	1213	1207	1207	1206	1208.8	0.73% ± 0.23%
SD07	30,000	1500 (5%)	1505	1507	1503	1502	1502	1503.8	0.25% ± 0.13%
SD08	30,000	1800 (6%)	1801	1804	1802	1801	1798	1801.2	0.07% ± 0.11%
SD09	30,000	2100 (7%)	2099	2100	2098	2094	2093	2096.8	0.15% ± 0.13%
SD10	30,000	2400 (8%)	2397	2396	2393	2391	2390	2393.4	0.27% ± 0.11%
SD11	30,000	2700 (9%)	2694	2691	2687	2686	2686	2688.8	0.41% ± 0.12%
SD12	30,000	3000 (10%)	2991	2986	2985	2975	2982	2983.8	0.54% ± 0.18%
SD13	30,000	6000 (20%)	5950	5945	5934	5934	5943	5941.2	0.98% ± 0.11%
SD14	30,000	9000 (30%)	8912	8898	8895	8898	8900	8900.6	1.10% ± 0.07
SD15	30,000	12,000 (40%)	11,850	11,859	11,860	11,859	11,870	11,859.6	1.17% ± 0.05%
SD16	30,000	15,000 (50%)	14,812	14,818	14,817	14,824	14,825	14,819.2	1.21% ± 0.03%
SD17	30,000	18,000 (60%)	17,781	17,790	17,795	17,779	17,777	17,784.4	1.20% ± 0.04%
SD18	30,000	21,000 (70%)	20,752	20,753	20,757	20,741	20,738	20,748.2	1.20 ± 0.04%
SD19	30,000	24,000 (80%)	23,713	23,726	23,721	23,716	23,709	23,717	1.18% ± 0.02%
SD20	30,000	27,000 (90%)	26,671	26,684	26,684	26,686	26,678	26,680.6	1.18 ± 0.02%
SD21	30,000	30,000 (100%)	29,633	29,651	29,629	29,652	29,646	29,642.2	1.19% ± 0.03%

^a^*SD* Simulated Dataset

^b^*R1-R5* Five replicates were prepared for each dataset

### Qualitative determination of biological composition with the 3MG method

#### Sequencing results and characterisation

We constructed mixed samples containing meat from 15 animals (M1). The animals fall into several categories. For example, pork, cattle, chicken, lamb, duck and rabbit are primarily used for human consumption. Ferret, nutria, raccoon dog and fox are commonly used in the fur industry. Dogs are companion animals. Camel and horse are used for multiple purposes. Rat and mouse co-inhabit with humans and their meat can potentially contaminate other meat for human consumption. Adulteration of meat not meant for human consumption have been reported, particularly through the addition of meat of fur animals to pork or beef or substitution of pork with horse meat [2]. The motivation is primarily economic gain, as profits can be made when expensive meat is replaced with cheap meat. Some of these adulterations can be culturally offensive, for example, adding pork in food for Islamic consumers [36, 37].

We constructed three M1 samples labeled as ‘R1’, ‘R2’ and ‘R3’, respectively. We obtained 23.45, 24.10 and 28.56 GB for each of the three replicates (Table 3). The percentages of bases having Quality scores ≥30 were 89.51, 88.97 and 89.97%.

**Table 3:** Summary of sequencing data for samples M1 and M2

Mix Sample ID	Raw Reads	Raw Bases	Clean Reads	Clean Bases	Error Rate	Q20	Q30	GC Content
M1-R1	78,172,066	23.45G	77,978,481	23.39G	0.02%	95.63%	89.51%	42.56%
M1-R2	80,340,353	24.10G	80,207,468	24.06G	0.03%	95.42%	88.97%	42.47%
M1-R3	95,203,148	28.56G	94,898,093	28.47G	0.02%	95.88%	89.97%	42.66%
M2-S1-R1	19,408,898	2.91G	18,733,774	2.81G	0.02%	95.72%	90.10%	43.53%
M2-S1-R2	19,548,720	2.93G	18,822,412	2.82G	0.02%	95.74%	90.16%	43.57%
M2-S1-R3	20,038,386	3.00G	19,283,538	2.89G	0.02%	95.54%	89.78%	43.07%
M2-S2-R1	20,918,540	3.13G	20,371,134	3.06G	0.02%	95.77%	90.26%	42.00%
M2-S2-R2	23,322,230	3.49G	22,772,368	3.42G	0.02%	96.03%	90.75%	42.39%
M2-S2-R3	20,064,544	3.00G	19,474,596	2.92G	0.02%	95.77%	90.25	42.35%
M2-S3-R1	18,390,256	2.75G	17,857,850	2.68G	0.02%	95.97%	90.66%	42.22%
M2-S3-R2	22,069,572	3.31G	21,472,386	3.22G	0.02%	96.30%	91.30%	42.11%
M2-S3-R3	22,361,728	3.35G	21,802,258	3.27G	0.02%	96.29%	91.32%	42.08%
M2-S4-R1	17,632,194	2.64G	17,169,806	2.58G	0.01%	97.20%	93.23%	42.18%
M2-S4-R2	20,086,894	3.01G	19,574,120	2.94G	0.02%	96.31%	91.32%	42.07%
M2-S4-R3	18,892,514	2.83G	18,223,384	2.73G	0.02%	95.70%	90.11%	43.85%
M2-S5-R1	17,744,966	2.66G	16,989,830	2.55G	0.02%	95.62%	90.03%	43.82%
M2-S5-R2	19,047,898	2.85G	18,376,398	2.76G	0.02%	95.61%	90.02%	43.38%
M2-S5-R3	18,449,648	2.76G	17,690,002	2.65G	0.02%	95.67%	90.15	43.50%
M2-S6-R1	18,881,556	2.83G	18,154,952	2.72G	0.02%	95.62%	90.05%	43.48%
M2-S6-R2	20,351,422	3.05G	19,725,574	2.96G	0.02%	95.95%	90.70%	42.51%
M2-S6-R3	18,283,292	2.74G	17,799,550	2.67G	0.02%	95.74%	90.27	42.38%

*M1* A mix of 15 species, *M2* A mix of two species chicken and pig; proportions of the chicken and pork are 10:0 (M2-S1), 8:2 (M2-S2), 6:4 (M2-S3), 4:6 (M2-S4), 2:8 (M2-S5) and 0:10 (M2-S6). R1-R3: Three replicates were prepared for each mixed sample

#### Qualitative analysis of M1 sample’s biological composition

**Fig. 2:**
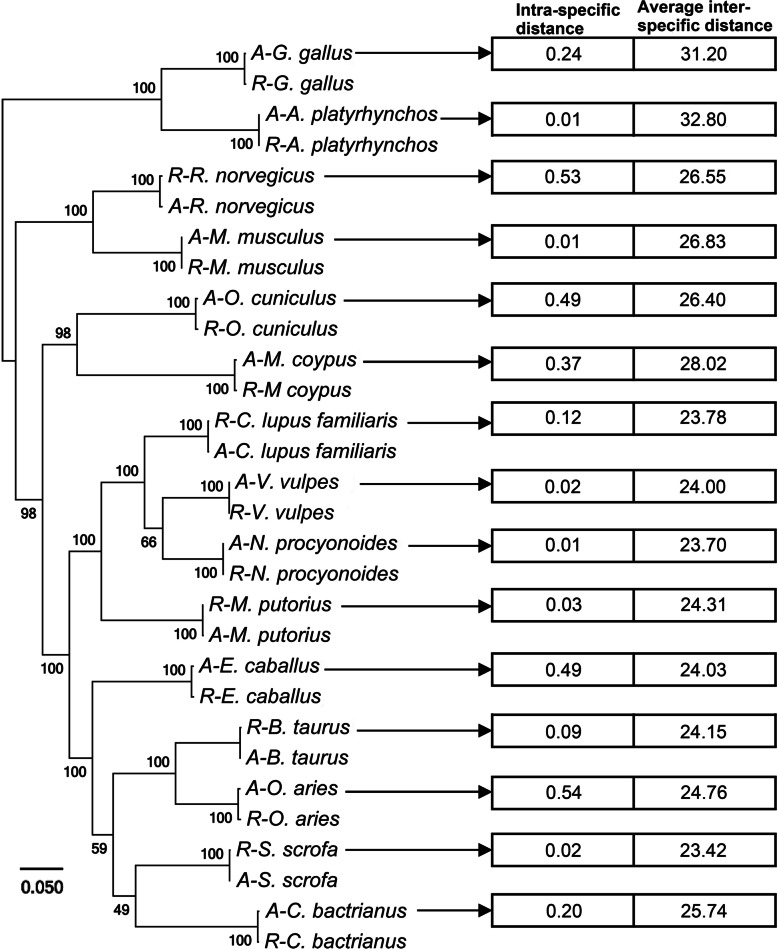
Phylogenetic analysis of the reference (‘R’) and assembled (‘A’) mitogenomes. The intra-specific and inter-specific nucleotide distances of mitogenomes are shown in the squares to the right of the tree. The intra-specific nucleotide distance was calculated between the reference and assembled mitogenomes for the same species. We calculated the inter-specific nucleotide distances between each of the 14 pairs of mitogenomes. Each pair consisted of one focal mitogenome and a mitogenome. The average inter-specific distances are shown

We analysed the NGS data generated from the M1 samples using the pipeline 3MG. In step one, 331,866 (0.43%), 222,702 (0.28%) and 267,495 (0.28%) reads were mapped to the mitogenomes and were extracted as mitochondrial reads (Table 4). In step two, we successfully assembled all 15 mitogenomes from mitochondrial reads. We constructed a phylogenetic tree using 15 pairs of assembled and reference mitogenomes (Fig. 2). The alignment of the 15 pairs of mitogenomes is shown in Fig. S4. The reference and assembled mitogenomes for the same species were clustered together (left part of Fig. 2).

**Table 4:** Summary of reads mapped to the mitogenomes of the 15 species

Sample ID	Species	Mix Sample ID	Total No. of read mapped to a particular mitogenome	No. of unique reads mapped to a particular mitogenomes (proportion)	The proportion of unique reads to all mitochondrial reads
S01-R1	*A. platyrhynchos*	M1-R1	17,350	13,548(78.09%)	4.08%
S01-R2		M1-R2	16,381	13,560(82.78%)	6.09%
S01-R3		M1-R3	35,170	31,664(90.03%)	11.84%
S02-R1	*B. taurus*	M1-R1	19,884	3904(19.63%)	1.18%
S02-R2		M1-R2	10,746	1840(17.12%)	0.83%
S02-R3		M1-R3	16,668	3988(23.93%)	1.49%
S03-R1	*C. bactrianus*	M1-R1	15,221	5330(35.02%)	1.61%
S03-R2		M1-R2	23,387	16,319(69.78%)	7.33%
S03-R3		M1-R3	25,856	17,560(67.91%)	6.56%
S04-R1	*C. lupus familiaris*	M1-R1	78,416	37,962(48.41%)	11.44%
S04-R2		M1-R2	51,085	25,057(49.50%)	11.25%
S04-R3		M1-R3	48,068	21,793(45.34%)	8.15%
S05-R1	*E. caballus*	M1-R1	49,594	14,541(29.32%)	4.38%
S05-R2		M1-R2	22,297	8899(39.91%)	4.00%
S05-R3		M1-R3	42,016	11,305(26.91%)	4.23%
S06-R1	*G. gallus*	M1-R1	22,008	18,504(84.08%)	5.58%
S06-R2		M1-R2	14,801	12,181(82.30%)	5.47%
S06-R3		M1-R3	9380	6094(64.97%)	2.28%
S07-R1	*M. musculus*	M1-R1	11,717	4963(42.36%)	1.50%
S07-R2		M1-R2	8919	3896(43.68%)	1.75%
S07-R3		M1-R3	16,902	10,664(63.09%)	3.99%
S08-R1	*M. putorius voucher*	M1-R1	19,235	3157(16.41%)	0.95%
S08-R2		M1-R2	20,029	8916(44.52%)	4.00%
S08-R3		M1-R3	19,847	7767(39.13%)	2.90%
S09-R1	*M. coypus*	M1-R1	40,437	33,785(83.55%)	10.18%
S09-R2		M1-R2	29,178	24,375(83.54%)	10.95%
S09-R3		M1-R3	15,724	10,892(69.27%)	4.07%
S10-R1	*N. procyonoides*	M1-R1	56,355	16,435(29.16%)	4.95%
S10-R2		M1-R2	27,077	2348(8.67%)	1.05%
S10-R3		M1-R3	37,645	11,567(30.73)	4.32%
S11-R1	*O. cuniculus*	M1-R1	10,921	4016(36.77%)	1.21
S11-R2		M1-R2	6602	1647(24.95%)	0.74%
S11-R3		M1-R3	10,260	4778(46.57%)	1.79%
S12-R1	*O. aries*	M1-R1	21,377	7895(36.93)	2.38%
S12-R2		M1-R2	12,139	4084(33.64%)	1.83%
S12-R3		M1-R3	17,950	6879(38.32%)	2.57%
S13-R1	*R. norvegicus*	M1-R1	8875	1456(16.41%)	0.44%
S13-R2		M1-R2	8239	2710(32.89%)	1.22%
S13-R3		M1-R3	9832	3098(31.51%)	1.16%
S14-R1	*S. scrofa domesticus*	M1-R1	42,116	28,114(66.75%)	8.47%
S14-R2		M1-R2	20,722	12,040(58.10%)	5.41%
S14-R3		M1-R3	33,601	22,651(67.41%)	8.47%
S15-R1	*V. vulpes*	M1-R1	92,357	49,294(53.37%)	14.85%
S15-R2		M1-R2	62,656	35,156(56.11%)	15.79%
S15-R3		M1-R3	53,858	25,960(48.20%)	9.70%

To compare the intra-specific and inter-specific distances, we calculated the distances, as shown in the right part of Fig. 2. Intra-specific distance was the distance between the assembled and reference mitogenomes for a particular species. By contrast, inter-specific distance was the average distance between the assembled mitogenomes of the focal species and those of the other 14 species. Intra-specific nucleotide distances were much smaller than the inter-specific distances. Thus, we assembled the mitogenomes of specific species from the metagenomic data with high accuracy. Our assembled mitogenomes unlikely contained chimeric sequences because the inter-specific distances were significantly larger than the intra-specific distances.

In step three, we mapped these mitochondrial reads to the 15 assembled mitogenomes. Approximately 10,000–90,000 reads were mapped to each mitogenome (Table 4). However, an average of 52.07% reads was mapped to multiple species. Thus, using the total number of mapped reads led to the overestimation of the meat content of a particular species. For example, 79.77% of the reads mapped to the *B. taurus* (cattle) mitogenome were non-specific, and 63.70% of the reads mapped to the *O. aries* (sheep) mitogenome were non-specific. Using the total number of reads in estimating the beef and lamb content led to overestimation. Hence, 3MG determines biological composition according to the number of reads uniquely mapped to a particular mitogenome.

In step four, we identified reads uniquely mapped to the mitogenome of each species. The proportion of unique reads to all mitochondrial reads was more than 2% for 12 species in at least one replicate sample (Table 4). The mapped read proportions for *B. taurus*, *O. cuniculus* and *R. norvegicus* were approximately 1%. In summary, through the analysis of the 15 species mix with this 3MG pipeline, 12 of 15 (80%) species were successfully identified with a confidence level of 95%.

#### Validation of 3MG analysis results for M1 samples by using LAMP experiments

**Fig. 3:**
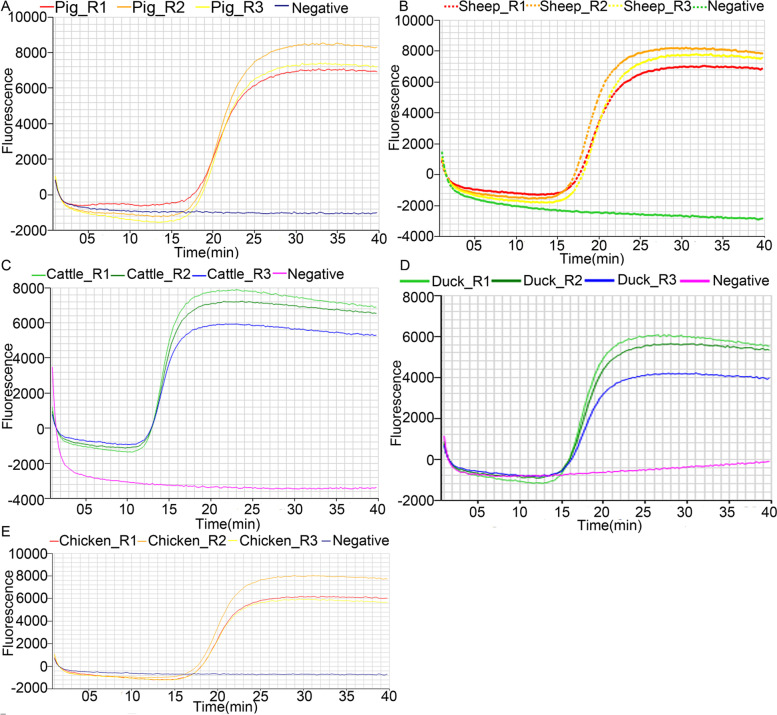
Composition determination of the M1 samples with the LAMP method. The X-axis represents time, whereas the Y-axis represents relative fluorescence abundance. Each species was tested in three replicates shown in different colors

LAMP is commonly used in detecting the biological composition of meat products. We used LAMP results to evaluate the accuracy of the 3MG results. Unfortunately, LAMP protocols are available for the meat of only five of 15 species (pig, sheep, cattle, duck and chicken). Thus, only these five species in the M1 samples were tested. The experiments were conducted separately for each target species (Fig. 3). The results confirmed the presence of meat from pig (Fig. 3A), sheep (Fig. 3B), cattle (Fig. 3C), duck (Fig. 3D) and chicken (Fig. 3E) in our experimental samples, consistent with the results obtained from the 3MG method.

### Quantitative determination of biological composition in mixed sample

#### Sequencing results and characteristics

To determine the performance of 3MG in estimating the biological composition in biomass, we prepared a series of samples by mixing meat from *S. scrofa domesticus* (pig) and *G. gallus* (chicken) in different proportions. We performed DNA extraction, library construction, DNA sequencing and DNA analyses in the same way as those for the M1 samples. The sequencing results are summarised in Table 3. We generated an average of 2.95 GB of data with 19,749,625 raw reads for each M2 sample. Approximately 90% of bases had quality scores greater than 30.

#### Quantitative analysis of M2 samples’ biological composition

**Fig. 4:**
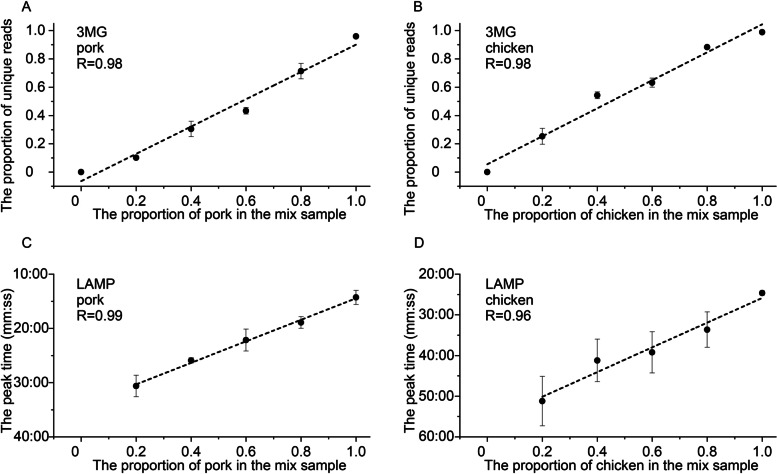
Results of quantitative analysis using the 3MG and LAMP for two species. **A** and **B** Quantitative analysis results of the 3MG for a combination of two species. The X-axis shows the mass proportions of pork (**A**) and chicken (**B**) in the mix samples. The Y-axis shows the proportions of reads uniquely mapped to pork (**A**) and chicken (**B**) mitogenomes from mixed samples. The R-value represents the correlation coefficient between the proportions of uniquely mapped reads and the mass proportions of the samples. **C** and **D** Results of quantitative analysis using LAMP for the mixed samples of two species. The Y-axis shows the peak times for detecting pork (**C**) and chicken (**D**) components in the mix samples. The R-value represents the correlation coefficient between peak time and the mass proportion in the mixed samples

The number of reads mapped to the pig and chicken mitogenomes were shown in Table S2. The proportion of reads uniquely mapped to the pig mitogenomes was called meat content estimated with 3MG. They were plotted against the known meat content (Fig. 4A). Regression analyses showed that the pork’s estimated and known meat content had a correlation coefficient of 0.98. Similarly, based on relative read counts, the estimated meat content for chicken were plotted against known meat content (Fig. 4B). Regression analyses showed that the estimated and known meat content had a correlation coefficient of 0.98. The high correlation coefficient between the estimated and known content suggested that the 3MG method can use the percentage of uniquely mapped reads in quantitatively determining biological composition in a meat mix.

#### Validation of 3MG analysis results using LAMP experiments

We conducted a LAMP experiment to determine the quantity of pork and chicken in different ratios. We then compared the LAMP results with those obtained from the 3MG method. The peak time for detecting composition was called meat content estimated by LAMP and plotted against the known meat content of pig (Fig. 4C) and chicken (Fig. 4D). Regression analyses showed that the estimated and known meat content had correlation coefficients of 0.99 (pork) and 0.96 (chicken). Consequently, the 3MG results were consistent with the LAMP results. However, the variations in the LAMP results for chicken were significantly higher than those in the 3MG results. This observation suggested that the 3MG results were more stable than the LAMP results, at least for chicken meat.

### Estimation of the relative correction factor for DNA–biomass ratio from different species

We mixed the meat of 15 species to construct M1 samples in equal mass ratios as described earlier. However, the number of reads mapped to each mitogenome of the 15 species varied significantly (Table 4). This discrepancy was likely due to the differences in mitogenome DNA content among the 15 species at the same meat biomass. As a result, a correction factor was needed when the meat mass was estimated from uniquely mapped read counts for a particular species. Using the number of reads uniquely mapped to the *S. scrofa domesticus* mitogenome as the baseline, we calculated the relative correction factors for the other species. The correction factors were 1.00 for *A. platyrhynchos*, 0.77 for *C. bactrianus*, 1.46 for *C. lupus familiaris*, 0.59 for *E. caballus*, 0.65 for *G. gallus*, 0.32 for *M. musculus*, 0.40 for *M. putorius voucher*, 1.24 for *M. coypus*, 0.43 for *N. procyonoides*, 0.31 for *O. aries* and 1.94 for *V. vulpes*. This set of relative correction factors might correlate with the relative copy numbers of mitogenome in the muscle tissues of each species. They can be used in estimating meat content for different species. Detailed discussions on the ratios of nuclear DNA to mitochondrial DNA and DNA to biomass are provided in the following text.

### Detection of other contaminating biological composition

**Fig. 5:**
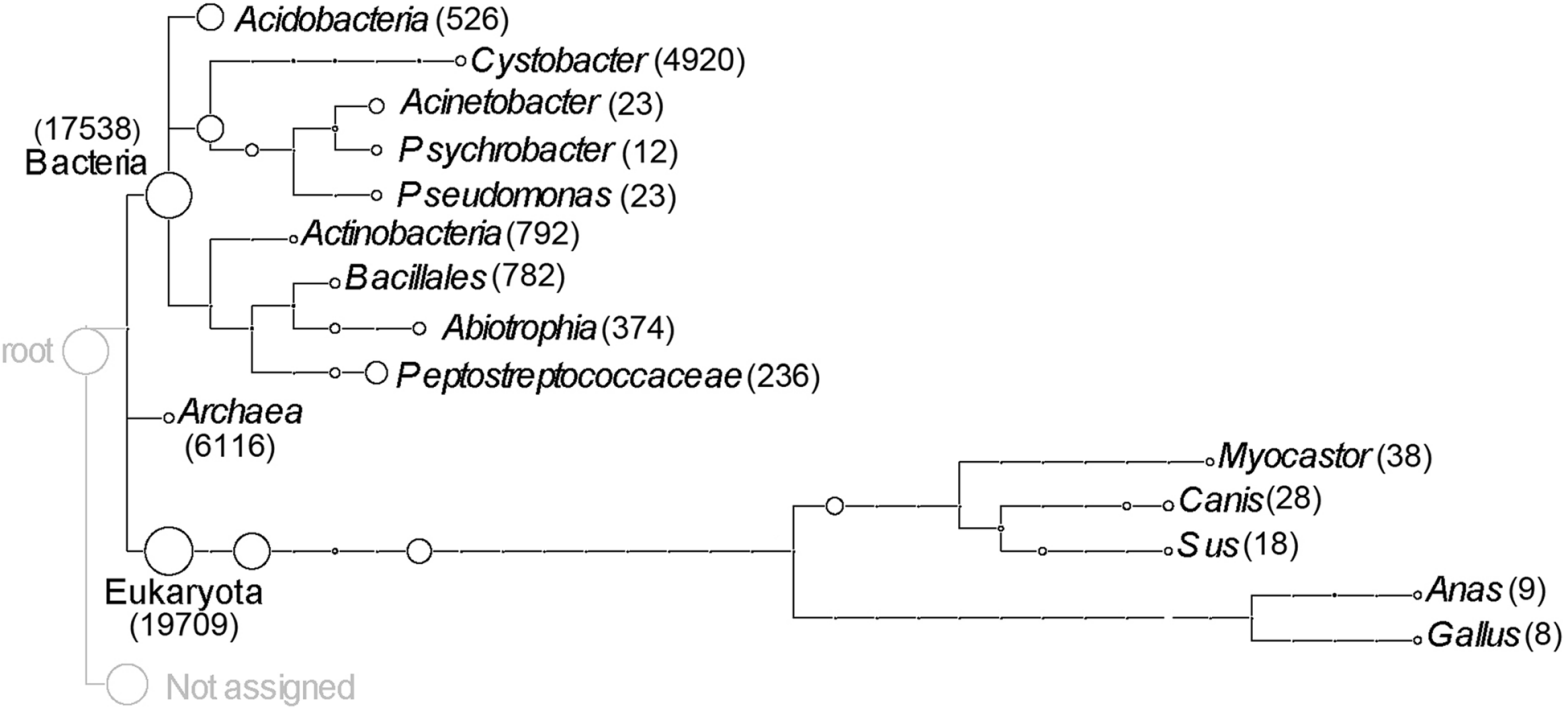
Analyses of reads unused by the 3MG. The phylogram shows the taxa at the genus level at which reads were mapped. Each circle of the tree represents a taxon labeled by its name and the number of reads assigned to it. The size of the circle represents the proportion of reads aligned to the taxon and cannot be aligned to a lower level of the taxon

To determine the presence of unexpected biological composition in the samples, we classified the unmapped reads with the RDP classifier and analysed them using MEGAN6. The unmapped reads can be divided into four categories: bacteria, Archaea, Eukaryota and ‘not assigned’ (Fig. 5). Five genera belonging to Eukaryota were annotated: *Myocastor*, *Canis*, *Sus*, *Anas* and *Gallus*. These reads may have been extremely diverse and thus were not mapped to the mitogenomes in the 3MG process. Overall, we detected few contaminants from other mammals and bacteria in our mock mix samples.

## Discussions

Meat adulteration and contamination can affect consumers’ well-being, disrupt market order and insult religious beliefs. Hence, the development of qualitative, quantitative and unbiased methods for detecting the composition of meat products is of great importance. In the present study, we found that meat composition from 15 species cannot be identified with the metabarcoding approach because of the lack of universal primers or the needed discrimination power. Therefore, we developed a meat mitochondrial metagenomics (3MG) method to determine the composition of 15 meat most commonly found in food markets.

The evaluation of detection sensitivity for the 3MG methods based on simulated datasets indicated that the method can detect a contaminating composition at a proportion of 2% and has an error rate of < 5%. This method successfully identified the presence of 12 of 15 (80%) species with the threshold of detection sensitivity. This result showed that the method can simultaneously detect the presence of multiple species with high sensitivity. It can detect a wide variety of adulterated meat in the market. In addition, the analyses of the two species mixed samples revealed correlation coefficients of 0.98 for pork and 0.98 for chicken between the number of uniquely mapped reads and the mass proportion. The 3MG results were more stable than the LAMP results, at least for chicken meat, indicating that the method can use the percentage of uniquely mapped reads in quantitatively determining biological composition in a meat mix.

To the best of our knowledge, this study is the first to demonstrate the usefulness of the mitochondrial metagenomics method in detecting meat composition and estimating biomass. This method has several advantages over methods based on PCR amplification and particular markers. It is a non-targeted approach and does not need to assume the biological composition of samples. Consequently, it is likely to have a lower false-negative detection rate. Given that PCR-based methods require species primers, they often fail to amplify sequences not matched by primers. Furthermore, the 3MG method is not affected by problems in PCR reactions, such as the generation of multiple PCR bands resulting from non-specific amplification. The detection of multiple composition with PCR-based methods requires simultaneous PCR reactions specific to multiple biological composition. This approach can be quite expensive. By contrast, the 3MG methods can potentially reduce the cost in this case. The 3MG method may facilitate the analysis of high-value products, such as medical and health-promoting products. In general, the 3MG method is suitable for non-targeted biomonitoring and requires meat composition with an abundance above specific levels, whereas PCR-based method is suitable for targeted biomonitoring and can detect biological composition at considerably low abundance levels.

We showed that the mitogenome DNAs of the 15 mammalian and avian species represent 0.28–0.43% of the total DNA. The generation of 1 GB of data costs around US$ 10, and 1 GB of data can produce sufficient mitochondrial reads for determining biological composition qualitatively and quantitatively. Mitogenomes from animals are relatively small and easy to assemble. In December 2020, more than ten thousand animal mitogenomes had been deposited in the NCBI RefSeq database (https://www.ncbi.nlm.nih.gov/genome/browse#!/organelles/). Thus, expecting that all species used in food and medicine will have their mitogenomes available soon is reasonable. Owing to the rapid drop in sequencing costs, fast accumulation of mitogenomes and the integrated bioinformatics software tools, we can expect the broad application of the 3MG method in the near term.

One problem encountered in this study was that beef was successfully detected using LAMP but was not detected in the mock sample when the 3MG method was used. One explanation is that the cattle mitogenome sequence has relatively small percentages of unique sequences. Our data showed that only 17.12–23.93% of reads mapped to the cattle mitogenome were unique to the cattle mitogenome (Table 4). LAMP primers were unique enough to amplify the cattle sequence successfully. Hence, the proportion of unique regions on a mitogenome is essential for its successful detection with the 3MG method. In addition, rabbit or rat meat was not successfully detected with the 3MG method. One explanation is that the mitochondrial DNA has a low proportion of all cellular DNA. Our data showed that the total number of reads mapped to the mitochondrial genomes of these two species was significantly lower than those mapped to the other species (Table 4). Additional studies are needed to optimise the 3MG for the detection of such species mixed samples. Several improvements can be made for the 3MG method. Firstly, internal controls can be added to the samples for the accurate determination of the amount of mito-DNA for particular animal species. As described previously [14, 38], the internal controls can be commonly used as metabarcoding markers, particularly COI and 18S. As the lack of universal primers prevent these markers in metabarcoding analysis, they should represent perfect sequences serving as internal controls for 3MG analysis. Secondly, correction factors should be estimated for biomass estimation based on read counts. For meat biomonitoring, biomass is more commonly used than read counts. Thus, an appropriate conversion rate from read count to the biomass for each meat type is needed. It should be emphasized that the sampling locations may affect the results of biomass estimation. For example, meat from different locations of the legs might have different ratios of fat and fibers, resulting in the variations in the DNA extraction rate and the nuclear to mitochondrial DNA ratio. In this study, we tried to extract samples with homogeneous compositions intraspecificly and interspecificly to minimize this effect. The sample heterogeneity problem is difficult to solve not only for the 3MG method, but also for other traditional detection methods in general. Therefore, we need to estimate two types of correction factors. The first one is the nuclear to mitochondrial DNA ratio (also known as the nuclear–mito ratio). The DNA to biomass ratio (DNA-mass ratio) should be calculated as well. Given that meat might contain different proportions of fats, a high degree of variations in nuclear–mito ratio and DNA–mass ratio are expected among different species. Thirdly, many studies have focused on the meat from 15 mammalian and avian species used in food safety biomonitoring. Meat from many other animal species is commonly consumed but has not been tested in the current study. For instance, fish represents another large group of animal meat widely consumed. The 3MG methods developed in the current study can be applied to fish meat in theory. Parameter optimisation and validation of 3MG on the assessment of fish meat are interesting subjects. Lastly, we should build an extensive reference database for unique mitogenomic DNA sequences from different varieties of related animal species given that many animals, such as chickens, pigs, cattle and sheep, have many endemic species. Building an extensive database containing variety-specific mitochondrial genome sequences will facilitate the identification of the sources of particular animal species.

The current research has laid the foundation for developing accurate and standard procedures for detecting the composition of meat qualitative and quantitatively. The methods will be necessary for the bioassessment and biomonitoring of meat products worldwide and significantly contribute to meat safety management.

## Supplementary Information

**Additional file 1: Table S1.** The primer sequences used for the LAMP experiments. **Table S2.** Analysis of universal primers for COX1, 16S rRNA, and 18S rRNA genes. **Table S3.** Summary of reads mapped to the mitogenomes of *S. scrofa domesticus* and *G. gallus*. **Fig. S1.** The distribution of universal primers on 16 s rRNA sequences. **Fig. S2.** The distribution of universal primers on 18 s rRNA sequences. **Fig. S3.** The pairwise p-distance of the 15 mitogenomes sequences. **Fig. S4.** Alignment of reassembled sequences of mitogenomes and those downloaded from GenBank for 15 species. The prefix “A” and “R” represent the assembled and reference mitogenomes, respectively.

## Data Availability

The Next Generation Sequencing data for this research is available on the SRA database (https://www.ncbi.nlm.nih.gov/sra). The accession number of a mixture of 15 commonly used animal meat raw sequence reads in Biosample is SAMN11812028. The raw data can be download with the SRA accessions number SRR9107560.
