# Supplementary material for: Development and evaluation of a meat mitochondrial metagenomic (3MG) method for composition determination of meat from fifteen mammalian and avian species
Source: BMC Genomics. 2022 Jan 7;23:36. doi: 10.1186/s12864-021-08263-0 (PMC8742424; doi:10.1186/s12864-021-08263-0)
Supplement: Supplementary file 1 — Additional file 1: Table S1. The primer sequences used for the LAMP experiments. Table S2. Analysis of universal primers for COX1, 16S rRNA, and 18S rRNA genes. Table S3. Summary of reads mapped to the mitogenomes of S. scrofa domesticus and G. gallus. Fig. S1. The distribution of universal primers on 16 s rRNA sequences. Fig. S2. The distribution of universal primers on 18 s rRNA sequences. Fig. S3. The pairwise p-distance of the 15 mitogenomes sequences. Fig. S4. Alignment of reassembled sequences of mitogenomes and those downloaded from GenBank for 15 species. The prefix “A” and “R” represent the assembled and reference mitogenomes, respectively. [file 12864_2021_8263_MOESM1_ESM.docx]

Supplementary material

**Development and evaluation of a** **meat mitochondrial metagenomic (3MG) method for composition determination of meat from fifteen mammalian and avian species**

Mei Jiang^1^, Shu-Fei Xu^2^, Tai-Shan Tang^3^, Li Miao^4^, Bao-Zheng Luo^5^, Yang Ni^6^, Fan-De Kong^2#^, Chang Liu^1#^

^1^Key Laboratory of Bioactive Substances and Resource Utilization of Chinese Herbal Medicine from the Ministry of Education, Institute of Medicinal Plant Development, Chinese Academy of Medical Sciences, Peking Union Medical College, Beijing 100193, PR China

^2^Technology Center of Xiamen Entry-exit Inspection and Quarantine Bureau, Xiamen, Fujian 361026, PR China

^3^Technology Center of Jiangsu Entry-exit Inspection and Quarantine Bureau, Nanjing, Jiangsu 210009, PR China

^4^Technology Center of Henan Entry-exit Inspection and Quarantine Bureau, Zhengzhou, Henan 450003, PR China

^5^Technology Center of Zhuhai Entry-exit Inspection and Quarantine Bureau, Zhuhai, Guangdong 519000, PR China

^6^College of Agriculture, Fujian Agriculture and Forestry University, Fuzhou, Fujian Province, Fuzhou 350002, PR China

^#^Corresponding Authors:

CL: Tel: +86-10-57833111; Fax: +86-10-62899715; Email: cliu6688@yahoo.com.

FDK: Tel: +86-592-6806048; Fax: +86-592-6806061; Email: kfd67@sina.com

Email Address：

MJ: [mjiang0502@163.com](mailto:mjiang0502@163.com)

SFX: xusf6050@163.com

TST: [56194644@qq.com](mailto:tangts@jsciq.gov.cn)

LM: [ml5628@163.com](mailto:hmchen@implad.ac.cn)

BZL: [bzluo@163.com](mailto:lys832000@163.com)

YN: ny_work@126.com

FDK: kfd67@sina.com

CL: [cliu6688@yahoo.com](mailto:cliu6688@yahoo.com)

**Table S1** The primer sequences used for the LAMP experiments

| Species | Gene | Sequences |
| --- | --- | --- |
| Chicken | CytB | F3: 5´-CATCCAACATCTCTGCTTGA-3´ |
|  |  | B3: 5´-GAGTGTGAGGAGGAGGATTA-3´ |
|  |  | FIP: 5´-TCAGCCGTATTGTACGTTCCGCTAGCCATGCACTACACAG-3´ |
|  |  | BIP: 5´-CATCCGGAATCTCCACGCAACGTCCGATGTGAAGGAAG-3´ |
|  |  | LoopF: 5´-TACGGAGGAGAAGGCTAGG-3´ |
|  |  | LoopB: 5´-CGCCTCATTCTTCTTCATCTGT-3´ |
| Duck | CytB | F3: 5´-GAGTAATCCTACTGCTCACTC-3´ |
|  |  | B3: 5´-GCCTGATTCGTGTAGGAAG-3´ |
|  |  | FIP: 5´-TTACGGTAGCTCCTCAGAACGATTATAGCAACTGCCTTCGTAG-3´ |
|  |  | BIP: 5´-ACCCTGGTAGAATGAGCCTGATGAATGGCGAAGAATCGG-3´ |
|  |  | LoopF: 5´-TCCTCATGGCAGGACATAAC-3´ |
|  |  | LoopB: 5´-GGAGGATTCTCAGTGGATAACC-3´ |
| Cattle | CytB | F3: 5´-TATCGGAGTAATCCTTCTGCT-3´ |
|  |  | B3: 5´-GGAATAATAGGTGGACTATGGC-3´ |
|  |  | FIP: 5´-TTGGTGATGACTGTTGCTCCTCCACAGTAATAGCCACAGCAT-3´ |
|  |  | BIP: 5´-AGCAATCCCATACATCGGCACGTAAGGGTTGCTTTGTCTACT-3´ |
|  |  | LOOPF: 5´-CCTCATGGTAGGACGTATCCTA-3´ |
|  |  | LOOPB: 5´-ATGAATCTGAGGCGGATTCTC-3´ |
| Sheep | CytB | F3: 5´-ACAATAGCCACAGCATTCAT-3´ |
|  |  | B3: 5´-ATCTGTGTCCGATGGAATTC-3´ |
|  |  | FIP: 5´-TCATTCGACTAGGTTTGTGCCACATTCTGAGGAGCAACAGTTA-3´ |
|  |  | BIP: 5´-CAAAGCTACCCTCACCCGATTTGTAGGTGAACTATGGCGAG-3´ |
|  |  | LoopF: 5´-ATTGCTGAAAGGAGGTTGGT-3´ |
|  |  | LoopB: 5´-CCATTCATCATCGCAGCC-3´ |
| Pork | CytB | F3: 5´-TCTTACTTCAGGACCATCTCA-3´ |
|  |  | B3: 5´-CCGGATCATGAGTTCCATG-3´ |
|  |  | FIP: 5´-ATGTGTGAGCATGGGCTGATTAAATCGCCCACTCTTTCC-3´ |
|  |  | BIP: 5´-GGGATGCTTAGACTCAGCCATGGTCCAGCTACAATTGATTTGAC-3´ |
|  |  | LoopF: 5´-AGTCATTAGTCCATCGAGATGT-3´ |
|  |  | LoopB: 5´-CCGTCAAAGGCCCTAACA-3´ |

**Table S2 Analysis of universal primers for COX1, 16S rRNA, and 18S rRNA genes**

|  | COX1 | | | | | | | |  | 16S | | | |  | 18S | | | |
| --- | --- | --- | --- | --- | --- | --- | --- | --- | --- | --- | --- | --- | --- | --- | --- | --- | --- | --- |
| Primer name | LCO1490 | HC02198 | I-B1 | I-130R | LepF1 | LEP-R1 | COI-C02 | COI-C04 |  | L2513 | H2714 | 16Sar-L | 16Sbr-H |  | Uni18S | Uni18SR | Uni18S2 | Uni18SR2 |
| Primer direction | F | R | F | R | F | R | F | R |  | F | R | F | R |  | F | R | F | R |
| *A. platyrhynchos* | |  |  |  |  |  |  |  |  |  |  |  | + |  | + | + | + | + |
| *B. taurus* |  |  | + |  |  |  |  | + |  | + | + | + | + |  | + | + | + | + |
| *C. bactrianus* |  |  |  |  |  |  |  |  |  | + | + | + | + |  | + | + |  | + |
| *C. lupus familiaris* | |  |  |  |  |  |  |  |  | + | + | + | + |  | + | + | + | + |
| *E. caballus* |  |  | + |  |  |  |  | + |  | + | + | + | + |  | + |  |  |  |
| *G. gallus* |  |  |  |  |  |  |  |  |  |  |  |  | + |  | + | + | + | + |
| *M. musculus* |  | + | + |  |  | + |  | + |  | + | + | + | + |  | + | + | + | + |
| *M. putorius voucher* | |  |  |  |  |  |  |  |  | + | + | + | + |  | + | + | + | + |
| *M. coypus* |  | + | + |  |  | + |  | + |  | + | + | + | + |  | + | + | + | + |
| *N. procyonoides* | |  |  |  |  |  |  |  |  | + | + | + | + |  | + | + | + | + |
| *O. cuniculus* |  |  |  |  |  |  |  |  |  | + | + | + | + |  | + | + |  | + |
| *O. aries* |  | + | + |  |  |  |  | + |  | + | + | + | + |  | + | + | + | + |
| *R. norvegicus* |  |  |  |  |  |  |  |  |  | + | + | + | + |  | + | + | + | + |
| *S. scrofa* |  |  |  |  |  |  |  |  |  | + | + | + | + |  | + | + | + | + |
| *V. vulpes* |  |  |  |  |  |  |  |  |  | + | + | + | + |  | + | + | + | + |

“F”: forward primer; “R”: reverse primer; “+”: primers were found matching the sequence.

**Table S3** Summary of reads mapped to the mitogenomes of *S. scrofa domesticus* and *G. gallus*.

| ID | Sample ID | Mass Ratio (pork: chicken) | Total No. of mitochondrial reads | Pork | | Chicken | |
| --- | --- | --- | --- | --- | --- | --- | --- |
|  |  |  |  | No. of Unique reads | Percentage of Unique reads to mitochondrial reads | No. of Unique reads | Percentage of Unique reads to mitochondrial reads |
| 1 | M2-S1-R1 | 0:10 | 32815 | 30 | 0.09% | 31728 | 96.69% |
| 2 | M2-S1-R2 | 0:10 | 21837 | 8 | 0.04% | 20787 | 95.19% |
| 3 | M2-S1-R3 | 0:10 | 25181 | 15 | 0.06% | 24096 | 95.69% |
| 4 | M2-S2-R1 | 2:8 | 33795 | 8933 | 26.43% | 23780 | 70.37% |
| 5 | M2-S2-R2 | 2:8 | 34351 | 6163 | 17.94% | 26973 | 78.52% |
| 6 | M2-S2-R3 | 2:8 | 29467 | 9266 | 31.45% | 19198 | 65.15% |
| 7 | M2-S3-R1 | 4:6 | 31294 | 16159 | 51.64% | 14327 | 45.78% |
| 8 | M2-S3-R2 | 4:6 | 42850 | 22972 | 53.61% | 18855 | 44.00% |
| 9 | M2-S3-R3 | 4:6 | 38652 | 22235 | 57.53% | 15527 | 40.17% |
| 10 | M2-S4-R1 | 6:4 | 36504 | 24687 | 67.63% | 10772 | 29.51% |
| 11 | M2-S4-R2 | 6:4 | 47480 | 28685 | 60.41% | 17862 | 37.62% |
| 12 | M2-S4-R3 | 6:4 | 47692 | 29314 | 61.47% | 11591 | 24.30% |
| 13 | M2-S5-R1 | 8:2 | 36158 | 32201 | 89.06% | 3461 | 9.57% |
| 14 | M2-S5-R2 | 8:2 | 40860 | 35672 | 87.30% | 4556 | 11.15% |
| 15 | M2-S5-R3 | 8:2 | 39761 | 35258 | 88.67% | 3896 | 9.80% |
| 16 | M2-S6-R1 | 10:0 | 47160 | 46588 | 98.79% | 29 | 0.06% |
| 17 | M2-S6-R2 | 10:0 | 52055 | 51491 | 98.92% | 11 | 0.02% |
| 18 | M2-S6-R3 | 10:0 | 48850 | 48347 | 98.97% | 19 | 0.04% |


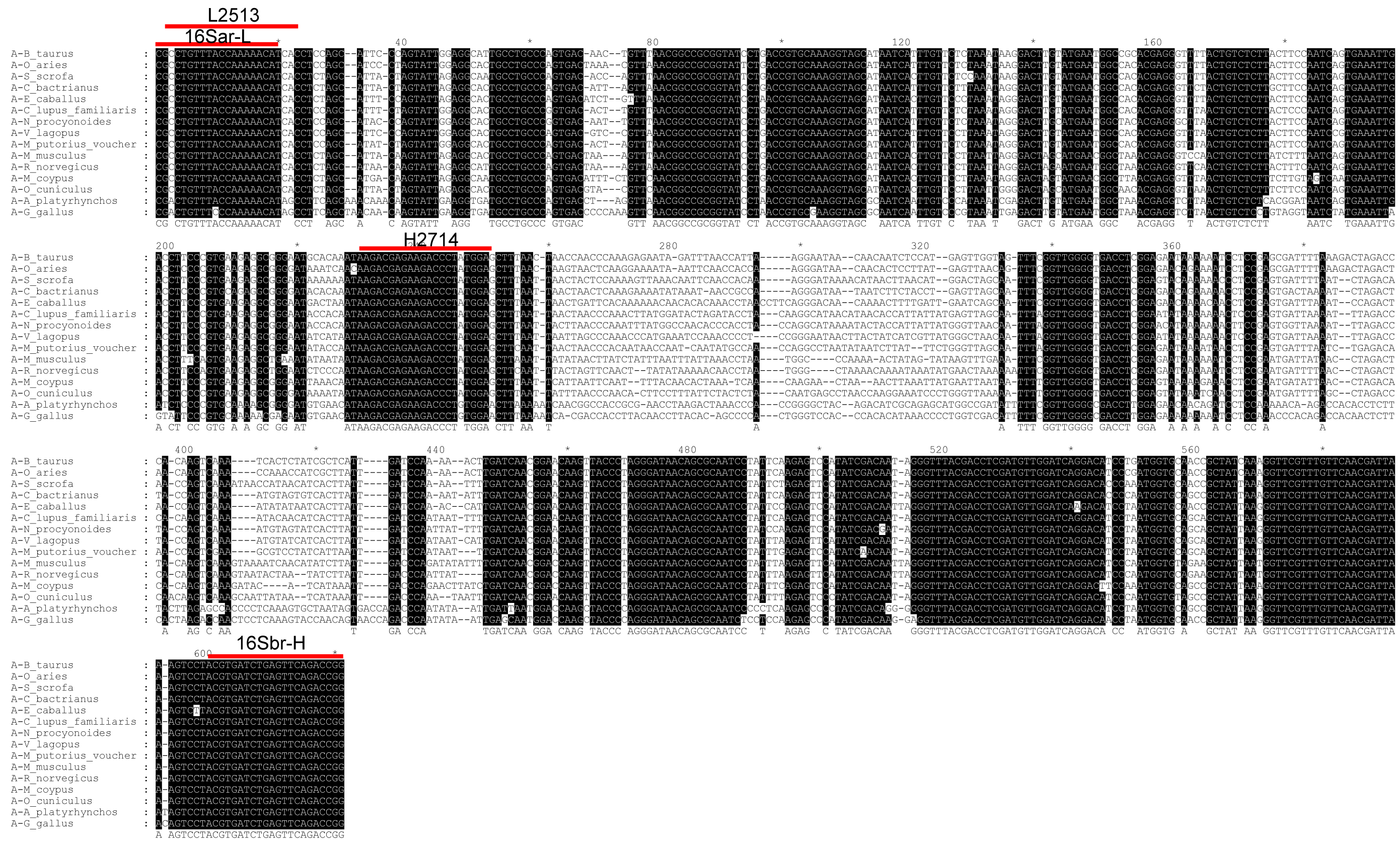


**Fig. S1 The distribution of universal primers on 16s rRNA sequences.**


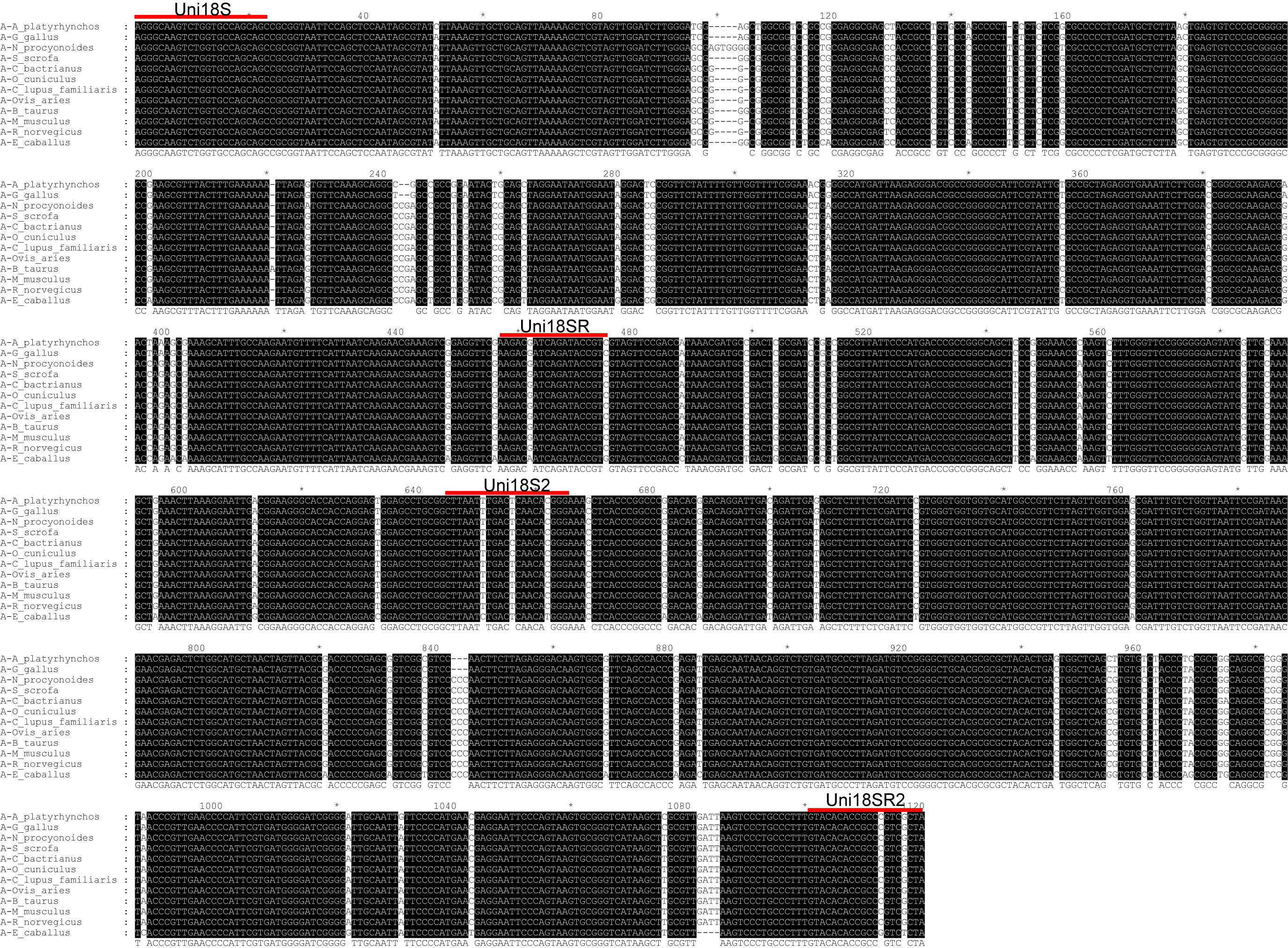


**Fig. S2 The distribution of universal primers on 18s rRNA sequences.**

**Fig. S3** The pairwise p-distance of the 15 mitogenomes sequences.

**
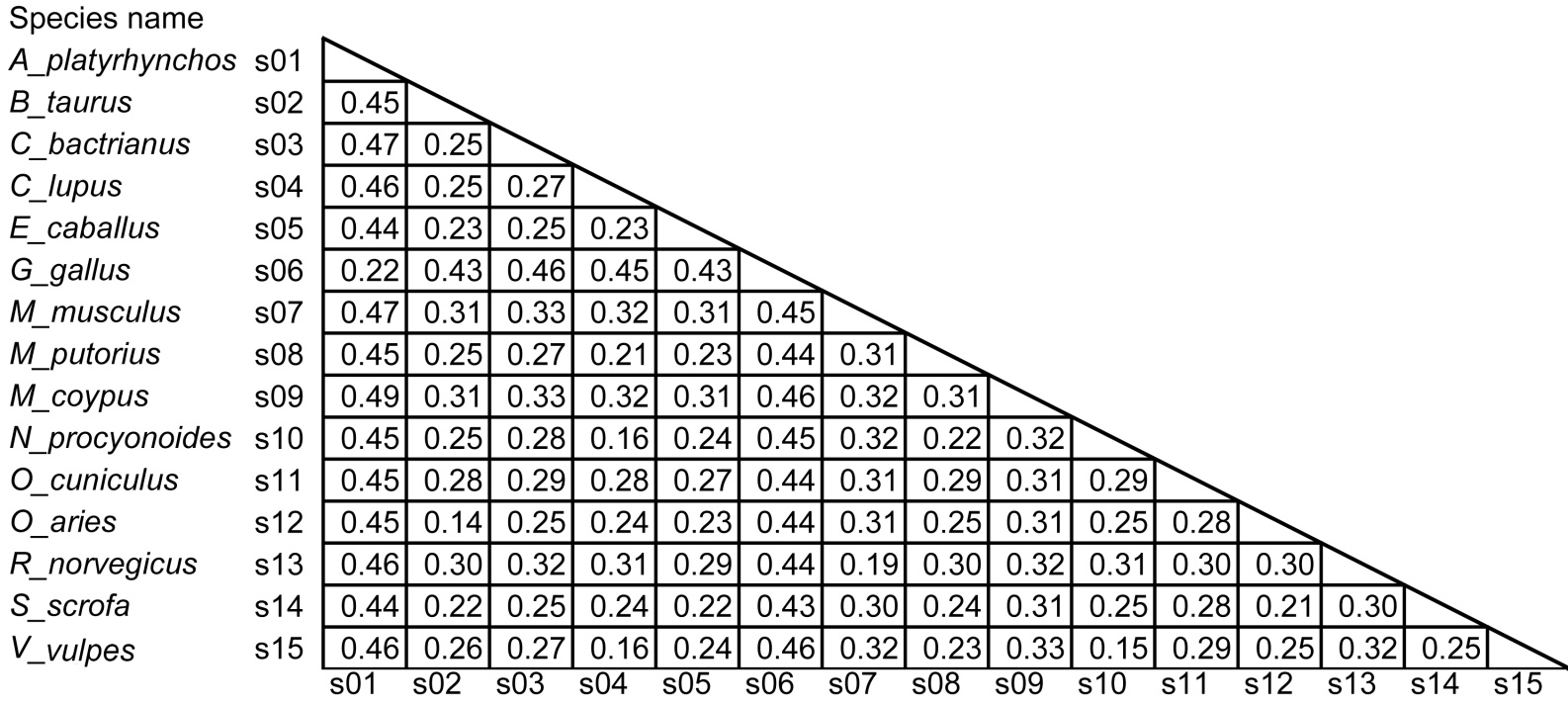
**

**Fig. S4** Alignment of reassembled sequences of mitogenomes and those downloaded from GenBank for 15 species. The prefix “A” and “R” represent the assembled and reference mitogenomes, respectively.

* 20 * 40 * 60 * 80 * 100 * 120 * 140 * 160
R-E_caballus : ----------------------------------------------------------------------------------------------------------------------------------------------------------------
A-E_caballus : ----------------------------------------------------------------------------------------------------------------------------------------------------------------
R-C_bactrianus : ----------------------------------------------------------------------------------------------------------------------------------------------------------------
A-C_bactrianus : ----------------------------------------------------------------------------------------------------------------------------------------------------------------
R-C_lupus_familiaris : ----------------------------------------------------------------------------------------------------------------------------------------------------------------
A-C_lupus_familiaris : ----------------------------------------------------------------------------------------------------------------------------------------------------------------
R-N_procyonoides : ----------------------------------------------------------------------------------------------------------------------------------------------------------------
A-N_procyonoides : ----------------------------------------------------------------------------------------------------------------------------------------------------------------
R-V_lagopus : ----------------------------------------------------------------------------------------------------------------------------------------------------------------
A-V_lagopus : ----------------------------------------------------------------------------------------------------------------------------------------------------------------
R-M_putorius : ----------------------------------------------------------------------------------------------------------------------------------------------------------------
A-M_putorius : ----------------------------------------------------------------------------------------------------------------------------------------------------------------
R-B_taurus : ----------------------------------------------------------------------------------------------------------------------------------------------------------------
A-B_taurus : ----------------------------------------------------------------------------------------------------------------------------------------------------------------
R-O_aries : ----------------------------------------------------------------------------------------------------------------------------------------------------------------
A-O_aries : ----------------------------------------------------------------------------------------------------------------------------------------------------------------
R-S_scrofa : --CAACCAAAACAAGCATTCCATTCGTATGCAAACCAAAACGCCAAGTACTTAATTACTATCTTTAAAACAAAAAAACCCATAAAAATTGCGCACAAACATACAAATATGCGACCCCAAAAATTTAACCATTAAAAACAAAAAATTTAATATATTATAGC
A-S_scrofa : --CAACCAAAACAAGCATTCCATTCGTATGCAAACCAAAACGCCAAGTACTTAATTACTATCTTTAAAACAAAAAAACCCATAAAAATTGCGCACAAACATACAAATATGCGACCCCAAAAATTTAACCATTAAAAACAAAAAATTTAATATATTATAGC
R-O_cuniculus : ----------------------------------------------------------------------------------------------------------------------------------------------------------------
A-O_cuniculus : ----------------------------------------------------------------------------------------------------------------------------------------------------------------
R-M_musculus : ----------------------------------------------------------------------------------------------------------------------------------------------------------------
A-M_musculus : ----------------------------------------------------------------------------------------------------------------------------------------------------------------
R-R_norvegicus : ----------------------------------------------------------------------------------------------------------------------------------------------------------------
A-R_norvegicus : ----------------------------------------------------------------------------------------------------------------------------------------------------------------
R-M_coypus : ----------------------------------------------------------------------------------------------------------------------------------------------------------------
A-M_coypus : ----------------------------------------------------------------------------------------------------------------------------------------------------------------
R-A_platyrhynchos : AGCTAGAAT----AGCCTAATAATGCTCTCAGGACCCCCCCCCCCTTCCCCCCCC-AGGGGTTGCGGGGTTATTTGGTTATGCATA-TCGTGCATACATTTATATTCCCCATATATTAACCTATGGTCCCGGTAATAAACACTAT-TA----ACCAA--C
A-A_platyrhynchos : AGCTAGAAT----AGCCTAATAATGCTCTCAGGACCCCCCCCCCCTTCCCCCCCCCAGGGGTTGCGGGGTTATTTGGTTATGCATA-TCGTGCATACATTTATATTCCCCATATATTAACCTATGGTCCCGGTAATAAACACTAT-TA----ACCAA--C
R-G_gallus : AATTTTATTTTTTAACCTAACTCCCCTACTAAGTGTACCCCCCCTTTCCCC-----AGGGG------GGGTAT---ACTATGCATAATCGTGCATACATTTATATACCACATATATTA-----TGGTACCGGTAATATATACTATATAT-GTACTAAACC
A-G_gallus : AATTTTATTTTTTAACCTAACTCCCCTACTAAGTGTACCCCCCCTTTCCCCCCC--AGGGG------GGGTAT---ACTATGCATAATCGTGCATACATTTATATACCACATATATTA-----TGGTACCGGTAATATATACTATATAT-GTACTAAACC
 ARCTABAATWWYWAGCCTAACAHTCCTATBAAGACCACCCCCCCHTTCCCCCCCYTAGGGGYTKYRRGGBTATWWRACTATGCATAATCGTGCATACATTTATATACCHCATATATTAAMMWWTGGTCCCGGTAATAHAHACTATWTAWTRTACTAAASC

 * 180 * 200 * 220 * 240 * 260 * 280 * 300 * 320
R-E_caballus : ----------------------------------------------------------------------------------------------------------------------------------------------------------------
A-E_caballus : ----------------------------------------------------------------------------------------------------------------------------------------------------------------
R-C_bactrianus : ----------------------------------------------------------------------------------------------------------------------------------------------------------------
A-C_bactrianus : ----------------------------------------------------------------------------------------------------------------------------------------------------------------
R-C_lupus_familiaris : ----------------------------------------------------------------------------------------------------------------------------------------------------------------
A-C_lupus_familiaris : ----------------------------------------------------------------------------------------------------------------------------------------------------------------
R-N_procyonoides : ----------------------------------------------------------------------------------------------------------------------------------------------------------------
A-N_procyonoides : ----------------------------------------------------------------------------------------------------------------------------------------------------------------
R-V_lagopus : ----------------------------------------------------------------------------------------------------------------------------------------------------------------
A-V_lagopus : ----------------------------------------------------------------------------------------------------------------------------------------------------------------
R-M_putorius : ----------------------------------------------------------------------------------------------------------------------------------------------------------------
A-M_putorius : ----------------------------------------------------------------------------------------------------------------------------------------------------------------
R-B_taurus : ----------------------------------------------------------------------------------------------------------------------------------------------------------------
A-B_taurus : ----------------------------------------------------------------------------------------------------------------------------------------------------------------
R-O_aries : ----------------------------------------------------------------------------------------------------------------------------------------------------------------
A-O_aries : ----------------------------------------------------------------------------------------------------------------------------------------------------------------
R-S_scrofa : CCTATGTACGTCGTGCATTAACTGCTAGTCCCCATGCATATAAGCATGTACATATTATTATTAATATTACATAGTACATATCATTATTGATCGTACATAGCACATATCATGTCAAATAACTCCAGTCAACATGCGTATCACCACCATTAGATCACGAGCT
A-S_scrofa : CCTATGTACGTCGTGCATTAACTGCTAGTCCCCATGCATATAAGCATGTACATATTATTATTAATATTACATAGTACATATCATTATTGATCGTACATAGCACATATCATGTCAAATAACTCCAGTCAACATGCGTATCACCACCATTAGATCACGAGCT
R-O_cuniculus : ----------------------------------------------------------------------------------------------------------------------------------------------------------------
A-O_cuniculus : ----------------------------------------------------------------------------------------------------------------------------------------------------------------
R-M_musculus : ----------------------------------------------------------------------------------------------------------------------------------------------------------------
A-M_musculus : ----------------------------------------------------------------------------------------------------------------------------------------------------------------
R-R_norvegicus : ----------------------------------------------------------------------------------------------------------------------------------------------------------------
A-R_norvegicus : ----------------------------------------------------------------------------------------------------------------------------------------------------------------
R-M_coypus : ----------------------------------------------------------------------------------------------------------------------------------------------------------------
A-M_coypus : ----------------------------------------------------------------------------------------------------------------------------------------------------------------
R-A_platyrhynchos : TATCCTACATGCACGGAC-TAAACCCAT----CACATGTC----AACGGACATACCCTACCT-ATC--GGAC---------TACCCTCCCAATGGAC---CCAGAGTGAATGCTCTAATACCCAAC-----ACCTCAACACCACATAA----------CA
A-A_platyrhynchos : TATCCTACATGCACGGAC-TAAACCCAT----CACATGTC----AACGGACATACCCTACCT-ATC--GGAC---------TACCCTCCCAATGGAC---CCAGAGTGAATGCTCTAATACCCAAC-----ACCTCAACACCACATAA----------CA
R-G_gallus : CATTATATGTATACGGGCATTAACCTATATTCCACATTTCTCCCAATGTCCATTCTATGCATGATCTAGGACATACTCATTTACCCTCCCCATAGACAGTTCCAAACCACTATCAAGCCACCTAACTATGAATGGTTACAGGACATAAATCTCACTCTCA
A-G_gallus : CATTATATGTATACGGGCATTAATCTATATTCCACATTTCTCCCAATGTCCATTCCATGCATGATCCAGGACACACTCATTCACCCTCCCCATAGACAGCTCCAAACCACTACCAAGTCACCTAACTATGAATGGTTACAGGACATAAATCTCACTCTCA
 CATHHTAHVTDCACGGACWTAAACCTATWYYCCACATDTCTMMSAATGTACATACYATDCHTRATCTWGGACAGWMYMWWTYACCCTCCCHATDGACWRGCCCAAATCAHTDCHAADAHACCCAACYAWSAABCGTAACACCACATAAAKMTCACKMKCA

 * 340 * 360 * 380 * 400 * 420 * 440 * 460 * 480
R-E_caballus : ----------------------------------------------------------------------------------------------------------------------------------------------------------------
A-E_caballus : ----------------------------------------------------------------------------------------------------------------------------------------------------------------
R-C_bactrianus : ----------------------------------------------------------------------------------------------------------------------------------------------------------------
A-C_bactrianus : ----------------------------------------------------------------------------------------------------------------------------------------------------------------
R-C_lupus_familiaris : ----------------------------------------------------------------------------------------------------------------------------------------------------------------
A-C_lupus_familiaris : ----------------------------------------------------------------------------------------------------------------------------------------------------------------
R-N_procyonoides : ----------------------------------------------------------------------------------------------------------------------------------------------------------------
A-N_procyonoides : ----------------------------------------------------------------------------------------------------------------------------------------------------------------
R-V_lagopus : ----------------------------------------------------------------------------------------------------------------------------------------------------------------
A-V_lagopus : ----------------------------------------------------------------------------------------------------------------------------------------------------------------
R-M_putorius : ----------------------------------------------------------------------------------------------------------------------------------------------------------------
A-M_putorius : ----------------------------------------------------------------------------------------------------------------------------------------------------------------
R-B_taurus : ----------------------------------------------------------------------------------------------------------------------------------------------------------------
A-B_taurus : ----------------------------------------------------------------------------------------------------------------------------------------------------------------
R-O_aries : ----------------------------------------------------------------------------------------------------------------------------------------------------------------
A-O_aries : ----------------------------------------------------------------------------------------------------------------------------------------------------------------
R-S_scrofa : TAATTACCATGCCGCGTGAAACCAGCAACCCGCTTGGCAGGGATCCCTCTTCTCGCTCCGGGCCCATAAATCGTGGGGGTTTCTACTGATGAACTTTAACAGGCATCTGGTTCTTACTTCAGGACCATCTCATCTAAAATCGCCCACTCTTTCCCCTTAA
A-S_scrofa : TAATTACCATGCCGCGTGAAACCAGCAACCCGCTTGGCAGGGATCCCTCTTCTCGCTCCGGGCCCATAAATCGTGGGGGTTTCTACTGATGAACTTTAACAGGCATCTGGTTCTTACTTCAGGACCATCTCATCTAAAATCGCCCACTCTTTCCCCTTAA
R-O_cuniculus : ----------------------------------------------------------------------------------------------------------------------------------------------------------------
A-O_cuniculus : ----------------------------------------------------------------------------------------------------------------------------------------------------------------
R-M_musculus : ----------------------------------------------------------------------------------------------------------------------------------------------------------------
A-M_musculus : ----------------------------------------------------------------------------------------------------------------------------------------------------------------
R-R_norvegicus : ----------------------------------------------------------------------------------------------------------------------------------------------------------------
A-R_norvegicus : ----------------------------------------------------------------------------------------------------------------------------------------------------------------
R-M_coypus : ----------------------------------------------------------------------------------------------------------------------------------------------------------------
A-M_coypus : ----------------------------------------------------------------------------------------------------------------------------------------------------------------
R-A_platyrhynchos : TGTCCCCAACCAGAACAAGGCCCCATAATGATGAATGCTTGACAGACATACCCTACCAACACTCCAAATTCCTCTCCACCCACCCATTACTCATGAAGCTGCGTACCAGATGGATTTATTAATCGTACACCTCACGTGAAATCAGCAATCCTTGCACATA
A-A_platyrhynchos : TGTCCCCAACCAGAACAAGGCCCCATAATGATGAATGCTTGACAGACATACCCTACCAACACTCCAAATTCCTCTCCACCCACCCATTACTCATGAAGCTGCGTACCAGATGGATTTATTAATCGTACACCTCACGTGAAATCAGCAATCCTTGCACATA
R-G_gallus : TGTTCTCC-CCCCAACAAGTCACC-TAACTATGAATGGTTACAGGACATACATT--TAAC-TACCATGTTCTAACCCATTTGGTTAT-GCTCGC-------CGTATCAGATGGATTTATTGATCGTCCACCTCACGAGAGATCAGCAACCCCTGCCTGTA
A-G_gallus : TGTTCTTC-CCCCAACAAGTCACC-TAACTATGAATGGTTACAGGACATACATT--TAAC-TACCATGTTCTAACCCATTTGGTTAT-GCTCGC-------CGTATCAGATGGATTTATTGATCGTCCACCTCACGAGAGATCAGCAACCCCTGCTTGTA
 TGTTCHCCACCCCAACAAGDCCCCRTAACBATGAATGCTTGVADGACATACCTTRCTAACRBHCCATATTCCDHBCCABTTDCTHATKACTCACKWWRMYRCGTATCAGATGGATTTATTAATCGTACACCTCACGAGADATCAGCAACCCTTGCCTDTA

 * 500 * 520 * 540 * 560 * 580 * 600 * 620 * 640
R-E_caballus : ----------------------------------------------------------------------------------------------------------------------------------------------------------------
A-E_caballus : ----------------------------------------------------------------------------------------------------------------------------------------------------------------
R-C_bactrianus : ----------------------------------------------------------------------------------------------------------------------------------------------------------------
A-C_bactrianus : ----------------------------------------------------------------------------------------------------------------------------------------------------------------
R-C_lupus_familiaris : ----------------------------------------------------------------------------------------------------------------------------------------------------------------
A-C_lupus_familiaris : ----------------------------------------------------------------------------------------------------------------------------------------------------------------
R-N_procyonoides : ----------------------------------------------------------------------------------------------------------------------------------------------------------------
A-N_procyonoides : ----------------------------------------------------------------------------------------------------------------------------------------------------------------
R-V_lagopus : ----------------------------------------------------------------------------------------------------------------------------------------------------------------
A-V_lagopus : ----------------------------------------------------------------------------------------------------------------------------------------------------------------
R-M_putorius : ----------------------------------------------------------------------------------------------------------------------------------------------------------------
A-M_putorius : ----------------------------------------------------------------------------------------------------------------------------------------------------------------
R-B_taurus : ----------------------------------------------------------------------------------------------------------------------------------------------------------------
A-B_taurus : ----------------------------------------------------------------------------------------------------------------------------------------------------------------
R-O_aries : ----------------------------------------------------------------------------------------------------------------------------------------------------------------
A-O_aries : ----------------------------------------------------------------------------------------------------------------------------------------------------------------
R-S_scrofa : ATAAGACATCTCGATGGACTAATGACTAATCAGCCCATGCTCACACATAACTGAGGTTTCATACATTTGGTATTTTTTAATTTTTGGGGATGCTTGGACTCAGCCATGGCCGTCAAAGGCCCTAACACAGTCAAATCAATTGTAGCTGGACTTCATGGAA
A-S_scrofa : ATAAGACATCTCGATGGACTAATGACTAATCAGCCCATGCTCACACATAACTGAGGTTTCATACATTTGGTATTTTTTAATTTTTGGGGATGCTTGGACTCAGCCATGGCCGTCAAAGGCCCTAACACAGTCAAATCAATTGTAGCTGGACTTCATGGAA
R-O_cuniculus : ----------------------------------------------------------------------------------------------------------------------------------------------------------------
A-O_cuniculus : ----------------------------------------------------------------------------------------------------------------------------------------------------------------
R-M_musculus : ----------------------------------------------------------------------------------------------------------------------------------------------------------------
A-M_musculus : ----------------------------------------------------------------------------------------------------------------------------------------------------------------
R-R_norvegicus : ----------------------------------------------------------------------------------------------------------------------------------------------------------------
A-R_norvegicus : ----------------------------------------------------------------------------------------------------------------------------------------------------------------
R-M_coypus : ----------------------------------------------------------------------------------------------------------------------------------------------------------------
A-M_coypus : ----------------------------------------------------------------------------------------------------------------------------------------------------------------
R-A_platyrhynchos : ATGTCCGACGTGACTAGCTTCAGGCCCATACGTTCCCCCTAAACCCCTCGCCCTCCTCACATTTT--TGCGCCTCTGGTTCCTCGGTCAGGGCCATCAATTGGGT--TCACTCACCTCTCCTTGCCCTTCAAAGTGGCATCTGTGGAATACTTCCACCAT
A-A_platyrhynchos : ATGTCCGACGTGACTAGCTTCAGGCCCATACGTTCCCCCTAAACCCCTCGCCCTCCTCACATTTT--TGCGCCTCTGGTTCCTCGGTCAGGGCCATCAATTGGGT--TCACTCACCTCTCCTTGCCCTTCAAAGTGGCATCTGTGGAATACTTCCACCAT
R-G_gallus : ATGTACTTCATGACCAGTCTCAGGCCCATTCTTTCCCCCTACACCCCTCGCCCTACTTGCCTTCCACCGTACCTCTGGTTCCTCGGTCAGGCACATCCCATGCATAACTCCTGAACTTTC-TCACTTTTCACGAAGTCATCTGTGGATTATCTTCCCCTC
A-G_gallus : ATGTACTTCATGACCAGTCTCAGGCCCATTCTTTCCCCCTACACCCCTCGCCCTACTTGCCTTCCACCGTACCTCTGGTTCCTCGGTCAGGCACATCCCATGCATAACTCCTGAACTTTC-TCACTTTTCACGAAGTCATCTGTGGATTATCTTCCCCTC
 ATGTVCBACVTGACTAGHCTCAGGCCCATTCDTTCCCCCTACACCCCTCGCCCTVCTTDCATTCHWYTGBDCCTCTGGTTCCTCGGTCAGGGCCATCACTTGGVTAWBBCCTBAACTBTCCTTACCHTTCACAAAGBCATCTGTGGADTACTTCCHCCAH

 * 660 * 680 * 700 * 720 * 740 * 760 * 780 * 800
R-E_caballus : ----------------------------------------------------------------------------------------------------------------------------------------------------------------
A-E_caballus : ----------------------------------------------------------------------------------------------------------------------------------------------------------------
R-C_bactrianus : ----------------------------------------------------------------------------------------------------------------------------------------------------------------
A-C_bactrianus : ----------------------------------------------------------------------------------------------------------------------------------------------------------------
R-C_lupus_familiaris : ----------------------------------------------------------------------------------------------------------------------------------------------------------------
A-C_lupus_familiaris : ----------------------------------------------------------------------------------------------------------------------------------------------------------------
R-N_procyonoides : ----------------------------------------------------------------------------------------------------------------------------------------------------------------
A-N_procyonoides : ----------------------------------------------------------------------------------------------------------------------------------------------------------------
R-V_lagopus : ----------------------------------------------------------------------------------------------------------------------------------------------------------------
A-V_lagopus : ----------------------------------------------------------------------------------------------------------------------------------------------------------------
R-M_putorius : ----------------------------------------------------------------------------------------------------------------------------------------------------------------
A-M_putorius : ----------------------------------------------------------------------------------------------------------------------------------------------------------------
R-B_taurus : ----------------------------------------------------------------------------------------------------------------------------------------------------------------
A-B_taurus : ----------------------------------------------------------------------------------------------------------------------------------------------------------------
R-O_aries : ----------------------------------------------------------------------------------------------------------------------------------------------------------------
A-O_aries : ----------------------------------------------------------------------------------------------------------------------------------------------------------------
R-S_scrofa : CTCATGATCCGGCACGACAATCCAAACAAGGTGCTATTCAGTCAATGGTTACGGGACATAACGTGCGTACACGTGCGTACACGTGCGTACACGTGCGTACACGTGCGTACACGTGCGTACACGTGCGTACACGTGCGTACACGTGCGTACACGTGCGTAC
A-S_scrofa : CTCATGATCCGGCACGACAATCCAAACAAGGTGCTATTCAGTCAATGGTTACGGGACATAACGTGCGTACACGTGCGTACACGTGCGTACACGTGCGTACACGTGCGTACACGTGCGTACACGTGCGTACACGTGCGTACACGTGCGTACACGTGCGTAC
R-O_cuniculus : ----------------------------------------------------------------------------------------------------------------------------------------------------------------
A-O_cuniculus : ----------------------------------------------------------------------------------------------------------------------------------------------------------------
R-M_musculus : ----------------------------------------------------------------------------------------------------------------------------------------------------------------
A-M_musculus : ----------------------------------------------------------------------------------------------------------------------------------------------------------------
R-R_norvegicus : ----------------------------------------------------------------------------------------------------------------------------------------------------------------
A-R_norvegicus : ----------------------------------------------------------------------------------------------------------------------------------------------------------------
R-M_coypus : ----------------------------------------------------------------------------------------------------------------------------------------------------------------
A-M_coypus : ----------------------------------------------------------------------------------------------------------------------------------------------------------------
R-A_platyrhynchos : CTCAATGCGTAATCGCGGCATCCTCCAGCTTTTTGGCGCCTCTGGTTCCTTTTATTTTTTCCGGGGTTACCTCACAGCTGGCCCTTCCCAGTGACTTCG-GGGGTCCCA--CAATCTAAGCCTGGAC-ACACCTGCGTTATCGCGC-TATCCTATATCTC
A-A_platyrhynchos : CTCAATGCGTAATCGCGGCATCCTCCAGCTTTTTGGCGCCTCTGGTTCCTTTTATTTTTTCCGGGGTTACCTCACAGCTGGCCCTTCCCAGTGACTTCG-GGGGTCCCA--CAATCTAAGCCTGGAC-ACACCTGCGTTATCGCGC-TATCCTATATCTC
R-G_gallus : TTTAGTCCGTGATCGCGGCATCTTCTCTCTTCT-ATTGCTGTTGGTTCCTTCTCTTTTT---GGGGCTTCTTCACAGGTTACCCTTCACAGTGCGGGTGCG-AGTGCTATTCAAGTGAAGCCTGGACTACACCTGCGTTG-CGTCC-TATCCTAGTCCTC
A-G_gallus : TTTAGTCCGTGATCGCGGCATCTTCTCTCTTCT-ATTGCTGTTGGTTCCTTCTCTTTTT---GGGGCTTCTTCACAGGTTGCCCTTCACAGTGCGGGTGCGGAGTGCTATTCAAGTGAAGCCTGGACTACACCTGCGTTG-CGTCC-TATCCTAGTCCTC
 CTCADTVCGTGATCGCGGCATCCTCHCDCTTTTYDDTGCHGTTGGTTCCTTCTVTTTTTWMCGGGGBTACHTCACAGBTBRCCCTTCHCAGTGHGBGTGCGGGGTCCTAYWCAAGCGAAGCCTGGACTACACCTGCGTTVWCGTGCGTATCCTAGHBCTC

 * 820 * 840 * 860 * 880 * 900 * 920 * 940 * 960
R-E_caballus : ----------------------------------------------------------------------------------------------------------------------------------------------------------------
A-E_caballus : ----------------------------------------------------------------------------------------------------------------------------------------------------------------
R-C_bactrianus : ----------------------------------------------------------------------------------------------------------------------------------------------------------------
A-C_bactrianus : ----------------------------------------------------------------------------------------------------------------------------------------------------------------
R-C_lupus_familiaris : ----------------------------------------------------------------------------------------------------------------------------------------------------------------
A-C_lupus_familiaris : ----------------------------------------------------------------------------------------------------------------------------------------------------------------
R-N_procyonoides : ----------------------------------------------------------------------------------------------------------------------------------------------------------------
A-N_procyonoides : ----------------------------------------------------------------------------------------------------------------------------------------------------------------
R-V_lagopus : ----------------------------------------------------------------------------------------------------------------------------------------------------------------
A-V_lagopus : ----------------------------------------------------------------------------------------------------------------------------------------------------------------
R-M_putorius : ----------------------------------------------------------------------------------------------------------------------------------------------------------------
A-M_putorius : ----------------------------------------------------------------------------------------------------------------------------------------------------------------
R-B_taurus : -----------------------------------------------------------------------------ACTAATGGCTAATCAGCCCATGCTCACACATAACTGTGCTGTCATACATTTGGTATTTTTTTATTTTGGGGGATGCTTGGACT
A-B_taurus : -----------------------------------------------------------------------------ACTAATGGCTAATCAGCCCATGCTCACACATAACTGTGCTGTCATACATTTGGTATTTTTTTATTTTGGGGGATGCTTGGACT
R-O_aries : ----------------------------------------------------------------------------------------------------------------------------------------------------------------
A-O_aries : ----------------------------------------------------------------------------------------------------------------------------------------------------------------
R-S_scrofa : ACGTGCGTACACGTGCGTACACGTGCGTACACGTGCGTACACGTGCGTACACGTGCGTACACGTGCGTACACGTGCGTACACGTGCGTACACGTGCGTACACGTGCGTACACGTGCGTACACGCGCATATAAGCAGGTAAATTATTAGCTCATTCAAACC
A-S_scrofa : ACGTGCGTACACGTGCGTACACGTGCGTACACGTGCGTACACGTGCGTACACGTGCGTACACGTGCGTACACGTGCGTACACGTGCGTACACGTGCGTACACGTGCGTACACGTGCGTACACGCGCATATAAGCAGGTAAATTATTAGCTCATTCAAACC
R-O_cuniculus : ----------------------------------------------------------------------------------------------------------------------------------------------------------------
A-O_cuniculus : ----------------------------------------------------------------------------------------------------------------------------------------------------------------
R-M_musculus : ----------------------------------------------------------------------------------------------------------------------------------------------------------------
A-M_musculus : ----------------------------------------------------------------------------------------------------------------------------------------------------------------
R-R_norvegicus : ----------------------------------------------------------------------------------------------------------------------------------------------------------------
A-R_norvegicus : ----------------------------------------------------------------------------------------------------------------------------------------------------------------
R-M_coypus : ----------------------------------------------------------------------------------------------------------------------------------------------------------------
A-M_coypus : ----------------------------------------------------------------------------------------------------------------------------------------------------------------
R-A_platyrhynchos : AGGGATTACTCAATGAGACGGTTGGCGTATATGGGGAATCACCTTGACACTGATGCACTTTG-ACCACATTCAGTTAATGCTCTCTCCACAGCTCTATATAATAGGGCTATTTAGTGAATGCTCGATGGACATACTTT----------------------
A-A_platyrhynchos : AGGGATTACTCAATGAGACGGTTGGCGTATATGGGGAATCACCTTGACACTGATGCACTTTG-ACCACATTCAGTTAATGCTCTCTCCACAGCTCTATATAATAGGGCTATTTAGTGAATGCTCGATGGACATACTTT----------------------
R-G_gallus : TCGTGTCCCTCGATGAGACGGTTTGCGTGTATGGGGAATCATCTTGACACTGATGCACTTTGGATCGCATTTGGTTA-TGGTTCTTCCACCCCCCCGGTAAATGGTGCTATTTAGTGAATGCTTGTCGGACATATTTTTATCAATTTTCACTTCCTCTAT
A-G_gallus : TCGTGTCCCTCGATGAGACGGTTTGCGTGTATGGGGAATCATCTTGACACTGATGCACTTTGGATCGCATTTGGTTA-TGGTTCTTCCACCCCCCCGGTAAATGGTGCTATTTAGTGAATGCTTGTCGGACATATTTTTATCAATTTTCACTTCCTCTAT
 ACGTGTBHCTCVATGAGACGGTTTGCGTATATGGGGAATCACCTTGACACTGATGCACTTTGGABCGCATTCGGTTAATGATTTGYCCACMCSYCCRTACAMTNGCGYWATTKWGYGAAYRCTCGTTKGACATATTTTTATTTATTDGCACDTTCDVACT

 * 980 * 1000 * 1020 * 1040 * 1060 * 1080 * 1100 * 1120
R-E_caballus : ----------------------------------------------------------------------------------------------------------------------------------------------------------------
A-E_caballus : ----------------------------------------------------------------------------------------------------------------------------------------------------------------
R-C_bactrianus : ----------------------------------------------------------------------------------------------------------------------------------------------------------------
A-C_bactrianus : ----------------------------------------------------------------------------------------------------------------------------------------------------------------
R-C_lupus_familiaris : ----------------------------------------------------------------------------------------------------------------------------------------------------------------
A-C_lupus_familiaris : ----------------------------------------------------------------------------------------------------------------------------------------------------------------
R-N_procyonoides : ----------------------------------------------------------------------------------------------------------------------------------------------------------------
A-N_procyonoides : ----------------------------------------------------------------------------------------------------------------------------------------------------------------
R-V_lagopus : ----------------------------------------------------------------------------------------------------------------------------------------------------------------
A-V_lagopus : ----------------------------------------------------------------------------------------------------------------------------------------------------------------
R-M_putorius : ----------------------------------------------------------------------------------------------------------------------------------------------------------------
A-M_putorius : ----------------------------------------------------------------------------------------------------------------------------------------------------------------
R-B_taurus : CAGCTATGGCCGTCAAAGGCCCTGACCCGGAGCATCTATTGTAGCTGGACTTAACTGCATCTTGAGCACC-AGCATA-----ATGATAAGCATGGACATTACAGTCAATGGTCACAGGACATAAATTATATTATAT-ATCCCCCCTTCATAAAAATTTCC
A-B_taurus : CAGCTATGGCCGTCAAAGGCCCTGACCCGGAGCATCTATTGTAGCTGGACTTAACTGCATCTTGAGCACC-AGCATA-----ATGATAAGCGTGGACATTACAGTCAATGGTCACAGGACATAAATTATATTATAT-ATCCCCCCTTCATAAAAATTTCC
R-O_aries : ----------------------------------------------------------------------------------------------------------------------------------------------------------------
A-O_aries : ----------------------------------------------------------------------------------------------------------------------------------------------------------------
R-S_scrofa : CCCCTTACCCCCCATTAAACTTATGCTCTACACACCCTATAACGCCTTGCCAAACCCCAAAAACAAAGCAGAGTGTACAAATACAATAAGCCTAACTTACACTAAACAACATTTAACAACACAAACCACCATATCTTATAAAACACTTATAAAA--CACT
A-S_scrofa : CCCCTTACCCCCCATTAAACTTATGCTCTACACACCCTATAACGCCTTGCCAAACCCCAAAAACAAAGCAGAGTGTACAAATACAATAAGCCTAACTTACACTAAACAACATTTAACAACACAAACCACCATATCTTATAAAACACTTATAAAA--CACT
R-O_cuniculus : ----------------------------------------------------------------------------------------------------------------------------------------------------------------
A-O_cuniculus : ----------------------------------------------------------------------------------------------------------------------------------------------------------------
R-M_musculus : ----------------------------------------------------------------------------------------------------------------------------------------------------------------
A-M_musculus : ----------------------------------------------------------------------------------------------------------------------------------------------------------------
R-R_norvegicus : ----------------------------------------------------------------------------------------------------------------------------------------------------------------
A-R_norvegicus : ----------------------------------------------------------------------------------------------------------------------------------------------------------------
R-M_coypus : ----------------------------------------------------------------------------------------------------------------------------------------------------------------
A-M_coypus : ----------------------------------------------------------------------------------------------------------------------------------------------------------------
R-A_platyrhynchos : ----------------------------------------------------------------------AAAAACA---------AAAC-------CACCCCAACCAC-------AACCCCACAATATATATATACATACAAAACGAAATGCATA-ACA
A-A_platyrhynchos : ----------------------------------------------------------------------AAAAACA---------AAAC-------CACCCCAACCAC-------AACCCCACAATATATATATACATACAAAACGAAATGCATA-ACA
R-G_gallus : TTTCTTCACAAAACTAGGAAATTCACCACAATTTTTTCTTTGTTATTTTTTAATTTTTTTTTTATTTTTTAAAAACATTTTTTAAAAAACTAAATTACATACAAACTACCGCATAAAATCCCTCAAACTATACAAACGTTTATCGTATAATATATATACA
A-G_gallus : TTTCTTCACAAAACTAGGAAATTCACCACAATTTTTTCTTTGTTATTTTTTAATTTTTTTTTTATTTTTTAAAAACATTTTTTAAAAAACTAAATTACATACAAACTACCGCATAAAATCCCTCAAACTATACAAACGTTTATCGTATAATATATATACA
 CHBCTTHVCCCVHCTAAGACHTTBACCCBAADCATCTHTTDDHGCTTTDCTAAACTBCATHTTVADHDCHAARAAYAYWWWTAHAAWAASCATADHHCAYACAAACCACCGTHTAAAAACMCAMAATATATWAWAWCATACAACAYTTAWWAAATATACA

 * 1140 * 1160 * 1180 * 1200 * 1220 * 1240 * 1260 * 1280
R-E_caballus : -----------------------------------------------------------------------------------------------------------------------------------------------------GTTAATGTAGC
A-E_caballus : -----------------------------------------------------------------------------------------------------------------------------------------------------GTTAATGTAGC
R-C_bactrianus : -----------------------------------------------------------------------------------------------------------------------------------------------------GTTAATGTAGC
A-C_bactrianus : -----------------------------------------------------------------------------------------------------------------------------------------------------GTTAATGTAGC
R-C_lupus_familiaris : -----------------------------------------------------------------------------------------------------------------------------------------------------GTTAATGTAGC
A-C_lupus_familiaris : -----------------------------------------------------------------------------------------------------------------------------------------------------GTTAATGTAGC
R-N_procyonoides : -----------------------------------------------------------------------------------------------------------------------------------------------------GTTAATGTAGC
A-N_procyonoides : -----------------------------------------------------------------------------------------------------------------------------------------------------GTTAATGTAGC
R-V_lagopus : -----------------------------------------------------------------------------------------------------------------------------------------------------GTTAATGTAGC
A-V_lagopus : -----------------------------------------------------------------------------------------------------------------------------------------------------GTTAATGTAGC
R-M_putorius : -----------------------------------------------------------------------------------------------------------------------------------------------------GTTAATGTAGC
A-M_putorius : -----------------------------------------------------------------------------------------------------------------------------------------------------GTTAATGTAGC
R-B_taurus : CCCTTAAATATCTACCACCACTTTTAACAGACTTTTCCCTAGATACTTATTTAAATTTTTCACGC-----TTTCAATACTCAATTTAGCA----CTCCAAACAAAGTCAA-TATATAAACGCAGGCCCCCCCCCCC-----------CG-TTGATGTAGC
A-B_taurus : CCCTTAAATATCTACCACCACTTTTAACAGACTTTTCCCTAGATACTTATTTAAATTTTTCACGC-----TTTCAATACTCAATTTAGCA----CTCCAAACAAAGTCAA-TATATAAACGCAGGCCCCCCCCCCC-----------CGGTTGATGTAGC
R-O_aries : -----------------------------------------------------------------------------------------------------------------------------------------------------GTTAATGTAGC
A-O_aries : -----------------------------------------------------------------------------------------------------------------------------------------------------GTTAATGTAGC
R-S_scrofa : TACTTAAATACGTGCTACGAAAGCAGGCACCTACCCCCCTAGATTTTTACGCCAATCTACCACAAATAAGTTTAAAATTACAACACAATAACCTCCCAAAATATAAGCACCTATTTAAGCATACGCCCACAATCTGAATATAGCTTATAGTTAATGTAGC
A-S_scrofa : TACTTAAATACGTGCTACGAAAGCAGGCACCTACCCCCCTAGATTTTTACGCCAATCTACCACAAATAAGTTTAAAATTACAACACAATAACCTCCCAAAATATAAGCACCTATTTAAGCATACGCCCACAATCTGAATATAGCTTATAGTTAATGTAGC
R-O_cuniculus : -----------------------------------------------------------------------------------------------------------------------------------------------------GTTAATGTAGC
A-O_cuniculus : -----------------------------------------------------------------------------------------------------------------------------------------------------GTTAATGTAGC
R-M_musculus : -----------------------------------------------------------------------------------------------------------------------------------------------------GTTAATGTAGC
A-M_musculus : -----------------------------------------------------------------------------------------------------------------------------------------------------GTTAATGTAGC
R-R_norvegicus : -----------------------------------------------------------------------------------------------------------------------------------------------------GTTAATGTAGC
A-R_norvegicus : -----------------------------------------------------------------------------------------------------------------------------------------------------GTTAATGTAGC
R-M_coypus : -----------------------------------------------------------------------------------------------------------------------------------------------------GTTAATGTAGC
A-M_coypus : -----------------------------------------------------------------------------------------------------------------------------------------------------GTTAATGTAGC
R-A_platyrhynchos : TGAC--------CTAAATTTATTAGAGAAACTCCAGCACTAAAGACG--ATCCAAACCCGA--TGACA-ATCATTACTTTGACCTAACAAACA----TTACCCA----ATTAACCAGCCA--------CCTGCCCC-------------GTCCACATAGC
A-A_platyrhynchos : TGAC--------CTAAATTTATTAGAGAAACTCCAGCACTAAAGACG--ATCCAAACCCGA--TGACA-ATCATTACTTTGACCTAACAAACA----TTACCCA----ATTAACCAGCCA--------CCTGCCCC-------------GTCCACATAGC
R-G_gallus : TTATTGTTTATTCTATCATTATTAGAGAAACTCCACTACCAAAACCATCATTAAAACAAAAATTTACATGCCACTTAACTCCCCTCACAAACAATCGTTATTTATATTGTTAATTAGCAAACACAAAACCCGCCTTCTACCACTATAAAGCCCCCATAGC
A-G_gallus : TTATTGTTTATTCTATCATTATTAGAGAAACTCCACTACCAAAACCATCATTAAAACAAAAATTTACATGCCACTTAACTCCCCTCACAAACAATCGTTATTTATATTGTTAATTAGCAAACACAAAACCTGCCTTCTACCACTATAAAGCCCCCATAGC
 TNMTTAAATATBYTMTACTWATTAGAGMAACTCCACCMCTARATACTTAATYMAAWCTANMACTNACAWGTYWCWAAWYTCAMCTCACAAACAWCCCTWAAYAAAATCATTWATTWRMAMACACGCCCCCYGCCYCMWWMYASYWTAHAGTTAATGTAGC

 * 1300 * 1320 * 1340 * 1360 * 1380 * 1400 * 1420 * 1440
R-E_caballus : TTAATAATATAAAGCAAGGCACTGAAAATGCCTAGATGAGTAT-TCTTAC-TCCATAAACACATAGGCTTGGTCCTAGCCTTTTTATTAGTTATTAATAGAATTACACATGCAAGTATCCGCACCCCAGTGAG-AATGCCCTCTAAATC-ACGTCTCTAC
A-E_caballus : TTAATAATATAAAGCAAGGCACTGAAAATGCCTAGATGAGTAT-TCTTAC-TCCATAAACACATAGGCTTGGTCCTAGCCTTTTTATTAGTTATTAATAGAATTACACATGCAAGTATCCGCACCCCAGTGAG-AATGCCCTCTAAATC-GCGTCTCTAC
R-C_bactrianus : TTAACTT--CAAAGCAAGGCGCTGAAAATGCCTGGATGGG-CA-CCTGGC-CCCATGAACACACAGGTTTGATCCCAGCCTTTCTATTAGTTTTTAATAAAATTATACATGCAAGTATCCGCATCCCAGTGAG-AGTGCCCCCTAGTTCCAAG---TA--
A-C_bactrianus : TTAACTT--CAAAGCAAGGCGCTGAAAATGCCTGGATGGG-CA-CCTGGC-CCCATGAACACACAGGTTTGATCCCAGCCTTTCTATTAGTTTTTAATAAAATTATACATGCAAGTATCCGCATCCCAGTGAG-AGTGCCCCCTAGTTCCAAG---TA--
R-C_lupus_familiaris : TTAATTA-ATAAAGCAAGGCACTGAAAATGCCAAGATGAGTCG-CACGAC-TCCATAAACATAAAGGTTTGGTCCTAGCCTTCCTATTAGTTTTTAGTAGACTTACACATGCAAGCCTCCACGCCCCAGTGAG-AATGCCCTTAAAATC-AC----CAGT
A-C_lupus_familiaris : TTAATTA-ATAAAGCAAGGCACTGAAAATGCCAAGATGAGTCG-CACGAC-TCCATAAACATAAAGGTTTGGTCCTAGCCTTCCTATTAGTTTTTAGTAGACTTACACATGCAAGCCTCCACGCCCCAGTGAG-AATGCCCTCAAAATC-AC----CAGT
R-N_procyonoides : TTAATTA-ATAAAGCAAGGCACTGAAAATGCCAAGATGAGTCA-CATGAC-TCCATAAACACAAAGGTTTGGTCCTAGCCTTCCTATTAGTCCTTAGTAGACTTACACATGCAAGCTTCCACGCCCCAGTGAG-AATGCCCTTAAAATC-AC----CAAC
A-N_procyonoides : TTAATTA-ATAAAGCAAGGCACTGAAAATGCCAAGATGAGTCA-CATGAC-TCCATAAACACAAAGGTTTGGTCCTAGCCTTCCTATTAGTCCTTAGTAGACTTACACATGCAAGCTTCCACGCCCCAGTGAG-AATGCCCTTAAAATC-AC----CAAC
R-V_lagopus : TTAATTA-ATAAAGCAAGGCACTGAAAATGCCAAGATGAGTCA-TGAGAC-TCCATAAACACAAAGGTTTGGTCCTGGCCTTCCTATTAGTCCTTAGTAAACTTACACATGCAAGCCTCCACGCCCCAGTGAG-AATGCCCTTAAAATC-AT----TAAC
A-V_lagopus : TTAATTA-ATAAAGCAAGGCACTGAAAATGCCAAGATGAGTCA-TGAGAC-TCCATAAACACAAAGGTTTGGTCCTGGCCTTCCTATTAGTCCTTAGTAAACTTACACATGCAAGCCTCCACGCCCCAGTGAG-AATGCCCTTAAAATC-AT----TAAC
R-M_putorius : TTATTAA-ATAAAGCAAGGCACTGAAAATGCCTAGAAGAGTCA-CAAGAC-TCCATAAACACAAAGGTTTGGTCCTAGCCTTCCTATTGATTATTAACAGAATTACACATGCAAGTCTCTACACCCCAGTGAG-AATGCCCTCCAAATCTATA---TGTT
A-M_putorius : TTATTAA-ATAAAGCAAGGCACTGAAAATGCCTAGAAGAGTCA-CAAGAC-TCCATAAACACAAAGGTTTGGTCCTAGCCTTCCTATTGATTATTAACAGAATTACACATGCAAGTCTCTACACCCCAGTGAG-AATGCCCTCCAAATCTATA---TGTT
R-B_taurus : TTAA-CC--CAAAGCAAGGCACTGAAAATGCCTAGATGAGTCT-CCCAAC-TCCATAAACACATAGGTTTGGTCCCAGCCTTCCTGTTAACTCTTAATAAACTTACACATGCAAGCATCTACACCCCAGTGAG-AATGCCCTCTAGGTT-AT-----TAA
A-B_taurus : TTAA-CC--CAAAGCAAGGCACTGAAAATGCCTAGATGAGTCT-CCCAAC-TCCATAAACACATAGGTTTGGTCCCAGCCTTCCTGTTAACTCTTAATAAACTTACACATGCAAGCATCTACACCCCAGTGAG-AATGCCCTCTAGGTT-AT-----TAA
R-O_aries : TTAAACT--TAAAGCAAGGCACTGAAAATGCCTAGATGAGTCT-ACTGAC-TCCATGAACATATAGGTTTGGTCCCAGCCTTCCTGTTAACTTTCAATAGACTTATACATGCAAGCATCCACGCCCCGGTGAGTAACGCCCTTCGAATC-ACA----CAG
A-O_aries : TTAAACT--TAAAGCAAGGCACTGAAAATGCCTAGATGAGTCT-ACTGAC-TCCATGAACATATAGGTTTGGTCCCAGCCTTCCTGTTAACTTTCAATAGACTTATACATGCAAGCATCCACGCCCCGGTGAGTAACGCCCTTCGAATC-ACA----CAG
R-S_scrofa : TTAAATTATCAAAGCAAGGCACTGAAAATGCCTAGATGGGCCT-CACAGC-CCCATAAACACACAGGTTTGGTCCTGGCCTTTCTATTAATTCTTAATAAAATTACACATGCAAGTATCCGCGCCCCGGTGAG-AATGCCCTCCAGATC-CT-----AAA
A-S_scrofa : TTAAATTATCAAAGCAAGGCACTGAAAATGCCTAGATGGGCCT-CACAGC-CCCATAAACACACAGGTTTGGTCCTGGCCTTTCTATTAATTCTTAATAAAATTACACATGCAAGTATCCGCGCCCCGGTGAG-AATGCCCTCCAGATC-CT-----AAA
R-O_cuniculus : TTAACAA--CAAAGCAAAGCACTGAAAATGCTTAGATGAGCCTTCCCGGC-TCCATAAACATAAAGGTTTGGTCCTGGCCTTTTTATTGTTTTGTAGCAACCTTACACATGCAAGACTCCTCACGCCAGTGAG-AATGCCCTTAACATCAAA-----CTA
A-O_cuniculus : TTAACAA--CAAAGCAAAGCACTGAAAATGCTTAGATGAGCCTTCCCGGC-TCCATAAACATAAAGGTTTGGTCCTGGCCTTTTTATTGTTTTGTAGCAACCTTACACATGCAAGACTCCTCACGCCAGTGAG-AATGCCCTTAACATCAAA-----CTA
R-M_musculus : TTAATAA--CAAAGCAAAGCACTGAAAATGCTTAGATGGATAA-TTGTAT-CCCATAAACACAAAGGTTTGGTCCTGGCCTTATAATTAATTAGAGGTAAAATTACACATGCAAACCTCCATAGACCGGTGTAAAATCCCTTAAACATTTACT----TAA
A-M_musculus : TTAATAA--CAAAGCAAAGCACTGAAAATGCTTAGATGGATAA-TTGTAT-CCCATAAACACAAAGGTTTGGTCCTGGCCTTATAATTAATTAGAGGTAAAATTACACATGCAAACCTCCATAGACCGGTGTAAAATCCCTTAAACATTTACT----TAA
R-R_norvegicus : TTA-TAA--TAAAGCAAAGCACTGAAAATGCTTAGATGGATTC-AAAAAT-CCCATAAACACAAAGGTTTGGTCCTGGCCTTATAATTAATTGGAGGTAAGATTACACATGCAAACATCCATAAACCGGTGTAAAATCCCTTAAACATTTGCC----TAA
A-R_norvegicus : TTA-TAA--TAAAGCAAAGCACTGAAAATGCTTAGATGGATTC-AAAAAT-CCCATAAACACAAAGGTTTGGTCCTGGCCTTATAATTAATTGGAGGTAAGATTACACATGCAAACATCCATAAACCGGTGTAAAATCCCTTAAACATTTGCC----TAA
R-M_coypus : TTA-TCA--TAAAGCAAGGCACTGAAAATGCCTAGATGAGTAG-TATTAC-TCCATAAACACAAAGGTTTGGTCCTGGCTTTTTTATTAATTATTAGCAGAATTATACATGCAAGAGTCATCATTCCTGTG-AGAATGCCCTACAAATCAAC------AA
A-M_coypus : TTA-TCA--TAAAGCAAGGCACTGAAAATGCCTAGATGAGTAG-TATTAC-TCCATAAACACAAAGGTTTGGTCCTGGCTTTTTTATTAATTATTAGCAGAATTATACATGCAAGAGTCATCATTCCTGTG-AGAATGCCCTACAAATCAAC------AA
R-A_platyrhynchos : TTACCAC-A-AAAGCATGGCACTGAAGCTGCCAAGACGGCACACGAACATGCCTGCGGACA-AAAGACTTAGTCCTAACCTTACAGTTGGTTTTTGCTAGACATATACATGCAAGTATCCGCGCCCCAGTGTA-AATGCCCTCAATAGCCTTCAC-CCCA
A-A_platyrhynchos : TTACCAC-A-AAAGCATGGCACTGAAGCTGCCAAGACGGCACACGAACATGCCTGCGGACA-AAAGACTTAGTCCTAACCTTACAGTTGGTTTTTGCTAGACATATACATGCAAGTATCCGCGCCCCAGTGTA-AATGCCCTCAATAGCCTTCAC-CCCA
R-G_gallus : TTAACCC-ACAAAGCATGGCACTGAAGATGCCAAGATGGTAC-CTACTATACCTGTGGGCA-AAAGACTTAGTCCTAACCTTTCTATTGGTTTTTGCTAGACATATACATGCAAGTATCCGCATCCCAGTGAA-AATGCCCCCAA-ACCTTTCTT-CCCA
A-G_gallus : TTAACCC-ACAAAGCATGGCACTGAAGATGCCAAGATGGTAC-CTACTATACCTGTGGGCA-AAAGACTTAGTCCTAACCTTTCTATTGGTTTTTGCTAGACATATACATGCAAGTATCCGCATCCCAGTGAA-AATGCCCCCAA-ACCTTTCTT-CCCA
 TTAATAAWATAAAGCAAGGCACTGAAAATGCCTAGATGAGTCACCAYGACRTCCATAAACACAAAGGTTTGGTCCTAGCCTTYCTATTARTTTTTAGTAGACTTACACATGCAAGCATCCACACCCCAGTGAGAAATGCCCTCAAAATCTACCTCTCAAA

 * 1460 * 1480 * 1500 * 1520 * 1540 * 1560 * 1580 * 1600
R-E_caballus : GATTAAAAGGAGCAGGTATCAAGCACAC-TAG-AAAGTAGCTCATAACACCTTGCT-CAGCCACACCCCCACGGG-ACACAGCAGTGATAAAAATTAAGCTATGAACGAAAGTTCGACTAAGTCATATTAAA--------TAAGGGTTGGTAAATTTCGT
A-E_caballus : GATTAAAAGGAGCAGGTATCAAGCACAC-TAG-AAAGTAGCTCATAACACCTTGCT-CAGCCACACCCCCACGGG-ACACAGCAGTGATAAAAATTAAGCTATGAACGAAAGTTCGACTAAGTCATATTAAA--------TAAGGGTTGGTAAATTTCGT
R-C_bactrianus : GAACAAAAGGAGCAGGCATCAAGCACAC-AAA-CCTGTAGCTAAAGACGCCTTGCT-TAGCCACACCCCCACGGG-ACACAGCAGTAACAAAAATTGAGTTATAAACGGAAGTTTGACTAAGTTATATTAT---------TCAGGGCCGGTAAATTTCGT
A-C_bactrianus : GAACAAAAGGAGCAGGCATCAAGCACAC-AAA-CCTGTAGCTAAAGACGCCTTGCT-TAGCCACACCCCCACGGG-ACACAGCAGTAACAAAAATTGAGTTATAAACGGAAGTTTGACTAAGTTATATTAT---------TCAGGGCCGGTAAATTTCGT
R-C_lupus_familiaris : GATCTAAAGGAGCAGGTATCAAGCACAC-TCT-TAAGTAGCTCATAACACCTTGCT-AAGCCACACCCCCACGGG-ATACAGCAGTGATAAAAATTAAGCCATAAACGAAAGTTTGACTAAGCCATACTAA---------ATAGGGTTGGTAAATTTCGT
A-C_lupus_familiaris : GATCTAAAGGAGCAGGTATCAAGCACAC-TCT-TAAGTAGCTCATAACACCTTGCT-AAGCCACACCCCCACGGG-ATACAGCAGTGATAAAAATTAAGCCATAAACGAAAGTTTGACTAAGCCATACTAA---------ATAGGGTTGGTAAATTTCGT
R-N_procyonoides : GATTTAAAGGAGCAGGTATCAAGCGCAC-TCT-TAAGTAGCTCACAACACCTTGCT-AAGCCACACCCCCACGGG-ATACAGCAGTGATAAAAATTAAGCCATGAACGAAAGTTCGACTAAGTCATACTAA---------AAAGGGTTGGTAAATTTCGT
A-N_procyonoides : GATTTAAAGGAGCAGGTATCAAGCGCAC-TCT-TAAGTAGCTCACAACACCTTGCT-AAGCCACACCCCCACGGG-ATACAGCAGTGATAAAAATTAAGCCATGAACGAAAGTTCGACTAAGTCATACTAA---------AAAGGGTTGGTAAATTTCGT
R-V_lagopus : GATCTAAAGGAGCAGGTATCAAGCGCAC-TCT-TAAGTAGCTCATAACACCTTGCT-AAGCCACACCCCCACGGG-ATACAGCAGTGATAAAAATTAAGCCATGAACGAAAGTTCGACTAAGTTATGCTAA---------AGAGGGTTGGTAAATTTCGT
A-V_lagopus : GATCTAAAGGAGCAGGTATCAAGCGCAC-TCT-TAAGTAGCTCATAACACCTTGCT-AAGCCACACCCCCACGGG-ATACAGCAGTGATAAAAATTAAGCCATGAACGAAAGTTCGACTAAGTTATGCTAA---------AGAGGGTTGGTAAATTTCGT
R-M_putorius : GATTAAAAGGAGCGGGTATCAAGCACAC-TAAATTAGTAGCTCATAACGCCTTGCT-CAACCACACCCCCACGGG-ATACAGCAGTGATAAAAATTAAGCCATAAACGAAAGTTTGACTAAGCCATGTTAAC--------AAAGAGCTGGTAAATTTCGT
A-M_putorius : GATTAAAAGGAGCGGGTATCAAGCACAC-TAAATTAGTAGCTCATAACGCCTTGCT-CAACCACACCCCCACGGG-ATACAGCAGTGATAAAAATTAAGCCATAAACGAAAGTTTGACTAAGCCATGTTAAC--------AAAGAGCTGGTAAATTTCGT
R-B_taurus : AACTAAGAGGAGCTGGCATCAAGCACAC--ACCCT-GTAGCTCACGACGCCTTGCT-TAACCACACCCC-ACGGG-AAACAGCAGTGACAAAAATTAAGCCATAAACGAAAGTTTGACTAAGTTATATTAA---------TTAGGGTTGGTAAATCTCGT
A-B_taurus : AACTAAGAGGAGCTGGCATCAAGCACAC--ACCCT-GTAGCTCACGACGCCTTGCT-TAACCACACCCCCACGGG-AAACAGCAGTGACAAAAATTAAGCCATAAACGAAAGTTTGACTAAGTTATATTAA---------TTAGGGTTGGTAAATCTCGT
R-O_aries : GACTAAAAGGAGCAGGTATCAAGCACAC--ACTCTTGTAGCTCACAACGCCTTGCT-TAACCACACCCCCACGGG-AGACAGCAGTAACAAAAATTAAGCCATAAACGAAAGTTTGACTAAGTCATATTGA---------CCAGGGTTGGTAAATCTCGT
A-O_aries : GACTAAAAGGAGCAGGTATCAAGCACAC--ACTCTTGTAGCTCACAACGCCTTGCT-TAACCACACCCCCACGGG-AGACAGCAGTAACAAAAATTAAGCCATAAACGAAAGTTTGACTAAGCCATATTGA---------CTAGGGTTGGTAAATCTCGT
R-S_scrofa : GATCAAAAGGAGCAGGTATCAAGCACACCTATAACGGTAGCTCATAACGCCTTGCT-CAACCACACCCCCACGGG-AAACAGCAGTGATAAAAATTAAGCCATGAACGAAAGTTTGACTAAGTTATATTAA---------TTAGAGTTGGTAAATCTCGT
A-S_scrofa : GATCAAAAGGAGCAGGTATCAAGCACACCTATAACGGTAGCTCATAACGCCTTGCT-CAACCACACCCCCACGGG-AAACAGCAGTGATAAAAATTAAGCCATGAACGAAAGTTTGACTAAGTTATATTAA---------TTAGAGTTGGTAAATCTCGT
R-O_cuniculus : GATCAAGAGGAGCGGACATTAAGCACAC-TAA-TCAGTAGCTCAAGATGCCTTGCT-TAACCACACCCCCAAGGG-ATACAGCAGTGATAAATATTTAGCAATGAACGTAAGTTTGACTAAGTTATGCTACT--------TTAGGGTTGGTAAATCTCGT
A-O_cuniculus : GATCAAGAGGAGCGGACATTAAGCACAC-TAA-TCAGTAGCTCAAGATGCCTTGCT-TAACCACACCCCCAAGGG-ATACAGCAGTGATAAATATTTAGCAATGAACGTAAGTTTGACTAAGTTATGCTACT--------TTAGGGTTGGTAAATCTCGT
R-M_musculus : AATTTAA-GGAGAGGGTATCAAGCACAT-TAA--AA-TAGCTTAAGACACCTTGCC-TAGCCACACCCCCACGGG-ACTCAGCAGTGATAAATATTAAGCAATAAACGAAAGTTTGACTAAGTTATAC-----CTC----TTAGGGTTGGTAAATTTCGT
A-M_musculus : AATTTAA-GGAGAGGGTATCAAGCACAT-TAA--AA-TAGCTTAAGACACCTTGCC-TAGCCACACCCCCACGGG-ACTCAGCAGTGATAAATATTAAGCAATAAACGAAAGTTTGACTAAGTTATAC-----CTC----TTAGGGTTGGTAAATTTCGT
R-R_norvegicus : AACTTAA-GGAGAGGGCATCAAGCACAT--AA--TA-TAGCTCAAGACGCCTTGCC-TAGCCACACCCCCACGGG-ACTCAGCAGTGATAAATATTAAGCAATGAACGAAAGTTTGACTAAGCTATAC-----CTC----TCAGGGTTGGTAAATTTCGT
A-R_norvegicus : AACTTAA-GGAGAGGGCATCAAGCACAT--AA--TA-TAGCTCAAGACGCCTTGCC-TAGCCACACCCCCACGGG-ACTCAGCAGTGATAAATATTAAGCAATGAACGAAAGTTTGACTAAGCTATAC-----CTC----TCAGGGTTGGTAAATTTCGT
R-M_coypus : GATCTAAAGGAGCTGGTATCAAGCACAC-TAA--CAGTAGCTCACAACACCTTGCT-TAGCCACACCCCCACGGG-ATACAGCAGTAATCAAAATTAAGCTATAAACGAAAGTTCGACTAAGTCATGCAATTTCTT----ATAGGGTTGGTAAATCTCGT
A-M_coypus : GATCTAAAGGAGCTGGTATCAAGCACAC-TAA--CAGTAGCTCACAACACCTTGCT-TAGCCACACCCCCACGGG-ATACAGCAGTAATCAAAATTAAGCTATAAACGAAAGTTCGACTAAGTCATGCAATTTCTT----ATAGGGTTGGTAAATCTCGT
R-A_platyrhynchos : GGCCTTAAGGAGCGGGTATCAGGCACACCCAA-GCAGTAGCCCAAGACGCCTTGCT-AAGCCACGCCCCCACGGGTATTCAGCAGTAGTTAACATTAAGCAATGAGTGCAAACTCGACTTAGTCATAGCAAGCCTCCACCCAAGGGTCGGTAAATCTTGT
A-A_platyrhynchos : GGCCTTAAGGAGCGGGTATCAGGCACACCCAA-GCAGTAGCCCAAGACGCCTTGCT-AAGCCACGCCCCCACGGGTATTCAGCAGTAGTTAACATTAAGCAATGAGTGCAAACTCGACTTAGTCATAGCAAGCCTCCACCCAAGGGTCGGTAAATCTTGT
R-G_gallus : AGCAA-AAGGAGCAGGTATCAGGCACACTCA--GCAGTAGCCCAAGACGCCTTGCTTAAGCCACACCCCCACGGGTACTCAGCAGTAATTAACCTTAAGCAATAAGTGTAAACTTGACTTAGCCATAGCAACCC--------AGGGTTGGTAAATCTTGT
A-G_gallus : AGCAA-AAGGAGCAGGTATCAGGCACACTCA--GCAGTAGCCCAAGACGCCTTGCTTAAGCCACACCCCCACGGGTACTCAGCAGTAATTAACCTTAAGCAATAAGTGTAAACTTGACTTAGCCATAGCAACCC--------AGGGTTGGTAAATCTTGT
 GATYAAAAGGAGCAGGTATCAAGCACACCTAAATCAGTAGCTCAAAACGCCTTGCTTTAGCCACACCCCCACGGGTATACAGCAGTGATAAAAATTAAGCCATAAACGAAAGTTTGACTAAGTCATACTAAYCCTCCACCTTAGGGTTGGTAAATTTCGT

 * 1620 * 1640 * 1660 * 1680 * 1700 * 1720 * 1740 * 1760
R-E_caballus : GCCAGCCACCGCGGTCATACGATTAACCCAAATTAATAAATC----TCCGGCGTAAAGCGTGTCAAA--GACTAATA--CCAAAATAAAGTTAAAACCCAGTTAAGCCGTAAAAAGCTACAACCA-AAGTAAAA-TAGACTACGAAAGTGACTTTAATAC
A-E_caballus : GCCAGCCACCGCGGTCATACGATTAACCCAAATTAATAAACCC---TCCGGCGTAAAGCGTGTCAAA--GACTAATA--CCAAAATAAAGTTAAAACCCAGTTAAGCCGTAAAAAGCTACAACCA-AAGTAAAA-TAGACTACGAAAGTGACTTTAATAC
R-C_bactrianus : GCCAGCCACCGCGGTCATACGATTAGCCCGAATTAATGGAAA----TCCGGCGTAAAGCGTGTTAAT--GAGTGACT--ATCAAATAGAGTTAAGTCTTGGCCAAGATGTAAAAATCTATGACCA-ACGCAAAAATAAACTACGAAAGTGACCCTAATAC
A-C_bactrianus : GCCAGCCACCGCGGTCATACGATTAGCCCGAATTAATGGAAA----TCCGGCGTAAAGCGTGTTAAT--GAGTGACT--ATCAAATAGAGTTAAGTCTTGGCCAAGATGTAAAAATCTATGACCA-ACGCAAAAATAAACTACGAAAGTGACCCTAATAC
R-C_lupus_familiaris : GCCAGCCACCGCGGTCATACGATTAACCCAAACTAATAGGCC----TACGGCGTAAAGCGTGTTCAA--GA-TACTT--TTACACTAAAGTTAAAACTTAACTAAGCCGTAAAAAGCTACAGTTA-TCATAAAA-TAAACCACGAAAGTGACTTTATAAT
A-C_lupus_familiaris : GCCAGCCACCGCGGTCATACGATTAACCCAAACTAATAGGCC----TACGGCGTAAAGCGTGTTCAA--GA-TACTT--TTACACTAAAGTTAAAACTTAACTAAGCCGTAAAAAGCTACAGTTA-TCATAAAA-TAAACCACGAAAGTGACTTTATAAT
R-N_procyonoides : GCCAGCCACCGCGGTCATACGATTAACCCAAACTAATAGGCC----CACGGCGTAAAGCGTGTTTAA--GA-TATCA--ACTAACTAAAGTTAAAACTTAACTAAGCTGTAAAAAGCTCCAGTTA-CCATAAAA-TAAACTACGAAAGTGACTTTAAAAT
A-N_procyonoides : GCCAGCCACCGCGGTCATACGATTAACCCAAACTAATAGGCC----CACGGCGTAAAGCGTGTTTAA--GA-TATCA--ACTAACTAAAGTTAAAACTTAACTAAGCTGTAAAAAGCTCCAGTTA-CCATAAAA-TAAACTACGAAAGTGACTTTAAAAT
R-V_lagopus : GCCAGCCACCGCGGTCATACGATTAACCCAAACTAATAGGAC----AACGGCGTAAAGCGTGTTTAA--GA-TGACA--CATCACTAAAGTTAAAACTTAACTAAGCCGTAAAAAGCTACAGTTA-CAATAAAA-TATACTACGAAAGTGACTTTAAAAT
A-V_lagopus : GCCAGCCACCGCGGTCATACGATTAACCCAAACTAATAGGAC----AACGGCGTAAAGCGTGTTTAA--GA-TGACA--CATCACTAAAGTTAAAACTTAACTAAGCCGTAAAAAGCTACAGTTA-CAATAAAA-TATACTACGAAAGTGACTTTAAAAT
R-M_putorius : GCCAGCCACCGCGGTCATACGATTAGCCCGAATCAATAGGCA----AACGGCGTAAAACGTGTTAAG--GA-TTATA--TTATATTAAAGTTAAAATTTGACAAGGCTGTAAAAAGCTACTGTTA-ATATAAGA-TAAACCACGAAAGTGACTTTATTAC
A-M_putorius : GCCAGCCACCGCGGTCATACGATTAGCCCGAATCAATAGGCA----AACGGCGTAAAACGTGTTAAG--GA-TTATA--TTATATTAAAGTTAAAATTTGACAAGGCTGTAAAAAGCTACTGTTA-ATATAAGA-TAAACCACGAAAGTGACTTTATTAC
R-B_taurus : GCCAGCCACCGCGGTCATACGATTAACCCAAGCTAACAGGAG----TACGGCGTAAAACGTGTTAAA--GCACCATA--CCA-AATAGGGTTAAATTCTAACTAAGCTGTAAAAAGCCATGATTA-AAATAAAAATAAATGACGAAAGTGACCCTA-CAA
A-B_taurus : GCCAGCCACCGCGGTCATACGATTAACCCAAGCTAACAGGAG----TACGGCGTAAAACGTGTTAAA--GCACCATA--CCA-AATAGGGTTAAATTCTAACTAAGCTGTAAAAAGCCATGATTA-AAATAAAAATAAATGACGAAAGTGACCCTA-CAA
R-O_aries : GCCAGCCACCGCGGTCATACGATTGACCCAAGCTAACAGGAG----TACGGCGTAAAGCGTGTTAAA--GCATCATA--CTA-AATAGAGTTAAATTTTAATTAAACTGTAAAAAGCCATAATTA-TAACAAAAATAAATGACGAAAGTAACCCTA-CAA
A-O_aries : GCCAGCCACCGCGGTCATACGATTGACCCAAGCTAACAGGAG----TACGGCGTAAAGCGTGTTAAA--GCATCATA--CTA-AATAGAGTTAAATTTTAATTAAACTGTAAAAAGCCATAATTA-TAACAAAAATAAATGACGAAAGTAACCCTA-CAA
R-S_scrofa : GCCAGCCACCGCGGTCATACGATTAACCCAAATTAATAGATC----CACGGCGTAAAGAGTGTTTAA--GAAAAAAAAACCACAATAGAGTTAAATTATAACTAAGCTGTAAAAAGCCCTAGTTA-AAATAAAA-TAACCCACGAAAGTGACTCTAATAA
A-S_scrofa : GCCAGCCACCGCGGTCATACGATTAACCCAAATTAATAGATC----CACGGCGTAAAGAGTGTTTAA--GAAAAAAAAACCACAATAGAGTTAAATTATAACTAAGCTGTAAAAAGCCCTAGTTA-AAATAAAA-TAACCCACGAAAGTGACTCTAATAA
R-O_cuniculus : GCCAGCCACCGCGGTCATACGATTAACCCAAATTAATAAATA----TCCGGCGTAAAGCGTGATTAG--AATAAACA--ACAAAATAAAATCAAATAACAACTAAGCTGTAGAAAGTAATAGTTGCAAACAAAAATAAACAACGAAAGTGATTTTA-TAC
A-O_cuniculus : GCCAGCCACCGCGGTCATACGATTAACCCAAATTAATAAATA----TCCGGCGTAAAGCGTGATTAG--AATAAACA--ACAAAATAAAATCAAATAACAACTAAGCTGTAAAAAGTAATAGTTGCAAACAAAAATAAACAACGAAAGTGATTTTA-TAC
R-M_musculus : GCCAGCCACCGCGGTCATACGATTAACCCAAACTAATTATCT-----TCGGCGTAAAACGTGTCAAC--TATAAATA--AATAAATAGAATTAAAATCCAACTTATATGTGAAAATTCATTGTTAGGACCTAAACTCAATAACGAAAGTAATTCTAGTCA
A-M_musculus : GCCAGCCACCGCGGTCATACGATTAACCCAAACTAATTATCT-----TCGGCGTAAAACGTGTCAAC--TATAAATA--AATAAATAGAATTAAAATCCAACTTATATGTGAAAATTCATTGTTAGGACCTAAACTCAATAACGAAAGTAATTCTAGTCA
R-R_norvegicus : GCCAGCCACCGCGGTCATACGATTAACCCAAACTAATTATTT-----TCGGCGTAAAACGTGCCAAC--TATAAATC--TCATAATAGAATTAAAATCCAACTTATATGTGAAAATTCATTGTTAGGACCTAAGCCCAATAACGAAAGTAATTCTAATCA
A-R_norvegicus : GCCAGCCACCGCGGTCATACGATTAACCCAAACTAATTATTT-----TCGGCGTAAAACGTGCCAAC--TATAAATC--TCATAATAGAATTAAAATCCAACTTATATGTGAAAATTCATTGTTAGGACCTAAGCCCAATAACGAAAGTAATTCTAATCA
R-M_coypus : GCCAGCCACCGCGGTCATACGATTAACCCTAATTAATAAACC-----CCGGCGTAAAGAGTATTAAA--GATACA-A--TAAAAATAAGATTAAATTTCATCTGGGTCGTAAAAAACTATAGATAAAAA-TAAAATCGATAACGAAGGTAATCTTAATAT
A-M_coypus : GCCAGCCACCGCGGTCATACGATTAACCCTAATTAATAAACC-----CCGGCGTAAAGAGTATTAAA--GATACA-A--TAAAAATAAGATTAAATTTCATCTGGGTCGTAAAAAACTATAGATAAAAA-TAAAATCGATAACGAAGGTAATCTTAATAT
R-A_platyrhynchos : GCCAGCCACCGCGGTCATACAAGAGACCCAAATCAACTGTCCTACAAGCGGCGTAAAGAGTGGTAAGATGCCTATCCTACCTAACTAAGATCAAAATGCAACTAAGCTGTCGCAAGCACAAGATG-CACCTAAA-CACACCATCAAGATGATCTTAGAAA
A-A_platyrhynchos : GCCAGCCACCGCGGTCATACAAGAGACCCAAATCAACTGTCCTACAAGCGGCGTAAAGAGTGGTAAGATGCCTATCCTACCTAACTAAGATCAAAATGCAACTAAGCTGTCGCAAGCACAAGATG-CACCTAAA-CACACCATCAAGATGATCTTAGAAA
R-G_gallus : GCCAGCCACCGCGGTCATACAAGAAACCCAAATCAATAG--CTAC--CCGGCGTAAAGAGTGGCCACATGT-TATCTGCACCAGCTAAGATTAAAATGCAACCAAGCTGTCATAAGCCTAAGATC-CACCTAAA-CCCAAC-CCAAATCCATCTTAGCCT
A-G_gallus : GCCAGCCACCGCGGTCATACAAGAAACCCAAATCAATAG--CTAC--CCGGCGTAAAGAGTGGCCACATGT-TATCTGCACCAGCTAAGATTAAAATGCAACCAAGCTGTCATAAGCCTAAGATC-CACCTAAA-CCCAAC-CCAAATCCATCTTAGCCT
 GCCAGCCACCGCGGTCATACGATTAACCCAAATTAATAGGCCTACATACGGCGTAAAGCGTGTTAAAATGATTAATADACCAAAATAAAGTTAAAATTTAACTAAGCTGTAAAAAGCTATAGTTAGAAAYAAAAATAAACCACGAAAGTGACTTTAATAA

 * 1780 * 1800 * 1820 * 1840 * 1860 * 1880 * 1900 * 1920
R-E_caballus : -CTCTG---------ACTACACGATAGCTAAGACCCAAACTGGGATTAGATACCCCACTATGCTTAGCCCTAAACTAAAATAGCTTACCACAACAAAGCTATTCGCCAGAGTACTACTAG--CAACAGCCTAAAACTCAAAGGACTTGGCGGTGCTTTAC
A-E_caballus : -CTCTG---------ACTACACGATAGCTAAGACCCAAACTGGGATTAGATACCCCACTATGCTTAGCCCTAAACTAAAATAGCTTACCACAACAAAGCTATTCGCCAGAGTACTACTAG--CAACAGCCTAAAACTCAAAGGACTTGGCGGTGCTTTAC
R-C_bactrianus : GATCT----------ACTACACGACAGCTAAGGTCCAAACTGGGATTAGATACCCCACTATGCTTAGCCTTAAACCTAGGTGA-TTACAACAACAAAATCACTCGCCAGAGTACTACTAG--CAACAGCTTAAAACTCAAAGGACTTGGCGGTGCTTCAT
A-C_bactrianus : GATCT----------ACTACACGACAGCTAAGGTCCAAACTGGGATTAGATACCCCACTATGCTTAGCCTTAAACCTAGGTGA-TTACAACAACAAAATCACTCGCCAGAGTACTACTAG--CAACAGCTTAAAACTCAAAGGACTTGGCGGTGCTTCAT
R-C_lupus_familiaris : AATCTG---------ACTACACGATAGCTAAGACCCAAACTGGGATTAGATACCCCACTATGCTTAGCCCTAAACATAGATAA-TT-TTACAACAAAATAATTCGCCAGAGGACTACTAG--CAATAGCTTAAAACTCAAAGGACTTGGCGGTGCTTTAT
A-C_lupus_familiaris : AATCTG---------ACTACACGATAGCTAAGACCCAAACTGGGATTAGATACCCCACTATGCTTAGCCCTAAACATAGATAA-TT-TTACAACAAAATAATTCGCCAGAGGACTACTAG--CAATAGCTTAAAACTCAAAGGACTTGGCGGTGCTTTAT
R-N_procyonoides : ACTCTG---------ACTACACGATAGCTAAGATCCAAACTGGGATTAGATACCCCACTATGCTTAGCCCTAAACATAAATAA-TT-CTACAACAAAATAATTCGCCAGAGGACTACTAG--CAACAGCTTAAAACTCAAAGGACTTGGCGGTGCTTTAT
A-N_procyonoides : ACTCTG---------ACTACACGATAGCTAAGATCCAAACTGGGATTAGATACCCCACTATGCTTAGCCCTAAACATAAATAA-TT-CTACAACAAAATAATTCGCCAGAGGACTACTAG--CAACAGCTTAAAACTCAAAGGACTTGGCGGTGCTTTAT
R-V_lagopus : TTTCTG---------ACTACACGATAGCTAAGACCCAAACTGGGATTAGATACCCCACTATGCTTAGCCCTAAACATAAATAG-TT-CTACAACAAAACAATTCGCCAGAGAACTACTAG--CAACAGCTTAAAACTCAAAGGACTTGGCGGTGCTTTAT
A-V_lagopus : TTTCTG---------ACTACACGATAGCTAAGACCCAAACTGGGATTAGATACCCCACTATGCTTAGCCCTAAACATAAATAG-TT-CTACAACAAAACAATTCGCCAGAGAACTACTAG--CAACAGCTTAAAACTCAAAGGACTTGGCGGTGCTTTAT
R-M_putorius : -TTCCA---------TCAACACGATAGCTGAGACCCAAACTGGGATTAGATACCCCACTATGCTCAGCCCTAAACATAAATAA-TTATCACAACAAAATTATCTGCCAGAGAACTACTAG--CAATAGCTTAAAACTCAAAGGACTTGGCGGTGCTTTAC
A-M_putorius : -TTCCA---------TCAACACGATAGCTGAGACCCAAACTGGGATTAGATACCCCACTATGCTCAGCCCTAAACATAAATAA-TTATCACAACAAAATTATCTGCCAGAGAACTACTAG--CAATAGCTTAAAACTCAAAGGACTTGGCGGTGCTTTAC
R-B_taurus : TAGCCG---------AC-GCACTATAGCTAAGACCCAAACTGGGATTAGATACCCCACTATGCTTAGCCCTAAACACAGATAA-TTACATAAACAAAATTATTCGCCAGAGTACTACTAG--CAACAGCTTAAAACTCAAAGGACTTGGCGGTGCTTTAT
A-B_taurus : TAGCCG---------AC-GCACTATAGCTAAGACCCAAACTGGGATTAGATACCCCACTATGCTTAGCCCTAAACACAGATAA-TTACATAAACAAAATTATTCGCCAGAGTACTACTAG--CAACAGCTTAAAACTCAAAGGACTTGGCGGTGCTTTAT
R-O_aries : TAGCTG---------AT-ACACCATAGCTAAGACCCAAACTGGGATTAGATACCCCACTATGCTTAGCCCTAAACACAAATAA-TTATAAAAACAAAATTATTCGCCAGAGTACTACCG---CAACAGCCCGAAACTCAAAGGACTTGGCGGTGCTTTAT
A-O_aries : TAGCTG---------AT-ACACCATAGCTAAGACCCAAACTGGGATTAGATACCCCACTATGCTTAGCCCTAAACACAAATAA-TTATAAGAACAAAATTATTCGCCAGAGTACTACCGG--CAACAGCCCGAAACTCAAAGGACTTGGCGGTGCTTTAT
R-S_scrofa : TC-CTG---------AC-ACACGATAGCTAGGACCCAAACTGGGATTAGATACCCCACTATGCCTAGCCCTAAACCCAAATAG-TTACAT-AACAAAACTATTCGCCAGAGTACTACTCG--CAACTGCCTAAAACTCAAAGGACTTGGCGGTGCTTCAC
A-S_scrofa : TC-CTG---------AC-ACACGATAGCTAGGACCCAAACTGGGATTAGATACCCCACTATGCCTAGCCCTAAACCCAAATAG-TTACAT-AACAAAACTATTCGCCAGAGTACTACTCG--CAACTGCCTAAAACTCAAAGGACTTGGCGGTGCTTCAC
R-O_cuniculus : TCTTCG---------AACTCACGATAGCTAAGGCCCAAACTGGGATTAGATACCCCACTATGCTTAGCCCTAAACTTTGATAA-TT-TCATAACAAAATTATTCGCCAGAGAACTACAAG--CCAAAGCTTAAAACTCAAAGGACTTGGCGGTGCTTTAT
A-O_cuniculus : TCTTCG---------AACTCACGATAGCTAAGGCCCAAACTGGGATTAGATACCCCACTATGCTTAGCCCTAAACTTTGATAA-TT-TCATAACAAAATTATTCGCCAGAGAACTACAAG--CCAAAGCTTAAAACTCAAAGGACTTGGCGGTGCTTTAT
R-M_musculus : TTTATA----------ATACACGACAGCTAAGACCCAAACTGGGATTAGATACCCCACTATGCTTAGCCATAAACCTAAATAATTAAATTTAACAAAACTATTTGCCAGAGAACTACTAG--CCATAGCTTAAAACTCAAAGGACTTGGCGGTACTTTAT
A-M_musculus : TTTATA----------ATACACGACAGCTAAGACCCAAACTGGGATTAGATACCCCACTATGCTTAGCCATAAACCTAAATAATTAAATTTAACAAAACTATTTGCCAGAGAACTACTAG--CCATAGCTTAAAACTCAAAGGACTTGGCGGTACTTTAT
R-R_norvegicus : TTTATAT--------AATGCACGATAGCTAAGACCCAAACTGGGATTAGATACCCCACTATGCTTAGCCCTAAACCTTAATAATTAAACCTA-CAAAATTATTTGCCAGAGAACTACTAG--CTACAGCTTAAAACTCAAAGGACTTGGCGGTACTTTAT
A-R_norvegicus : TTTATAT--------AATGCACGATAGCTAAGACCCAAACTGGGATTAGATACCCCACTATGCTTAGCCCTAAACCTTAATAATTAAACCTA-CAAAATTATTTGCCAGAGAACTACTAG--CTACAGCTTAAAACTCAAAGGACTTGGCGGTACTTTAT
R-M_coypus : ATCAGA----------ATATACTAAAGCTAAGACACAAACTGGGATTAGATACCCCACTATGCTTAGTTGTAAACACAGATTCTTAA---CAACAAAAATATCCGCCAGAGAACTACTAG--CAACAGCTTAAAACTCAAAGGACTTGGCGGTGCTTTAA
A-M_coypus : ATCAGA----------ATATACTAAAGCTAAGACACAAACTGGGATTAGATACCCCACTATGCTTAGTTGTAAACACAGATTCTTAA---CAACAAAAATATCCGCCAGAGAACTACTAG--CAACAGCTTAAAACTCAAAGGACTTGACGGTGCTTTAA
R-A_platyrhynchos : CTAGCGATTAATTTGAACCCACGAAAGCCAGGGCCCAAACTGGGATTAGATACCCCACTATGCCTGGCCCTAAATCTTGATAC-TTACCCT-ACCGAAGTATCCGCCAGAGAACTACGAGCACAAACGCTTAAAACTCTAAGGACTTGGCGGTGCCCTAA
A-A_platyrhynchos : CTAGCGATTAATTTGAACCCACGAAAGCCAGGGCCCAAACTGGGATTAGATACCCCACTATGCCTGGCCCTAAATCTTGATAC-TTACCCT-ACCGAAGTATCCGCCAGAGAACTACGAGCACAAACGCTTAAAACTCTAAGGACTTGGCGGTGCCCTAA
R-G_gallus : C-AACGATTAATTTTAACCCACGAAAGCTAGGACCCAAACTGGGATTAGATACCCCACTATGCCTAGCCCTAAATCTAGATAC-CTCCCATCACACATGTATCCGCCTGAGAACTACGAGCACAAACGCTTAAAACTCTAAGGACTTGGCGGTGCCCCAA
A-G_gallus : C-AACGATTAATTTTAACCCACGAAAGCTAGGACCCAAACTGGGATTAGATACCCCACTATGCCTAGCCCTAAATCTAGATAC-CTCCCATCACACATGTATCCGCCTGAGAACTACGAGCACAAACGCTTAAAACTCTAAGGACTTGGCGGTGCCCCAA
 TTTCTGATTAATTTKACTACACGATAGCTAAGACCCAAACTGGGATTAGATACCCCACTATGCTTAGCCCTAAACATAAATAATTTACCACAACAAAATTATTCGCCAGAGAACTACTAGCACAACAGCTTAAAACTCAAAGGACTTGGCGGTGCTTTAT

 * 1940 * 1960 * 1980 * 2000 * 2020 * 2040 * 2060 * 2080
R-E_caballus : ATCCCTCTAGAGGAGCCTGTTCCATAATCGATAAACCCCGATAAACCCCACCATCCCTTGCTA-ATTCAGCCTATATACCGCCATCTTCAGCAAACCCTAAAC-AAGGTACCGAAGTAAGCACAAATATCCAA-CATAAAAACGTTAGGTCAAGGTGTAG
A-E_caballus : ATCCCTCTAGAGGAGCCTGTTCCATAATCGATAAACCCCGATAAACCCCACCATCCCTTGCTA-ATTCAGCCTATATACCGCCATCTTCAGCAAACCCTAAAC-AAGGTACCGAAGTAAGCACAAATATCCAA-CATAAAAACGTTAGGTCAAGGTGTAG
R-C_bactrianus : A-CCCCCTAGAGGAGCCTGTTCTATAATCGATAAACCCCGATCAACCTCACCAACCCTTGCTA-ATTCAGTCTATATACCGCCATCTCCAGCAAACCCCTAT--AGGGATCAATAGTAAGCTTAACTATTCAAACATAAAAACGTTAGGTCAAGGTGTAA
A-C_bactrianus : A-CCCCCTAGAGGAGCCTGTTCTATAATCGATAAACCCCGATCAACCTCACCAACCCTTGCTA-ATTCAGTCTATATACCGCCATCTCCAGCAAACCCCTAT--AGGGATCAATAGTAAGCTTAACTATTCAAACATAAAAACGTTAGGTCAAGGTGTAA
R-C_lupus_familiaris : ATCCCTCTAGAGGAGCCTGTTCTATAATCGATAAACCCCGATAAACCTCACCACCTTTCGCTA-ATTCAGTCTATATACCGCCATCTTCAGCAAACCCTCAA--AAGGTAGAACAGTAAGCACAATCATTTTA-CATAAAAAAGTTAGGTCAAGGTGTAA
A-C_lupus_familiaris : ATCCCTCTAGAGGAGCCTGTTCTATAATCGATAAACCCCGATAAACCTCACCACCTTTCGCTA-ATTCAGTCTATATACCGCCATCTTCAGCAAACCCTCAA--AAGGTAGAACAGTAAGCACAATCATTTTA-CATAAAAAAGTTAGGTCAAGGTGTAA
R-N_procyonoides : ATCCCTCTAGAGGAGCCTGTTCTGTAATCGATAAACCCCGATAAACCTCACCATCCCTTGCTA-ATACAGTCTATATACCGCCATCTTCAGCAAACCCTCAA--AAGGCAGAGTAGTAAGCATAATCATTTTA-CATAAAAAAGTTAGGTCAAGGTGTAA
A-N_procyonoides : ATCCCTCTAGAGGAGCCTGTTCTGTAATCGATAAACCCCGATAAACCTCACCATCCCTTGCTA-ATACAGTCTATATACCGCCATCTTCAGCAAACCCTCAA--AAGGCAGAGTAGTAAGCATAATCATTTTA-CATAAAAAAGTTAGGTCAAGGTGTAA
R-V_lagopus : ATCCCTCTAGAGGAGCCTGTTCTATAATCGATAAACCCCGATAAACCTCACCATCTCTTGCTA-ATACAGTCTATATACCGCCATCTTCAGCAAACCCTTAA--AAGGCAGAGCAGTAAGCAAGATCATCACG-CATAAAAAAGTTAGGTCAAGGTGTAA
A-V_lagopus : ATCCCTCTAGAGGAGCCTGTTCTATAATCGATAAACCCCGATAAACCTCACCATCTCTTGCTA-ATACAGTCTATATACCGCCATCTTCAGCAAACCCTTAA--AAGGCAGAGCAGTAAGCAAGATCATCACG-CATAAAAAAGTTAGGTCAAGGTGTAA
R-M_putorius : ATCCCTCTAGAGGAGCCTGTTCTATAATCGATAAACCCCGATAGACCTCACCACTTCTAGCTT-AATCAGTCTATATACCGCCATCTTCAGCAAACCCTTAA--AGGGAAGAAAAGTAAGCACAATAATATTA-CATAAAAAAGTTAGGTCAAGGTGTAA
A-M_putorius : ATCCCTCTAGAGGAGCCTGTTCTATAATCGATAAACCCCGATAGACCTCACCACTTCTAGCTT-AATCAGTCTATATACCGCCATCTTCAGCAAACCCTTAA--AGGGAAGAAAAGTAAGCACAATAATATTA-CATAAAAAAGTTAGGTCAAGGTGTAA
R-B_taurus : ATCCTTCTAGAGGAGCCTGTTCTATAATCGATAAACCCCGATAAACCTCACCAATTCTTGCTA-ATACAGTCTATATACCGCCATCTTCAGCAAACCCTAAAA-AGGAAA-AAAAGTAAGCGTAATTATGATA-CATAAAAACGTTAGGTCAAGGTGTAA
A-B_taurus : ATCCTTCTAGAGGAGCCTGTTCTATAATCGATAAACCCCGATAAACCTCACCAATTCTTGCTA-ATACAGTCTATATACCGCCATCTTCAGCAAACCCTAAAA-AGGAAA-AAAAGTAAGCGTAATTATGATA-CATAAAAACGTTAGGTCAAGGTGTAA
R-O_aries : ACCCTTCTAGAGGAGCCTGTTCTATAATCGATAAACCCCGATAAACCTCACCAATCCTTGCTA-ATACAGTCTATATACCGCCATCTTCAGCAAACCCTAAAA-AAGGGACAAAAGTAAGCTCAATAATAACA-CATAAAGACGTTAGGTCAAGGTGTAA
A-O_aries : ACCCTTCTAGAGGAGCCTGTTCTATAATCGATAAACCCCGATAAACCTCACCAATCCTTGCTA-ATACAGTCTATATACCGCCATCTTCAGCAAACCCTAAAA-AAGGGACAAAAGTAAGCTCAATAATAACA-CATAAAGACGTTAGGTCAAGGTGTAA
R-S_scrofa : ATCCACCTAGAGGAGCCTGTTCTATAATCGATAAACCCCGATAGACCTTACCAACCCTTGCCA-ATTCAGCCTATATACCGCCATCTTCAGCAAACCCTAAAA-A-GGAACAATAGTAAGCACAATCATAACA-CATAAAAACGTTAGGTCAAGGTGTAG
A-S_scrofa : ATCCACCTAGAGGAGCCTGTTCTATAATCGATAAACCCCGATAGACCTTACCAACCCTTGCCA-ATTCAGCCTATATACCGCCATCTTCAGCAAACCCTAAAA-A-GGAACAATAGTAAGCACAATCATAACA-CATAAAAACGTTAGGTCAAGGTGTAG
R-O_cuniculus : ACCCACCTAGAGGAGCCTGTTCCGTAATCGATAAACCCCGATAAACCCTACCACTCTTTGCCA-ACTCAGCCTATATACCGCCATCTTCAGCGAACCCTAAA--AAGGAGCAAAAGTAAGCTCAATTAC--CACCGTAAAAACGTTAGGTCAAGGTGTAG
A-O_cuniculus : ACCCACCTAGAGGAGCCTGTTCCGTAATCGATAAACCCCGATAAACCTTACCACTCTTTGCCA-ACTCAGCCTATATACCGCCATCTTCAGCGAACCCTAAA--AAGGAGAAAAAGTAAGCTCAATTAC--TACCGTAAAAACGTTAGGTCAAGGTGTAG
R-M_musculus : ATCCATCTAGAGGAGCCTGTTCTATAATCGATAAACCCCGCTCTACCTCACCATCTCTTGCTA-ATTCAGCCTATATACCGCCATCTTCAGCAAACCCTAAA--AAGGTATTAAAGTAAGCAAAAGAATCAAA-CATAAAAACGTTAGGTCAAGGTGTAG
A-M_musculus : ATCCATCTAGAGGAGCCTGTTCTATAATCGATAAACCCCGCTCTACCTCACCATCTCTTGCTA-ATTCAGCCTATATACCGCCATCTTCAGCAAACCCTAAA--AAGGTATTAAAGTAAGCAAAAGAATCAAA-CATAAAAACGTTAGGTCAAGGTGTAG
R-R_norvegicus : ATCCATCTAGAGGAGCCTGTTCTATAATCGATAAACCCCGTTATACCTTACCCCTTCTCGCTA-ATTCAGCCTATATACCGCCATCTTCAGCAAACCCTAAA--AAGGCACTAAAGTAAGCACAAGAA-CAAA-CATAAAAACGTTAGGTCAAGGTGTAG
A-R_norvegicus : ATCCATCTAGAGGAGCCTGTTCTATAATCGATAAACCCCGTTCTACCTTACCCCTTCTCGCTA-ATTCAGCCTATATACCGCCATCTTCAGCAAACCCTAAA--AAGGCACTAAAGTAAGCACAAGAA-CAAA-CATAAAAACGTTAGGTCAAGGTGTAG
R-M_coypus : ACCCATCTAGAGGAGCCTGTTCTATAATCGATAAACCCCGATAAACCTCACCACTTCTCGCTA-ATTCAGTCTATATACCGCCATCTTCAGCAAACCCCAAC--AGGGATTAAAAGTAAGCACAACGATCAT--CATAAAAACGTTAGGTCAAGGTGTAG
A-M_coypus : ACCCATCTAGAGGAGCCTGTTCTATAATCGATAAACCCCGATAAACCTCACCACTTCTCGCTA-ATTCAGTCTATATACCGCCATCTTCAGCAAACCCCAAC--AGGGATTAAAAGTAAGCACAACGATCAT--CATAAAAACGTTAGGTCAAGGTGTAG
R-A_platyrhynchos : ACCCACCTAGAGGAGCCTGTTCTGTAATCGATGATCCACGATCAACCCAACCGCCCCTTGCCAAGCACAGCCTACATACCGCCGTCGCCAGCCCACCTCGAATGAGAGCGCAACAGTGGGCGCAACAGCACCC-CGCTAATAAGACAGGTCAAGGTATAG
A-A_platyrhynchos : ACCCACCTAGAGGAGCCTGTTCTGTAATCGATGATCCACGATCAACCCAACCGCCCCTTGCCAAGCACAGCCTACATACCGCCGTCGCCAGCCCACCTCGAATGAGAGCGCAACAGTGGGCGCAACAGCACCC-CGCTAATAAGACAGGTCAAGGTATAG
R-G_gallus : ACCCACCTAGAGGAGCCTGTTCTATAATCGATAATCCACGATTCACCCAACCACCCCTTGCCA-GCACAGCCTACATACCGCCGTCGCCAGCCCACCTCTAATGAAAGAACAACAGTGAGCTCAATAGCCCCT-CGCTAATAAGACAGGTCAAGGTATAG
A-G_gallus : ACCCACCTAGAGGAGCCTGTTCTATAATCGATAATCCACGATTCACCCAACCACCCCTTGCCA-GCACAGCCTACATACCGCCGTCGCCAGCCCACCTCTAATGAAAGAACAACAGTGAGCTCAATAGCCCCT-CGCTAATAAGACAGGTCAAGGTATAG
 ATCCATCTAGAGGAGCCTGTTCTATAATCGATAAACCCCGATAAACCTCACCACCCCTTGCTAAATTCAGTCTATATACCGCCATCTTCAGCAAACCCTAAAAGAAGGAACAAAAGTAAGCACAATAATCAYAMCATAAAAACGTTAGGTCAAGGTGTAG

 * 2100 * 2120 * 2140 * 2160 * 2180 * 2200 * 2220 * 2240
R-E_caballus : CCCATGGGATGGAGAGAAATGGGCTACATTTTCT-ACC-CTAAGAACAAGAACTTTAACCCGGACGAAAGTCTCCATGAAA--CTGGAGACTA-AAGGAGGATTTAGCAGTAAATTAAGAATAGA------GAGCTTAATTGAATCAGGCCATGAAGCGC
A-E_caballus : CCCATGGGATGGAGAGAAATGGGCTACATTTTCT-ACC-CTAAGAACAAGAACTTTAACCCGGACGAAAGTCTCCATGAAA--CTGGAGACTA-AAGGAGGATTTAGCAGTAAATTAAGAATAGA------GAGCTTAATTGAATCAGGCCATGAAGCGC
R-C_bactrianus : CCGATGGGCTGGGAAGAAATGGGCTACATTTTCTTGTC-TTAAGAAAACTTCAAAATCCTT--ACGAAAGCCCCTATGAAA--CTGAGGGCCC-AAGGAGGATTTAGTAGTAAATCAAGAATAGA------GTGCTTGATTGAACTAGGCCATGAAGCAC
A-C_bactrianus : CCGATGGGCTGGGAAGAAATGGGCTACATTTTCTTGTC-TTAAGAAAACTTCAAAATCCTT--ACGAAAGCCCCTATGAAA--CTGAGGGCCC-AAGGAGGATTTAGTAGTAAATCAAGAATAGA------GTGCTTGATTGAACTAGGCCATGAAGCAC
R-C_lupus_familiaris : CTTATGAGGTGGGAAGAAATGGGCTACATTTTCT-ACC-C-AAGAACAT---------TTC--ACGAATGTTTTTATGAAA--TTAAAAACTG-AAGGAGGATTTAGTAGTAAATTAAGAATAGA------GAGCTTAATTGAATAGGGCCATGAAGCAC
A-C_lupus_familiaris : CTTATGAGGTGGGAAGAAATGGGCTACATTTTCT-ACC-C-AAGAACAT---------TTC--ACGAATGTTTTTATGAAA--TTAAAAACTG-AAGGAGGATTTAGTAGTAAATTAAGAATAGA------GAGCTTAATTGAATAGGGCCATGAAGCAC
R-N_procyonoides : CTAATGAGATGGGAAGAAATGGGCTACATTTTCT-ACA-C-AAGAATA----------TAT--ACGAAAGTTTTTATGAAA--CTATAAACCG-AAGGAGGATTTAGTAGTAAACTAAGAATAGA------GAGCTTAGTTGAATAGGGCCATGAAGCAC
A-N_procyonoides : CTAATGAGATGGGAAGAAATGGGCTACATTTTCT-ACA-C-AAGAATA----------TAT--ACGAAAGTTTTTATGAAA--CTATAAACCG-AAGGAGGATTTAGTAGTAAACTAAGAATAGA------GAGCTTAGTTGAATAGGGCCATGAAGCAC
R-V_lagopus : CTAATGAGATGGGAAGAAATGGGCTACATTTTCT-GTT-TTAAGAACAC---------TTT--ACGAAAGTTTTTATGAAA--CTAAGAACTG-AAGGAGGATTTAGTAGTAAATTAAGAATAGA------GAGCTTAATTGAATAGGGCCATGAAGCAC
A-V_lagopus : CTAATGAGATGGGAAGAAATGGGCTACATTTTCT-GTT-TTAAGAACAC---------TTT--ACGAAAGTTTTTATGAAA--CTAAGAACTG-AAGGAGGATTTAGTAGTAAATTAAGAATAGA------GAGCTTAATTGAATAGGGCCATGAAGCAC
R-M_putorius : CCTATGAAGTGGGAAGAAATGGGCTACATTTTCT-AAC-C-AAGAACACA--------CTC--ACGAAAGTTTTTATGAAAA-CTAAAAACTA-AAGGTGGATTTAGTAGTAAATTAAGAATAGA------GAGCTTAATTGAATAGGGCCATAAAGCAC
A-M_putorius : CCTATGAAGTGGGAAGAAATGGGCTACATTTTCT-AAC-C-AAGAACACA--------CTC--ACGAAAGTTTTTATGAAAA-CTAAAAACTA-AAGGTGGATTTAGTAGTAAATTAAGAATAGA------GAGCTTAATTGAATAGGGCCATAAAGCAC
R-B_taurus : CCTATGAAATGGGAAGAAATGGGCTACATTCTCT-ACA-CCAAGAGAATCAA-------GC--ACGAAAGTTATTATGAAA--CCAATAACCA-AAGGAGGATTTAGCAGTAAACTAAGAATAGA------GTGCTTAGTTGAATTAGGCCATGAAGCAC
A-B_taurus : CCTATGAAATGGGAAGAAATGGGCTACATTCTCT-ACA-CCAAGAGAATCAA-------GC--ACGAAAGTTATTATGAAA--CCAATAACCA-AAGGAGGATTTAGCAGTAAACTAAGAATAGA------GTGCTTAGTTGAATTAGGCCATGAAGCAC
R-O_aries : CCTATGGAGTGGGAAGAAATGGGCTACATTTTCT-AC--CCAAGAAAATTTA-------AT--ACGAAAGCCATTATGAAA--TTAATAGCCA-AAGGAGGATTTAGCAGTAAACTAAGAATAGA------GTGCTTAGTTGAATCAGGCCATGAAGCAC
A-O_aries : CCTATGGAGTGGGAAGAAATGGGCTACATTTTCT-AC--CCAAGAAAATTTA-------AT--ACGAAAGCCATTATGAAA--TTAATAGCCA-AAGGAGGATTTAGCAGTAAACTAAGAATAGA------GTGCTTAGTTGAATCAGGCCATGAAGCAC
R-S_scrofa : CTTATGGGTTGGAAAGAAATGGGCTACATTTTCT-AC--ATAAGAATACCCAC-----CAT--ACGAAAGTTTTTATGAAA--CTAAAAACCA-AAGGAGGATTTAGCAGTAAATCAAGAATAGA------GTGCTTGATTGAATAAGGCCATGAAGCAC
A-S_scrofa : CTTATGGGTTGGAAAGAAATGGGCTACATTTTCT-AC--ATAAGAATACCCAC-----CAT--ACGAAAGTTTTTATGAAA--CTAAAAACCA-AAGGAGGATTTAGCAGTAAATCAAGAATAGA------GTGCTTGATTGAATAAGGCCATGAAGCAC
R-O_cuniculus : CCCATAGAGTGGAGAGCAATGGGCTACATTTTCT-AC--TTCAGAATAT--------------ACGAAAGCCCTTATGAAACTCTAAGGGCCA-AAGGAGGATTTAGTAGTAAATTAAGAATAGA------GTGCTTAATTGAACAAGGCCATGAAGCAC
A-O_cuniculus : CCCATAGAGTGGAGAGCAATGGGCTACATTTTCT-AC--TTCAGAATAT--------------ACGAAAGCCCTTATGAAACTCTAAGGGCCA-AAGGAGGATTTAGTAGTAAATTAAGAATAGA------GTGCTTAATTGAACAAGGCCATGAAGCAC
R-M_musculus : CCAATGAAATGGGAAGAAATGGGCTACATTTTCTTATA--AAAGAACATT-------------ACTATACCCTTTATGAAA--CTAAAGGACT-AAGGAGGATTTAGTAGTAAATTAAGAATAGA------GAGCTTAATTGAATTGAGCAATGAAGTAC
A-M_musculus : CCAATGAAATGGGAAGAAATGGGCTACATTTTCTTATA--AAAGAACATT-------------ACTATACCCTTTATGAAA--CTAAAGGACT-AAGGAGGATTTAGTAGTAAATTAAGAATAGA------GAGCTTAATTGAATTGAGCAATGAAGTAC
R-R_norvegicus : CCAATGAAGCGGAAAGAAATGGGCTACATTTTCTTTTCCCAGAGAACATT-------------ACGAAACCCTTTATGAAA--CTAAAGGACA-AAGGAGGATTTAGTAGTAAATTAAGAATAGA------GAGCTTAATTGAATAGAGCAATGAAGTAC
A-R_norvegicus : CCAATGAAGCGGAAAGAAATGGGCTACATTTTCTTTTCCCAGAGAACATT-------------ACGAAACCCTTTATGAAA--CTAAAGGACA-AAGGAGGATTTAGTAGTAAATTAAGAATAGA------GAGCTTAATTGAATAGAGCAATGAAGTAC
R-M_coypus : CCAATGAAGTGGAAAGAAATGGGCTACATTTTCTTTTT---AAGAACATCTA-----------ACAGTAATCCTTATGAAA--CTAAGGATTT-AAGGAGGATTTAGTAGTAAATTAAGAATAGA------GAGCTTAATTGAATAAGGCCATAAAGCAC
A-M_coypus : CCAATGAAGTGGAAAGAAATGGGCTACATTTTCTTTTT---AAGAACATCTA-----------ACAGTAATCCTTATGAAA--CTAAGGATTT-AAGGAGGATTTAGTAGTAAATTAAGAATAGA------GAGCTTAATTGAATAAGGCCATAAAGCAC
R-A_platyrhynchos : CCTATGGGACGGA-AGAAATGGGCTACATTCCCT-ATG-CATAGGGCAAC-------------ACGGAAAGAAGTATGAAA--CT-GCTTCTAGAAGGAGGATTTAGCAGTAAAGCGGGACAATA------AAGCTCGCTTTAAGCCGGCCCTAGGGCAC
A-A_platyrhynchos : CCTATGGGACGGA-AGAAATGGGCTACATTCCCT-ATG-CATAGGGCAAC-------------ACGGAAAGAAGTATGAAA--CT-GCTTCTAGAAGGAGGATTTAGCAGTAAAGCGGGACAATA------AAGCTCGCTTTAAGCCGGCCCTAGGGCAC
R-G_gallus : CCTATGGGGTGGG-AGAAATGGGCTACATTTTCT-A-A-CATAGAACAA--------------ACGAAAAAGGATGTGAAA--CCCGCCCTTAGAAGGAGGATTTAGCAGTAAAGTGAGATCATACCCCCTAAGCTCACTTTAAGACGGCTCTGAGGCAC
A-G_gallus : CCTATGGGGTGGG-AGAAATGGGCTACATTTTCT-A-A-CATAGAACAA--------------ACGAAAAAGGACGTGAAA--CCCGCCCTTAGAAGGAGGATTTAGCAGTAAAGTGAGATCATACCCCCTAAGCTCACTTTAAGACGGCTCTGAGGCAC
 CCTATGAGGTGGGAAGAAATGGGCTACATTTTCTTACCCCTAAGAACATYTACWWWWMCTTGGACGAAAGTCTTTATGAAAMTCTAAAAACCAGAAGGAGGATTTAGTAGTAAATTAAGAATAGACCCCCTGAGCTTAATTGAATAAGGCCATGAAGCAC

 * 2260 * 2280 * 2300 * 2320 * 2340 * 2360 * 2380 * 2400
R-E_caballus : GCACACACCGCCCGTCACCCTCCTTAAATATCACAAATC-ATA-----ACA-TAACATAAAACCGTGACCCAA---ACATATGAAAGGAGACAAGTCGTAACAAGGTAAGTATACCGGAAGGTGTACTTGGAT-AACCAAAGTGTAGCT-TAAA--CAAA
A-E_caballus : GCACACACCGCCCGTCACCCTCCTTAAATATCACAAATC-ACA-----ACA-TAACATAAAACCGTGACCCAA---ACATATGAAAGGAGACAAGTCGTAACAAGGTAAGTATACCGGAAGGTGTACTTGGAT-AACCAAAGTGTAGCT-TAAA--CAAA
R-C_bactrianus : GCACACACCGCCCGTCACCCTCTTCAAATCCAATGAGCCCGCA-----AGAA-AATATAAA--TAAGTGCAAA---ACGTATGAGAAGAGACAAGTCGTAACAAGGTAAGCATACTGGAAAGTGTGCTTGGATGAG-CAAAACGTAGCT-TAAA--AAAA
A-C_bactrianus : GCACACACCGCCCGTCACCCTCTTCAAATCCAATGAGCCCGCA-----AGAA-AATATAAA--TAAGTGCAAA---ACGTATGAGAAGAGACAAGTCGTAACAAGGTAAGCATACTGGAAAGTGTGCTTGGATGAG-CAAAACGTAGCT-TAAA--AAAA
R-C_lupus_familiaris : GCACACACCGCCCGTCACCCTCCTCAAGT--AATAAGAC-ACA-AC-CATAACCATATTAACTTAA-C-TAAA---AC--ACAAGAGGAGACAAGTCGTAACAAGGTAAGCATACCGGAAGGTGTGCTTGGATTAATCAAAGTGTAGCT-TAAC--TAAA
A-C_lupus_familiaris : GCACACACCGCCCGTCACCCTCCTCAAGT--AATAAGAC-ACA-AC-CATAACCATATTAACTTAA-C-TAAA---AC--ACAAGAGGAGACAAGTCGTAACAAGGTAAGCATACCGGAAGGTGTGCTTGGATTAATCAAAGTGTAGCT-TAAC--TAAA
R-N_procyonoides : GCACACACCGCCCGTCACCCTCCTCAAGT--GACAAAACCAAA-GC-CACAACCATATTAAC-CAAAC-CAAA---AC--ACAAGAGGAGACAAGTCGTAACAAGGTAAGCATACCGGAAGGTGTGCTTGGACTAACCAAAGTGTAGCT-TAAC--AAAA
A-N_procyonoides : GCACACACCGCCCGTCACCCTCCTCAAGT--GACAAAACCAAA-GC-CACAACCATATTAAC-CAAAC-CAAA---AC--ACAAGAGGAGACAAGTCGTAACAAGGTAAGCATACCGGAAGGTGTGCTTGGACTAACCAAAGTGTAGCT-TAAC--AAAA
R-V_lagopus : GCACACACCGCCCGTCACCCTCCTCAAGT--AATAAGACTGAG-AC-CACAATCATATTAACTCAAGT-CAAA---AC--ACGAGAGGAGATAAGTCGTAACAAGGTAAGCATACCGGAAGGTGTGCTTGGATTAACCAAAGTGTAGCT-TAAT--AAAA
A-V_lagopus : GCACACACCGCCCGTCACCCTCCTCAAGT--AATAAGACTGAG-AC-CACAATCATATTAACTCAAGT-CAAA---AC--ACGAGAGGAGATAAGTCGTAACAAGGTAAGCATACCGGAAGGTGTGCTTGGATTAACCAAAGTGTAGCT-TAAT--AAAA
R-M_putorius : GCACACACCGCCCGTCACCCTCCTCAAGC--AACACACTCAAATAC-TACA-TAATA--AAAGTAAAC-CTAA---A---GCAAGAGGAGACAAGTCGTAACAAGGTAAGCATACTGGAAAGTGTGCTTGGGTAAATCAAAGTGTAGCT-TAAC--TAAA
A-M_putorius : GCACACACCGCCCGTCACCCTCCTCAAGC--AACACACTCAAATAC-TACA-TAATA--AAAGTAAAC-CTAA---A---GCAAGAGGAGACAAGTCGTAACAAGGTAAGCATACTGGAAAGTGTGCTTGGGTAAATCAAAGTGTAGCT-TAAC--TAAA
R-B_taurus : GCACACACCGCCCGTCACCCTCCTCAAAT---AGATT-CAGTGCAT-CTAACCC-TATTTAAACGCAC--TAG---CTACATGAGAGGAGACAAGTCGTAACAAGGTAAGCATACTGGAAAGTGTGCTTGGATAAATCAAGATATAGCT-TAAA--CAAA
A-B_taurus : GCACACACCGCCCGTCACCCTCCTCAAAT---AGATT-CAGTGCAT-CTAACCC-TATTTAAACGCAC--TAG---CTACATGAGAGGAGACAAGTCGTAACAAGGTAAGCATACTGGAAAGTGTGCTTGGATAAATCAAGATATAGCT-TAAA--CAAA
R-O_aries : GCACACACCGCCCGTCACCCTCCTCAAGT---AAATA-TGATATAC-TTAAACC-TATTTACATATAT--CAA---CCACACGAGAGGAGACAAGTCGTAACAAGGTAAGCATACTGGAAAGTGTGCTTGGATAAACCAAGATATAGCT-TAAT--TAAA
A-O_aries : GCACACACCGCCCGTCACCCTCCTCAAGT---AAATA-TGATATAC-TTAAACC-TATTTACATATAT--CAA---CCACACGAGAGGAGACAAGTCGTAACAAGGTAAGCATACTGGAAAGTGTGCTTGGATAAACCAAGATATAGCT-TAAT--TAAA
R-S_scrofa : GCACACACCGCCCGTCACCCTCCTCAAGC---ATGTAGTAATAAAA-ATAACCTATATTCAATTACA---CAA---CCATGCAAGAAGAGACAAGTCGTAACAAGGTAAGCATACTGGAAAGTGTGCTTGGATTA-CCAAAGCATAGCT-TAAAC-TAAA
A-S_scrofa : GCACACACCGCCCGTCACCCTCCTCAAGC---ATGTAGTAATAAAA-ATAACCTATATTCAATTACA---CAA---CCATGCAAGAAGAGACAAGTCGTAACAAGGTAAGCATACTGGAAAGTGTGCTTGGATTA-CCAAAGCATAGCT-TAAAC-TAAA
R-O_cuniculus : GCACACACCGCCCGTCACCCTCCTCAAGTGACAAATATTTACTTATACCTAATTACATAAA--TAGAC---AA---GC--ATAAGAGGAGATAAGTCGTAACAAGGTAAGCATACTGGAAAGTGTGCTTGGACATTTCAGAGCGTAGCT-TAACT-TAAA
A-O_cuniculus : GCACACACCGCCCGTCACCCTCCTCAAGTGACAAATATTTACTTATACCTAATTACATAAA--TAGAC---AA---GC--ATAAGAGGAGATAAGTCGTAACAAGGTAAGCATACTGGAAAGTGTGCTTGGACACTTCAGAGCGTAGCT-TAACT-TAAA
R-M_musculus : GCACACACCGCCCGTCACCCTCCTCAAAT----TAAATTAA-A----CTTA-ACATAATTAATTTC-TAGACATCCGTTTATGAGAGGAGATAAGTCGTAACAAGGTAAGCATACTGGAAAGTGTGCTTGGAATAATCATAGTGTAGCT-TAATATTAAA
A-M_musculus : GCACACACCGCCCGTCACCCTCCTCAAAT----TAAATTAA-A----CTTA-ACATAATTAATTTC-TAGACATCCGTTTATGAGAGGAGATAAGTCGTAACAAGGTAAGCATACTGGAAAGTGTGCTTGGAATAATCATAGTGTAGCT-TAATATTAAA
R-R_norvegicus : GCACACACCGCCCGTCACCCTCCTCAAAT----TAGATTGGCA----TTCATATATACATAATTTCACTAACA--AATTTATGAGAGGAGATAAGTCGTAACAAGGTAAGCATACTGGAAAGTGTGCTTGGAATAATCACAGTGTAGCT-TAATCACAAA
A-R_norvegicus : GCACACACCGCCCGTCACCCTCCTCAAAT----TAGATTGGCA----TTCATATATACATAATTTCACTAACA--AATTTATGAGAGGAGATAAGTCGTAACAAGGTAAGCATACTGGAAAGTGTGCTTGGAATAATCACAGTGTAGCT-TAATCACAAA
R-M_coypus : GCACACACCGCCCGTCACCCTCCTCAAAT-------ATTAA------CTCAAGCTTATCAAAGCT-----ACA---AGATATAAGAGGAGATAAGTCGTAACAAGGTAAACATACTGGAAAGTGTGCTTGGAATAA-CAAAGCATAGCT-TAAC--TAAA
A-M_coypus : GCACACACCGCCCGTCACCCTCCTCAAAT-------ATTAA------CTCAAGCTTATCAAAGCT-----ACA---AGATATAAGAGGAGATAAGTCGTAACAAGGTAAGCATACTGGAAAGTGTGCTTGGAATAA-CAAAGCATAGCT-TAAC--TAAA
R-A_platyrhynchos : GTACATACCGCCCGTCACCCTCCTCA-------TAAGCCA-CACCCCCACATAATTA-ATAC--CACGTAAAT---GC-CA-AAGATGAGGTAAGTCGTAACAAGGTAAGTGTACCGGAAGGTGTACTTAGAATACTCAAGACGTAGCTATAACCCCAAA
A-A_platyrhynchos : GTACATACCGCCCGTCACCCTCCTCA-------TAAGCCA-CACCCCCACATAATTA-ATAC--CACGTAAAT---GC-CA-AAGATGAGGTAAGTCGTAACAAGGTAAGTGTACCGGAAGGTGTACTTAGAATACTCAAGACGTAGCTATAACCCCAAA
R-G_gallus : GTACATACCGCCCGTCACCCTCTTCA-------CAAGCCATCAACATCA-ATAAATATATACTTCCCCTCCCG---GC-TA-AAGACGAGGCAAGTCGTAACAAGGTAAGTGTACCGGAAGGTGCACTTAGACTAC-CAAGGCGTAGCTATAACTTCAAA
A-G_gallus : GTACATACCGCCCGTCACCCTCTTCA-------CAAGCCATCAACATCA-ATAAATATATACTTCCCCTCCCG---GC-TA-AAGACGAGGCAAGTCGTAACAAGGTAAGTGTACCGGAAGGTGCACTTAGACTAC-CAAGGCGTAGCTATAACTTCAAA
 GCACACACCGCCCGTCACCCTCCTCAAGTVHAATAAATCAACATACHCACAACCATATTAAATTAAACTCAAATCMACATATAAGAGGAGACAAGTCGTAACAAGGTAAGCATACTGGAAAGTGTGCTTGGATTAATCAAAGTGTAGCTATAACCTTAAA

 * 2420 * 2440 * 2460 * 2480 * 2500 * 2520 * 2540 * 2560
R-E_caballus : GCATCCAGCTTACACCTAGAAGATTTCACTCAA-AAT--GAACACTTTGA-----ACTAAAGCTAGCCCAAACAATACCTAATT------CAATTACCC---TTAGTCAC-TT------AACTAAAACATTCA-CCAAACCAT-TAAAGTATAGGAGATA
A-E_caballus : GCATCCAGCTTACACCTAGAAGATTTCACTCAA-AAT--GAACACTTTGA-----ACTAAAGCTAGCCCAAACAATACCTAATT------CAATTACCC---TTAGTCAC-TT------AACTAAAACATTCA-CCAAACCAT-TAAAGTATAGGAGATA
R-C_bactrianus : GTACCTAATTTACACTTAGGAGATTTCGTGGA--AAT--GAACGTTTTGA-----ACTAGAGCTAGCCCAGAAAATACCACATTT-----CAACTATT----TCAAAACCGTT------AAACAAAACATTTACTCAC-TCTTCTAAAGTATAGGAGATA
A-C_bactrianus : GTACCTAATTTACACTTAGGAGATTTCGTGGA--AAT--GAACGTTTTGA-----ACTAGAGCTAGCCCAGAAAATACCACATTT-----CAACTATT----TCAAAACCGTT------AAACAAAACATTTACTCAC-TCTTCTAAAGTATAGGAGATA
R-C_lupus_familiaris : GCGTCTGGCCTACACCCAGAAGATTTCATT-ACTTAT--GGCCACTTTGA-----ACAAAAGCTAGCCCAACTAACCCCAAACT------TAAGTATT----ACAGACACATA------AAATAAAACATTTAGTTAAACAAT-AAAAGTATAGGAGATA
A-C_lupus_familiaris : GCGTCTGGCCTACACCCAGAAGATTTCATT-ACTTAT--GGCCACTTTGA-----ACAAAAGCTAGCCCAACTAACCCCAAACT------TAAGTATT----ACAGACACATA------AAATAAAACATTTAGTTAAACAAT-AAAAGTATAGGAGATA
R-N_procyonoides : GCATCTGGCTTACACCCAGAAGATTTCATA-CTCCAT--GACCACTTTGA-----ACCAAAGCTAGCCCATACAAACCCAAACC------AAAATACC----ACAGATACATA------AAACAAAACATTCAGTTAAGTCAT-AAAAGTATAGGAGATA
A-N_procyonoides : GCATCTGGCTTACACCCAGAAGATTTCATA-CTCCAT--GACCACTTTGA-----ACCAAAGCTAGCCCATACAAACCCAAACC------AAAATACC----ACAGATACATA------AAACAAAACATTCAGTTAAGTCAT-AAAAGTATAGGAGATA
R-V_lagopus : GCATCTGGCTTACACCCAGAAGATTTCATG-ACTAAT--GACCACTTTGA-----ACGAAAGCTAGCCCAATCAGCTTTAAATT------AAACTGTT----ATGTAATCGCA------AAATAAAACATTTAGTTAAAATATTAAAAGTATAGGAGATA
A-V_lagopus : GCATCTGGCTTACACCCAGAAGATTTCATG-ACTAAT--GACCACTTTGA-----ACGAAAGCTAGCCCAATCAGCTTTAAATT------AAACTGTT----ATGTAATCGCA------AAATAAAACATTTAGTTAAAATATTAAAAGTATAGGAGATA
R-M_putorius : GCATCTGGCTTACACCCAGAAGATTTCATATATTAAT--GACCACTTTGA-----ACCAATACTAGCCCAACTTATCACTAACT------CAATTATC----ACAGTCACATA------AATCAAAACATTTAATCACATTAT-TACAGTATAGGAGATA
A-M_putorius : GCATCTGGCTTACACCCAGAAGATTTCATATATTAAT--GACCACTTTGA-----ACCAATACTAGCCCAACTTATCACTAACT------CAATTATC----ACAGTCACATA------AATCAAAACATTTAATCACATTAT-TACAGTATAGGAGATA
R-B_taurus : GCATCCAGTTTACACCTAGAAGACTTCATTCAT-TAT--GAATATCTTGA-----ACTAGACCTAGCCCAAAGATACCCTCTC----GACTAAACAACC---AAGATAGAATA------AAACAAAACATTTAATCCCAA-TT-TAAAGTATAGGAGATA
A-B_taurus : GCATCCAGTTTACACCTAGAAGACTTCATTCAT-TAT--GAATATCTTGA-----ACTAGACCTAGCCCAAAGATACCCTCTC----GACTAAACAACC---AAGATAGAATA------AAACAAAACATTTAATCCCAA-TT-TAAAGTATAGGAGATA
R-O_aries : GCATCTAGTTTACACCTAGAAGATTTCACACAT-TAT--GAGTATCTTGA-----ACTATACCTAGCCCAAAATCTCCCACTCTCCAGTTTAAATAACT---AA-ATTAATTA------AAATAAAACATTTA--CCCTA-AT-TAAAGTATAGGAGATA
A-O_aries : GCATCTAGTTTACACCTAGAAGATTTCACACAT-TAT--GAGTATCTTGA-----ACTATTCCTAGCCCAAAACCTCCCACTCTCCAGTTTAAATAACT---AA-ATTAATTA------AAATAAAACATTTA--CCCTA-AT-TAAAGTATAGGAGATA
R-S_scrofa : GCACCTAGTTTACACCTAGAAGATCCCATAATA-TAT--GGGTACTTTGA-----ACCAAAGCTAGCTCAACATATTAAAC------AAATACAAAAAT---ACACCAAAATA------AAATAAAACATTCA--CCTAACAT-TAAAGTATAGGAGATA
A-S_scrofa : GCACCTAGTTTACACCTAGAAGATCCCATAATA-TAT--GGGTACTTTGA-----ACCAAAGCTAGCTCAACATATTAAAC------AAATACAAAAAT---ACACCAAAATA------AAATAAAACATTCA--CCTAACAT-TAAAGTATAGGAGATA
R-O_cuniculus : GCACTTGGCTTACACCCAAGAGATTTCATTTAC---T--GACCACTCTGA-----GCCAACACTAGCCCTATGAAACTAACACT------GAACCAGA----GTAATTAGCTA------AACTAAACCATTCA--CCCCATATTAAAAGTATAGGAGATA
A-O_cuniculus : GCACTTGGCTTACACCCAAGAGATTTCATTTAC---T--GACCACTCTGA-----GCCAACACTAGCCCTATGAAACTAACACT------GAACCAGA----GTAATTAGCTA------AACTAAACCATTCA--CCCCATATTAAAAGTATAGGAGATA
R-M_musculus : GCATCTGGCCTACACCCAGAAGATTTCATGACC-AAT--GAACACTCTGA-----ACTAATCCTAGCCCTAGC---CCTACACA--AATATAATTATACTATTAT-----ATA------AATCAAAACATTTA----TCCTACTAAAAGTATTGGAGAAA
A-M_musculus : GCATCTGGCCTACACCCAGAAGATTTCATGACC-AAT--GAACACTCTGA-----ACTAATCCTAGCCCTAGC---CCTACACA--AATATAATTATACTATTAT-----ATA------AATCAAAACATTTA----TCCTACTAAAAGTATTGGAGAAA
R-R_norvegicus : GCATCTGGCCTACACCCAGAAGAATTCATAAA--AAT--GAACACTTTGA-----ACTAATCCTAGCCCTACA---ACCA-ACC--AACATAACTAAACCCCCCAC----ATA------AACTAAAACATTTA----ACTCA--AAAAGTATTGGAGAAA
A-R_norvegicus : GCATCTGGCCTACACCCAGAAGAATTCATAAA--AAT--GAACACTTTGA-----ACTAACCCTAGCCCTACA---ACCA-ACC--AACATAACTAACCCCCCCCCCCCCATA------AACTAAAACATTTA----ACTCA--AAAAGTATTGGAGAAA
R-M_coypus : GCATCCGGCTTACACCCGGAAGATATCAATTTC-ATT--GACTGCTTTGA-----ACTAATACTAGCCTAATA---TTCACATAT-AACTCAAGTACTATATATATATATATAT--TACAAACAAAACATTTA----CATCT--AAAAGTATAGGTGATA
A-M_coypus : GCATCCGGCTTACACCCGGAAGATATCAATTTC-ATT--GACTGCTTTGA-----ACTAATACTAGCCTAATA---TTCACATAT-AACTCAAGTACTATATATATATATATAT--TACAAACAAAACATTTA----CATCT--AAAAGTATAGGTGATA
R-A_platyrhynchos : GCACTCAGCTTACACCTGAGAGATATCTGC--TAAACCAGGTCGTCTTGA---AGCCTTCCTCTAGCTCAGCCG--CTTAAACAACGCAAAACTAAAGAATC-CCACTAATTAAGACTTAACTAAAGCATTTTCTA--GTCCT----AGTATAGGCGATA
A-A_platyrhynchos : GCACTCAGCTTACACCTGAGAGATATCTGC--TAAACCAGGTCGTCTTGA---AGCCTTCCTCTAGCTCAGCCG--CTTAAACAACGCAAAACTAAAGAATC-CCACTAATTAAGACTTAACTAAAGCATTTTCTA--GTCCT----AGTATAGGCGATA
R-G_gallus : GCATTCAGCTTACACCTGAAAGATACCCTCAACAGACAAGGTCGCCTTGACTTGCCCCCCCTCTAGCCCGACAAA-CTCGTACC-CTTAACATAAAAAACTTACCTCCCCCT----CTTAACCAAAACATTATAAATTGTCCC----AGTATAGGCGATA
A-G_gallus : GCATTCAGCTTACACCTGAAAGATACCCTCAACAGACAAGGTCGCCTTGACTTGCCCCCCCTCTAGCCCGACAAA-CTCGTACC-CTTAACATAAAAAACTTACCTCCCCCT----CTTAACCAAAACATTATAAATTGTCCC----AGTATAGGCGATA
 GCATCTGGCTTACACCCAGAAGATTTCATWAACTAATMAGACCACTTTGACTTRSACTAAAGCTAGCCCAACAAATCCCACACTTCAAAATAAATAACCYWTACAAWTACATAWGACTTAAATAAAACATTTARTCACATCATTAAAAGTATAGGAGATA

 * 2580 * 2600 * 2620 * 2640 * 2660 * 2680 * 2700 * 2720
R-E_caballus : GAAATTTT-----AACT-TGGCGCTATAGAGA-AA---GTACCGTAAGGGAACGATGAAAGAT------GCATTAAAAGTACTAAACAGCAAAGCTTACCCCTTTTACCTTTTGCATAATGATTTAACTAG-AATAAACTTAGCAAAGAGAACTTAAGCT
A-E_caballus : GAAATTTT-----AACT-TGGCGCTATAGAGA-AA---GTACCGTAAGGGAACGATGAAAGAT------GCATTAAAAGTACTAAACAGCAAAGCTTACCCCTTTTACCTTTTGCATAATGATTTAACTAG-AATAAACTTAGCAAAGAGAACTTAAGCT
R-C_bactrianus : GAAATTTA-----CTAC-TGGCGCTATAGAGA-GA---GTACCGTAAGGGAACGATGAAAGAA------TACCTAAAAGTGATAAAAAGCAAAGATTAACCCTTGTACCTTTTGCATAATGATTTAACTAG-AAA-ATTTTAGCAAAGAGAACTTAAGTT
A-C_bactrianus : GAAATTTA-----CTAC-TGGCGCTATAGAGA-GA---GTACCGTAAGGGAACGATGAAAGAA------TACCTAAAAGTGATAAAAAGCAAAGATTAACCCTTGTACCTTTTGCATAATGATTTAACTAG-AAA-ATTTTAGCAAAGAGAACTTAAGTT
R-C_lupus_familiaris : GAAATTT------TAAT-TGGAGCGATAGAGA-TA---GTACCGTAAGGGAATGATGAAAGAC------ATCTTAACAGTATTAAACAGCAAAGATTACCCCTTCTACCTTTTGCATAATGAACTAGCCAG-AAACAACTTAACAAAGAGAACTTAAGCT
A-C_lupus_familiaris : GAAATTT------TAAT-TGGAGCGATAGAGA-TA---GTACCGTAAGGGAATGATGAAAGAC------ATCTTAACAGTATTAAACAGCAAAGATTACCCCTTCTACCTTTTGCATAATGAACTAGCCAG-AAACAACTTAACAAAGAGAACTTAAGCT
R-N_procyonoides : GAAATTA------TAAT-TGGAGCTATAGAGA-TA---GTACCGTAAGGGAATGATGAAAGAC------ATTTTAAAAGTACAGAATAGCAAAGACTACCCCTTTTACCTTTTGCATAATGAACTAGCCAG-AAATAACTTAACAAAGAGAACTTAAGCT
A-N_procyonoides : GAAATTA------TAAT-TGGAGCTATAGAGA-TA---GTACCGTAAGGGAATGATGAAAGAC------ATTTTAAAAGTACAGAATAGCAAAGACTACCCCTTTTACCTTTTGCATAATGAACTAGCCAG-AAATAACTTAACAAAGAGAACTTAAGCT
R-V_lagopus : GAAATTC------TAAT-TGGAGCTATAGAGA-CA---GTACCGTAAGGGAATGATGAAAGAC------ATTTTCAAAGTACAAAACAGCAAAGATTATACCTTCTACCTTTTGCATAATGAACTAGCCAG-AAATAACTTAACAAAGAGAACTTAAGCT
A-V_lagopus : GAAATTC------TAAT-TGGAGCTATAGAGA-CA---GTACCGTAAGGGAATGATGAAAGAC------ATTTTCAAAGTACAAAACAGCAAAGATTATACCTTCTACCTTTTGCATAATGAACTAGCCAG-AAATAACTTAACAAAGAGAACTTAAGCT
R-M_putorius : GAAATTC------TACT-TGGAGCTATAGAGA-AA---GTACCGCAAGGGAACGATGAAAGAA------AAATTCAAAGTAATAAACAGCAAAGATTACACCTTATACCTTTTGCATAATGAGCTAGCTAG-AA-TAATTCAGCAAAGAGATCTTAAGCT
A-M_putorius : GAAATTC------TACT-TGGAGCTATAGAGA-AA---GTACCGCAAGGGAACGATGAAAGAA------AAATTCAAAGTAATAAACAGCAAAGATTACACCTTATACCTTTTGCATAATGAGCTAGCTAG-AA-TAATTCAGCAAAGAGATCTTAAGCT
R-B_taurus : GAAAT-CT-----AAGTACGGCGCTATAGAGA-AA---GTACCGCAAGGGAACGATGAAAGAAAA----AAACTAAAAGTATAAAAAAGCAAAGATTACCCCTTGTACCTTTTGCATAATGAATTAACTAGTATAAGACTTAACAAAATGAATTTTAGCT
A-B_taurus : GAAAT-CT-----AAGTACGGCGCTATAGAGA-AA---GTACCGCAAGGGAACGATGAAAGAAAA----AAACTAAAAGTATAAAAAAGCAAAGATTACCCCTTGTACCTTTTGCATAATGAATTAACTAGTATAAGACTTAACAAAATGAATTTTAGCT
R-O_aries : GAAATTCT-----AAACACGGCGCTATAGAGA-AA---GTACCGCAAGGGAATGATGAAAGAAAA----AAATCATA-GTACAAAAAAGCAAAGATTAACCCTTGTACCTTTTGCATAATGAATTAACGAGCAAAAAACTTAACAAAACGAATTTTAGCT
A-O_aries : GAAATTCT-----AAACACGGCGCTATAGAGA-AA---GTACCGCAAGGGAATGATGAAAGAAAA----AAATCATA-GTACAAAAAAGCAAAGATTAACCCTTGTACCTTTTGCATAATGAATTAACGAGCAAAAAACTTAACAAAACGAATTTTAGCT
R-S_scrofa : GAAATTTT-----TATCCTGACGCTATAGAGA-TA---GTACCGTAAGGGAAAGATGAAAGAATA----AAATAAAA-GTAAAAAAAAGCAAAGATTACCCCTTCTACCTTTTGCATAATGGTTTAACCAG-AAAAAATCTAACAAAGAGAACTTTAGCT
A-S_scrofa : GAAATTTT-----TATCCTGACGCTATAGAGA-TA---GTACCGTAAGGGAAAGATGAAAGAATA----AAATAAAA-GTAAAAAAAAGCAAAGATTACCCCTTCTACCTTTTGCATAATGGTTTAACCAG-AAAAAATCTAACAAAGAGAACTTTAGCT
R-O_cuniculus : GAAATTTA-----TATCCAGGCGCTATAGAGA-AA---GTACCGTAAGGGAAAGATGAAAGAT------TAGTTAATAGCACAAAAAAGCAGAGATTACTTCTCTTACCTTTTGCATAATGAATTAGCTAG-AAAATCCTTAGCAAAAAGAATTTTAGTT
A-O_cuniculus : GAAATTTA-----TATCCAGGCGCTATAGAGA-AA---GTACCGTAAGGGAAAGATGAAAGAT------TAGTTAATAGCACAAAAAAGCAGAGATTACTTCTCTTACCTTTTGCATAATGAATTAGCTAG-AAAATCCTTAGCAAAAAGAATTTTAGTT
R-M_musculus : GAAATTCG---TACATCTAGGAGCTATAGAAC-TA---GTACCGCAAGGGAAAGATGAAAGAC------TAATTAAAAGTAAGAACAAGCAAAGATTAAACCTTGTACCTTTTGCATAATGAACTAACTAG-AAAACTTCTAACTAAAAGAATTACAGCT
A-M_musculus : GAAATTCG---TACATCTAGGAGCTATAGAAC-TA---GTACCGCAAGGGAAAGATGAAAGAC------TAATTAAAAGTAAGAACAAGCAAAGATTAAACCTTGTACCTTTTGCATAATGAACTAACTAG-AAAACTTCTAACTAAAAGAATTACAGCT
R-R_norvegicus : GAAATTTA---CTTACC-AGGAGCTATAGAGA-AA---GTACCGCAAGGGAATGGTGAAAGAC------TAATTTAAAGTAAAAATAAGCAAAGATTAAACCTTGTACCTTTTGCATAATGAATTAACTAG-AAAATCCTTAACAAAAAGAATTTAAGCT
A-R_norvegicus : GAAATTTA---CTTACC-AGGAGCTATAGAGA-AA---GTACCGCAAGGGAATGGTGAAAGAC------TAATTTAAAGTAAAAATAAGCAAAGATTAAACCTTGTACCTTTTGCATAATGAATTAACTAG-AAAATCCTTAACAAAAAGAATTTAAGCT
R-M_coypus : GAAATTTAAAATTTACATAGC-GCTATAGAGA-TA---GTACCGTAAGGGAAAGATGAAAGAA------GAAT-AAAAGTACAAACAAGCATAGATTACACCTAGTACCTTTTGCATAATGAGTTAACTAG-AATAAACTTGACAAAAAGAATTTAAGTC
A-M_coypus : GAAATTTAAAATTTACATAGC-GCTATAGAGA-TA---GTACCGTAAGGGAAAGATGAAAGAA------GAAT-AAAAGTACAAACAAGCATAGATTACACCTAGTACCTTTTGCATAATGAGTTAACTAG-AATAAACTTGACAAAAAGAATTTAAGTC
R-A_platyrhynchos : GAAAAGAC------ACTTAGACGCGATAGAGACCA---GTACCGTAAGGGAAAGATGAAATAATAGTGAAAACTA-AAGCAAGAGACAGCAAAGACTAACCCTTGTACCTTTTGCATCATGATTTAGCAAG--AACAACCAAGCAAAGTGAACTGAAGTT
A-A_platyrhynchos : GAAAAGAC------ACTTAGACGCGATAGAGACCA---GTACCGTAAGGGAAAGATGAAATAATAGTGAAAACTA-AAGCAAGAGACAGCAAAGACTAACCCTTGTACCTTTTGCATCATGATTTAGCAAG--AACAACCAAGCAAAGTGAACTGAAGTT
R-G_gallus : GAAAAGAC-----TACCCCGGCGCAATAGAGGCTAACTGTACCGCAAGGGAAAGATGAAATAGCAATGAAAACCATAAGCAAAAAACAGCAAAGACCAACCCTTGTACCTTTTGCATCATGATTTAGCAAG--AACAACCAAGCAAAGTGAGCTAAAGTT
A-G_gallus : GAAAAGAC-----TACCCCGGCGCAATAGAGGCTAACTGTACCGCAAGGGAAAGATGAAATAGCAATGAAAACCATAAGCAAAAAACAGCAAAGACCAACCCTTGTACCTTTTGCATCATGATTTAGCAAG--AACAACCAAGCAAAGTGAGCTAAAGTT
 GAAATTTWAAATTTACYYTGGCGCTATAGAGACWAACTGTACCGTAAGGGAAAGATGAAAGAAWARTGAAAATTAAAAGTAAAAAAAAGCAAAGATTACCCCTTGTACCTTTTGCATAATGAATTAACTAGYAAAAAACTTAACAAAGAGAACTTAAGCT

 * 2740 * 2760 * 2780 * 2800 * 2820 * 2840 * 2860 * 2880
R-E_caballus : AAGCACCCCGAAACC-AGACGAGCTACCTATGAACAGTTACAAAT-GAACCAACTCATCTATGTCGCAAAATAGTGAGAAGATTCGTAGGTAGAGGTGAAAAGCCCAACGAGCCTGGTGATAGCTGGTTGTCCAGAAACAGAATTTCAGTTCAAATTTAA
A-E_caballus : AAGCACCCCGAAACC-AGACGAGCTACCTATGGACAGTTACAAA--GAACCAACTCATCTATGTCGCAAAATAGTGAGAAGATTCGTAGGTAGAGGTGAAAAGCCCAACGAGCCTGGTGATAGCTGGTTGTCCAGAAACAGAATTTCAGTTCAAATTTAA
R-C_bactrianus : AAATACCCCGAAACC-AGACGAGCTACTTGCGAACAGCC-TAC-G-GAGCGAACTCGTCTATGTGGCAAAATAGTGAGAAGATTTGCAAGTAGAGGTGACAAGCCTAACGAGCCTGGTGATAGCTGGTTGTCCAGGAAATGAATATAAGTTCAACTTTAA
A-C_bactrianus : AAATACCCCGAAACC-AGACGAGCTACTTGCGAACAGCC-TAC-G-GAGCGAACTCGTCTATGTGGCAAAATAGTGAGAAGATTTGCAAGTAGAGGTGACAAGCCTAACGAGCCTGGTGATAGCTGGTTGTCCAGGAAATGAATATAAGTTCAACTTTAA
R-C_lupus_familiaris : AAGCTCCCCGAAACC-AGACGAGCTACCCATAAACAATC-TAAAA-GGATCAACTCATCTATGTAGCAAAATAGTGAGAAGATTTGTGGGTAGAGGTGAAAAGCCTAACGAGCCTGGTGATAGCTGGTTACCCACAGACAGAATTTTAGTTCAACTTTAA
A-C_lupus_familiaris : AAGCTCCCCGAAACC-AGACGAGCTACCCATAAACAATC-TAAAA-GGATCAACTCATCTATGTAGCAAAATAGTGAGAAGATTTGTGGGTAGAGGTGAAAAGCCTAACGAGCCTGGTGATAGCTGGTTACCCACAGACAGAATTTTAGTTCAACTTTAA
R-N_procyonoides : AAGCCCCCCGAAACC-AGACGAGCTACCTATGAACAATC-TAGAA-GGATCAACTCATCTATGTGGCAAAATAGTGAGAAGATTTATAGGTAGAGGTGAAAAGCCTAACGAGCCTGGTGATAGCTGGTTACCCACAGACAGAATTTTAGTTCGACTTTAA
A-N_procyonoides : AAGCCCCCCGAAACC-AGACGAGCTACCTATGAACAATC-TAGAA-GGATCAACTCATCTATGTGGCAAAATAGTGAGAAGATTTATAGGTAGAGGTGAAAAGCCTAACGAGCCTGGTGATAGCTGGTTACCCACAGACAGAATTTTAGTTCGACTTTAA
R-V_lagopus : AAGTCCCCCGAAACC-AGACGAGCTACCTATGAACAATC-TAAAA-GGATCAACTCATCTATGTGGCAAAATAGTGAGAAGATTTGTAGGTAGAGGTGAAAAGCCTAACGAGCCTGGTGATAGCTGGTTACCCACAAATAGAATTTTAGTTCAACTTTAA
A-V_lagopus : AAGTCCCCCGAAACC-AGACGAGCTACCTATGAACAATC-TAAAA-GGATCAACTCATCTATGTGGCAAAATAGTGAGAAGATTTGTAGGTAGAGGTGAAAAGCCTAACGAGCCTGGTGATAGCTGGTTACCCACAAATAGAATTTTAGTTCAACTTTAA
R-M_putorius : AAATCCCCCGAAACC-AGACGAGCTACCTACGAACAATC-CACAG-GGATACACTCATCTATGTCGCAAAATAGTGAGAAGATTCATAGGTAGAGGTGAAAAGCCTAACGAGCCTGGTGATAGCTGGTTGCCCA-GAACAGAATCTCAGTTCAACTTTAA
A-M_putorius : AAATCCCCCGAAACC-AGACGAGCTACCTACGAACAATC-CACAG-GGATACACTCATCTATGTCGCAAAATAGTGAGAAGATTCATAGGTAGAGGTGAAAAGCCTAACGAGCCTGGTGATAGCTGGTTGCCCA-GAACAGAATCTCAGTTCAACTTTAA
R-B_taurus : AAGCAGCCCGAAACC-AGACGAGCTACTCACAAACAGTT-TACCAAGAACTAACTCATCTATGTGGCAAAATAGTGAGAAGATTTGTAAGTAGAGGTGACATGCCTAACGAGCCTGGTGATAGCTGGTTGTCCAGAAAATGAATCTAAGTTCAGCTTTAA
A-B_taurus : AAGCAGCCCGAAACC-AGACGAGCTACTCACAAACAGTT-TACCAAGAACTAACTCATCTATGTGGCAAAATAGTGAGAAGATTTGTAAGTAGAGGTGACATGCCTAACGAGCCTGGTGATAGCTGGTTGTCCAGAAAATGAATCTAAGTTCAGCTTTAA
R-O_aries : AAGTAACCCGAAACC-AGACGAGCTACTTATAGACAGTT-TATTA-GAACCAACTCATCTATGTGGCAAAATAGTGAGAAGATCCATAAGTAGAGGTGACATGCCTAACGAGCCTGGTGATAGCTGGTTGTCCAGAAAATGAATTTTAGTTCAGCTTTAA
A-O_aries : AAGTAACCCGAAACC-AGACGAGCTACTTATAGACAGTT-TATTA-GAACCAACTCATCTATGTGGCAAAATAGTGAGAAGATCCATAAGTAGAGGTGACATGCCTAACGAGCCTGGTGATAGCTGGTTGTCCAGAAAATGAATTTTAGTTCAGCTTTAA
R-S_scrofa : AGATACCCCGAAACC-AGACGAGCTACCTATGAGCAGTT-TAAAA-GAACCAACTCATCTATGTGGCAAAATAGTGAGAAGACTTGTAGGTAGAGGTGAAAAGCCTAACGAGCCTGGTGATAGCTGGTTGTCC-GAGAAAGAATTTTAGTTCAACCTTAA
A-S_scrofa : AGATACCCCGAAACC-AGACGAGCTACCTATGAGCAGTT-TAAAA-GAACCAACTCATCTATGTGGCAAAATAGTGAGAAGACTTGTAGGTAGAGGTGAAAAGCCTAACGAGCCTGGTGATAGCTGGTTGTCC-GAGAAAGAATTTTAGTTCAACCTTAA
R-O_cuniculus : AAGAACCCCGAAACC-AGACGAGCTATTTATGAGCAGTT--GAAA-GAACGAACCCGTCCATGTGGCAAAATGGTGGGAAGACTTGTAAATAGAGGTGAAAAGCCAACCGAGCCTGGTGATAGCTGGTTGTCCA-GAATAGAATTTTAGTTCAACTTTAA
A-O_cuniculus : AAGAACCCCGAAACC-AGACGAGCTATTTATGAGCAGTT--GAAA-GAACGAACCCGTCCATGTGGCAAAATGGTGGGAAGACTTGTAAATAGAGGTGAAAAGCCAACCGAGCCTGGTGATAGCTGGTTGTCCA-GAATAGAATTTTAGTTCAACTTTAA
R-M_musculus : AGAAACCCCGAAACC-AAACGAGCTACCTAAAAACAATT--T-TATGAATCAACTCGTCTATGTGGCAAAATAGTGAGAAGATTTTTAGGTAGAGGTGAAAAGCCTAACGAGCTTGGTGATAGCTGGTTACCCAAAAAATGAATTTAAGTTCAATTTTAA
A-M_musculus : AGAAACCCCGAAACC-AAACGAGCTACCTAAAAACAATT--T-TATGAATCAACTCGTCTATGTGGCAAAATAGTGAGAAGATTTTTAGGTAGAGGTGAAAAGCCTAACGAGCTTGGTGATAGCTGGTTACCCAAAAAATGAATTTAAGTTCAATTTTAA
R-R_norvegicus : AAGAACCCCGAAACC-AAACGAGCTACCTAAAAACAATT--T-CATGAATCAACCCGTCTATGTAGCAAAATAGTGGGAAGATTTTTAGGTAGAGGTGAAAAGCCTATCGAGCTTGGTGATAGCTGGTTGCCCAAAAAA-GAATTTCAGTTCAACTTTAA
A-R_norvegicus : AAGAACCCCGAAACC-AAACGAGCTACCTAAAAACAATT--T-CATGAATCAACCCGTCTATGTAGCAAAATAGTGGGAAGATTTTTAGGTAGAGGTGAAAAGCCTATCGAGCTTGGTGATAGCTGGTTGCCCAAAAAA-GAATTTCAGTTCAACTTTAA
R-M_coypus : AAAATTCCCGAAACC-AAACGAGCTACTTCTGAACAGCT--AACATGAGCAAACTCGTCTATGTAGCAAAATAGTGAGAAGATTTAGAAGTAGAGGTGAAAAGCCTATCGAGCTTGGTGATAGCTGGTTATCCAAATAA-GAATCTCAGTTCAGCTTTAA
A-M_coypus : AAAATTCCCGAAACC-AAACGAGCTACTTCTGAACAGCT--AACATGAGCAAACTCGTCTATGTAGCAAAATAGTGAGAAGATTTAGAAGTAGAGGTGAAAAGCCTATCGAGCTTGGTGATAGCTGGTTATCCAAATAA-GAATCTCAGTTCAGCTTTAA
R-A_platyrhynchos : TGCCATCCCGAAACCCAAGCGAGCTACTTACGAGCAGCTA---TTAGAGCGAACCCGTCTCTGTTGCAAAAGAGTGGGATGACTTGTCAGTAGAGGTGAAAAGCCAACCGAGCTGGGTGATAGCTGGTTACCTGTGAAATGAATCTAAGTTCTCCCTTAA
A-A_platyrhynchos : TGCCATCCCGAAACCCAAGCGAGCTACTTACGAGCAGCTA---TTAGAGCGAACCCGTCTCTGTTGCAAAAGAGTGGGATGACTTGTCAGTAGAGGTGAAAAGCCAACCGAGCTGGGTGATAGCTGGTTACCTGTGAAATGAATCTAAGTTCTCCCTTAA
R-G_gallus : TGCCTTCCCGAAACCCAAGCGAGCTACTTGCGAGCAGCTAAAATTTGAGCGAACCCGTCTCTGTTGCAAAAGAGCGGGATGACTTGCCAGTAGAGGTGAAAAGCCTACCGAGCTGGGTGATAGCTGGTTACCTGTCAAACGAATCTAAGTTCCCCCTTAA
A-G_gallus : TGCCTTCCCGAAACCCAAGCGAGCTACTTGCGAGCAGCTAAAATTTGAGCGAACCCGTCTCTGTTGCAAAAGAGTGGGATGACTTGCCAGTAGAGGTGAAAAGCCTACCGAGCTGGGTGATAGCTGGTTACCTGTCAAACGAATCTAAGTTCCCCCTTAA
 AAGCACCCCGAAACCCAGACGAGCTACCTATGAACAGTTATAAAATGAACCAACTCATCTATGTGGCAAAATAGTGAGAAGATTTGTAGGTAGAGGTGAAAAGCCTAACGAGCCTGGTGATAGCTGGTTGCCCAGAAAAAGAATTTTAGTTCAACTTTAA

 * 2900 * 2920 * 2940 * 2960 * 2980 * 3000 * 3020 * 3040
R-E_caballus : ATTTACCT---AAAAACTAC---TC------AATTCTAATGTAAAT----TTAAATTATAGTCTAAAAAGGTACAGCTTTTTAGATACAGGTTACAACCTTCATTAGAGAGTAAG-AACAAGATAA-----ACC-CATAGTTGGCTTAAAAGCAGCCATC
A-E_caballus : ATTTACCT---AAAAACTAC---TC------AATTCTAATGTAAAT----TTAAATTATAGTCTAAAAAGGTACAGCTTTTTAGATACAGGTTACAACCTTCATTAGAGAGTAAG-AACAAGATAA-----ACC-CATAGTTGGCTTAAAAGCAGCCATC
R-C_bactrianus : AAATACCT---AAAAAACTG---CT------AATTTTATTGTATTT----TTAAAAGCTAGTCTAAAAGGGTACAGCTTTTTAGATTAAGGATACAACCTTCCTTAGAGAGTAAA-AACAA-CCAA-----TAC-CATAGTAGGCTTAAAAGCAGCCATC
A-C_bactrianus : AAATACCT---AAAAAACTG---CT------AATTTTATTGTATTT----TTAAAAGCTAGTCTAAAAGGGTACAGCTTTTTAGATTAAGGATACAACCTTCCTTAGAGAGTAAA-AACAA-CCAA-----TAC-CATAGTAGGCTTAAAAGCAGCCATC
R-C_lupus_familiaris : ATTTACCT---AAAAAAAAT---AA------AATTTTAATGTAAAT----TTAAAATATAGTCTAAGAAGGTACAGCTTCTTAGAATCAGGATACAACCTTTATTAGAGAGTATA-TACTA-ATAT-----CAC-CATAGTTGGCTTAAAAGCAGCCACC
A-C_lupus_familiaris : ATTTACCT---AAAAAAAAT---AA------AATTTTAATGTAAAT----TTAAAATATAGTCTAAGAAGGTACAGCTTCTTAGAATCAGGATACAACCTTTATTAGAGAGTATA-TACTA-ATAT-----CAC-CATAGTTGGCTTAAAAGCAGCCACC
R-N_procyonoides : ATTTACCT---CAAAAAAAC---AA------AATTTAAATGTAAAT----TTAAAATATAGTCTAAAAAGGTACAGCTTTTTAGAATAAGGACACAACCTTTATTAGCGAGTAAA-TATTG-ACAT-----CAC-CATAGTTGGCCTAAAAGCAGCCATC
A-N_procyonoides : ATTTACCT---CAAAAAAAC---AA------AATTTAAATGTAAAT----TTAAAATATAGTCTAAAAAGGTACAGCTTTTTAGAATAAGGACACAACCTTTATTAGCGAGTAAA-TATTG-ACAT-----CAC-CATAGTTGGCCTAAAAGCAGCCATC
R-V_lagopus : ATTTACCA---AAAAAAAAC---AA------AGTTCTAATGTAAAT----TTAAAATATAATCTAAAAAGGTACAGCTTTTTAGAACAAGGACACAACCTTTATTAGAGAGTAAA-TACTA-ACAC-----AAC-CATAGTTGGCCTAAAAGCAGCCATC
A-V_lagopus : ATTTACCA---AAAAAAAAC---AA------AGTTCTAATGTAAAT----TTAAAATATAATCTAAAAAGGTACAGCTTTTTAGAACAAGGACACAACCTTTATTAGAGAGTAAA-TACTA-ACAC-----AAC-CATAGTTGGCCTAAAAGCAGCCATC
R-M_putorius : ATTTACCT---AATAACCCC---CA------AATTGTAATGTAAAT----TTAAAATATAGTCTAAAAAGGTACAGCTTTTTAGAATAAGGATACAACCTTGCTTAGAGAGTAAA-ATTAA-ACAA-----AAC-CATAGTAGGCCTAAGAGCAGCCACC
A-M_putorius : ATTTACCT---AATAACCCC---CA------AATTGTAATGTAAAT----TTAAAATATAGTCTAAAAAGGTACAGCTTTTTAGAATAAGGATACAACCTTGCTTAGAGAGTAAA-ATTAA-ACAA-----AAC-CATAGTAGGCCTAAGAGCAGCCACC
R-B_taurus : AGATACC-----AAAAATTCAAATA------AACCCCACTGTAGCT----TTAAAAGTTAGTCTAAAAAGGTACAGCCTTTTAGAA-ACGGATACAACCTTGACTAGAGAGTAAA-ATTT-AACAC-----TAC-CATAGTAGGCCTAAAAGCAGCCATC
A-B_taurus : AGATACC-----AAAAATTCAAATA------AACCCCACTGTAGCT----TTAAAAGTTAGTCTAAAAAGGTACAGCCTTTTAGAA-ACGGATACAACCTTGACTAGAGAGTAAA-ATTT-AACAC-----TAC-CATAGTAGGCCTAAAAGCAGCCATC
R-O_aries : AGATACC-----AAAAATACAAATA------AATCCCACTGTATCT----TTAAAAGTTAGTCTAAAAAGGTACAGCCTTTTAGAA-ATGGGTACAACCTTCACTAGAGAGTAAG-ATCT-AAAAA-----TAC-CATAGTAGGCCTAAAAGCAGCCATC
A-O_aries : AGATACC-----AAAAATACAAATA------AATCCCACTGTATCT----TTAAAAGTTAGTCTAAAAAGGTACAGCCTTTTAGAA-ATGGGTACAACCTTCACTAGAGAGTAAG-ATCT-AAAAA-----TAC-CATAGTAGGCCTAAAAGCAGCCATC
R-S_scrofa : AAATACCC---CAAAAACCC---TA------AATTCCAATGTATTT----TTAAGAGATAGTCTAAAAAGGTACAGCTTTTTAGAA-ACGGATACAACCTTGACTAGAGAGTAAA-ATCTTAATAC-----TAC-CATAGTAGGCCTAAAAGCAGCCATC
A-S_scrofa : AAATACCC---CAAAAACCC---TA------AATTCCAATGTATTT----TTAAGAGATAGTCTAAAAAGGTACAGCTTTTTAGAA-ACGGATACAACCTTGACTAGAGAGTAAA-ATCTTAATAC-----TAC-CATAGTAGGCCTAAAAGCAGCCATC
R-O_cuniculus : ATTTTCCT---AAAGGACAT---AA------AACCCTAATGAAAAT----TTAAATGTTACTCTAAAGAGGGACAGCTCTTTAGATATAGGATACAGCCTTTTATAAAGAGTAAG-TTATTTTTCA-----ATC-CATAGTTGGCTTAAAAGCAGCCATC
A-O_cuniculus : ATTTTCCT---AAAGGACAT---AA------AACCCTAATGAAAAT----TTAAATGTTACTCTAAAGAGGGACAGCTCTTTAGATATAGGACACAGCCTTTTATAAAGAGTAAG-TTATTTT-CA-----ATC-CATAGTTGGCTTAAAAGCAGCCATC
R-M_musculus : ACTTGCTA---AAAAAACAA--CAA------AATCAAAAAGTAAGT----TTAGATTATAGCCAAAAGAGGGACAGCTCTTCTGGAAC-GGAAAAAACCTTTAATAGTGAATAATTAACAAAACAGCTTTTAAC-CATTGTAGGCCTAAAAGCAGCCACC
A-M_musculus : ACTTGCTA---AAAAAACAA--CAA------AATCAAAAAGTAAGT----TTAGATTATAGCCAAAAGAGGGACAGCTCTTCTGGAAC-GGAAAAAACCTTTAATAGTGAATAATTAACAAAACAGCTTTTAAC-CATTGTAGGCCTAAAAGCAGCCACC
R-R_norvegicus : GCTTACCA---TCAGAACAA--CAA------A-TCAAAATGTAAAC----TTAAAATATAGCCAAAAGAGGGACAGCTCTTTAGGAAAAGGAAAAAACCTTAAATAGTGAATAAACAACTACA-ATCACTTAAC-CATTGTAGGCTTAAAAGCAGCCATC
A-R_norvegicus : GCTTACCA---TCAGAACAA--CAA------A-TCAAAATGTAAAC----TTAAAATATAACCAAAAGAGGGACAGCTCTTTAGGAAAAGGAAAAAACCTTAAATAGTGAATAAACAACTACA-ACCACTTAAC-CATTGTAGGCTTAAAAGCAGCCATC
R-M_coypus : GTTTTCC----TAAAAACCA--TAA------AATTTAAATGAAAAC----TTAAATGCTAATTTAAGGAGGGACAGCTCCTTAAGTAA-GGATACATCCTTAAACAGAGGGTAAA-AACTATAAAT------AC-CATAGTAGGCTTAAAAGCAGCCACC
A-M_coypus : GTTTTCC----TAAAAACCA--TAA------AATTTAAATGAAAAC----TTAAATGCTAATTTAAGGAGGGACAGCTCCTTAAGTAA-GGATACATCCTTAAACAGAGGGTAAA-AACTATAAAT------AC-CATAGTAGGCTTAAAAGCAGCCACC
R-A_platyrhynchos : TCTTCCCTACCGGACAACACCCAG-------AACCACAATGAGAT---GATTAAGAGCTATTTAATGGAGGTACAGCTCCATTAAAAAAGGACACAACCTCGACTAGTGGATAAA--TCTAATCACCAACCTT---ACTGTGGGCCTTAAAGCAGCCATC
A-A_platyrhynchos : TCTTCCCTACCGGACAACACCCAG-------AACCACAATGAGAT---GATTAAGAGCTATTTAATGGAGGTACAGCTCCATTAAAAAAGGACACAACCTCGACTAGTGGATAAA--TCTAATCACCAACCTT---ACTGTGGGCCTTAAAGCAGCCATC
R-G_gallus : CCCACCC--CCTAAAGACACCCACCTTTGTCAACCTTGAGAACGTTGGGGTTAAGAGCAATTCGATGGGGGTACAGCTCCATCGAAAAAGAACACAACCTCCTCCAGCGGATAA-----TAATCACCCCTCCCCGCACTGTGGGCCTTCAAGCAGCCACC
A-G_gallus : CCCACCC--CCTAAAGACACCCACCTTTGTCAACCTTGAGAACGTTGGGGTTAAGAGCAATTCGATGGGGGTACAGCTCCATCGAAAAAGAACACAACCTCCTCCAGCGGATAA-----TAATCACCCCTCCCCGCACTGTGGGCCTTCAAGCAGCCACC
 ATTTACCTACCAAAAAACACMMAAATTTGTCAATTCTAATGTAAATGGGRTTAAAAGATAGTCTAAAAAGGTACAGCTTTTTAGAAAAAGGATACAACCTTTATTAGAGAGTAAAYAACTAAACACCACTYAACGCATAGTAGGCCTAAAAGCAGCCATC

 * 3060 * 3080 * 3100 * 3120 * 3140 * 3160 * 3180 * 3200
R-E_caballus : AATTAA-GAAAGCGTTCAAGCTCAA-CGAC-ACA-TC---TATCTTAATCCCAACAATCAACCCAAACTAACTCCTAATCTCAT--ACTGGACTATTCTATCAACACATAGAAGCAATAATGTTAATATGAGTAACAAGAA-----TTATTT-CTCCTT-
A-E_caballus : AATTAA-GAAAGCGTTCAAGCTCAA-CGAC-ACA-TC---TATCTTAATCCCAACAATCAACCCAAACTAACTCCTAATCTCAT--ACTGGACTATTCTATCAACACATAGAAGCAATAATGTTAATATGAGTAACAAGAA-----TTATTT-CTCCTT-
R-C_bactrianus : AATTAA-GAAAGCGTTCAAGCTCAA-CATC-AAACTA---AACCTTAATCCCAATAGTTA---ACAAGGAACTCCTAATCCGAT--ACTGGACTAATCTATTAATTTATAGAGGCAATAATGTTAATATGAGTAACAAGAA-----ATATTT-CTCCTT-
A-C_bactrianus : AATTAA-GAAAGCGTTCAAGCTCAA-CATC-AAACTA---AACCTTAATCCCAATAGTTA---ACAAGGAACTCCTAATCCGAT--ACTGGACTAATCTATTAATTTATAGAGGCAATAATGTTAATATGAGTAACAAGAA-----ATATTT-CTCCTT-
R-C_lupus_familiaris : AATTGA-GAAAGCGTTCCAGCTCAA-CAAA-CAA-TA---TAACTTAATCCCAACCATAC---TACATCAACTCCTAATTATACCCCCTGGGTCATTCTATTTAAGTATAGAAGCAATAATGCTAGTATGAGTAACAAGAA-----CCATTTTCTCCCC-
A-C_lupus_familiaris : AATTGA-GAAAGCGTTCCAGCTCAA-CAAA-CAA-TA---TAACTTAATCCCAACCATAC---TACATCAACTCCTAATTATACCCCCTGGGTCATTCTATTTAAGTATAGAAGCAATAATGCTAGTATGAGTAACAAGAA-----CCATTTTCTCCCC-
R-N_procyonoides : AATTGA-GAAAGCGTTCAAGCTCAA-CAAA-AGA-TG---CAACTTAATCCCAACAATGA---TATATCAACTCCTAACC-CACTCCCTGGGTCATTCTATTTAAATATAGAAGCAACAATGCTAGTATGAGTAACAAGAA-----ACAATT-CTCCTC-
A-N_procyonoides : AATTGA-GAAAGCGTTCAAGCTCAA-CAAA-AGA-TG---CAACTTAATCCCAACAATGA---TATATCAACTCCTAACC-CACTCCCTGGGTCATTCTATTTAAATATAGAAGCAACAATGCTAGTATGAGTAACAAGAA-----ACAATT-CTCCTC-
R-V_lagopus : AATTGA-GAAAGCGTTCAAGCTCAA-CAAA-TAA-TG---CAACTTAATCCCAACCATAT---TGCATAAACTCCTAACT-TAC-CCCTGGGTTATTCTATCTAAGTATAGAAGCAACAATGCTAGTATGAGTAACAAGAA-----TTATTT-CTCCCC-
A-V_lagopus : AATTGA-GAAAGCGTTCAAGCTCAA-CAAA-TAA-TG---CAACTTAATCCCAACCATAT---TGCATAAACTCCTAACT-TAC-CCCTGGGTTATTCTATCTAAGTATAGAAGCAACAATGCTAGTATGAGTAACAAGAA-----TTATTT-CTCCCC-
R-M_putorius : AATTAA-GAAAGCGTTCAAGCTCAA-CAAT-ACA-GC---CACCTTAATCCCTATAATCA---TATA-CAACTCCTAACA-CAC-TACTGGGCTAATCTATTTTATAATAGAAGCAATAATGCTAGTATGAGTAACAAGAA-----ACATTT-CTCCTT-
A-M_putorius : AATTAA-GAAAGCGTTCAAGCTCAA-CAAT-ACA-GC---CACCTTAATCCCTATAATCA---TATA-CAACTCCTAACA-CAC-TACTGGGCTAATCTATTTTATAATAGAAGCAATAATGCTAGTATGAGTAACAAGAA-----ACATTT-CTCCTT-
R-B_taurus : AATTAA-GAAAGCGTTAAAGCTCAA-CAAC-AAAAAT---TAAATAGATTCCAACAACAAA---TGATTAACTCCTAGCCCCAA-TACTGGACTAATCTATTATAGAATAGAAGCAATAATGTTAATATGAGTAACAAGAA-----AAATTTTCTCCTT-
A-B_taurus : AATTAA-GAAAGCGTTAAAGCTCAA-CAAC-AAAAAT---TAAATAGATTCCAACAACAAA---TGATTAACTCCTAGCCCCAA-TACTGGACTAATCTATTATAGAATAGAAGCAATAATGTTAATATGAGTAACAAGAA-----AAATTTTCTCCTT-
R-O_aries : AATTAA-GAAAGCGTTAAAGCTCAA-CAAC-AATAGT---ATTATTAATCCCAGCAATAACA-TTAGCCAACTCCTAGATTTAA-TACTGGACTATTCTATTACTAAATAGAAG-AATAATGTTAATATGAGTAACAAGAA-----ATATTTTCTCCTC-
A-O_aries : AATTAA-GAAAGCGTTAAAGCTCAA-CAAC-AATAGT---ATTATTAATCCCAGCAATAACA-TTAGCCAACTCCTAGATTTAA-TACTGGACTATTCTATTACTAAATAGAAGCAATAATGTTAATATGAGTAACAAGAA-----ATATTTTCTCCTC-
R-S_scrofa : AATTGA-GAAAGCGTTAAAGCTCAA-CAAA-TTCACC---AACAT-AATCCCAAAAACTAA---TAACAAACTCCTAG-CCCAA-TACCGGACTAATCTATTGAAACATAGAAGCAATAATGTTAATATGAGTAACAAGAA-----GC-CTTTCTCCTC-
A-S_scrofa : AATTGA-GAAAGCGTTAAAGCTCAA-CAAA-TTCACC---AACAT-AATCCCAAAAACTAA---TAACAAACTCCTAG-CCCAA-TACCGGACTAATCTATTGAAACATAGAAGCAATAATGTTAATATGAGTAACAAGAA-----GC-CTTTCTCCTC-
R-O_cuniculus : AATTAA-GAAAGCGTTAAAGCTCAA-CAACTAAAAAC---CAACTTAATAAGAATATTTTA---AAACGAACTCTTATAAACCCTAACTGGACTAATCTATAAATCTATAGAAGAAATAATGCTAATATAAGTAACAAGAA-----TACTATTCTCCTT-
A-O_cuniculus : AATTAA-GAAAGCGTTAAAGCTCAA-CAACTAAAAAC---CAACTTAATAAGAATATTTTA---AAACGAACTCTTATAAACCCTAACTGGACTAATCTATAAATCTATAGAAGAAATAATGCTAATATAAGTAACAAGAA-----TACTATTCTCCTT-
R-M_musculus : AATAAA-GAAAGCGTTCAAGCTCAA-CATAAA-ATTT-CA---ATTAATTCCATAA-TTT---ACACCAACTTCCTAAACTTAA-AATTGGGTTAATCTATAACTTTATAGATGCAACACTGTTAGTATGAGTAACAAGAATT---CCAATT-CTCCAG-
A-M_musculus : AATAAA-GAAAGCGTTCAAGCTCAA-CATAAA-ATTT-CA---ATTAATTCCATAA-TTT---ACACCAACTTCCTAAACTTAA-AATTGGGTTAATCTATAACTTTATAGATGCAACACTGTTAGTATGAGTAACAAGAATT---CCAATT-CTCCAG-
R-R_norvegicus : AATAAA-GAAAGCGTTCAAGCTCAA-CATACATACTTACACACACTAATTCCACAA-ACC---TCAATAAATTCCTATATCACA-AATTGGGCTAATCTATAGATCCATAGATGAAATACTGTTAATATGAGTAACAAGAA-----CCAATT-CTCCTA-
A-R_norvegicus : AATAAA-GAAAGCGTTCAAGCTCAA-CATACATACTTACACACACTAATTCCACAA-ACC---TCAATAAATTCCTATATTACA-AATTGGGCTAATCTATAGACCCATAGATGAAATACTGTTAATATGAGTAACAAGAA-----CCAATT-CTCCTA-
R-M_coypus : AATTAA-GAAAGCGTTCTAGCTCAA-CA-AAACACCT--A-ATTTTATCCCTAAAACATA---TCAATAAACCCCTGTCGAGAC-AATTGGATTAATCTATTATTTCATAGAAGAAACAATGTTAATATTAGTAATTAGAA-----TTATTT-CTCCTT-
A-M_coypus : AATTAA-GAAAGCGTTCTAGCTCAA-CA-AAACACCT--A-ATTTTATCCCTAAAACATA---TCAATAAACCCCTGTCGAGAC-AATTGGATTAATCTATTATTTCATAGAAGAAACAATGTTAATATTAGTAATTAGAA-----TTATTT-CTCCTT-
R-A_platyrhynchos : AACAAA-GAGTGCGTCAAAGCTCCA-CACTCAAA------------AATGCCAAAACAAG----AT--GAATCCCTTACCACA---AACAGGTTAACCTATGAAT--ATAGGAGAATTAATGCTAAAATGAGTAACTTGGGGCCACACCCACCCCTCTAG
A-A_platyrhynchos : AACAAA-GAGTGCGTCAAAGCTCCA-CACTCAAA------------AATGCCAAAACAAG----AT--GAATCCCTTACCACA---AACAGGTTAACCTATGAAT--ATAGGAGAATTAATGCTAAAATGAGTAACTTGGGGCCACACCCACCCCTCTAG
R-G_gallus : AACAAAAGAGTGCGTCAAAGCTCCCTCATTAAAA------------AAT-CTAAAACCCT----ATTTGACTCCCTCAACCAA---AGCAGGTTAACCTATGACA--ATAGAAGAATCAATGCTAAAATGAGTAATCTGGA-----ACCTATCCTCCTA-
A-G_gallus : AACAAAAGAGTGCGTCAAAGCTCCCTCATTAAAA------------AAT-CTAAAACCCT----ATTTGACTCCCTCAACCAA---AGCAGGTTAACCTATGACA--ATAGAAGAATCAATGCTAAAATGAGTAATCTGGA-----ACCTATCCTCCTA-
 AATTAAAGAAAGCGTTCAAGCTCAATCAAAAAAAATTACACAACTTAATCCCAAMAATWAAMCTAAATAAACTCCTAACCCCACTWACTGGGCTAATCTATTAAAWTATAGAAGCAATAATGTTAATATGAGTAACAAGAAKYCACACATTTTCTCCTTG

 * 3220 * 3240 * 3260 * 3280 * 3300 * 3320 * 3340 * 3360
R-E_caballus : --GCATAAGCTTATATCAG--AACGAATAC-TCACTGATAGTTAACAACAA-GATAGGGATAATCCAAAAACTAA-TCATCTA--TTTA-AACCATTGTTAACCCAACACAGGCATGCATCTATAAGGAAAGATTAAAAGAAGTAAAAGGAACTCGGCAA
A-E_caballus : --GCATAAGCTTATATCAG--AACGAATAC-TCACTGATAGTTAACAACAA-GATAGGGATAATCCAAAAACTAA-TCATCTA--TTTA-AACCATTGTTAACCCAACACAGGCATGCATCTATAAGGAAAGATTAAAAGAAGTAAAAGGAACTCGGCAA
R-C_bactrianus : --GCATAAGCTTATGTCAGC-AACGAATACTCTACTGACAGTTAACA-CTT-AATAAACTTAACCCACCGATAAACA-ATTTA--TTAA-ATCCACTGTTAATCCGACACAGGGATGCA---TTAAGGAAAGATTAAAAGAAGCAAAAGGAACTCGGCAA
A-C_bactrianus : --GCATAAGCTTATGTCAGC-AACGAATACTCTACTGACAGTTAACA-CTT-AATAAACTTAACCCACCGATAAACA-ATTTA--TTAA-ATCCACTGTTAATCCGACACAGGGATGCA---TTAAGGAAAGATTAAAAGAAGCAAAAGGAACTCGGCAA
R-C_lupus_familiaris : --GCATAAGCTTATATCAGG-AACGGATAGACCACTGATAGTTAACAATCT-GATAATATCAACCCAAAAATGAA-ATACTTA--TCCA-CCCCATTGTTAACCCAACACAGGTATGCAT--TCAAGGAAAGATTAAAAGGAGTAAAAGGAACTCGGCAA
A-C_lupus_familiaris : --GCATAAGCTTATATCAGG-AACGGATAGACCACTGATAGTTAACAATCT-GATAATATCAACCCAAAAATGAA-ATACTTA--TCCA-CCCCATTGTTAACCCAACACAGGTATGCAT--TCAAGGAAAGATTAAAAGGAGTAAAAGGAACTCGGCAA
R-N_procyonoides : --GCATGAGCTTATATCAGA-AACGGATAAACCACTGATAGTTAACAACCC-GATAATATTAACCTAATAATAAA-ATACTTA--TCAA-CCCTATTGTTAACCCGACACAGGCATGCGC--ACAAGGAAAGATTAAAAGAAGTAAAAGGAACTCGGCAA
A-N_procyonoides : --GCATGAGCTTATATCAGA-AACGGATAAACCACTGATAGTTAACAACCC-GATAATATTAACCTAATAATAAA-ATACTTA--TCAA-CCCTATTGTTAACCCGACACAGGCATGCGC--ACAAGGAAAGATTAAAAGAAGTAAAAGGAACTCGGCAA
R-V_lagopus : --GCATAAGCTTATATCAGA-AACGGATAAACCACTGATAGTTAACAACCT-GATAAGACTAATCCAAAAATAAA-ATACTTA--TCTA-CCCCATTGTTAACCCAACACAGGCATGCAC--CCAAGGAAAGATTAAAAGAAGTAAAAGGAACTCGGCAA
A-V_lagopus : --GCATAAGCTTATATCAGA-AACGGATAAACCACTGATAGTTAACAACCT-GATAAGACTAATCCAAAAATAAA-ATACTTA--TCTA-CCCCATTGTTAACCCAACACAGGCATGCAC--CCAAGGAAAGATTAAAAGAAGTAAAAGGAACTCGGCAA
R-M_putorius : --GCATAAGCTTATAACAGTTAACGAATAC-CCACTGATAGTTAACAACAA-GATAAAGATAAACCACTAATAAATATTCTTA--TCAA-ACCAATTGTTAGTCCAACACAGGCATGCA---ACAAGGAAAGATTAAAAGAAGTAAAAGGAACTCGGCAA
A-M_putorius : --GCATAAGCTTATAACAGTTAACGAATAC-CCACTGATAGTTAACAACAA-GATAAAGATAAACCACTAATAAATATTCTTA--TCAA-ACCAATTGTTAGTCCAACACAGGCATGCA---ACAAGGAAAGATTAAAAGAAGTAAAAGGAACTCGGCAA
R-B_taurus : --GCATAAGTCTAAGTCAGT-GCCTGATAATACTCTGACCACTAACAGTC--AATAAAAATAATCCAACAATAAA-CAATTTA--TTGA-TTATACTGTTAACCCAACACAGGAGTGCAT--CTAAGGAAAGATTAAAAGAAGTAAAAGGAACTCGGCAA
A-B_taurus : --GCATAAGTCTAAGTCAGT-GCCTGATAATACTCTGACCACTAACAGTC--AATAAAAATAATCCAACAATAAA-CAATTTA--TTGA-TTATACTGTTAACCCAACACAGGAGTGCAT--CTAAGGAAAGATTAAAAGAAGTAAAAGGAACTCGGCAA
R-O_aries : --GCACAAGTTTAAGTCAGT-AACTGATAATACCCTGACCGTTAACAGTA--AATAAAAATAACCCAACAATAAA-TGATTTA--TTAC-TTATACTGTTAACCCAACACAGGAGTGCAC--CCA-GGAAAGATTCAAAGAAGTAAAAGGAACTCGGCAA
A-O_aries : --GCACAAGTTTAAGTCAGT-AACTGATAATACCCTGACCGTTAACAGTA--AATAAAAATAACCCAACAATAAA-TGATTTA--TTAC-TTATACTGTTAACCCAACACAGGAGTGCAC--CCA-GGAAAGATTCAAAGAAGTAAAAGGAACTCGGCAA
R-S_scrofa : --GCACACGCTTACATCAGT-AACTAATAATATACTGATAATTAACAATC--AATAAAC-CAAAACAACACTAAA-GCGTTTA--TTAA-TTATATTGTTAACCCAACACAGGAGTGCAC--CAA-GGAAAGATTAAAAGAAGTAAAAGGAACTCGGCAA
A-S_scrofa : --GCACACGCTTACATCAGT-AACTAATAATATACTGATAATTAACAATC--AATAAAC-CAAAACAACACTAAA-GCGTTTA--TTAA-TTATATTGTTAACCCAACACAGGAGTGCAC--CAA-GGAAAGATTAAAAGAAGTAAAAGGAACTCGGCAA
R-O_cuniculus : --GCACAAGCTTATATCAG--ATCGGATGC-CCACTGATAGTTAACAGCCCCAATAGAAATAATCCACAAATTAA-TAATCTA--TTAACTTTCACTGTTAACCCAACACAGGAGTGCTA--TATAGGAAAGATTAAAAGAAGAAAAAGGAACTCGGCAA
A-O_cuniculus : --GCACAAGCTTATATCAG--ATCGGATGC-CCACTGATAGTTAACAGCCCTAATAGAAATAATCCACAAATTAA-TAATCTA--TTAACTTTCACTGTTAACCCAACACAGGAGTGCTA--TATAGGAAAGATTAAAAGAAGAAAAAGGAACTCGGCAA
R-M_musculus : --GCATACGCGTATAACAAC--TCGGATAA-CCATTGTTAGTTAATCAGACTATAGGCAATAATCACACTATAAA-TAATCCACCTATAACTTCTCTGTTAACCCAACACCGGAATGCCT---AAAGGAAAGATCCAAAAAGATAAAAGGAACTCGGCAA
A-M_musculus : --GCATACGCGTATAACAAC--TCGGATAA-CCATTGTTAGTTAATCAGACTATAGGCAATAATCACACTATAAA-TAATCCACCTATAACTTCTCTGTTAACCCAACACCGGAATGCCT---AAAGGAAAGATCCAAAAAGATAAAAGGAACTCGGCAA
R-R_norvegicus : --GCACAAGTGTATGACAAC--CCGGATAA-CCATTGTCAATTA-TCGAATCATAGGTACTAACCCAACAATAAAATTACCTATCCCTAACTC----GTTAGCCCAACACAGGCGTGCTT---TAAGGAAAGATTAAAAAAAGTAAAAGGAACTCGGCAA
A-R_norvegicus : --GCACAAGTGTATGACAAC--CCGGATAA-CCATTGTCAATTA-TCGAATCATAGGTACTAACCCAACAATAAAATTACCTATCCCTAACTC----GTTAGCCCAACACAGGCGTGCTT---TAAGGAAAGATTAAAAAAAGTAAAAGGAACTCGGCAA
R-M_coypus : --GCACAAGCCTA-AACTAT--TC----AA-ACATAATAAATTAACAACCATATAAAAGATACACCTACAAGTAGATTAT-TA-CTGCAATT-----GTTATCCCAACCCAGGAGTGCAA---TAAGGAAAGACTAAAACAAGTAAAAGGAACTCGGCAA
A-M_coypus : --GCACAAGCCTA-AACTAT--TC----AA-ACATAATAAATTAACAACCATATAAAAGATACACCTACAAGTAGATTAT-TA-CTGCAATT-----GTTATCCCAACCCAGGAGTGCAA---TAAGGAAAGACTAAAACAAGTAAAAGGAACTCGGCAA
R-A_platyrhynchos : CGGCGCAAGCTTACATGAGA--------ACATTATTAACAGACCCAGACAT--ATACAAAAACTCCTACAAG-AC--CAGGTA--TAAACTCACCCTGTTAACCCGACTCAGGAGCGCCC---ATAAGAGAGATTAAAATCTGTGAAAGGAACTCGGCAA
A-A_platyrhynchos : CGGCGCAAGCTTACATGAGA--------ACATTATTAACAGACCCAGACAT--ATACAAAAACTCCTACAAG-AC--CAGGTA--TAAACTCACCCTGTTAACCCGACTCAGGAGCGCCC---ATAAGAGAGATTAAAATCTGTGAAAGGAACTCGGCAA
R-G_gallus : CGGCGTAAACTTACATTAAT--------ACATTATTAACAGAACTCAACTT--ATACCCCCACACTAACAAGCAA--TACGTA--TTC-CTCAATCTGTTAAGCCAACCCAGGAGCGCCC---ACAGGAT-GATTAAAACCTACAGAAGGAACTCGGCAA
A-G_gallus : CGGCGTAAACTTACATTAAT--------ACATTATTAACAGAACTCAACTT--ATACCCCCACACTAACAAGCAA--TACGTA--TTC-CTCAATCTGTTAAGCCAACCCAGGAGCGCCC---ACAGGAT-GATTAAAACCTACAGAAGGAACTCGGCAA
 CGGCATAAGCTTATATCAGTTAACGGATAAACCACTGATAGTTAACAACCTTAATAAAAATAATCCAACAATAAAATTATTTAYCTTAAMTTCCACTGTTAACCCAACACAGGAGTGCACCTCHAAGGAAAGATTAAAAGAAGTAAAAGGAACTCGGCAA

 * 3380 * 3400 * 3420 * 3440 * 3460 * 3480 * 3500 * 3520
R-E_caballus : ACAC-AAACCCCGCCTGTTTACCAAAAACATCACCTCTAGC--ATTT-CCAGTATTAGAGGCACTGCCTGCCCAGTGAC---ATCTGTTTAAACGGCCGCGGTATCCTAACCGTGCAAAGGTAGCATAATCACTTGTTCCCTAAATAGGGACTTGTATGA
A-E_caballus : ACAC-AAACCCCGCCTGTTTACCAAAAACATCACCTCTAGC--ATTT-CCAGTATTAGAGGCACTGCCTGCCCAGTGAC---ATCTGTTTAAACGGCCGCGGTATCCTAACCGTGCAAAGGTAGCATAATCACTTGTTCCCTAAATAGGGACTTGTATGA
R-C_bactrianus : ACAC-GAGCCCCGCCTGTTTACCAAAAACATCACCTCTAGC--ATTA-CTAGTATTAGAGGCACTGCCTGCCCAGTGAC---ATTAGTTAAA-CGGCCGCGGTATCCTGACCGTGCAAAGGTAGCATAATCATTTGTTCTTTAAATAGGGACTTGTATGA
A-C_bactrianus : ACAC-GAGCCCCGCCTGTTTACCAAAAACATCACCTCTAGC--ATTA-CTAGTATTAGAGGCACTGCCTGCCCAGTGAC---ATTAGTTAAA-CGGCCGCGGTATCCTGACCGTGCAAAGGTAGCATAATCATTTGTTCTTTAAATAGGGACTTGTATGA
R-C_lupus_familiaris : ACAC-AAACCCCGCCTGTTTACCAAAAACATCACCTCCAGC--ATTT-CTAGTATTGGAGGCACTGCCTGCCCGGTGAC---ACTTGTTTAA-CGGCCGCGGTATCCTGACCGTGCAAAGGTAGCATAATCATTTGTTCTCTAAATAGGGACTTGTATGA
A-C_lupus_familiaris : ACAC-AAACCCCGCCTGTTTACCAAAAACATCACCTCCAGC--ATTT-CTAGTATTGGAGGCACTGCCTGCCCGGTGAC---ACTTGTTTAA-CGGCCGCGGTATCCTGACCGTGCAAAGGTAGCATAATCATTTGTTCTCTAAATAGGGACTTGTATGA
R-N_procyonoides : TCAC-AAGCCCCGCCTGTTTACCAAAAACATCACCTCCAGC--ATAC-CCAGTATTGGAGGCACTGCCTGCCCGGTGAC---AATTGTTTAA-CGGCCGCGGTATCCTGACCGTGCAAAGGTAGCATAATCATTTGTTCTCTAAATAGGGACTTGCATGA
A-N_procyonoides : TCAC-AAGCCCCGCCTGTTTACCAAAAACATCACCTCCAGC--ATAC-CCAGTATTGGAGGCACTGCCTGCCCGGTGAC---AATTGTTTAA-CGGCCGCGGTATCCTGACCGTGCAAAGGTAGCATAATCATTTGTTCTCTAAATAGGGACTTGCATGA
R-V_lagopus : ACAC-AAACCCCGCCTGTTTACCAAAAACATCACCTCCAGC--ATTC-CCAGTATTGGAGGCACTGCCTGCCCAGTGAC---GTCCGTTAAA-CGGCCGCGGTATCCTGACCGTGCAAAGGTAGCATAATCATTTGTTCCTTAAATAGGGACTTGTATGA
A-V_lagopus : ACAC-AAACCCCGCCTGTTTACCAAAAACATCACCTCCAGC--ATTC-CCAGTATTGGAGGCACTGCCTGCCCAGTGAC---GTCCGTTAAA-CGGCCGCGGTATCCTGACCGTGCAAAGGTAGCATAATCATTTGTTCCTTAAATAGGGACTTGTATGA
R-M_putorius : ACTC-AAACCCCGCCTGTTTACCAAAAACATCACCTCCAGC--ATAT-CTAGTATTGGAGGCACTGCCTGCCCAGTGAC---ACTAGTTTAA-CGGCCGCGGTATCCTGACCGTGCAAAGGTAGCATAATCATTTGTTCTCTAAATAGGGACTTGTATGA
A-M_putorius : ACTC-AAACCCCGCCTGTTTACCAAAAACATCACCTCCAGC--ATAT-CTAGTATTGGAGGCACTGCCTGCCCAGTGAC---ACTAGTTTAA-CGGCCGCGGTATCCTGACCGTGCAAAGGTAGCATAATCATTTGTTCTCTAAATAGGGACTTGTATGA
R-B_taurus : ACAC-AAACCCCGCCTGTTTACCAAAAACATCACCTCCAGC--ATTC-CCAGTATTGGAGGCATTGCCTGCCCAGTGAC---AACTGTTTAA-CGGCCGCGGTATCCTGACCGTGCAAAGGTAGCATAATCATTTGTTCTCTAAATAAGGACTTGTATGA
A-B_taurus : ACAC-AAACCCCGCCTGTTTACCAAAAACATCACCTCCAGC--ATTC-CCAGTATTGGAGGCATTGCCTGCCCAGTGAC---AACTGTTTAA-CGGCCGCGGTATCCTGACCGTGCAAAGGTAGCATAATCATTTGTTCTCTAAATAAGGACTTGTATGA
R-O_aries : ACACTAAACCCCGCCTGTTTACCAAAAACATCACCTCCAGC--ATCC-CTAGTATTGGAGGCACTGCCTGCCCAGTGACT--AAACGTTAAA-CGGCCGCGGTATTCTGACCGTGCAAAGGTAGCATAATCATTTGTTCTCTAAATAAGGACTTGTATGA
A-O_aries : ACACTAAACCCCGCCTGTTTACCAAAAACATCACCTCCAGC--ATCC-CTAGTATTGGAGGCACTGCCTGCCCAGTGACT--AAACGTTAAA-CGGCCGCGGTATTCTGACCGTGCAAAGGTAGCATAATCATTTGTTCTCTAAATAAGGACTTGTATGA
R-S_scrofa : ACAC-AAACCCCGCCTGTTTACCAAAAACATCACCTCTAGC--ATTA-CTAGTATTAGAGGCAATGCCTGCCCAGTGAC---ACCAGTTTAA-CGGCCGCGGTATTCTGACCGTGCAAAGGTAGCATAATCACTTGTTCTCCAAATAAGGACTTGTATGA
A-S_scrofa : ACAC-AAACCCCGCCTGTTTACCAAAAACATCACCTCTAGC--ATTA-CTAGTATTAGAGGCAATGCCTGCCCAGTGAC---ACCAGTTTAA-CGGCCGCGGTATTCTGACCGTGCAAAGGTAGCATAATCACTTGTTCTCCAAATAAGGACTTGTATGA
R-O_cuniculus : ACCC-TAACCCCGCCTGTTTACCAAAAACATCACCTCTAGC--ATTA-CTAGTATTAGAGGCACTGCCTGCCCAGTGAC---GTACGTTCAA-CGGCCGCGGTATCCTGACCGTGCAAAGGTAGCATAATCACTTGTTCCTTAATTGGGGACTAGCATGA
A-O_cuniculus : ACCC-TAACCCCGCCTGTTTACCAAAAACATCACCTCTAGC--ATTA-CTAGTATTAGAGGCACTGCCTGCCCAGTGAC---GTACGTTCAA-CGGCCGCGGTATCCTGACCGTGCAAAGGTAGCATAATCACTTGTTCCTTAATTGGGGACTAGCATGA
R-M_musculus : ACAA-GAACCCCGCCTGTTTACCAAAAACATCACCTCTAGC--ATTA-CAAGTATTAGAGGCACTGCCTGCCCAGTGAC---TAAAGTTTAA-CGGCCGCGGTATCCTGACCGTGCAAAGGTAGCATAATCACTTGTTCCTTAATTAGGGACTAGCATGA
A-M_musculus : ACAA-GAACCCCGCCTGTTTACCAAAAACATCACCTCTAGC--ATTA-CAAGTATTAGAGGCACTGCCTGCCCAGTGAC---TAAAGTTTAA-CGGCCGCGGTATCCTGACCGTGCAAAGGTAGCATAATCACTTGTTCCTTAATTAGGGACTAGCATGA
R-R_norvegicus : ACAC-GAGCCCCGCCTGTTTACCAAAAACATCACCTCTAGC--ATAA-CAAGTATTAGAGGCATTGCCTGCCCAGTGAC---TAAAGTTAAA-CGGCCGCGGTATCCTGACCGTGCAAAGGTAGCATAATCACTTGTTCCTTAATTAGGGACTAGAATGA
A-R_norvegicus : ACAC-GAACCCCGCCTGTTTACCAAAAACATCACCTCTAGC--ATAA-CAAGTATTAGAGGCATTGCCTGCCCAGTGAC---TAAAGTTAAA-CGGCCGCGGTATCCTGACCGTGCAAAGGTAGCATAATCACTTGTTCCTTAATTAGGGACTAGAATGA
R-M_coypus : ACAT-AAACCCCGCCTGTTTACCAAAAACATCACCTCTAGC--ATGA-TAAGTATTAGAGGCAATGCCTGCCCAGTGACAT-TTCTGTTCAA-CGGCCGCGGTATCCTGACCGTGCAAAGGTAGCATAATCACTTGTTCCTTAAATAGGGACTAGTATGA
A-M_coypus : ACAT-AAACCCCGCCTGTTTACCAAAAACATCACCTCTAGC--ATGA-CAAGTATTAGAGGCAATGCCTGCCCAGTGACAT-TTCTGTTCAA-CGGCCGCGGTATCCTGACCGTGCAAAGGTAGCATAATCACTTGTTCCTTAAATAGGGACTAGTATGA
R-A_platyrhynchos : AA-CAAGG-CCCGACTGTTTACCAAAAACATAGCCTTCAGCAAACAAACAAGTATTGAAGGTGATGCCTGCCCAGTGACCT---AGGTTAAA-CGGCCGCGGTATCCTAACCGTGCAAAGGTAGCGCAATCAATTGTCCCATAAATCGAGACTTGTATGA
A-A_platyrhynchos : AA-CAAGG-CCCGACTGTTTACCAAAAACATAGCCTTCAGCAAACAAACAAGTATTGAAGGTGATGCCTGCCCAGTGACCT---AGGTTAAA-CGGCCGCGGTATCCTAACCGTGCAAAGGTAGCGCAATCAATTGTCCCATAAATCGAGACTTGTATGA
R-G_gallus : AC-CAAAGACCCGACTGTTTCCCAAAAACATAGCCTTCAGCTAACAA-CAAGTATTGAAGGTGATGCCTGCCCAGTGACCCCCAAAGTTCAA-CGGCCGCGGTATCCTAACCGTGCGAAGGTAGCGCAATCAATTGTCCCGTAAATTGAGACTTGTATGA
A-G_gallus : AC-CAAAGACCCGACTGTTTCCCAAAAACATAGCCTTCAGCTAACAA-CAAGTATTGAAGGTGATGCCTGCCCAGTGACCCCCAAAGTTCAA-CGGCCGCGGTATCCTAACCGTGCGAAGGTAGCGCAATCAATTGTCCCGTAAATTGAGACTTGTATGA
 ACACAAAACCCCGCCTGTTTACCAAAAACATCACCTCCAGCWAATTAACTAGTATTGGAGGCACTGCCTGCCCAGTGACCTCAAAAGTTTAAACGGCCGCGGTATCCTGACCGTGCAAAGGTAGCATAATCATTTGTTCCCTAAATAGGGACTTGTATGA

 * 3540 * 3560 * 3580 * 3600 * 3620 * 3640 * 3660 * 3680
R-E_caballus : ATGGCCACACGAGGGTTTTACTGTCTCTTACTTCCAATCAGTGAAATTGACCTTCCCGTGAAGAGGCGGGAATGACTAAATAAGACGAGAAGACCCTATGGAGCTTTAATTAACTGAT--TCACAAA--AAACAACACACAAACCTTAACCTTCAG---G
A-E_caballus : ATGGCCACACGAGGGTTTTACTGTCTCTTACTTCCAATCAGTGAAATTGACCTTCCCGTGAAGAGGCGGGAATGACTAAATAAGACGAGAAGACCCTATGGAGCTTTAATTAACTGAT--TCACAAA--AAACAACACACAAACCT-AACCTTCAG---G
R-C_bactrianus : ACGGCCACACGAGGGTTCTACTGTCTCTTGCTTCCAATCAGTGAAATTGACCTCCCCGTGCAGAGGCGGGGATACACAAATAAGACGAGAAGACCCTATGGAGCTTTAATTAACTAAC--TCAAAGA--AAATA---TAAATAACC----GCCAAG---G
A-C_bactrianus : ACGGCCACACGAGGGTTCTACTGTCTCTTGCTTCCAATCAGTGAAATTGACCTCCCCGTGCAGAGGCGGGGATACACAAATAAGACGAGAAGACCCTATGGAGCTTTAATTAACTAAC--TCAAAGA--AAATA---TAAATAACC----GCCAAG---G
R-C_lupus_familiaris : ATGGCCACACGAGGGTTTAACTGTCTCTTACTCCCAATCAGTGAAATTGACCTTCCCGTGAAGAGGCGGGAATACCACAATAAGACGAGAAGACCCTATGGAGCTTTAATTAACTAAC--CCAAACT--TATGGATACTAG-ATAC---CTACAAG---G
A-C_lupus_familiaris : ATGGCCACACGAGGGTTTAACTGTCTCTTACTCCCAATCAGTGAAATTGACCTTCCCGTGAAGAGGCGGGAATACCACAATAAGACGAGAAGACCCTATGGAGCTTTAATTAACTAAC--CCAAACT--TATGGATACTAG-ATAC---CTACAAG---G
R-N_procyonoides : ATGGCTACACGAGGGCTTAACTGTCTCTTACTTCCAATCAGTGAAATTGACCTTCCCGTGAAGAGGCGGGAATACCACAATAAGACGAGAAGACCCTATGGAGCTTTAATTACTTAAC--CCAAATT--TATGG---CCAACACCCAC-CTACCAG---G
A-N_procyonoides : ATGGCTACACGAGGGCTTAACTGTCTCTTACTTCCAATCAGTGAAATTGACCTTCCCGTGAAGAGGCGGGAATACCACAATAAGACGAGAAGACCCTATGGAGCTTTAATTACTTAAC--CCAAATT--TATGG---CCAACACCCAC-CTACCAG---G
R-V_lagopus : ATGGCCACACGAGGGTTTAACTGTCTCTTACTTCCAATCCGTGAAATTGACCTTCCCGTGAAGAGGCGGGAATATCATAATAAGACGAGAAGACCCTATGGAGCTTTAATTAATTAGC--CCAAACC--CATGAAATCCAA-ACCC---CT-CCGG---G
A-V_lagopus : ATGGCCACACGAGGGTTTAACTGTCTCTTACTTCCAATCCGTGAAATTGACCTTCCCGTGAAGAGGCGGGAATATCATAATAAGACGAGAAGACCCTATGGAGCTTTAATTAATTAGC--CCAAACC--CATGAAATCCAA-ACCC---CT-CCGG---G
R-M_putorius : ATGGCCACACGAGGGTTTAACTGTCTCTTACTTCCAATCAGTGAAATTGACCTTCCCGTGAAGAGGCGGGAATATACCAATAAGACGAGAAGACCCTATGGAGCTTCAATTAACTAAC--CCACAAT--AACCAA--TCAATATGC---CAACCAG---G
A-M_putorius : ATGGCCACACGAGGGTTTAACTGTCTCTTACTTCCAATCAGTGAAATTGACCTTCCCGTGAAGAGGCGGGAATATACCAATAAGACGAGAAGACCCTATGGAGCTTCAATTAACTAAC--CCACAAT--AACCAA--TCAATATGC---CAACCAG---G
R-B_taurus : ATGGCCGCACGAGGGTTTTACTGTCTCTTACTTCCAATCAGTGAAATTGACCTTCCCGTGAAGAGGCGGGAATGCACAAATAAGACGAGAAGACCCTATGGAGCTTTAACTAACCAAC--CCAAAGA--GAATAGATTTAA---CC----ATTAAG---G
A-B_taurus : ATGGCCGCACGAGGGTTTTACTGTCTCTTACTTCCAATCAGTGAAATTGACCTTCCCGTGAAGAGGCGGGAATGCACAAATAAGACGAGAAGACCCTATGGAGCTTTAACTAACCAAC--CCAAAGA--GAATAGATTTAA---CC----ATTAAG---G
R-O_aries : ATGGCCACACGAGGGTTTTACTGTCTCTTACTTCCAATCAGTGAAATTGACCTCCCCGTGAAGAGGCGGGGATAAATCAACAAGACGAGAAGACCCTATGGAGCTTTAACTAAGTAAC--TCAAGGA--AAATAAATTCAA---CC----ACCAAG---G
A-O_aries : ATGGCCACACGAGGGTTTTACTGTCTCTTACTTCCAATCAGTGAAATTGACCTCCCCGTGAAGAGGCGGGGATAAATCAACAAGACGAGAAGACCCTATGGAGCTTTAACTAAGTAAC--TCAAGGA--AAATAAATTCAA---CC----ACCAAG---G
R-S_scrofa : ATGGCCACACGAGGGTTTTACTGTCTCTTACTTCCAATCAGTGAAATTGACCTTCCCGTGAAGAGGCGGGAATAAAAAAATAAGACGAGAAGACCCTATGGAGCTTTAATTAACTACT--CCAAAAGTTAAACA-ATTCAA---CC----ACAAAG---G
A-S_scrofa : ATGGCCACACGAGGGTTTTACTGTCTCTTACTTCCAATCAGTGAAATTGACCTTCCCGTGAAGAGGCGGGAATAAAAAAATAAGACGAGAAGACCCTATGGAGCTTTAATTAACTACT--CCAAAAGTTAAACA-ATTCAA---CC----ACAAAG---G
R-O_cuniculus : ATGGCAACACGAGGGTTAAACTGTCTCTTTCTTCCAATCAGTGAAATTGACCTCCCCGTGAAGAGGCGGGGATAAAATAATAAGACGAGAAGACCCTATGGAGCTTTAATTATTTAAC--CCAACACTTCCTTTATTCTACTCTAC----AATGAGCCTA
A-O_cuniculus : ATGGCAACACGAGGGTTAAACTGTCTCTTTCTTCCAATCAGTGAAATTGACCTCCCCGTGAAGAGGCGGGGATAAAATAATAAGACGAGAAGACCCTATGGAGCTTTAATTATTTAAC--CCAACACTTCCTTTATTCTACTCTAC----AATGAGCCTA
R-M_musculus : ACGGCTAAACGAGGGTCCAACTGTCTCTTATCTTTAATCAGTGAAATTGACCTTTCAGTGAAGAGGCTGAAATATAATAATAAGACGAGAAGACCCTATGGAGCTTAAATTATATAAC--TTATCT---ATTTAATTTATTAAACC----TA-ATG---G
A-M_musculus : ACGGCTAAACGAGGGTCCAACTGTCTCTTATCTTTAATCAGTGAAATTGACCTTTCAGTGAAGAGGCTGAAATATAATAATAAGACGAGAAGACCCTATGGAGCTTAAATTATATAAC--TTATCT---ATTTAATTTATTAAACC----TA-ATG---G
R-R_norvegicus : ATGGCTAAACGAGGGTTCAACTGTCTCTTACTTTCAATCAGTGAAATTGACCTTCCAGTGAAGAGGCTGGAATCTCCCAATAAGACGAGAAGACCCTATGGAGCTTCAATTTACTAGT--TCAACTT--ATATAA---AAACAACC----TA-ATG---G
A-R_norvegicus : ATGGCTAAACGAGGGTTCAACTGTCTCTTACTTTCAATCAGTGAAATTGACCTTCCAGTGAAGAGGCTGGAATCTCCCAATAAGACGAGAAGACCCTATGGAGCTTCAATTTACTAGT--TCAACTT--ATATAA---AAACAACC----TA-ATG---G
R-M_coypus : ACGGCTTAACGAGGGTTTAACTGTCTCTTTCTTGTAGTCAATGAAATTGACCTTCCCGTGAAGAGGCGGGAATTAAACAATAAGACGAGAAGACCCTATGGAGCTTTAATTCATTAAT--TCAATT---TTACAA--CACTAAATC----AACAAG---A
A-M_coypus : ACGGCTTAACGAGGGTTTAACTGTCTCTTTCTTGTAGTCAATGAAATTGACCTTCCCGTGAAGAGGCGGGAATTAAACAATAAGACGAGAAGACCCTATGGAGCTTTAATTCATTAAT--TCAATT---TTACAA--CACTAAATC----AACAAG---A
R-A_platyrhynchos : ATGGCTAAACGAGGTCTTAACTGTCTCTCACGGATAATCAGTGAAATTGATCTCCCCGTGCAAAAGCGGGGATGTGAACATAAGACGAGAAGACCCTGTGGAACTTAAA--AATCAACGGCCACCGCG-AACCTAAGACTAAACCC-----ACCGG---G
A-A_platyrhynchos : ATGGCTAAACGAGGTCTTAACTGTCTCTCACGGATAATCAGTGAAATTGATCTCCCCGTGCAAAAGCGGGGATGTGAACATAAGACGAGAAGACCCTGTGGAACTTAAA--AATCAACGGCCACCGCG-AACCTAAGACTAAACCC-----ACCGG---G
R-G_gallus : ATGGCTAAACGAGGTCTTAACTGTCTCCTGTAGGTAATCTATGAAATTAGTATTCCCGTGCAAAAACGAGAATGTGAACATAAGACGAGAAGACCCTGTGGAACTTTAA--AATCA-CGACCACCTTACAACCTTACAC-AGCCCC-----ACTGG---G
A-G_gallus : ATGGCTAAACGAGGTCTTAACTGTCTCCTGTAGGTAATCTATGAAATTAGTATTCCCGTGCAAAAACGAGAATGTGAACATAAGACGAGAAGACCCTGTGGAACTTTAA--AATCA-CGACCACCTTACAACCTTACAC-AGCCCC-----ACTGG---G
 ATGGCCACACGAGGGTTTAACTGTCTCTTACTTCCAATCAGTGAAATTGACCTTCCCGTGAAGAGGCGGGAATATAAMAATAAGACGAGAAGACCCTATGGAGCTTTAATTAACTAACGRCCAAATTTTAAAYAAATYCAAAACCCAMACAACAAGCCTG

 * 3700 * 3720 * 3740 * 3760 * 3780 * 3800 * 3820 * 3840
R-E_caballus : GACAACAAAACTTT-TGAT--TGAATCAGCAA-TTTCGGTTGGGGTGACCTCGGAGAACAAAACAACCTCCGAGT-----GATT-------TAAATCCAGACT-AACCAGTCAAAA----TATATAA---TCACTTATT-GATCCAA---ACCATTGATC
A-E_caballus : GACAACAAAACTTT-TGAT--TGAATCAGCAA-TTTCGGTTGGGGTGACCTCGGAGAACAAAACAACCTCCGAGT-----GATT-------TAAATCCAGACT-AACCAGTCAAAA----TATATAA---TCACTTATT-GATCCAA---ACCATTGATC
R-C_bactrianus : GATAATAATCTTCTACC----TGAGTTAGCAA-TTTCGGTTGGGGTGACCTCGGAGAACAGAAAAACCTCCGAGT-----GACT-------AAAATCTAGACT-TACCAGTCAAAA----TGTAGTG---TCACTTATT-GATCCAA--AATATTTGATC
A-C_bactrianus : GATAATAATCTTCTACC----TGAGTTAGCAA-TTTCGGTTGGGGTGACCTCGGAGAACAGAAAAACCTCCGAGT-----GACT-------AAAATCTAGACT-TACCAGTCAAAA----TGTAGTG---TCACTTATT-GATCCAA--AATATTTGATC
R-C_lupus_familiaris : CATAACATAACACCATTATTATGAGTTAGCAA-TTTAGGTTGGGGTGACCTCGGAATATAAAAAAACTCCCGAGT-----GATT-------AAAATTTAGACC-CACAAGTCAAAA----TACAACA---TCACTTATT-GATCCAAT-AATTTTTGATC
A-C_lupus_familiaris : CATAACATAACACCATTATTATGAGTTAGCAA-TTTAGGTTGGGGTGACCTCGGAATATAAAAAAACTCCCGAGT-----GATT-------AAAATTTAGACC-CACAAGTCAAAA----TACAACA---TCACTTATT-GATCCAAT-AATTTTTGATC
R-N_procyonoides : CATAAAATACTACCATTATTATGGGTTAACAA-TTTAGGTTGGGGTGACCTCGGAACATAAAAAAACTTCCGAGT-----GGTT-------AAAATTTAGACC-TACCAGTCAAAA----TGTAGTA---TCACTTATT-GATCCAAT-TATTTTTGATC
A-N_procyonoides : CATAAAATACTACCATTATTATGGGTTAACAA-TTTAGGTTGGGGTGACCTCGGAACATAAAAAAACTTCCGAGT-----GGTT-------AAAATTTAGACC-TACCAGTCAAAA----TGTAGTA---TCACTTATT-GATCCAAT-TATTTTTGATC
R-V_lagopus : AATAACTTACTATCATCGTTATGGGCTAACAA-TTTAGGTTGGGGTGACCTCGGAATATAAAAAAACTCCCGAGT-----GATT-------AAAATTTAGACC-TACCAGTCAAAA----TGTATCA---TCACTTATT-GATCCAAT-AATCATTGATC
A-V_lagopus : AATAACTTACTATCATCGTTATGGGCTAACAA-TTTAGGTTGGGGTGACCTCGGAATATAAAAAAACTCCCGAGT-----GATT-------AAAATTTAGACC-TACCAGTCAAAA----TGTATCA---TCACTTATT-GATCCAAT-AATCATTGATC
R-M_putorius : CCTAATATAATCTTAT--TTCTGGGTTAGCAA-TTTAGGTTGGGGTGACCTCGGAGAATAAAATAACCTCCGAGT-----GATT-------TAATCTGAGACA-AACCAGTCGAAG----CGTCCTA---TCATTAATT-GATCCAAT-AAT--TTGATC
A-M_putorius : CCTAATATAATCTTAT--TTCTGGGTTAGCAA-TTTAGGTTGGGGTGACCTCGGAGAATAAAATAACCTCCGAGT-----GATT-------TAATCTGAGACA-AACCAGTCGAAG----CGTCCTA---TCATTAATT-GATCCAAT-AAT--TTGATC
R-B_taurus : AATAA--CAACAAT--CTCCATGAGTTGGTAG-TTTCGGTTGGGGTGACCTCGGAGAATAAAAAATCCTCCGAGC-----GATTTT-----AAAGACTAGACC-CACAAGTCAAA----TCACTCTA---TCGCTCATT-GATCCAAA-AAC--TTGATC
A-B_taurus : AATAA--CAACAAT--CTCCATGAGTTGGTAG-TTTCGGTTGGGGTGACCTCGGAGAATAAAAAATCCTCCGAGC-----GATTTT-----AAAGACTAGACC-CACAAGTCAAA----TCACTCTA---TCGCTCATT-GATCCAAA-AAC--TTGATC
R-O_aries : GATAA--CAACACT--CCTTATGAGTTAACAG-TTTCGGTTGGGGTGACCTCGGAGAACAGAAAATCCTCCGAGC-----GATTTT-----AAAGACTAGACT-AACAAGTCAAA----CCAAACCA---TCGCTTATT-GATCCAAA-AAC--TTGATC
A-O_aries : GATAA--CAACACT--CCTTATGAGTTAACAG-TTTCGGTTGGGGTGACCTCGGAGAACAGAAAATCCTCCGAGC-----GATTTT-----AAAGACTAGACT-AACAAGTCAAA----CCAAACCA---TCGCTTATT-GATCCAAA-AAC--TTGATC
R-S_scrofa : GATAAAACATAACT--TAACATGGACTAGCAA-TTTCGGTTGGGGTGACCTCGGAGTACAAAAAACCCTCCGAGT-----GATTTT-----AA--TCTAGACA-AACCAGTCAAAATAACCATAACA---TCACTTATT-GATCCAAA-ATT--TTGATC
A-S_scrofa : GATAAAACATAACT--TAACATGGACTAGCAA-TTTCGGTTGGGGTGACCTCGGAGTACAAAAAACCCTCCGAGT-----GATTTT-----AA--TCTAGACA-AACCAGTCAAAATAACCATAACA---TCACTTATT-GATCCAAA-ATT--TTGATC
R-O_cuniculus : ACCAAGGAAATCCC-------TGGGTTAAAAA-TTTTGGTTGGGGTGACCTCGGAGTATAAATCAACCTCCGAAT-----GATT-------TTAGCCTAGACCCAACAAGTCAAAGCAATTATA--A---TCATAAATT-GACCCAAATAAT--TTGATC
A-O_cuniculus : ACCAAGGAAATCCC-------TGGGTTAAAAA-TTTTGGTTGGGGTGACCTCGGAGTATAAATCAACCTCCGAAT-----GATT-------TTAGCCTAGACCCAACAAGTCAAAGCAATTATA--A---TCATAAATT-GACCCAAATAAT--TTGATC
R-M_musculus : CCCAA---AA-ACTATAG-TATAAGTTTGAAA-TTTCGGTTGGGGTGACCTCGGAGAATAAAAAATCCTCCGAAT-----GATT-------ATAACCTAGACT-TACAAGTCAAAG---TAAAATCAACATATCTTATT-GACCCAGATATATTTTGATC
A-M_musculus : CCCAA---AA-ACTATAG-TATAAGTTTGAAA-TTTCGGTTGGGGTGACCTCGGAGAATAAAAAATCCTCCGAAT-----GATT-------ATAACCTAGACT-TACAAGTCAAAG---TAAAATCAACATATCTTATT-GACCCAGATATATTTTGATC
R-R_norvegicus : GCTAA---AACAAAATAAATATGAACTAAAAAATTTCGGTTGGGGTGACCTCGGAGAATAAAAAATCCTCCGAAT-----GATT-------TTAACCTAGACT-CACAAGTCAAAG---TAATACTAA--TATCTTATT-GACCCAATTAT----TGATC
A-R_norvegicus : GCTAA---AACAAAATAAATATGAACTAAAAAATTTCGGTTGGGGTGACCTCGGAGAATAAAAAATCCTCCGAAT-----GATT-------TTAACCTAGACT-CACAAGTCAAAG---TAATACTAA--TATCTTATT-GACCCAATTAT----TGATC
R-M_coypus : ACTAA---AA--CTTAAATTATGAATTAATAA-TTTTGGTTGGGGTGACCTCGGAGTAAAAAGAAACCTCCGAAT-----GATA-------TTAACCTAGACA-CACAAGTCAAAG---ATATATCA---TAA---ATT-GACCCAGAACTTATCTGATC
A-M_coypus : ACTAA---AA--CTTAAATTATGAATTAATAA-TTTTGGTTGGGGTGACCTCGGAGTAAAAAGAAACCTCCGAAT-----GATA-------TTAACCTAGACA-CACAAGTCAAAG---ATACATCA---TAA---ATT-GACCCAGAACTTATCTGATC
R-A_platyrhynchos : GCTACAGACATCGCAGAGCA-TG-GCCGATATTTTTCGGTTGGGGCGACCTTGGAGAACAACAGATCCTCCAAAAACA-AGACCACACCTCTTTACTTAGAGC-CACCCCTCAAAG------TGCTAA---TAGTGACCAGACCCAAT--ATAATTGATT
A-A_platyrhynchos : GCTACAGACATCGCAGAGCA-TG-GCCGATATTTTTCGGTTGGGGCGACCTTGGAGAACAACAGATCCTCCAAAAACA-AGACCACACCTCTTTACTTAGAGC-CACCCCTCAAAG------TGCTAA---TAGTGACCAGACCCAAT--ATAATTGATT
R-G_gallus : TCCACCCACA-CATAAACCCCTG-GTCGACATTTTTCGGTTGGGGCGACCTTGGAGAAAAAAAAATCCTCCAAACCCACAGACCACAACTCTTCACTAAGACC-AACTCCTCAAAG------TACCAA---CAGTAACCAGACCCAAT--ATAATTGAGC
A-G_gallus : TCCACCCACA-CATAAACCCCTG-GTCGACATTTTTCGGTTGGGGCGACCTTGGAGAAAAAAAAATCCTCCAAACCCACAGACCACAACTCTTCACTAAGACC-AACTCCTCAAAG------TACCAA---CAGTAACCAGACCCAAT--ATAATTGAGC
 GATAACAAAATACTATAATTATGAGTTAACAATTTTCGGTTGGGGTGACCTCGGAGAATAAAAAAACCTCCGAGTMCACAGATTTTAMCTCAAAACCTAGACCCAACMAGTCAAAGYAATTATACCAACATCACTTATTAGATCCAATTAATATTTGATC

 * 3860 * 3880 * 3900 * 3920 * 3940 * 3960 * 3980 * 4000
R-E_caballus : AACGGAACAAGTTACCCTAGGGATAACAGCGCAATCCTATTCCAGAGTCCATATCGACAATTAGGGTTTACGACCTCGATGTTGGATCAAGACATCCTAATGGTGCAACCGCTATTAAGGGTTCGTTTGTTCAACGATTAA-AGTCTTACGTGATCTGAG
A-E_caballus : AACGGAACAAGTTACCCTAGGGATAACAGCGCAATCCTATTCCAGAGTCCATATCGACAATTAGGGTTTACGACCTCGATGTTGGATCAAGACATCCTAATGGTGCAACCGCTATTAAGGGTTCGTTTGTTCAACGATTAA-AGTCTTACGTGATCTGAG
R-C_bactrianus : AACGGAACAAGTTACCCTAGGGATAACAGCGCAATCCTATTCAAGAGTTCATATCGACAAT-AGGGTTTACGACCTCGATGTTGGATCAGGACACCCCAATGGTGCAGCCGCTATTAAAGGTTCGTTTGTTCAACGATTAA-AGTCCTACGTGATCTGAG
A-C_bactrianus : AACGGAACAAGTTACCCTAGGGATAACAGCGCAATCCTATTCAAGAGTTCATATCGACAAT-AGGGTTTACGACCTCGATGTTGGATCAGGACACCCCAATGGTGCAGCCGCTATTAAAGGTTCGTTTGTTCAACGATTAA-AGTCCTACGTGATCTGAG
R-C_lupus_familiaris : AACGGAACAAGTTACCCTAGGGATAACAGCGCAATCCTATTCAAGAGTCCATATCGACAAT-AGGGTTTACGACCTCGATGTTGGATCAGGACATCCTAATGGTGCAGCAGCTATTAAGGGTTCGTTTGTTCAACGATTAA-AGTCCTACGTGATCTGAG
A-C_lupus_familiaris : AACGGAACAAGTTACCCTAGGGATAACAGCGCAATCCTATTCAAGAGTCCATATCGACAAT-AGGGTTTACGACCTCGATGTTGGATCAGGACATCCTAATGGTGCAGCAGCTATTAAGGGTTCGTTTGTTCAACGATTAA-AGTCCTACGTGATCTGAG
R-N_procyonoides : AACGGAACAAGTTACCCTAGGGATAACAGCGCAATCCTATCCAAGAGTCCATATCGACGAT-AGGGTTTACGACCTCGATGTTGGATCAGGACATCCTAATGGTGCAGCAGCTATTAAGGGTTCGTTTGTTCAACGATTAA-AGTCCTACGTGATCTGAG
A-N_procyonoides : AACGGAACAAGTTACCCTAGGGATAACAGCGCAATCCTATCCAAGAGTCCATATCGACGAT-AGGGTTTACGACCTCGATGTTGGATCAGGACATCCTAATGGTGCAGCAGCTATTAAGGGTTCGTTTGTTCAACGATTAA-AGTCCTACGTGATCTGAG
R-V_lagopus : AACGGAACAAGTTACCCTAGGGATAACAGCGCAATCCTATTTAAGAGTTCATATCGACAAT-AGGGTTTACGACCTCGATGTTGGATCAGGACATCCTAATGGTGCAGCAGCTATTAAGGGTTCGTTTGTTCAACGATTAA-AGTCCTACGTGATCTGAG
A-V_lagopus : AACGGAACAAGTTACCCTAGGGATAACAGCGCAATCCTATTTAAGAGTTCATATCGACAAT-AGGGTTTACGACCTCGATGTTGGATCAGGACATCCTAATGGTGCAGCAGCTATTAAGGGTTCGTTTGTTCAACGATTAA-AGTCCTACGTGATCTGAG
R-M_putorius : AACGGAACAAGTTACCCTAGGGATAACAGCGCAATCCTATTTGAGAGTCCATATCAACAAT-AGGGTTTACGACCTCGATGTTGGATCAGGACATCCTAATGGTGCAGCAGCTATTAATGGTTCGTTTGTTCAACGATTAA-AGTCCTACGTGATCTGAG
A-M_putorius : AACGGAACAAGTTACCCTAGGGATAACAGCGCAATCCTATTTGAGAGTCCATATCAACAAT-AGGGTTTACGACCTCGATGTTGGATCAGGACATCCTAATGGTGCAGCAGCTATTAATGGTTCGTTTGTTCAACGATTAA-AGTCCTACGTGATCTGAG
R-B_taurus : AACGGAACAAGTTACCCTAGGGATAACAGCGCAATCCTATTCAAGAGTCCATATCGACAAT-AGGGTTTACGACCTCGATGTTGGATCAGGACATCCTGATGGTGCAACCGCTATCAAAGGTTCGTTTGTTCAACGATTAA-AGTCCTACGTGATCTGAG
A-B_taurus : AACGGAACAAGTTACCCTAGGGATAACAGCGCAATCCTATTCAAGAGTCCATATCGACAAT-AGGGTTTACGACCTCGATGTTGGATCAGGACATCCTGATGGTGCAACCGCTATCAAAGGTTCGTTTGTTCAACGATTAA-AGTCCTACGTGATCTGAG
R-O_aries : AACGGAACAAGTTACCCTAGGGATAACAGCGCAATCCTATTCAAGAGTCCATATCGACAAT-AGGGTTTACGACCTCGATGTTGGATCAGGACACCCCGATGGTGCAACCGCTATCAAAGGTTCGTTTGTTCAACGATTAA-AGTCCTACGTGATCTGAG
A-O_aries : AACGGAACAAGTTACCCTAGGGATAACAGCGCAATCCTATTCAAGAGTCCATATCGACAAT-AGGGTTTACGACCTCGATGTTGGATCAGGACATCCCGATGGTGCAACCGCTATCAAAGGTTCGTTTGTTCAACGATTAA-AGTCCTACGTGATCTGAG
R-S_scrofa : AACGGAACAAGTTACCCTAGGGATAACAGCGCAATCCTATTCTAGAGTTCCTATCGACAAT-AGGGTTTACGACCTCGATGTTGGATCAGGACACCCAAATGGTGCAACCGCTATTAAAGGTTCGTTTGTTCAACGATTAA-AGTCCTACGTGATCTGAG
A-S_scrofa : AACGGAACAAGTTACCCTAGGGATAACAGCGCAATCCTATTCTAGAGTTCCTATCGACAAT-AGGGTTTACGACCTCGATGTTGGATCAGGACACCCAAATGGTGCAACCGCTATTAAAGGTTCGTTTGTTCAACGATTAA-AGTCCTACGTGATCTGAG
R-O_cuniculus : AACGGAACAAGTTACCCTAGGGATAACAGCGCAATCCTATTTTAGAGTCCCTATCGACAAT-AGGGTTTACGACCTCGATGTTGGATCAGGACATCCCAATGGTGTAGCCGCTATTAAAGGTTCGTTTGTTCAACGATTAA-AGTCCTACGTGATCTGAG
A-O_cuniculus : AACGGAACAAGTTACCCTAGGGATAACAGCGCAATCCTATTTTAGAGTCCCTATCGACAAT-AGGGTTTACGACCTCGATGTTGGATCAGGACATCCCAATGGTGTAGCCGCTATTAAAGGTTCGTTTGTTCAACGATTAA-AGTCCTACGTGATCTGAG
R-M_musculus : AACGGACCAAGTTACCCTAGGGATAACAGCGCAATCCTATTTAAGAGTTCATATCGACAATTAGGGTTTACGACCTCGATGTTGGATCAGGACATCCCAATGGTGTAGAAGCTATTAATGGTTCGTTTGTTCAACGATTAA-AGTCCTACGTGATCTGAG
A-M_musculus : AACGGACCAAGTTACCCTAGGGATAACAGCGCAATCCTATTTAAGAGTTCATATCGACAATTAGGGTTTACGACCTCGATGTTGGATCAGGACATCCCAATGGTGTAGAAGCTATTAATGGTTCGTTTGTTCAACGATTAA-AGTCCTACGTGATCTGAG
R-R_norvegicus : AACGGACCAAGTTACCCTAGGGATAACAGCGCAATCCTATTTAAGAGTTCATATCGACAATTAGGGTTTACGACCTCGATGTTGGATCAGGACATCCCAATGGTGCAGAAGCTATTAATGGTTCGTTTGTTCAACGATTAA-AGTCCTACGTGATCTGAG
A-R_norvegicus : AACGGACCAAGTTACCCTAGGGATAACAGCGCAATCCTATTTAAGAGTTCATATCGACAATTAGGGTTTACGACCTCGATGTTGGATCAGGACATCCCAATGGTGCAGAAGCTATTAATGGTTCGTTTGTTCAACGATTAA-AGTCCTACGTGATCTGAG
R-M_coypus : AACGGACCAAGTTACCCTAGGGATAACAGCGCAATCCTATTTCAGAGTCCATATCGACAAT-AGGGTTTACGACCTCGATGTTGGATCAGGACTTCCAAATGGTGCAGCCGCTATTAAAGGTTCGTTTGTTCAACGATTAA-AGTCCTACGTGATCTGAG
A-M_coypus : AACGGACCAAGTTACCCTAGGGATAACAGCGCAATCCTATTTCAGAGTCCATATCGACAAT-AGGGTTTACGACCTCGATGTTGGATCAGGACTTCCAAATGGTGCAGCCGCTATTAAAGGTTCGTTTGTTCAACGATTAA-AGTCCTACGTGATCTGAG
R-A_platyrhynchos : AATGGACCAAGCTACCCCAGGGATAACAGCGCAATCCCCCTCAAGAGCCCCTATCGACAGG-GGGGTTTACGACCTCGATGTTGGATCAGGACATCCTAATGGTGCAGCCGCTATTAAGGGTTCGTTTGTTCAACGATTAATAGTCCTACGTGATCTGAG
A-A_platyrhynchos : AATGGACCAAGCTACCCCAGGGATAACAGCGCAATCCCCCTCAAGAGCCCCTATCGACAGG-GGGGTTTACGACCTCGATGTTGGATCAGGACATCCTAATGGTGCAGCCGCTATTAAGGGTTCGTTTGTTCAACGATTAATAGTCCTACGTGATCTGAG
R-G_gallus : AATGGACCAAGCTACCCCAGGGATAACAGCGCAATCTCCTCCAAGAGCCCATATCGACAAG-GAGGTTTACGACCTCGATGTTGGATCAGGACAACCTAATGGTGCAACCGCTATTAAGGGTTCGTTTGTTCAACGATTAACAGTCCTACGTGATCTGAG
A-G_gallus : AATGGACCAAGCTACCCCAGGGATAACAGCGCAATCTCCTCCAAGAGCCCATATCGACAAG-GAGGTTTACGACCTCGATGTTGGATCAGGACAACCTAATGGTGCAACCGCTATTAAGGGTTCGTTTGTTCAACGATTAACAGTCCTACGTGATCTGAG
 AACGGAACAAGTTACCCTAGGGATAACAGCGCAATCCTATTCAAGAGTCCATATCGACAATTAGGGTTTACGACCTCGATGTTGGATCAGGACATCCTAATGGTGCAGCCGCTATTAARGGTTCGTTTGTTCAACGATTAAYAGTCCTACGTGATCTGAG

 * 4020 * 4040 * 4060 * 4080 * 4100 * 4120 * 4140 * 4160
R-E_caballus : TTCAGACCGGAGTAATCCAGGTCGGTTTCTATCTATTC-TAT-ACTTTTCCCAGTACGAAAGGACAAGAAAAGTAGGGCCCACTTTACAAGAA-GCGCCCTCAAA--CTAATAGATGACAT-AATCTAAATC---TAACTAATTTATAACTTC--TAC--
A-E_caballus : TTCAGACCGGAGTAATCCAGGTCGGTTTCTATCTATTC-TAT-ACTTTTCCCAGTACGAAAGGACAAGAAAAGTAGGGCCTACTTTACAAGAA-GCGCCCTCAAA--CTAATAGATGACAT-AATCTAAATC---TAACTAATTTATAACTTC--TAC--
R-C_bactrianus : TTCAGACCGGAGTAATCCAGGTCGGTTTCTATCTATTA--TTAATTTCTCCCAGTACGAAAGGACAAGAGAAATAAGGCCTACTCTAAA-GGA-GCGCCTTAGAA--CTAACTAATGATAT-AATCTTAACT---TACCTAGTTCAAAAAAAA--TAC--
A-C_bactrianus : TTCAGACCGGAGTAATCCAGGTCGGTTTCTATCTATTA--TTAATTTCTCCCAGTACGAAAGGACAAGAGAAATAAGGCCTACTCTAAA-GGA-GCGCCTTAGAA--CTAACTAATGATAT-AATCTTAACT---TACCTAGTTCAAAAAAAA-ATAC--
R-C_lupus_familiaris : TTCAGACCGGAGTAATCCAGGTCGGTTTCTATCTATTA-TACAACCTCCCCCAGTACGAAAGGACAAGGGATGTAAGGCCTACCTCACA-GAG-GCGCCTTAAAA--CTAATAGATGAAGTCAA-CTCAATC---TAACCAGTTTATCTCCTC-ATA---
A-C_lupus_familiaris : TTCAGACCGGAGTAATCCAGGTCGGTTTCTATCTATTA-TACAACCTCCCCCAGTACGAAAGGACAAGGGATGTAAGGCCTACCTCACA-GAG-GCGCCTTAAAA--CTAATAGATGAAGTCAA-CTCAATC---TAACCAGTTTATCTCCTC-ATA---
R-N_procyonoides : TTCAGACCGGAGTAATCCAGGTCGGTTTCTATCTATTA-AACAATTTCTCCCAGTACGAAAGGACAAGAGAAATAAGGCCCACCTCACA-AAA-GCGCCTTAAGA--CTAATAGATGAAACTGAACTTAATC---TAGCCAGTTTATTTCCCC-ATA---
A-N_procyonoides : TTCAGACCGGAGTAATCCAGGTCGGTTTCTATCTATTA-AACAATTTCTCCCAGTACGAAAGGACAAGAGAAATAAGGCCCACCTCACA-AAA-GCGCCTTAAGA--CTAATAGATGAAACTGAACTTAATC---TAGCCAGTTTATTTCCCC-ATA---
R-V_lagopus : TTCAGACCGGAGTAATCCAGGTCGGTTTCTATCTATTA-AATAATTTCTCCCAGTACGAAAGGACAAGAGAAATAGGGCCTACCTTACA-GAG-GCGCCTTAAAA--CTAATAGATGAAATTAAGCTTAATC---TAGTCAGTTTACTCCTTT-ATA---
A-V_lagopus : TTCAGACCGGAGTAATCCAGGTCGGTTTCTATCTATTA-AATAATTTCTCCCAGTACGAAAGGACAAGAGAAATAGGGCCTACCTTACA-GAG-GCGCCTTAAAA--CTAATAGATGAAATTAAGCTTAATC---TAGTCAGTTTACTCCTTT-ATA---
R-M_putorius : TTCAGACCGGAGCAATCCAGGTCGGTTTCTATCTATTATAATTACTTCTCCCAGTACGAAAGGACAAGAGAAGTAGGGCCTATTCTACA-GGA-AAGCCTTAGGA--CTAATAGATGATAT-AATCTCAATC---TAACCAGTCCACTCCCCCCATA---
A-M_putorius : TTCAGACCGGAGCAATCCAGGTCGGTTTCTATCTATTATAATTACTTCTCCCAGTACGAAAGGACAAGAGAAGTAGGGCCTATTCTACA-GGA-AAGCCTTAGGA--CTAATAGATGATAT-AATCTCAATC---TAACCAGTCCACTCCCCCCATA---
R-B_taurus : TTCAGACCGGAGTAATCCAGGTCGGTTTCTATCTATTA--CGTATTTCTCCCAGTACGAAAGGACAAGAGAAATAAGGCCAACTTTAAATCAA-GCGCCTTA-AG--ACAACCAATGATAA-CATCTCAAC----TGACA---ACACAA--AA--CCC--
A-B_taurus : TTCAGACCGGAGTAATCCAGGTCGGTTTCTATCTATTA--CGTATTTCTCCCAGTACGAAAGGACAAGAGAAATAAGGCCAACTTTAAATCAA-GCGCCTTA-AG--ACAACCAATGATAA-CATCTCAAC----TGACA---ACACAA--AA--CCC--
R-O_aries : TTCAGACCGGAGTAATCCAGGTCGGTTTCTATCTGTTA--TGTATTTCTCCCAGTACGAAAGGACAAGAGAAATAAGGCCAACTTTAA-CAAA-GCGCCTTA-AA--CCAATTAATGACTT-TATCTTAAT----TAATT---TCACAACAAA--ACC--
A-O_aries : TTCAGACCGGAGTAATCCAGGTCGGTTTCTATCTGTTA--TGTATTTCTCCCAGTACGAAAGGACAAGAGAAATAAGGCCAACTTTAA-CAAA-GCGCCTTA-AA--CCAATTAATGACTT-TATCTTAAT----TAATT---TCACAACAAA--ACC--
R-S_scrofa : TTCAGACCGGAGCAATCCAGGTCGGTTTCTATCTATTA--TAAATTTCTCCCAGTACGAAAGGACAAGAGAAATGGGACCAACCTCA--CAAACGCGTCTCAGAG--ATAATTAATGATAT-AATCTTAACC---TAATTAACTCATAATAAA--TCC--
A-S_scrofa : TTCAGACCGGAGCAATCCAGGTCGGTTTCTATCTATTA--TAAATTTCTCCCAGTACGAAAGGACAAGAGAAATGGGACCAACCTCA--CAAACGCGTCTCAGAG--ATAATTAATGATAT-AATCTTAACC---TAATTAACTCATAATAAA--TCC--
R-O_cuniculus : TTCAGACCGGAGAAATCCAGGTCGGTTTCTATCTATTA--AGTATTTCTCCCAGTACGAAAGGACAAGAGAAATAGAGCCTACTGCACC-ACA-GAGCTCTAAGT--CCAAAAGATGAAAT-AATCTTAATC---TAGTACACTTACCCAACA--TAG--
A-O_cuniculus : TTCAGACCGGAGAAATCCAGGTCGGTTTCTATCTATTA--AGTATTTCTCCCAGTACGAAAGGACAAGAGAAATAGAGCCTACTGCACC-ACA-GAGCTCTAAGT--CCAAAAGATGAAAT-AATCTTAATC---TAGTACACTTACCCAACA--TAG--
R-M_musculus : TTCAGACCGGAGCAATCCAGGTCGGTTTCTATCTATTT--ACGATTTCTCCCAGTACGAAAGGACAAGAGAAATAGAGCCACCTTACAAA-TAAGCGCTCTCAAC--TTAATTTATGAATAAAATCTAAATA---AAATATATACGTACACCCTCT----
A-M_musculus : TTCAGACCGGAGCAATCCAGGTCGGTTTCTATCTATTT--ACGATTTCTCCCAGTACGAAAGGACAAGAGAAATAGAGCCACCTTACAAA-TAAGCGCTCTCAAC--TTAATTTATGAATAAAATCTAAATA---AAATATATACGTACACCCTCT----
R-R_norvegicus : TTCAGACCGGAGCAATCCAGGTCGGTTTCTATCTATTT--ACAATTTCTCCCAGTACGAAAGGACAAGAGAAATGGAGCCTCCTTACCA--TAAGTGCTCCCAAC--C-AATTTATGAAAAAAATCTCAATA---AAGTATATATGTACAATAAATT---
A-R_norvegicus : TTCAGACCGGAGCAATCCAGGTCGGTTTCTATCTATTT--ACAATTTCTCCCAGTACGAAAGGACAAGAGAAATGGAGCCTCCTTACCA--TAAGTGCTCCCAAC--C-AATTTATGAAAAAAATCTCAATA---AAGTATATATGTACAATAAAT----
R-M_coypus : TTCAGACCGGAGTAATCCAGGTCGGTTTCTATCTATTA--AAAATTTCTCCTAGTACGAAAGGACAAGAGAAATAAGGCCAATGAATCAATTATGCCTTAGTAGC--T-AAAAGATGA-TATCATATCAACC---CAGCAGCTACTAAAAACACAC----
A-M_coypus : TTCAGACCGGAGTAATCCAGGTCGGTTTCTATCTATTA--AAAATTTCTCCTAGTACGAAAGGACAAGAGAAATAAGGCCAATGAATCAATTATGCCTTAGTAGC--T-AAAAGATGA-TATCATATCAACC---CAGCAGCTACTAAAAACACAC----
R-A_platyrhynchos : TTCAGACCGGAGCAATCCAGGTCGGTTTCTATCTATGA-AC-TACTCTCCCCAGTACGAAAGGACCGGGAAAGTAAGGCCAATACTAC--AAGCACGCCTT--CCCTCTAAGTAGTGAAACCAA-CTCAACTATGAAGAGGACTCCCCCCCACCACCCCA
A-A_platyrhynchos : TTCAGACCGGAGCAATCCAGGTCGGTTTCTATCTATGA-AC-TACTCTCCCCAGTACGAAAGGACCGGGAAAGTAAGGCCAATACTAC--AAGCACGCCTT--CCCTCTAAGTAGTGAAACCAA-CTCAACTATGAAGAGGACTCCCCCCCACCACCCCA
R-G_gallus : TTCAGACCGGAGCAATCCAGGTCGGTTTCTATCTATGG-ACAC--TCCTCCTAGTACGAAAGGACCGGAGAAGTGGGGTCAATACCACT-GAGCACACCCCAACCTTCTAAGCAATGAATACAA-CTCAACTGCCAAGA--ACCCCTCCCCCACACCCGA
A-G_gallus : TTCAGACCGGAGCAATCCAGGTCGGTTTCTATCTATGA-ACACACTCCTCCTAGTACGAAAGGACCGGAGAAGTGGGGTCAATACCACT-GAGCACACCCCAACCTTCTAAGCAATGAATACAA-CTCAACTGCCAAGA--ACCCCCCCCCCACACCC-A
 TTCAGACCGGAGTAATCCAGGTCGGTTTCTATCTATTATAAYAATTTCTCCCAGTACGAAAGGACAAGAGAAATAGGGCCAACTTTACAAGAACGCGCCTTAAAAYTCTAATAGATGAAATYAATCTCAATCRYSTARCAAATTCAYACCMCACATACCA

 * 4180 * 4200 * 4220 * 4240 * 4260 * 4280 * 4300 * 4320
R-E_caballus : CGCCCTAGAACAGGG---CTCGTTAGGGTGGCAGAGCCCGGAAATTGCATAAAACTTAAACCTTTACACTCAGAGGTTCAACTCCTCTCCCTAAC-A------ACATG---------------------TTCATAATTAACGTCCTCCTCCTAATTGTCC
A-E_caballus : CGCCCTAGAACAGGG---CTCGTTAGGGTGGCAGAGCCCGGAAATTGCATAAAACTTAAACCTTTACACTCAGAGGTTCAACTCCTCTCCCTAAC-A------ACATG---------------------TTCATAATTAACGTTCTCCTCCTAATTGTCC
R-C_bactrianus : AGCCCTAGACAAGGG---CTTATTAGGGTGGCAGAGACCGGTAATTGCATAAAACTTAAGATTTTAGACCCAGAGGTTCAACCCCTCTCCCTAAT-AT-----GTATG---------------------TTCATAGTAAATACCCTCACACTTATCGTCC
A-C_bactrianus : AGCCCTAGACAAGGG---CTTATTAGGGTGGCAGAGACCGGTAATTGCATAAAACTTAAGATTTTAGACCCAGAGGTTCAACCCCTCTCCCTAAT-AT-----GTATG---------------------TTCATAGTAAATACCCTCACACTTATCGTCC
R-C_lupus_familiaris : AGCCCGAGAAAAGGGG-CTTTGTTAGGGTG-CAGGGCCCGGTAACTGCGTAAAACTTAAACCTTTACTATCAGAGGTTCAATTCCTCTCCCTAAC-A------AAATG---------------------TTCTTTATCAACATTATCTCTCTTATTATCC
A-C_lupus_familiaris : AGCCCGAGAAAAGGGG-CTTTGTTAGGGTGGCAGAGCCCGGTAACTGCGTAAAACTTAAACCTTTACTATCAGAGGTTCAATTCCTCTCCCTAAC-A------AAATG---------------------TTCTTTATCAACATTATCTCTCTTATTATCC
R-N_procyonoides : AGCCCGAGAAAAGGG--CTTTGTTAGGGTGGCAGAGCCCGGTAATTGCGTAAAACTTAAACCTTTATCCCCAGAGGTTCAACTCCTCTCCCTAAC-A------ACATG---------------------TTCCTCATCAACATCATCTCTCTAATCGTCC
A-N_procyonoides : AGCCCGAGAAAAGGG--CTTTGTTAGGGTGGCAGAGCCCGGTAATTGCGTAAAACTTAAACCTTTATCCCCAGAGGTTCAACTCCTCTCCCTAAC-A------ACATG---------------------TTCCTCATCAACATCATCTCTCTAATCGTCC
R-V_lagopus : AACCCAAGAAAAGGGGGCTTTGTTAGGGTGGCAGAGCCCGGCAATTGCGTAAGACTTAAACCTTTATCCTCAGAGGTTCAACTCCTCTCCCTAAC-A------ACATG---------------------TTCTTTGTCAACATTATCTCCCTTATCGTCC
A-V_lagopus : AACCCAAGAAAAGGGGGCTTTGTTAGGGTGGCAGAGCCCGGCAATTGCGTAAGACTTAAACCTTTATCCTCAGAGGTTCAACTCCTCTCCCTAAC-A------ACATG---------------------TTCTTTGTCAACATTATCTCCCTTATCGTCC
R-M_putorius : ACCCTAGAAATAGGG---TTTGTTAGGGTGGCAGAGCCCAGTAATTGCGTAAAACTTAAACCTTTATTCCCAGAGGTTCAAATCCTCTCCCTAAC-A------TCATG---------------------CTTATAATTAACATTATTTCACTTATTGTAC
A-M_putorius : ACCCTAGAAATAGGG---TTTGTTAGGGTGGCAGAGCCCAGTAATTGCGTAAAACTTAAACCTTTATTCCCAGAGGTTCAAATCCTCTCCCTAAC-A------TCATG---------------------CTTATAATTAACATTATTTCACTTATTGTAC
R-B_taurus : TGCCCTAGAACAGGG--CTTAGTTAAGGTGGCAGAGCCCGGTAATTGCATAAAACTTAAACTTTTATATCCAGAGATTCAAATCCTCTCCTTAAC-A------AAATG---------------------TTCATAATTAACATCTTAATACTAATTATTC
A-B_taurus : TGCCCTAGAACAGGG--CTTAGTTAAGGTGGCAGAGCCCGGTAATTGCATAAAACTTAAACTTTTATATCCAGAGATTCAAATCCTCTCCTTAAC-A------AAATG---------------------TTCATAATTAACATCTTAATACTAATTATTC
R-O_aries : TGCCCTAGAAAAGGG--CCCAGTTAAGGTGGCAGAGCCCGGTAATTGCGTAAAACTTAAACCTTTATACTCAGAGATTCAAATCCTCTCCTTAAC-A------AAATG---------------------TTTATAATCAACGTTCTAACACTCATCATTC
A-O_aries : TGCCCTAGAAAAGGG--CCCAGTTAAGGTGGCAGAGCCCGGTAATTGCGTAAAACTTAAACCTTTATACTCAGAGATTCAAATCCTCTCCTTAAC-A------AAATG---------------------TTTATAATCAACGTTCTAACACTCATTATTC
R-S_scrofa : AGCCCTAGAACAGGG--CACA-TTAGGGTGGCAGAGACCGGTAATTGCGTAAAACTTAAACCTTTATTACCAGAGGTTCAACTCCTCTCCCTAAT-A------GCATG---------------------TTCATAATTAACATTCTAAGCCTAATCATTC
A-S_scrofa : AGCCCTAGAACAGGG--CACA-TTAGGGTGGCAGAGACCGGTAATTGCGTAAAACTTAAACCTTTATTACCAGAGGTTCAACTCCTCTCCCTAAT-A------GCATG---------------------TTCATAATTAACATTCTAAGCCTAATCATTC
R-O_cuniculus : AGCCCTAGACCAGAG---CTAGTTAAGGTGGCAGAGCCCGGTAATTGCGTAAAACTTAAAACTTTATAACCAGAGGTTCAACTCCTCTCCTTAAC-A------ATATG---------------------TTCCTAATTAATACACTCCTTTTAATCCTAC
A-O_cuniculus : AGCCCTAGACCAGAG---CTAGTTAAGGTGGCAGAGCCCGGTAATTGCGTAAAACTTAAAACTTTATAACCAGAGGTTCAACTCCTCTCCTTAAC-A------ATATG---------------------TTCCTAATTAATACACTCCTTTTAATCCTAC
R-M_musculus : -AACCTAGAGAAGG-----TTATTAGGGTGGCAGAGCCAGGAAATTGCGTAAGACTTAAAACCTTGTTCCCAGAGGTTCAAATCCTCTCCCTAAT-A--------GTG---------------------TTCTTTATTAATATCCTAACACTCCTCGTCC
A-M_musculus : -AACCTAGAGAAGG-----TTATTAGGGTGGCAGAGCCAGGAAATTGCGTAAGACTTAAAACCTTGTTCCCAGAGGTTCAAATCCTCTCCCTAAT-A--------GTG---------------------TTCTTTATTAATATCCTAACACTCCTCGTCC
R-R_norvegicus : AAACCTAGCCCAGG-----TTATTAGGGTGGCAGAGCCAGGTAATTGCGTAAGACTTAAAACCTTGTTCCCAGAGGTTCAAATCCTCTCCCTAAT-A--------GTG---------------------TACTTTATTAATATCCTAACACTCCTAATCC
A-R_norvegicus : AAACCTAGCCCAGG-----TTATTAGGGTGGCAGAGCCAGGTAATTGCGTAAGACTTAAAACCTTGTTCCCAGAGGTTCAAATCCTCTCCCTAAT-A--------GTG---------------------TACTTTATTAATATCCTAACACTCCTAATCC
R-M_coypus : GACCCGAGAGAAGGG---TTTGTTAAGGTGGCAGAGCCCGGTAATTGCATAAAACTTAAGACTTTACTGTCAGAGGTTCAACTCCTCTCCTTAAC-AC-----TCATG---------------------TACATAATCAATTTCCTTTTACTAGTAGTTC
A-M_coypus : GACCCGAGAGAAGGG---TTTGTTAAGGTGGCAGAGCCCGGTAATTGCATAAAACTTAAGACTTTACTGTCAGAGGTTCAACTCCTCTCCTTAAC-AC-----TCATG---------------------TACATAATCAATTTCCTTTTACTAGTAGTTC
R-A_platyrhynchos : A-TCCTAGAAAAGGA--TC-AGCTAGAGTGGCAGAGCCCGGCAAATGCAAAAGGCTTAAGCCCTT-TACCCAGAGGTTCAAATCCTCTCCCTAGCTAC-----ACATGCCACAAACAACAATAGTAAGCTACCTCATTATAGCCCTGCTATACATCATCC
A-A_platyrhynchos : A-TCCTAGAAAAGGA--TC-AGCTAGAGTGGCAGAGCCCGGCAAATGCAAAAGGCTTAAGCCCTT-TACCCAGAGGTTCAAATCCTCTCCCTAGCTAC-----ACATGCCACAAACAACAATAGTAAGCTACCTCATTATAGCCCTGCTATACATCATCC
R-G_gallus : ACTCCTAGAAAAGGA--TCCAGCTAGCGTGGCAGAGCTCGGCAAATGCAAAAGGCTTAAGCCCTT-TATCCAGAGGTTCAAATCCTCTCCCTAGCTACCCCGGACATGACCCTGCCCACCCTAACAAACCTTCTAATCATAACCTTATCCTATATTCTCC
A-G_gallus : ACTCCTAGAAAAGGA--TCCAGCTAGCGTGGCAGAGCTCGGCAAATGCAAAAGGCTTAAGCCCTT-TATCCAGAGGTTCAAATCCTCTCCCTAGCTACCCCGGACATGACCCTGCCCACCCTAACAAACCTTCTAATCATAACCTTATCCTATATTCTCC
 AGCCCTAGAAAAGGGGGCYTTGTTAGGGTGGCAGAGCCCGGTAATTGCGTAAAACTTAAACCTTTATACCCAGAGGTTCAAMTCCTCTCCCTAACTACCCCGGACATGMCMCWRMCMACMMTARYAARCTTCATAATTAACATCCTMWCACTAATCGTCC

 * 4340 * 4360 * 4380 * 4400 * 4420 * 4440 * 4460 * 4480
R-E_caballus : CAATCTTGCTCGCCGTAGCATTCCTCACACTAGTTGAACGAAAAGTCTTAGGCTATATGCAACTTCGCAAAGGACCCAACATCGTAGGCCCCTATGGCCTACTACAACCTATTGCCGATGCCCTCAAACTATTTATCAAAGAGCCACTACAACCACTAAC
A-E_caballus : CAATCTTGCTCGCCGTAGCATTCCTCACACTAGTTGAACGAAAAGTCTTAGGCTATATGCAACTTCGCAAAGGACCCAACATCGTAGGCCCCTACGGCCTACTACAACCTATTGCCGATGCCCTCAAACTATTTATCAAAGAGCCACTACAACCACTAAC
R-C_bactrianus : CCATCCTCCTAGCAATAGCATTCCTCACCCTTGTCGAACGAAAAATCCTAGGCTACATGCAACTCCGAAAGGGCCCTAACGTTGTGGGTCCCTATGGCCTGCTACAACCAATTGCAGATGCTATCAAATTATTCACCAAAGAGCCGCTACGACCTGCCAC
A-C_bactrianus : CCATCCTCCTAGCAATAGCATTCCTCACCCTTGTCGAACGAAAAATCCTAGGCTACATGCAACTCCGAAAGGGCCCTAACGTTGTGGGTCCCTATGGCCTGCTACAACCAATTGCAGATGCTATCAAATTATTCACCAAAGAGCCGCTACGACCTGCCAC
R-C_lupus_familiaris : CAATCCTTCTTGCCGTAGCCTTCCTCACCCTCGTTGAACGAAAAGTCTTAGGCTATATACAACTTCGAAAAGGACCTAATATTGTAGGCCCCTACGGCCTCCTTCAACCAATCGCAGACGCAGTAAAACTCTTCACAAAAGAACCTCTACGACCACTTAC
A-C_lupus_familiaris : CAATCCTTCTTGCCGTAGCCTTCCTCACCCTCGTTGAACGAAAAGTCTTAGGCTATATACAACTTCGAAAAGGACCTAATATTGTAGGCCCCTACGGCCTCCTTCAACCAATCGCAGACGCAGTAAAACTCTTCACAAAAGAACCTCTACGACCACTTAC
R-N_procyonoides : CAATTCTCCTTGCCGTAGCCTTCCTGACCCTTGTTGAACGAAAAATCCTAGGCTACATACAATTTCGAAAAGGACCAAACGTCGTAGGTCCATATGGCCTCCTCCAACCCATCGCCGACGCAGTGAAGCTATTTACAAAAGAACCCCTACGACCCCTTAC
A-N_procyonoides : CAATTCTCCTTGCCGTAGCCTTCCTGACCCTTGTTGAACGAAAAATCCTAGGCTACATACAATTTCGAAAAGGACCAAACGTCGTAGGTCCATATGGCCTCCTCCAACCCATCGCCGACGCAGTGAAGCTATTTACAAAAGAACCCCTACGACCCCTTAC
R-V_lagopus : CAATTCTTCTCGCCGTAGCCTTCCTTACTCTTGTTGAACGTAAAGTTCTAGGCTACATACAACTCCGAAAAGGGCCCAATATTGTAGGACCCTATGGCCTCCTTCAACCAATCGCCGATGCTGTAAAACTCTTTACAAAAGAACCTCTACGTCCCCTTAC
A-V_lagopus : CAATTCTTCTCGCCGTAGCCTTCCTTACTCTTGTTGAACGTAAAGTTCTAGGCTACATACAACTCCGAAAAGGGCCCAATATTGTAGGACCCTATGGCCTCCTTCAACCAATCGCCGATGCTGTAAAACTCTTTACAAAAGAACCTCTACGTCCCCTTAC
R-M_putorius : CAATCCTACTCGCCGTAGCTTTCCTGACATTAGTAGAACGAAAAGTCTTAGGATACATACAACTTCGCAAAGGCCCAAACATTGTAGGACCCTACGGCCTCCTACAACCAATTGCAGATGCTGTAAAACTTTTCACAAAAGAGCCATTACGACCCCTAAC
A-M_putorius : CAATCCTACTCGCCGTAGCTTTCCTGACATTAGTAGAACGAAAAGTCTTAGGATACATACAACTTCGCAAAGGCCCAAACATTGTAGGACCCTACGGCCTCCTACAACCAATTGCAGATGCTGTAAAACTTTTCACAAAAGAGCCATTACGACCCCTAAC
R-B_taurus : CCATCCTATTGGCCGTAGCATTCCTTACGTTAGTGGAACGAAAAGTTCTAGGCTATATACAACTCCGAAAAGGTCCAAATGTCGTAGGTCCATATGGCCTACTTCAACCCATCGCCGATGCAATCAAACTTTTCATTAAAGAACCACTACGACCCGCTAC
A-B_taurus : CCATCCTATTGGCCGTAGCATTCCTTACGTTAGTGGAACGAAAAGTTCTAGGCTATATACAACTCCGAAAAGGTCCAAATGTCGTAGGTCCATATGGCCTACTTCAACCCATCGCCGATGCAATCAAACTTTTCATTAAAGAACCACTACGACCCGCTAC
R-O_aries : CTATTCTCCTAGCTGTAGCTTTTCTTACACTAGTTGAACGAAAAGTTCTAGGTTATATACAATTTCGAAAAGGCCCAAACGTTGTAGGGCCATATGGCTTACTTCAACCCATCGCCGACGCAATTAAACTCTTCATCAAAGAACCCCTACGACCCGCCAC
A-O_aries : CTATTCTCCTAGCTGTAGCTTTTCTTACACTAGTTGAACGAAAAGTTCTAGGTTATATACAATTTCGAAAAGGCCCAAACGTTGTAGGGCCATATGGCTTACTTCAACCCATCGCCGACGCAATTAAACTCTTCATCAAAGAACCCCTACGACCCGCCAC
R-S_scrofa : CTATCCTACTGGCCGTAGCATTCCTCACCCTAGTAGAACGAAAAGTACTAGGTTATATGCAACTACGAAAAGGACCCAACGTTGTAGGCCCCTACGGCCTACTCCAACCCATCGCCGATGCCCTAAAACTATTCACCAAAGAACCCCTACGACCAGCCAC
A-S_scrofa : CTATCCTACTGGCCGTAGCATTCCTCACCCTAGTAGAACGAAAAGTACTAGGTTATATGCAACTACGAAAAGGACCCAACGTTGTAGGCCCCTACGGCCTACTCCAACCCATCGCCGATGCCCTAAAACTATTCACCAAAGAACCCCTACGACCAGCCAC
R-O_cuniculus : CTGTACTTTTAGCCATAGCATTCCTCACCTTAGTCGAACGAAAAATCTTAGGGTACATACAACTACGTAAAGGCCCAAACATTGTAGGACCCTATGGCCTCCTCCAACCAATCGCAGACGCTATTAAGCTATTCACTAAAGAACCCCTACGACCCCTAAC
A-O_cuniculus : CTGTACTTTTAGCCATAGCATTCCTCACCTTAGTCGAACGAAAAATCTTAGGGTACATACAACTACGTAAAGGCCCAAACATTGTAGGACCCTATGGCCTCCTCCAACCAATCGCAGACGCTATTAAGCTATTCACTAAAGAACCCCTACGACCCCTAAC
R-M_musculus : CCATTCTAATCGCCATAGCCTTCCTAACATTAGTAGAACGCAAAATCTTAGGGTACATACAACTACGAAAAGGCCCTAACATTGTTGGTCCATACGGCATTTTACAACCATTTGCAGACGCCATAAAATTATTTATAAAAGAACCAATACGCCCTTTAAC
A-M_musculus : CCATTCTAATCGCCATAGCCTTCCTAACATTAGTAGAACGCAAAATCTTAGGGTACATACAACTACGAAAAGGCCCTAACATTGTTGGTCCATACGGCATTTTACAACCATTTGCAGACGCCATAAAATTATTTATAAAAGAACCAATACGCCCTTTAAC
R-R_norvegicus : CAATCTTAATTGCCATGGCCTTCCTCACCCTAGTAGAACGGAAAATCCTAGGCTACATACAACTACGCAAAGGCCCCAACATCGTAGGCCCATATGGTATTCTACAACCATTTGCAGATGCCATAAAACTATTCATAAAAGAACCCATACGCCCCCTAAC
A-R_norvegicus : CAATCTTAATTGCCATGGCCTTCCTCACCCTAGTAGAACGGAAAATCCTAGGCTACATACAACTACGCAAAGGCCCCAACATCGTAGGCCCATATGGTATTCTACAACCATTTGCAGATGCCATAAAACTATTCATAAAAGAACCCATACGCCCCCTAAC
R-M_coypus : CCATTCTTCTAGCAATAGCATTCTTAACCTTAGTAGAACGAAAAATCTTAGGATACATACAATTACGAAAAGGCCCAAACATTGTAGGCCCATACGGCATTCTACAACCAATAGCAGACGCATTAAAATTATTCATCAAAGAACCTTTACGCCCATCTAC
A-M_coypus : CCATTCTTCTAGCAATAGCATTCTTAACCTTAGTAGAACGAAAAATCTTAGGATACATACAATTACGAAAAGGCCCAAACATTGTAGGCCCATACGGCATTCTACAACCAATAGCAGACGCATTAAAATTATTCATCAAAGAACCTTTACGCCCATCTAC
R-A_platyrhynchos : CAATCTTAATTGCCGTGGCTTTCTTGACTCTAGTCGAACGAAAAATTCTAAGCTACATGCAATCCCGTAAAGGCCCCAACATCGTGGGGCCTTTTGGCCTGCTCCAACCCATTGCAGACGGAATCAAACTATTCATTAAAGAGCCCATTCGACCTTCCAC
A-A_platyrhynchos : CAATCTTAATTGCCGTGGCTTTCTTGACTCTAGTCGAACGAAAAATTCTAAGCTACATGCAATCCCGTAAAGGCCCCAACATCGTGGGGCCTTTTGGCCTGCTCCAACCCATTGCAGACGGAATCAAACTATTCATTAAAGAGCCCATTCGACCTTCCAC
R-G_gallus : CCATCCTAATCGCCGTGGCCTTCTTAACACTTGTAGAACGAAAAATCCTAAGCTACATGCAGGCCCGAAAGGGCCCAAACATTGTGGGCCCTTTTGGTCTACTCCAACCCGTTGCAGACGGGGTAAAACTATTCATTAAAGAGCCAATCCGACCATCTAC
A-G_gallus : CCATCCTAATCGCCGTGGCCTTCTTAACACTTGTAGAACGAAAAATCCTAAGCTACATGCAGGCCCGAAAGGGCCCAAACATTGTGGGCCCTTTTGGTCTACTCCAACCCGTTGCAGACGGGGTAAAACTATTCATTAAAGAGCCAATCCGACCATCTAC
 CAATCCTACTCGCCGTAGCMTTCCTCACCCTAGTAGAACGAAAAATCCTAGGCTACATACAACTHCGAAAAGGCCCAAACATTGTAGGCCCCTATGGCCTMCTACAACCAATYGCAGACGCAATAAAACTATTCATAAAAGAACCCCTACGACCCCTTAC

 * 4500 * 4520 * 4540 * 4560 * 4580 * 4600 * 4620 * 4640
R-E_caballus : ATCATCGACATCCATATTCATCATCGCACCAATCCTAGCCCTAACCCTGGCCTTAACCATATGAATCCCTCTGCCCATACCATACCCACTAATCAACATAAACCTAGGAATTCTATTCATACTAGCCATGTCCAGCCTAGCTGTCTACTCAATCCTTTGA
A-E_caballus : ATCATCGACATCCATATTCATCATCGCACCAATCCTAGCCCTAACCCTGGCCTTAACCATATGAATCCCTCTGCCCATACCATACCCACTAATCAACATAAACCTAGGAATTCTATTCATACTAGCCATGTCCAGCCTAGCTGTCTACTCAATCCTTTGA
R-C_bactrianus : CTCCTCTGTTACTATGTTCATTATCGCCCCCGTTCTAGCCCTAACCCTAGCCCTAACCATATGAATTCCACTCCCAATACCACACCCCCTCATCAATATAAACCTAGGCGTGCTATTTTTACTAGCAATATCTAGCCTAGCCGTTTACTCTATCCTATGA
A-C_bactrianus : CTCCTCTGTTACTATGTTCATTATCGCCCCCGTTCTAGCCCTAACCCTAGCCCTAACCATATGAATTCCACTCCCAATACCACACCCCCTCATCAATATAAACCTAGGCGTGCTATTTTTACTAGCAATATCTAGCCTAGCCGTTTACTCTATCCTATGA
R-C_lupus_familiaris : ATCCTCTATATCAATATTCATCCTAGCCCCCATTCTAGCTCTATCACTAGCCCTAACTATGTGAATTCCCCTCCCAATACCCTACCCACTCATTAATATAAACTTGGGAGTCCTATTCATACTAGCAATATCAAGCCTCGCCGTGTACTCCATCCTCTGA
A-C_lupus_familiaris : ATCCTCTATATCAATATTCATCCTAGCCCCCATTCTAGCTCTATCACTAGCCCTAACTATGTGAATTCCCCTCCCAATACCCTACCCACTCATTAATATAAACTTGGGAGTCCTATTCATACTAGCAATATCAAGCCTCGCCGTGTACTCCATCCTCTGA
R-N_procyonoides : ATCCTCCATATCAATGTTTATCCTAGCACCTATCCTAGCCTTATCCCTGGCCCTAACCATATGAATTCCACTCCCCATACCATATCCACTAATTAACATAAACTTAGGAGTCCTATTCATACTAGCAATATCAAGTCTAGCCGTATACTCCATTCTTTGA
A-N_procyonoides : ATCCTCCATATCAATGTTTATCCTAGCACCTATCCTAGCCTTATCCCTGGCCCTAACCATATGAATTCCACTCCCCATACCATATCCACTAATTAACATAAACTTAGGAGTCCTATTCATACTAGCAATATCAAGTCTAGCCGTATACTCCATTCTTTGA
R-V_lagopus : ATCATCGATATCAATATTTATTCTAGCACCTATCCTAGCCCTATCACTGGCCCTAACCATATGAATCCCACTCCCTATGCCCTACCCACTCATTAACATAAATTTAGGAGTGCTGTTTATATTAGCTATGTCTAGCCTCGCCGTATATTCTATTCTCTGA
A-V_lagopus : ATCATCGATATCAATATTTATTCTAGCACCTATCCTAGCCCTATCACTGGCCCTAACCATATGAATCCCACTCCCTATGCCCTACCCACTCATTAACATAAATTTAGGAGTGCTGTTTATATTAGCTATGTCTAGCCTCGCCGTATATTCTATTCTCTGA
R-M_putorius : ATCATCTATTACCATGTTCGTCATAGCTCCTATCCTAGCCCTTACACTAGCCCTAACAATATGAATCCCACTACCAATGCCCTATCCCCTTATCAATATGAACTTAGGGATCCTATTTATACTAGCAATATCAAGCCTAGCTGTTTACTCTATCCTATGA
A-M_putorius : ATCATCTATTACCATGTTCGTCATAGCTCCTATCCTAGCCCTTACACTAGCCCTAACAATATGAATCCCACTACCAATGCCCTATCCCCTTATCAATATGAACTTAGGGATCCTATTTATACTAGCAATATCAAGCCTAGCTGTTTACTCTATCCTATGA
R-B_taurus : ATCTTCAGCCTCAATATTTATCCTAGCACCTATCATAGCTTTAGGCCTAGCCTTAACCATGTGAATTCCCCTACCAATACCCTATCCTCTTATCAACATAAACCTAGGAGTCCTATTTATACTAGCCATATCAAGCCTAGCCGTATACTCCATTCTCTGA
A-B_taurus : ATCTTCAGCCTCAATATTTATCCTAGCACCTATCATAGCTTTAGGCCTAGCCTTAACCATGTGAATTCCCCTACCAATACCCTATCCTCTTATCAACATAAACCTAGGAGTCCTATTTATACTAGCCATATCAAGCCTAGCCGTATACTCCATTCTCTGA
R-O_aries : ATCCTCAATCTCAATATTCATTCTAGCCCCCATCCTAGCACTAACCCTAGCCTTAACTATATGAATCCCCCTACCCATACCCTATCCCCTCATCAATATAAACTTAGGAGTCCTCTTCATATTAGCCATATCAAGCCTAGCCGTATACTCAATCCTCTGA
A-O_aries : ATCCTCAATCTCAATATTCATTCTAGCTCCCATCCTAGCACTAACCCTAGCCTTAACTATATGAATCCCCCTACCCATACCCTATCCCCTCATCAATATAAACTTAGGAGTCCTCTTCATATTAGCCATATCAAGCCTAGCCGTATACTCAATCCTCTGA
R-S_scrofa : ATCCTCAATCTCCATGTTCATTATTGCACCAATCCTAGCCTTATCCCTAGCACTAACAATATGAGTTCCACTACCAATACCCTACCCTCTAATCAACATAAATCTAGGAGTACTATTCATGCTAGCCATGTCAAGCCTAGCAGTCTATTCTATCCTATGA
A-S_scrofa : ATCCTCAATCTCCATGTTCATTATTGCACCAATCCTAGCCTTATCCCTAGCACTAACAATATGAGTTCCACTACCAATACCCTACCCTCTAATCAACATAAATCTAGGAGTACTATTCATGCTAGCCATGTCAAGCCTAGCAGTCTATTCTATCCTATGA
R-O_cuniculus : ATCCTCTCCGCTACTCTTTATTATCGCCCCAACCCTAGCTCTAACTCTCGCACTATCAATGTGACTTCCTATCCCCATACCTTACCCACTAGTCAACCTAAATATAGGCATTCTATTCATCCTAGCAACCTCCAGCTTAGCCGTTTACTCAATCCTATGA
A-O_cuniculus : ATCCTCTCCGCTACTCTTTATTATCGCCCCAACCCTAGCTCTAACTCTCGCACTATCAATGTGACTTCCTATCCCCATACCTTACCCACTAGTCAACCTAAATATAGGCATTCTATTCATCCTAGCAACCTCCAGCTTAGCCGTTTACTCAATCCTATGA
R-M_musculus : AACCTCTATATCCTTATTTATTATTGCACCTACCCTATCACTCACACTAGCATTAAGTCTATGAGTTCCCCTACCAATACCACACCCATTAATTAATTTAAACCTAGGGATTTTATTTATTTTAGCAACATCTAGCCTATCAGTTTACTCCATTCTATGA
A-M_musculus : AACCTCTATATCCTTATTTATTATTGCACCTACCCTATCACTCACACTAGCATTAAGTCTATGAGTTCCCCTACCAATACCACACCCATTAATTAATTTAAACCTAGGGATTTTATTTATTTTAGCAACATCTAGCCTATCAGTTTACTCCATTCTATGA
R-R_norvegicus : CACCTCAATATCACTATTTATTATCGCCCCAACCCTCTCCCTTACACTAGCTCTAAGCCTATGAATCCCCTTACCAATACCTCACCCCCTTATCAACCTCAACCTAGGCATACTATTTATTCTAGCCACATCAAGTCTTTCAGTCTACTCCATTCTATGA
A-R_norvegicus : CACCTCAATATCACTATTTATCATCGCCCCAACCCTCTCCCTTACACTAGCTCTAAGCCTATGAATCCCCTTACCAATACCTCACCCCCTTATCAACCTCAACCTAGGCATACTATTTATTCTAGCCACATCAAGTCTTTCAGTCTACTCCATTCTATGA
R-M_coypus : ATCATCAATATCTCTTTTTATCATTGCCCCTTCCCTAGCCTTAACCCTCGCTATCTCAATATGAATTCCCATTCCAATGCCATATTCACTTATTAATCTAAATCTAGGGGCTTTATTCATCTTAGCTACATCAAGTTTAGCCGTATATTCCATCTTATGA
A-M_coypus : ATCATCAATATCTCTTTTTATCATTGCCCCTTCCCTAGCCTTAACCCTCGCTATCTCAATATGAATTCCCATTCCAATGCCATATTCACTTATTAATCTAAATCTAGGGGCTTTATTCATCTTAGCTACATCAAGTTTAGCCGTATATTCCATCTTATGA
R-A_platyrhynchos : CTCCTCACCGCTCCTCTTCATCATAATGCCCATACTAGCCCTCCTCCTAGCCCTCACCGCCTGAGTGCCCCTCCCCCTCCCGTTCTCACTAGTAGACCTGAACCTCGGGGTCCTCTTTATAGTAGCCATATCAAGCTTAGCCGTCTACTCAATCCTATGA
A-A_platyrhynchos : CTCCTCACCGCTCCTCTTCATCATAATGCCCATACTAGCCCTCCTCCTAGCCCTCACCGCCTGAGTGCCCCTCCCCCTCCCGTTCTCACTAGTAGACCTGAACCTCGGGGTCCTCTTTATAGTAGCCATATCAAGCTTAGCCGTCTACTCAATCCTATGA
R-G_gallus : CTCCTCCCCCTTCCTCTTCATTATTACTCCAATCCTAGCACTACTCCTAGCCCTGACTATTTGAGTCCCCCTCCCACTACCATTCCCCCTTGCAGACCTCAACCTAGGACTACTATTTCTCCTAGCCATATCAAGCCTAACTGTCTACTCTCTACTCTGA
A-G_gallus : CTCCTCCCCCTTCCTCTTCATTATTACTCCAATCCTAGCACTACTCCTAGCCCTGACTATTTGAGTCCCCCTCCCACTACCATTCCCCCTTGCAGACCTCAACCTAGGACTACTATTTCTCCTAGCCATATCAAGCCTAACTGTCTACTCTCTACTCTGA
 ATCCTCAATATCAATATTCATYATAGCACCTATCCTAGCCCTAACCCTAGCCCTAACCATATGAATTCCCCTCCCAATACCMTACCCACTAATCAACATAAACCTAGGAGTCCTATTTATACTAGCCATATCAAGCCTAGCCGTMTACTCCATCCTATGA

 * 4660 * 4680 * 4700 * 4720 * 4740 * 4760 * 4780 * 4800
R-E_caballus : TCAGGATGGGCCTCAAACTCAAAATACGCCCTAATTGGAGCTCTACGAGCAGTAGCACAAACCATCTCATACGAAGTAACTCTAGCAATCATCCTACTCTCAGTCCTCCTAATAAGCGGATCATTCACATTATCAACACTTATTATTACCCAAGAATACC
A-E_caballus : TCAGGATGGGCCTCAAACTCAAAATACGCCCTAATTGGAGCTCTACGAGCAGTAGCACAAACCATCTCATACGAAGTAACTCTAGCAATCATCCTACTCTCAGTCCTCCTAATAAGCGGATCATTCACATTATCAACACTTATTATTACCCAAGAATACC
R-C_bactrianus : TCCGGCTGAGCCTCCAACTCAAAATACGCATTAATCGGCGCCCTTCGGGCCGTCGCCCAGACCATCTCATATGAAGTCACATTGGCTATCATCTTACTCTCCGTGCTTCTAATAAATGGATCCTTTACCCTATCAACACTTATCACAACACAAGAACACA
A-C_bactrianus : TCCGGCTGAGCCTCCAACTCAAAATACGCATTAATCGGCGCCCTTCGGGCCGTCGCCCAGACCATCTCATATGAAGTCACATTGGCTATCATCTTACTCTCCGTGCTTCTAATAAATGGATCCTTTACCCTATCAACACTTATCACAACACAAGAACACA
R-C_lupus_familiaris : TCAGGATGAGCCTCAAACTCCAAATACGCCCTAATCGGAGCCCTTCGAGCAGTAGCTCAAACAATCTCATATGAAGTAACGCTAGCAATTATTCTTCTATCAGTCCTCCTAATAAACGGGTCATTTACACTATCCACGCTAATTATTACCCAAGAACATA
A-C_lupus_familiaris : TCAGGATGAGCCTCAAACTCCAAATACGCCCTAATCGGAGCCCTTCGAGCAGTAGCTCAAACAATCTCATATGAAGTAACGCTAGCAATTATTCTTCTATCAGTCCTCCTAATAAACGGGTCATTTACACTATCCACGCTAATTATTACCCAAGAACATA
R-N_procyonoides : TCAGGATGAGCCTCAAACTCCAAATACGCCCTAATCGGGGCCCTACGGGCCGTAGCCCAGACAATCTCATATGAAGTTACACTGGCAATTATCCTATTATCCGTCCTATTGATAAACGGATCATTCACATTATCCACACTCATTATCACCCAAGAACATA
A-N_procyonoides : TCAGGATGAGCCTCAAACTCCAAATACGCCCTAATCGGGGCCCTACGGGCCGTAGCCCAGACAATCTCATATGAAGTTACACTGGCAATTATCCTATTATCCGTCCTATTGATAAACGGATCATTCACATTATCCACACTCATTATCACCCAAGAACATA
R-V_lagopus : TCAGGATGGGCCTCAAACTCCAAATACGCCCTAATTGGGGCCCTGCGAGCCGTAGCTCAGACAATCTCATATGAGGTCACGCTAGCAATCATCCTCCTCTCCATTTTATTAATAAACGGATCATTCACATTATCTACACTTATCATTACCCAAGAGCACA
A-V_lagopus : TCAGGATGGGCCTCAAACTCCAAATACGCCCTAATTGGGGCCCTGCGAGCCGTAGCTCAGACAATCTCATATGAGGTCACGCTAGCAATCATCCTCCTCTCCATTTTATTAATAAACGGATCATTCACATTATCTACACTTATCATTACCCAAGAGCACA
R-M_putorius : TCCGGGTGGGCCTCAAACTCAAAATACGCCCTAATCGGAGCCCTACGGGCCGTAGCCCAAACAATCTCCTACGAAGTCACATTAGCCATCATCCTATTATCAGTCCTACTAATAAATGGCTCCTTTACCCTATCCACTCTAATCATCACACAAGAACACC
A-M_putorius : TCCGGGTGGGCCTCAAACTCAAAATACGCCCTAATCGGAGCCCTACGGGCCGTAGCCCAAACAATCTCCTACGAAGTCACATTAGCCATCATCCTATTATCAGTCCTACTAATAAATGGCTCCTTTACCCTATCCACTCTAATCATCACACAAGAACACC
R-B_taurus : TCAGGCTGAGCTTCCAACTCAAAATACGCACTAATCGGAGCCCTACGAGCAGTAGCACAAACAATCTCATACGAAGTAACGCTAGCAATTATCCTGTTATCAGTGGTCCTAATAAGTGGGTCCTTTACCCTCTCCACATTAATTACTACACAAGAACAAA
A-B_taurus : TCAGGCTGAGCTTCCAACTCAAAATACGCACTAATCGGAGCCCTACGAGCAGTAGCACAAACAATCTCATACGAAGTAACGCTAGCAATTATCCTGTTATCAGTACTCCTAATAAGTGGGTCCTTTACCCTCTCCACATTAATTACTACACAAGAACAAA
R-O_aries : TCAGGTTGAGCCTCCAACTCAAAATATGCTCTCATTGGAGCCCTACGGGCAGTAGCACAAACAATCTCTTATGAAGTAACACTAGCAATTATTTTACTATCAGTCCTACTAATAAATGGGTCCTTTACCCTTTCTACACTAATCATTACACAAGAACAAG
A-O_aries : TCAGGTTGAGCCTCCAACTCAAAATATGCTCTCATTGGAGCCCTACGGGCAGTAGCACAAACAATCTCTTATGAAGTAACACTAGCAATTATTTTACTATCAGTCCTACTAATAAATGGATCCTTTACCCTTTCTACACTAATCATTACACAAGAACAAG
R-S_scrofa : TCAGGATGAGCATCTAACTCAAAATACGCACTCATCGGGGCCCTACGAGCAGTAGCCCAAACAATTTCATATGAAGTAACACTGGCAATCATCCTACTATCAGTGCTCCTAATAAATGGATCATATACTCTATCCACCCTAATCACAACACAAGAGCACA
A-S_scrofa : TCAGGATGAGCATCTAACTCAAAATACGCACTCATCGGGGCCCTACGAGCAGTAGCCCAAACAATTTCATATGAAGTAACACTGGCAATCATCCTACTATCAGTGCTCCTAATAAATGGATCATATACTCTATCCACCCTAATCACAACACAAGAGCACA
R-O_cuniculus : TCAGGATGAGCATCCAACTCAAAATATGCCCTATTTGGTGCTCTCCGAGCAGTCGCACAAACCATTTCTTACGAAGTCACACTTGCAATTATCCTCCTATGCATCCTCCTAATAAATGGCTCATTTACATTATCATCCCTAATCACAACACAAGAATATA
A-O_cuniculus : TCAGGATGAGCATCCAACTCAAAATACGCCCTATTTGGTGCTCTCCGAGCAGTCGCACAAACCATTTCCTACGAAGTCACACTTGCAATTATCCTCCTATGCATCCTCCTAATAAATGGCTCATTTACATTATCATCCCTAATCACAACACAAGAATATA
R-M_musculus : TCAGGATGAGCCTCAAACTCCAAATACTCACTATTCGGAGCTTTACGAGCCGTAGCCCAAACAATTTCATATGAAGTAACCATAGCTATTATCCTTTTATCAGTTCTATTAATAAATGGATCCTACTCTCTACAAACACTTATTACAACCCAAGAACACA
A-M_musculus : TCAGGATGAGCCTCAAACTCCAAATACTCACTATTCGGAGCTTTACGAGCCGTAGCCCAAACAATTTCATATGAAGTAACCATAGCTATTATCCTTTTATCAGTTCTATTAATAAATGGATCCTACTCTCTACAAACACTTATTACAACCCAAGAACACA
R-R_norvegicus : TCAGGATGAGCATCAAATTCAAAATACTCCCTATTCGGAGCCCTACGAGCCGTTGCCCAAACCATCTCTTACGAAGTCACAATAGCCATTATCCTCTTATCCGTCCTCCTAATAAGCGGCTCCTTCTCCCTACAAATACTTATCACTACACAAGAACATA
A-R_norvegicus : TCAGGATGAGCATCAAATTCAAAATACTCCCTATTCGGAGCCCTACGAGCCGTTGCCCAAACCATCTCTTACGAAGTCACAATAGCCATTATCCTCTTATCCGTCCTCCTAATAAGCGGCTCCTTCTCCCTACAAATACTTATCACTACACAAGAACATA
R-M_coypus : TCCGGGTGAGCATCAAATTCTAAATATGCTTTATTTGGTGCCCTACGGGCTGTAGCACAAACTATTTCTTATGAAGTAACACTCGCCATCATTCTCCTCTCTGTCCTATTACTAAATGGCTCCTTCACTCTATCAACCCTAATAATCACACAAAAAAACA
A-M_coypus : TCCGGGTGAGCATCAAATTCTAAATATGCTTTATTTGGTGCCCTACGGGCTGTAGCACAAACTATTTCTTATGAAGTAACACTCGCCATCATTCTCCTCTCTGTCCTATTACTAAATGGCTCCTTCACTCTATCAACCCTAATAATCACACAAAAAAACA
R-A_platyrhynchos : TCAGGCTGAGCCTCAAACTCAAAATATGCACTAATCGGAGCCCTACGGGCAGTTGCACAAACCATCTCATATGAAGTAACACTAGCACTCATCCTACTGTCAGTAATCATACTAACCGGAAACTACACACTCAGCACTTTTGCCATCGCACAAGAACCCC
A-A_platyrhynchos : TCAGGCTGAGCCTCAAACTCAAAATATGCACTAATCGGAGCCCTACGGGCAGTTGCACAAACCATCTCATATGAAGTAACACTAGCACTCATCCTACTGTCAGTAATCATACTAACCGGAAACTACACACTCAGCACTTTTGCCATCGCACAAGAACCCC
R-G_gallus : TCCGGATGAGCATCAAACTCCAAGTATGCCCTAATCGGAGCCCTTCGAGCCGTCGCACAAACAATCTCATACGAAGTCACCCTAGCCATCATCCTGTTATCCACAATCATACTGAGCGGCAATTACACCTTAAGCACCCTGGCCATCACCCAAGAGCCCA
A-G_gallus : TCCGGATGAGCATCAAACTCCAAGTATGCCCTAATCGGAGCCCTTCGAGCCGTCGCACAAACAATCTCATACGAAGTCACCCTAGCCATCATCCTGTTATCCACAATCATACTGAGCGGCAATTACACCTTAAGCACCCTGGCCATCACCCAAGAACCCA
 TCAGGATGAGCCTCAAACTCAAAATACGCCCTAATCGGAGCCCTACGAGCMGTAGCACAAACAATCTCATATGAAGTAACACTAGCAATCATCCTACTATCAGTCCTCCTAATAAATGGATCCTTCACMCTATCCACACTAATCATTACACAAGAACACA

 * 4820 * 4840 * 4860 * 4880 * 4900 * 4920 * 4940 * 4960
R-E_caballus : TCTGATTAATCTTCCCATCATGACCCTTAGCCATAATGTGATTCATCTCAACATTAGCCGAAACCAACCGAGCTCCATTTGACCTAACAGAAGGAGAATCAGAACTCGTCTCTGGATTCAACGTTGAATACGCAGCCGGCCCATTTGCTCTATTCTTCCT
A-E_caballus : TCTGATTAATCTTCCCATCATGACCCTTAGCCATAATGTGATTCATCTCAACATTAGCCGAAACCAACCGAGCTCCATTTGACCTAACAGAAGGAGAATCAGAACTCGTCTCTGGATTCAACGTTGAATACGCAGCCGGCCCATTTGCTCTATTCTTCCT
R-C_bactrianus : TATGAATAATCGTACCTGCTTGACCTCTGGCTATAATATGATTTATCTCCACGCTAGCTGAAACCAACCGAGCCCCCTTTGACCTCACCGAAGGGGAATCTGAATTAGTATCAGGCTTTAACGTAGAATATGCAGCAGGCCCCTTCGCTATATTTTTCAT
A-C_bactrianus : TATGAATAATCGTACCTGCTTGACCTCTGGCTATAATATGATTTATCTCCACGCTAGCTGAAACCAACCGAGCCCCCTTTGACCTCACCGAAGGGGAATCTGAATTAGTATCAGGCTTTAACGTAGAATATGCAGCAGGCCCCTTCGCTATATTTTTCAT
R-C_lupus_familiaris : TATGATTAATCTTTCCGGCCTGACCCCTAGCCATGATATGATTCATCTCTACCCTAGCAGAAACTAATCGAGCCCCCTTCGACTTAACTGAAGGAGAATCCGAACTAGTCTCTGGATTTAACGTAGAGTATGCAGCAGGTCCTTTCGCCCTATTCTTTCT
A-C_lupus_familiaris : TATGATTAATCTTTCCGGCCTGACCCCTAGCCATGATATGATTCATCTCTACCCTAGCAGAAACTAATCGAGCCCCCTTCGACTTAACTGAAGGAGAATCCGAACTAGTCTCTGGATTTAACGTAGAGTATGCAGCAGGTCCTTTCGCCCTATTCTTTCT
R-N_procyonoides : TATGATTAATTTTTCCAGCCTGACCGTTAGCCATAATATGATTTATTTCCACACTAGCAGAAACAAACCGAGCCCCCTTCGACCTAACTGAAGGAGAATCGGAACTTGTATCTGGGTTTAACGTAGAATACGCCGCAGGTCCATTCGCCCTCTTCTTTAT
A-N_procyonoides : TATGATTAATTTTTCCAGCCTGACCGTTAGCCATAATATGATTTATTTCCACACTAGCAGAAACAAACCGAGCCCCCTTCGACCTAACTGAAGGAGAATCGGAACTTGTATCTGGGTTTAACGTAGAATACGCCGCAGGTCCATTCGCCCTCTTCTTTAT
R-V_lagopus : TGTGACTAATCTTCCCCGCCTGACCTCTGGCCATAATATGGTTTATCTCTACTTTAGCGGAAACAAATCGAGCCCCCTTTGATTTGACTGAAGGGGAGTCAGAGCTGGTCTCAGGATTTAACGTAGAGTATGCAGCCGGACCCTTCGCCCTATTTTTCCT
A-V_lagopus : TGTGACTAATCTTCCCCGCCTGACCTCTGGCCATAATATGGTTTATCTCTACTTTAGCGGAAACAAATCGAGCCCCCTTTGATTTGACTGAAGGGGAGTCAGAGCTGGTCTCAGGATTTAACGTAGAGTATGCAGCCGGACCCTTCGCCCTATTTTTCCT
R-M_putorius : TATGACTAATTTTCCCTGCATGACCTTTAGCTATAATATGATTTATTTCAACCTTAGCAGAAACTAACCGCGCCCCGTTCGACCTAACTGAGGGAGAATCCGAACTAGTTTCAGGGTTCAACGTCGAATATGCAGCAGGACCATTCGCCCTATTCTTCCT
A-M_putorius : TATGACTAATTTTCCCTGCATGACCTTTAGCTATAATATGATTTATTTCAACCTTAGCAGAAACTAACCGCGCCCCGTTCGACCTAACTGAGGGAGAATCCGAACTAGTTTCAGGGTTCAACGTCGAATATGCAGCAGGACCATTCGCCCTATTCTTCCT
R-B_taurus : TATGGTTAATCCTCCCAGCATGGCCTCTAGCAATAATATGATTTATCTCAACACTAGCAGAAACAAACCGAGCTCCATTTGATTTAACTGAAGGAGAATCAGAGCTAGTCTCGGGCTTCAACGTAGAATATGCAGCAGGACCATTTGCCCTCTTCTTCAT
A-B_taurus : TATGGTTAATCCTCCCAGCATGGCCTCTAGCAATAATATGATTTATCTCAACACTAGCAGAAACAAACCGAGCTCCATTTGATTTAACTGAAGGAGAATCAGAGCTAGTCTCGGGCTTCAACGTAGAATATGCAGCAGGACCATTTGCCCTCTTCTTCAT
R-O_aries : TATGATTAATCTTCCCAGCATGACCCCTAGCAATAATATGATTTATCTCAACACTAGCAGAAACAAACCGAGCACCATTTGACCTCACCGAAGGAGAATCTGAACTAGTATCAGGCTTTAACGTAGAATATGCTGCCGGACCATTCGCCCTATTCTTTAT
A-O_aries : TATGATTAATCTTCCCAGCATGACCCCTAGCAATAATATGATTTATCTCAACACTAGCAGAAACAAACCGAGCACCATTTGACCTCACCGAAGGAGAATCTGAACTAGTATCAGGCTTTAACGTAGAATATGCTGCCGGACCATTCGCCCTATTCTTTAT
R-S_scrofa : TTTGAATAATCTTTACATCCTGACCCCTAGCCATAATATGATTTATCTCAACCCTAGCAGAAACCAACCGAGCCCCGTTCGACCTTACAGAAGGAGAGTCAGAACTTGTATCAGGCTTTAACGTAGAATATGCAGCCGGACCTTTCGCCATATTCTTCAT
A-S_scrofa : TTTGAATAATCTTTACATCCTGACCCCTAGCCATAATATGATTTATCTCAACCCTAGCAGAAACCAACCGAGCCCCGTTCGACCTTACAGAAGGAGAGTCAGAACTTGTATCAGGCTTTAACGTAGAATATGCAGCCGGACCTTTCGCCATATTCTTCAT
R-O_cuniculus : TATGAATCCTACTACCAGCATGACCACTTGCCATAATATGATTTATCTCGACCCTAGCAGAAACCAACCGGGCTCCATTTGACTTAACCGAAGGTGAATCAGAGCTCGTCTCCGGCTTCAATGTGGAGTACGCAGGAGGCCCCTTTGCTCTTTTCTTTTT
A-O_cuniculus : TATGAATCCTACTACCAGCATGACCACTTGCCATAATATGATTTATCTCAACCCTAGCAGAAACCAACCGGGCTCCATTTGACTTAACCGAAGGTGAATCAGAGCTCGTCTCCGGCTTCAATGTGGAGTACGCAGGAGGCCCCTTTGCTCTTTTCTTTTT
R-M_musculus : TATGATTACTTCTGCCAGCCTGACCCATAGCCATAATATGATTTATCTCAACCCTAGCAGAAACAAACCGGGCCCCCTTCGACCTGACAGAAGGAGAATCAGAATTAGTATCAGGGTTTAACGTAGAATACGCAGCCGGCCCATTCGCGTTATTCTTTAT
A-M_musculus : TATGATTACTTCTGCCAGCCTGACCCATAGCCATAATATGATTTATCTCAACCCTAGCAGAAACAAACCGGGCCCCCTTCGACCTGACAGAAGGAGAATCAGAATTAGTATCAGGGTTTAACGTAGAATACGCAGCCGGCCCATTCGCGTTATTCTTTAT
R-R_norvegicus : TCTGACTATTAATCCCCGCCTGACCAATAGCCATAATATGATACATTTCAACCCTAGCAGAAACAAATCGAGCTCCCTTCGACTTAACAGAAGGAGAATCAGAATTAGTCTCAGGCTTTAACGTCGAATACGCCGCAGGACCATTCGCCCTATTCTTCAT
A-R_norvegicus : TCTGACTATTAATCCCCGCCTGACCAATAGCCATAATATGATACATTTCAACCCTAGCAGAAACAAATCGAGCTCCCTTCGACTTAACAGAAGGAGAATCAGAATTAGTCTCAGGCTTTAACGTTGAATACGCCGCAGGACCATTCGCCCTATTCTTCAT
R-M_coypus : TCTGGTTAATCCTCCCTACATGACCACTAGCAATAATATGATTTGTGTCTACTCTAGCAGAAACAAACCGAGCCCCCTTCGACCTAACCGAAGGAGAATCTGAACTAGTCTCAGGTTTTAACGTTGAATACGCTGCGGGACCGTTTGCTTTATTCTTTAT
A-M_coypus : TCTGGTTAATCCTCCCTACATGACCACTAGCAATAATATGATTTGTGTCTACTCTAGCAGAAACAAACCGAGCCCCCTTCGACCTAACCGAAGGAGAATCTGAACTAGTCTCAGGTTTTAACGTTGAATACGCTGCGGGACCATTTGCTTTATTCTTTAT
R-A_platyrhynchos : TTTACCTCATCTTCTCCTCGTGGCCCCTGGCAATAATGTGATATGTATCCACCCTAGCAGAAACAAACCGGGCCCCATTTGACCTAACGGAGGGCGAGTCTGAACTGGTCTCAGGGTTTAACGTTGAATACGCCGCAGGGCCTTTCGCCCTGTTTTTCCT
A-A_platyrhynchos : TTTACCTCATCTTCTCCTCGTGGCCCCTGGCAATAATGTGATATGTATCCACCCTAGCAGAAACAAACCGGGCCCCATTTGACCTAACGGAGGGCGAGTCTGAACTGGTCTCAGGGTTTAACGTTGAATACGCCGCAGGGCCTTTCGCCCTGTTTTTCCT
R-G_gallus : TCTACCTCATTTTTTCCGCATGACCCCTCGCAATAATATGATACATCTCTACCCTTGCTGAAACCAACCGCGCCCCATTTGACCTAACAGAAGGAGAGTCAGAGCTAGTCTCAGGATTTAATGTAGAATATGCCGCCGGACCATTCGCCATATTCTTCTT
A-G_gallus : TCTACCTCATTTTTTCCGCATGACCCCTCGCAATAATATGATACATCTCTACCCTTGCTGAAACCAACCGCGCCCCATTTGACCTAACAGAAGGAGAGTCAGAGCTAGTCTCAGGATTTAATGTAGAATATGCCGCCGGACCATTCGCCATATTCTTCTT
 TATGATTAATCTTCCCAGCATGACCCCTAGCCATAATATGATTTATCTCAACCCTAGCAGAAACAAACCGAGCCCCCTTTGACCTAACWGAAGGAGAATCAGAACTAGTCTCAGGCTTTAACGTAGAATATGCAGCAGGACCATTCGCCCTATTCTTCAT

 * 4980 * 5000 * 5020 * 5040 * 5060 * 5080 * 5100 * 5120
R-E_caballus : AGCAGAATACGCAAACATCATCATGATAAACATCTTCACAACAACCCTATTTCTAGGAGCA--TTTCACAACCCCT-ACCTGCC-AGAACTCTACTCAATTAATTTCACCATTAAAGCTCTCCTTCTAACATGTTCCTTCCTATGAATCCGAGCATCCTA
A-E_caballus : AGCAGAATACGCAAACATCATCATGATAAACATCTTCACAACAACCCTATTTCTAGGAGCA--TTTCACAACCCTT-ACCTGCC-AGAACTCTACTCAATTAATTTCACCATTAAAGCTCTCCTTCTAACATGTTCCTTCCTATGAATCCGAGCATCCTA
R-C_bactrianus : AGCAGAGTACGCCAATATCATCATGATAAATGCCTTCACAACTATCCTCTTCTTCGGAGCC--TTTCACAATCCCT-ACATGCC-GGAGCTATATACAGTCAATTTCGTAGCCAAAACACTGCTACTAACCGCAACTTTCCTATGAATTCGAGCATCCTA
A-C_bactrianus : AGCAGAGTACGCCAATATCATCATGATAAATGCCTTCACAACTATCCTCTTCTTCGGAGCC--TTTCACAATCCCT-ACATGCC-GGAGCTATATACAGTCAATTTCGTAGCCAAAACACTGCTACTAACCGCAACTTTCCTATGAATTCGAGCATCCTA
R-C_lupus_familiaris : AGCAGAGTACGCAAATATTATTATAATAAACATCCTCACAACAATTCTGTTCTTCGGCGCA--TTCCACAACCCAT-TCATACC-AGAACTCTACTCTATTAACTTCACTATAAAAACCCTCTTATTAACCATCTGCTTCCTATGAATTCGAGCATCATA
A-C_lupus_familiaris : AGCAGAGTACGCAAATATTATTATAATAAACATCCTCACAACAATTCTGTTCTTCGGCGCA--TTCCACAACCCAT-TCATACC-AGAACTCTACTCTATTAACTTCACTATAAAAACCCTCTTATTAACCATCTGCTTCCTATGAATTCGAGCATCATA
R-N_procyonoides : AGCAGAATACGCTAACATTATCATAATAAATGCCCTTACAACAATTCTATTTTTCGGCGCA--TTCCATAACCCGT-TCCTACC-CGAACTCTACTCCATTAACTTTACAATTAAAGCCCTACTCCTAACCATCTCATTCCTATGAATCCGAGCATCATA
A-N_procyonoides : AGCAGAATACGCTAACATTATCATAATAAATGCCCTTACAACAATTCTATTTTTCGGCGCA--TTCCATAACCCGT-TCCTACC-CGAACTCTACTCCATTAACTTTACAATTAAAGCCCTACTCCTAACCATCTCATTCCTATGAATCCGAGCATCATA
R-V_lagopus : AGCAGAGTATGCAAATATTATCATGATAAACATCCTCACAACGATTTTATTCTTTGGCGCA--TTCCATAACCCGT-TCCTACC-AGAACTCTACTCAATCAACTTCACTATTAAGACCCTTTTACTAACCATCTCCTTCCTATGAATTCGAGCATCATA
A-V_lagopus : AGCAGAGTATGCAAATATTATCATGATAAACATCCTCACAACGATTTTATTCTTTGGCGCA--TTCCATAACCCGT-TCCTACC-AGAACTCTACTCAATCAACTTCACTATTAAGACCCTTTTACTAACCATCTCCTTCCTATGAATTCGAGCATCATA
R-M_putorius : AGCCGAATACGCCAACATCATCATAATAAATATCCTCACAACTATCCTATTCTTCGGCGCA--TTTCACACCCCCT-ACCTTCC-AGAATTATATTCCATTAATTTCACTATAAAAACCCTCTTATTAACAACTTCTTTCCTATGAATTCGAGCATCATA
A-M_putorius : AGCCGAATACGCCAACATCATCATAATAAATATCCTCACAACTATCCTATTCTTCGGCGCA--TTTCACACCCCCT-ACCTTCC-AGAATTATATTCCATTAATTTCACTATAAAAACCCTCTTATTAACAACTTCTTTCCTATGAATTCGAGCATCATA
R-B_taurus : AGCAGAGTACGCAAATATTATCATAATAAATATCTTTACAGCAATTTTATTCCTAGGAACA--TCCCACAATCCAC-ACATACC-AGAACTCTACACAATCAATTTTACCATTAAATCCCTACTGCTCACAATATCCTTCCTATGAATCCGAGCATCCTA
A-B_taurus : AGCAGAGTACGCAAATATTATCATAATAAATATCTTTACAGCAATTTTATTCCTAGGAACA--TCCCACAATCCAC-ACATACC-AGAACTCTACACAATCAATTTTACCATTAAATCCCTACTGCTCACAATATCCTTCCTATGAATCCGAGCATCCTA
R-O_aries : AGCAGAATATGCGAATATTATCATAATAAACATCTTCACAACAACCCTCTTCTTAGGAGCA--TTTCACAACCCAT-ACATACC-AGAACTTTACACAATCAACTTCACCATCAAATCGCTACTACTCTCAATTACCTTCCTATGAATCCGAGCATCCTA
A-O_aries : AGCAGAATATGCAAATATTATCATAATAAACATCTTCACAACAACCCTCTTCTTAGGAGCA--TTTCACAACCCAT-ACATACC-AGAACTTTACACAATCAACTTCACCATCAAATCGCTACTACTCACAATTACCTTCCTATGAATCCGAGCATCCTA
R-S_scrofa : AGCAGAATATGCCAACATCATCATAATAAATGCATTCACAGCAATTCTCTTCCTAGGAGCA--TTCCACGACCCAC-ACACATC-AGAACTATATACAATCAACTTCGTACTAAAAACACTCGCATTAACAATCACCTTCCTATGAATCCGAGCATCATA
A-S_scrofa : AGCAGAATATGCCAACATCATCATAATAAATGCATTCACAGCAATTCTCTTCCTAGGAGCA--TTCCACGACCCAC-ACACATC-AGAACTATATACAATCAACTTCGTACTAAAAACACTCGCATTAACAATCACCTTCCTATGAATCCGAGCATCATA
R-O_cuniculus : AGCTGAATATACTAATATTATCCTTATAAATGCCCTTACAGCCATCCTATTCCTAGGCTCA--TTTCACAG-CCAC-ACTAACCCAGAAATATTTACAGTCAACTTTGCCACCAAGACCCTCCTACTCACAATAACATTTCTATGGATTCGAGCATCCTA
A-O_cuniculus : AGCTGAATATACTAATATTATCCTTATAAATGCCCTTACAGCCATCCTATTCCTAGGCTCA--TTTCACAG-CCAC-ACTAACCCAGAAATATTTACAGTCAACTTTGCCACAAAGACTCTCCTACTCACAATAACATTTCTATGGATTCGAGCATCCTA
R-M_musculus : AGCAGAGTACACTAACATTATTCTAATAAACGCCCTAACAACTATTATCTTCCTAGGACC---C-CTATACTATATCAATTTACCAGAACTCTACTCAACTAACTTCATAATAGAAGCTCTACTACTATCATCAACATTCCTATGGATCCGAGCATCTTA
A-M_musculus : AGCAGAGTACACTAACATTATTCTAATAAACGCCCTAACAACTATTATCTTCCTAGGACC---C-CTATACTATATCAATTTACCAGAACTCTACTCAACTAACTTCATAATAGAAGCTCTACTACTATCATCAACATTCCTATGGATCCGAGCATCTTA
R-R_norvegicus : AGCCGAGTACACCAATATTATCCTAATAAACGCCCTAACATCAATTGTATTCCTAGGCCC---C-TTATATCACATCAATTACCCTGAATTATACTCAACCAGCTTCATAACAGAAACACTACTTCTATCCACAACTTTCCTATGAATCCGAGCATCCTA
A-R_norvegicus : AGCCGAGTACACCAATATTATCCTAATAAATGCCCTAACATCAATTGTATTCCTAGGCCC---C-TTATATCATATCAATTACCCTGAATTATACTCAACCAGCTTCATAACAGAAACACTACTTCTATCCACAACTTTCCTATGAATCCGAGCATCCTA
R-M_coypus : AGCCGAATACATAAACATTTTATTAATAAATGCCTTAACAACAATTATCTTCTTAAATTC---TACTATAATA-ACTATACACCCTAATTTATTCTCAATAAACTTCACAATCAAAACATTAATTCTAACTGCACTATTTTTATGAATTCGAGCTTCATA
A-M_coypus : AGCCGAATACATAAACATTTTATTAATAAATGCCTTAACAACAATTATCTTCTTAAATTC---TACTATAATA-ACTATACACCCTAATTTATTCTCAATAAACTTCACAATCAAAACATTAATTCTAACTGCACTATTCTTATGAATTCGAGCTTCATA
R-A_platyrhynchos : AGCCGAATATGCCAACATCATGCTGATAAACACGCTCACAGCCATCATCTTCCTTAACCCAAGCGCCCTAGGGCCC---CCTAC-AGAGCTATTCCCCATCATCCTAGCCACAAAAGTCCTCCTTCTATCCTCCGGCTTCCTATGGGTCCGAGCCTCCTA
A-A_platyrhynchos : AGCCGAATATGCCAACATCATGCTGATAAACACGCTCACAGCCATCATCTTCCTTAACCCAAGCGCCCTAGGGCCC---CCTAC-AGAGCTATTCCCCATCATCCTAGCCACAAAAGTCCTCCTTCTATCCTCCGGCTTCCTATGGGTCCGAGCCTCCTA
R-G_gallus : AGCCGAATACGCCAACATTATACTAATAAACACACTAACCACCGTCCTATTCCTGAACCCAAGCTTCCTAAATCTC---CCACC-AGAATTATTTCCCATTGCACTCGCTACAAAAACCCTCCTCCTTTCATCCTCATTTCTATGAATCCGGGCCTCATA
A-G_gallus : AGCCGAATACGCCAACATTATACTAATAAACACACTAACCACCGTCCTATTCCTGAACCCAAGCTTCCTAAATCTC---CCACC-AGAATTATTTCCCATTGCACTCGCTACAAAAACCCTCCTCCTTTCATCCTCATTTCTATGAATCCGGGCCTCATA
 AGCAGAATACGCCAACATTATCATAATAAAYACCCTCACAACAATTCTATTCCTAGGCGCAAGTTCCACAACCCATCACMTACCCAGAACTATACTCAATCAACTTCACAATAAAAACCCTCCTACTAACAAYMTCCTTCCTATGAATCCGAGCATCMTA

 * 5140 * 5160 * 5180 * 5200 * 5220 * 5240 * 5260 * 5280
R-E_caballus : CCCACGATTCCGATATGACCAACTTATACACCTCCTATGAAAGAACTTCCTACCACTCACACTAGCCCTCTGCATATGACACGTCTCACTTCCAATCATACTATCCAGCATCCCACCACAAACA---TAGGAAATATGTCTGA--CAAAAGAGTTACTTT
A-E_caballus : CCCACGATTCCGATATGACCAACTTATACACCTCCTATGAAAGAACTTCCTACCACTCACACTAGCCCTCTGCATATGACACGTCTCACTTCCAATCATACTATCCAGCATCCCACCACAAACA---TAAGAAATATGTCTGA--CAAAAGAGTTACTTT
R-C_bactrianus : TCCCCGATTCCGATACGATCAGCTAATACACCTCCTATGAAAAAATTTTCTTCCCCTTACCCTAGCCCTATGCATATGACACGTGTCGCTGCCCATTTCAACAGCAGGAATTCCCCCTCAAACA---TAAGAAATATGTCTGA--CAAAAGAGTTACTTT
A-C_bactrianus : TCCCCGATTCCGATACGATCAGCTAATACACCTCCTATGAAAAAATTTTCTTCCCCTTACCCTAGCCCTATGCATATGACACGTGTCGCTGCCCATTTCAACAGCAGGAATTCCCCCTCAAACA---TAAGAAATATGTCTGA--CAAAAGAGTTACTTT
R-C_lupus_familiaris : CCCTCGATTCCGCTACGATCAGTTAATACACTTATTATGAAAAAATTTTCTACCCTTAACTTTAGCCCTATGCATATGACATGTTGCCTTACCCATTATCACCGCAAGTATCCCACCCCAAACA---TAAGAAATATGTCTGA--TAAAAGAGTTACTTT
A-C_lupus_familiaris : CCCTCGATTCCGCTACGATCAGTTAATACACTTATTATGAAAAAATTTTCTACCCTTAACTTTAGCCCTATGCATATGACATGTTGCCTTACCCATTATCACCGCAAGTATCCCACCCCAAACA---TAAGAAATATGTCTGA--TAAAAGAGTTACTTT
R-N_procyonoides : CCCTCGGTTCCGCTATGACCAACTAATACATCTATTATGAAAAAACTTCCTGCCACTAACCCTAGCCCTATGCATATGGCATGTAGCCCTACCCATCATCACAGCAAGCATCCCACCCCAAACA---TAAGAAATATGTCTGA--CAAAAGAGTTACTTT
A-N_procyonoides : CCCTCGGTTCCGCTATGACCAACTAATACATCTATTATGAAAAAACTTCCTGCCACTAACCCTAGCCCTATGCATATGGCATGTAGCCCTACCCATCATCACAGCAAGCATCCCACCCCAAACA---TAAGAAATATGTCTGA--CAAAAGAGTTACTTT
R-V_lagopus : CCCTCGATTCCGCTACGATCAACTAATACACCTCTTATGAAAAAATTTCCTGCCACTAACTTTGGCCCTATGTATATGACACGTTGCCCTACCTATCATTACCGCAAGCATCCCACCTCAAACA---TAAGAAATATGTCTGA--CAAAAGAGTTACTTT
A-V_lagopus : CCCTCGATTCCGCTACGATCAACTAATACACCTCTTATGAAAAAATTTCCTGCCACTAACTTTGGCCCTATGTATATGACACGTTGCCCTACCTATCATTACCGCAAGCATCCCACCTCAAACA---TAAGAAATATGTCTGA--CAAAAGAGTTACTTT
R-M_putorius : CCCCCGATTCCGCTATGACCAACTAATACACTTGCTATGAAAAAATTTTCTTCCCTTGACATTAGCCTTATGTATATGACATATAGCCCTCCCCATTATAACTGCGAGCATCCCCCCACAAACA---TAAGAAATATGTCTGA--CAAAAGAGTTACTTT
A-M_putorius : CCCCCGATTCCGCTATGACCAACTAATACACTTGCTATGAAAAAATTTTCTTCCCTTGACATTAGCCTTATGTATATGACATATAGCCCTCCCCATTATAACTGCGAGCATCCCCCCACAAACA---TAAGAAATATGTCTGA--CAAAAGAGTTACTTT
R-B_taurus : CCCTCGATTTCGCTATGACCAACTAATACACTTACTATGAAAAAATTTTCTACCTCTGACACTAGCCCTGTGCATGTGACACGTATCCCTACCCATCCTTACATCAGGCATCCCACCACAAACA---TAAGAAATATGTCTGA--CAAAAGAGTTACTTT
A-B_taurus : CCCTCGATTTCGCTATGACCAACTAATACACTTACTATGAAAAAATTTTCTACCTCTGACACTAGCCCTGTGCATGTGACACGTATCCCTACCCATCCTTACATCAGGCATCCCACCACAAACA---TAAGAAATATGTCTGA--CAAAAGAGTTACTTT
R-O_aries : CCCCCGATTCCGTTACGACCAACTAATACACTTACTATGAAAAAATTTTCTACCCCTAACACTAGCCCTATGCATATGACACGTATCGCTACCTATTCTCCTATCAAGCATCCCCCCACAAACA---TAAGAAATATGTCTGA--CAAAAGAGTTACTTT
A-O_aries : CCCCCGATTCCGTTACGACCAACTAATACACTTACTATGAAAAAATTTTCTACCCCTAACACTAGCCCTATGCATATGACACGTATCACTACCTATTCTCCTATCAAGCATCCCCCCACAAACA---TAAGAAATATGTCTGA--CAAAAGAGTTACTTT
R-S_scrofa : CCCACGATTCCGATATGACCAACTAATACATTTACTATGAAAAAGCTTCCTGCCCCTAACACTAGCTCTATGTATATGACACATCTCACTCCCTATTATAACAGCAAGTATTCCCCCACAATCA---TA-GAAATATGTCTGA--TAAAAGAGTTACTTT
A-S_scrofa : CCCACGATTCCGATATGACCAACTAATACATTTACTATGAAAAAGCTTCCTGCCCCTAACACTAGCTCTATGTATATGACACATCTCACTCCCTATTATAACAGCAAGTATTCCCCCACAATCA---TA-GAAATATGTCTGA--TAAAAGAGTTACTTT
R-O_cuniculus : TCCCCGATTTCGCTATGACCAGCTAATACACCTCTTGTGAAAAAGTTTTCTACCCCTCACATTAGCCCTGTGCATATGACATATCTCAATGCCTATCATACTTTCAAGTATCCCTCCTCAGATA---TA-GAAATATGTCTGA--TAAAAGAGTTACTTT
A-O_cuniculus : TCCCCGATTTCGCTATGACCAGCTAATACACCTCTTGTGAAAAAGTTTTCTACCCCTCACATTAGCCCTGTGCATATGACATATCTCAATACCTATCATACTTTCAAGTATCCCTCCTCAGATA---TA-GAAATATGTCTGA--TAAAAGAGTTACTTT
R-M_musculus : TCCACGCTTCCGTTACGATCAACTTATACATCTTCTATGAAAAAACTTTCTACCCCTAACACTAGCATTATGTATGTGACATATTTCTTTACCAATTTTTACAGCGGGAGTACCACCATACATA---TA-GAAATATGTCTGA--TAAAAGAATTACTTT
A-M_musculus : TCCACGCTTCCGTTACGATCAACTTATACATCTTCTATGAAAAAACTTTCTACCCCTAACACTAGCATTATGTATGTGACATATTTCTTTACCAATTTTTACAGCGGGAGTACCACCATACATA---TA-GAAATATGTCTGA--TAAAAGAATTACTTT
R-R_norvegicus : CCCCCGTTTTCGATATGACCAACTAATGCACCTCCTATGAAAAAATTTCCTCCCACTAACACTAGCATTCTGCATATGATACATTTCCCTGCCAATTTTCCTAGCAGGAATTCCACCCTACACA---TA-GAAATATGTCTGA--CAAAAGAGTTACTTT
A-R_norvegicus : CCCCCGTTTTCGATATGACCAACTAATGCACCTCCTATGAAAAAATTTCCTCCCACTAACACTAGCATTCTGCATATGATACATTTCCCTGCCAATTTTCCTAGCAGGAATTCCACCCTACACA---TA-GAAATATGTCTGA--CAAAAGAGTTACTTT
R-M_coypus : TCCCCGATTCCGTTATGACCAGCTCATGCACCTATTATGAAAAAATTTTCTCCCACTAACCCTTGCACTATGCATATGACACATCTCAATACCAATCTTCCTATCCAGCATCCCTCCTCAATCAATCTA-GAAATATGTCTGA--AAATAGAGTTACTTT
A-M_coypus : TCCCCGATTCCGTTATGACCAGCTCATGCACCTATTATGAAAAAATTTTCTCCCACTAACCCTTGCACTATGCATATGACACATCTCAATACCAATCTTCCTATCCAGCATCCCTCCTCAATCAATCTA-GAAATATGTCTGA--AAATAGAGTTACTTT
R-A_platyrhynchos : CCCCCGATTCCGATATGACCAGCTTATGCACCTCCTATGAAAAAACTTCCTACCCCTCACACTAGCCCTATGCCTCTGACACACTAGCCTACCCATCTGCTACGCAGGCCTACCTCCTTCCACA----AGGAAATGTGCCTGAACTCAAAGGGTCACTAT
A-A_platyrhynchos : CCCCCGATTCCGATATGACCAGCTTATGCACCTCCTATGAAAAAACTTCCTACCCCTCACACTAGCCCTATGCCTCTGACACACTAGCCTACCCATCTGCTACGCAGGCCTACCTCCTTCCACA----AGGAAATGTGCCTGAACTCAAAGGGTCACTAT
R-G_gallus : TCCACGGTTCCGCTATGACCAACTAATACATCTTCTATGAAAAAACTTCCTCCCCCTAACCCTAGCCTTATGCCTCTGACATACCAGCATACCAATCAGCTACGCCGGCCTCCCCCCAATC-TA----AGGAAGCGTGCCTGAACAAAAAGGATCACTAT
A-G_gallus : TCCACGGTTCCGCTATGACCAACTAATACATCTTCTATGAAAAAACTTCCTCCCCCTAACCCTAGCCTTATGCCTCTGACATACCAGCATACCAATCAGCTACGCCGGCCTCCCCCCAATC-TA----AGGAAGCGTGCCTGAACAAAAAGGATCACTAT
 CCCCCGATTCCGCTATGACCAACTAATACACCTMCTATGAAAAAATTTTCTACCCCTAACACTAGCCCTATGCATATGACACATYTCCCTACCCATCATCACAGCAAGCATCCCACCACAAACAATCTAAGAAATATGTCTGAACCAAAAGAGTTACTTT

 * 5300 * 5320 * 5340 * 5360 * 5380 * 5400 * 5420 * 5440
R-E_caballus : GATAGAGTAAAACATAGAGG-CTCAA--ACCCTCTTATTTC----------TAGAACTACAGGAATTGAACCTGCTCCTGAGAATTCAAAATCCTCCGTGCTACCGAATT-ACACCATGTCCTACAA------GTAAGGTCAGCTAAAT-AAGCTATCGG
A-E_caballus : GATAGAGTAAAACATAGAGG-CTCAA--ACCCTCTTATTTC----------TAGAACTACAGGAATTGAACCTGCTCCTGAGAATTCAAAATCCTCCGTGCTACCGAATT-ACACCATGTCCTACAA------GTAAGGTCAGCTAAAT-AAGCTATCGG
R-C_bactrianus : GATAGAGTAAATAATAGAGG-TTTGA--GCCCTCTTGTTTC----------TAGAACCGTAGGAATTGAACCTACCCCTAAGAATTCAAAATTCTTCGTGCTACCACGCT-ACACCACATTCTATA-------GTAAGGTCAGCTAAAT-AAGCTATCGG
A-C_bactrianus : GATAGAGTAAATAATAGAGG-TTTGA--GCCCTCTTGTTTC----------TAGAACCGTAGGAATTGAACCTACCCCTAAGAATTCAAAATTCTTCGTGCTACCACGCT-ACACCACATTCTATA-------GTAAGGTCAGCTAAAT-AAGCTATCGG
R-C_lupus_familiaris : GATAGAGTAAATAATAGAGG-TTTAA--ATCCTCTTATTTC----------TAGAATAATAGGCTTCGAACCTAATCTTAAGAATTCAAAGATCTTCGTGCTACCAAACTTACACTATATTCTACA-------GTAAGGTCAGCTAAATTAAGCTATCGG
A-C_lupus_familiaris : GATAGAGTAAATAATAGAGG-TTTAA--ATCCTCTTATTTC----------TAGAATAATAGGCTTCGAACCTAATCTTAAGAATTCAAAGATCTTCGTGCTACCAAACTTACACTATATTCTACA-------GTAAGGTCAGCTAAATTAAGCTATCGG
R-N_procyonoides : GATAGAGTAAAGCATAGAGG-TTCAA--GCCCTCTTATTTC----------TAGAATAATAGGGCTTGAACCTAATCCTAAGAATTCAAAGATCTTCGTGCTACCAAATTTACACCATATTCTAAA-------GTAAGGTCAGCTAAATTAAGCTATCGG
A-N_procyonoides : GATAGAGTAAAGCATAGAGG-TTCAA--GCCCTCTTATTTC----------TAGAATAATAGGGCTTGAACCTAATCCTAAGAATTCAAAGATCTTCGTGCTACCAAATTTACACCATATTCTAAA-------GTAAGGTCAGCTAAATTAAGCTATCGG
R-V_lagopus : GATAGAGTAAATCATAGAGG-TTTAA--ACCCTCTTATTTC----------TAGAATAATAGGCCTCGAACCTAATCCTAAGAATTCAAAGATCTTCGTGCTACCAAATTTACACCATATTCTACA-------GTAAGGTCAGCTAAATTAAGCTATCGG
A-V_lagopus : GATAGAGTAAATCATAGAGG-TTTAA--ACCCTCTTATTTC----------TAGAATAATAGGCCTCGAACCTAATCCTAAGAATTCAAAGATCTTCGTGCTACCAAATTTACACCATATTCTACA-------GTAAGGTCAGCTAAATTAAGCTATCGG
R-M_putorius : GATAGAGTAAATCATAGAGG-TTCAA--ACCCTCTTATTTC----------TAGAACTAAAGGAATCGAACCTAATCCTAAGAACTCAAAAATCTTCGTGCTACCAAATTTACACCAAATTCTAAA-------GTAAGGTCAGCTAA-TTAAGCTATCGG
A-M_putorius : GATAGAGTAAATCATAGAGG-TTCAA--ACCCTCTTATTTC----------TAGAACTAAAGGAATCGAACCTAATCCTAAGAACTCAAAAATCTTCGTGCTACCAAATTTACACCAAATTCTAAA-------GTAAGGTCAGCTAA-TTAAGCTATCGG
R-B_taurus : GATAGAGTAAATAATAGAGG-TTCAA--ACCCTCTTATTTC----------TAGAACTATAGGAATCGAACCTACTCCTAAGAATCCAAAACTCTTCGTGCTCCCAAT-T-ACACCAAATTCTATTA------GTAAGGTCAGCTAA-TTAAGCTATCGG
A-B_taurus : GATAGAGTAAATAATAGAGG-TTCAA--ACCCTCTTATTTC----------TAGAACTATAGGAATCGAACCTACTCCTAAGAATCCAAAACTCTTCGTGCTCCCAAT-T-ACACCAAATTCTATTA------GTAAGGTCAGCTAA-TTAAGCTATCGG
R-O_aries : GATAGAGTAAATAATAGAGG-TTTAA--ATCCTCTTATTTC----------TAGAACTATAGGAATTGAACCTACTCCTAAGAACCCAAAACTCTTCGTGCTCCCAAT-T-ACACCAAATTCTAATA------GTAAGGTCAGCTAA-TTAAGCTATCGG
A-O_aries : GATAGAGTAAATAATAGAGG-TTTAA--ATCCTCTTATTTC----------TAGAACTATAGGAATTGAACCTACTCCTAAGAACCCAAAACTCTTCGTGCTCCCAAT-T-ACACCAAATTCTAATA------GTAAGGTCAGCTAA-TTAAGCTATCGG
R-S_scrofa : GATAGAGTAAAAAATAGAGG-TTCAA--ACCCTCTTATTTC----------TAGAACAATAGGACTCGAACCTAAACCTGAGAATTCAAAATTCTCCGTGCTACCAAAAT-ACACCACATTCTA-CA------GTAAGGTCAGCTAAGCTAAGCTATCGG
A-S_scrofa : GATAGAGTAAAAAATAGAGG-TTCAA--ACCCTCTTATTTC----------TAGAACAATAGGACTCGAACCTAAACCTGAGAATTCAAAATTCTCCGTGCTACCAAAAT-ACACCACATTCTA-CA------GTAAGGTCAGCTAAGCTAAGCTATCGG
R-O_cuniculus : GATAGAGTAAATAATAGAGGATCTTA--GCCCTCTTATTTC----------TAGAGCTATAGGACTTGAACCCACTCTCAAGAACTCAAAATTCTTCGTGCTACCATGT--ACACCAAGCTCTAAACCACTAAGTAAGGTCAGCTAAAT-AAGCTATCGG
A-O_cuniculus : GATAGAGTAAATAATAGAGGATCTTA--GCCCTCTTATTTC----------TAGAGCTATAGGACTTGAACCCACTCTCAAGAACTCAAAATTCTTCGTGCTACCATGT--ACACCAAGCTCTAAACCACTAAGTAAGGTCAGCTAAAT-AAGCTATCGG
R-M_musculus : GATAGAGTAAATTATAGAGG-TTCAA--GCCCTCTTATTTC----------TAGGACAATAGGAATTGAACCTACACTTAAGAATTCAAAATTCTCCGTGCTACCTAA---ACACCTTATCCTA-AT-----AGTAAGGTCAGCTAA-TTAAGCTATCGG
A-M_musculus : GATAGAGTAAATTATAGAGG-TTCAA--GCCCTCTTATTTC----------TAGGACAATAGGAATTGAACCTACACTTAAGAATTCAAAATTCTCCGTGCTACCTAA---ACACCTTATCCTA-AT-----AGTAAGGTCAGCTAA-TTAAGCTATCGG
R-R_norvegicus : GATAGAGTAAATAATAGAGG-TTTAA--ATCCTCTTATTTC----------TAGGACAATAGGAATTGAACCTACACCTAAGAATTCAAAATTCTCCGTGCTACCAAT---ACACCCTATCCTACAT-----AGTAAGGTCAGCTAA-CTAAGCTATCGG
A-R_norvegicus : GATAGAGTAAATAATAGAGG-TTTAA--ATCCTCTTATTTC----------TAGGACAATAGGAATTGAACCTACACCTAAGAATTCAAAATTCTCCGTGCTACCAAT---ACACCCTATCCTACAT-----AGTAAGGTCAGCTAA-CTAAGCTATCGG
R-M_coypus : GATAGAGTAAATAATAGAGG-TTTAA--GTCCTCTTATTTC----------TAGAAAAATAGGAATTGAACCTAACCTAAAGAACTCAAAATTCTTCGTGCTACCCCT---ACACTATATTCTA-TT-----AGTAAGGTCAGCTAAATTAAGCTATTGG
A-M_coypus : GATAGAGTAAATAATAGAGG-TTTAA--GTCCTCTTATTTC----------TAGAAAAATAGGAATTGAACCTAACCTAAAGAACTCAAAATTCTTCGTGCTACCCCT---ACACTATATTCTA-TT-----AGTAAGGTCAGCTAAATTAAGCTATTGG
R-A_platyrhynchos : GATAAAGTGAAC-ATAGAGG-TACAACAGCCCTCTCATTTCCTATTGACCTTAGAAAAGTAGGAATTGAACCTACACAGAAGAGATCAAAACTCTCCATACTTCCCTT---ATATTATTTTCTA---------GTAGAGTCAGCTAA-TCAAGCTACCGG
A-A_platyrhynchos : GATAAAGTGAAC-ATAGAGG-TACAACAGCCCTCTCATTTCCTATTGACCTTAGAAAAGTAGGAATTGAACCTACACAGAAGAGATCAAAACTCTCCATACTTCCCTT---ATATTATTTTCTA---------GTAGAGTCAGCTAA-TCAAGCTACCGG
R-G_gallus : GATAAAGTGAAC-ATAGAGG-TATAACAACCCTCTCACTTCC--TTAATCCTAGAAAAGTAGGAATCGAACCTACACAGAAGAGATCAAAACTCTTCATACTCCCTCT---ATATTATTTTCTA---------GTAAGGTCAGCTAA-CTAAGCTATCGG
A-G_gallus : GATAAAGTGAAC-ATAGAGG-TATAACAACCCTCTCACTTCC--TTAATCCTAGAAAAGTAGGAATCGAACCTACACAGAAGAGATCAAAACTCTTCATACTCCCTCT---ATATTATTTTCTA---------GTAAGGTCAGCTAA-CTAAGCTATCGG
 GATAGAGTAAATAATAGAGGATTTAACAACCCTCTTATTTCCTATTRAYCYTAGAACAATAGGAATTGAACCTACTCCTAAGAATTCAAAATTCTTCGTGCTACCAAATTTACACCATATTCTAMAACACTAAGTAAGGTCAGCTAAATTAAGCTATCGG

 * 5460 * 5480 * 5500 * 5520 * 5540 * 5560 * 5580 * 5600
R-E_caballus : GCCCATACCCCGAAAATGTTGGATTACACCCTTCCCGTACTAATAAATCCCCTTATCTTCACAACTATTCTAATAACAGTTCTTCTAGGAACTATAATCGTTATAATAAGCTCACACTGACTAATAATCTGAATCGGATTTGAAATAAATCTACTAGCCA
A-E_caballus : GCCCATACCCCGAAAATGTTGGATTACACCCTTCCCGTACTAATAAACCCCCTTATCTTCACAACTATTCTAATAACAGTTCTTCTAGGAACTATAATCGTTATAATAAGCTCACACTGACTAATAATCTGAATCGGATTTGAAATAAATCTACTAGCCA
R-C_bactrianus : GCCCATACCCCGAAAATGTTGGTTTATACCCTTCCCGTACTAATAAATCCTCTAATTCTCGGCATCATTCTACTCACAATTATAGCAGGAACTTTAATTGTTATAATTAGCTCTCACTGACTGTTCATTTGAATCGGCTTCGAAATAAATATACTCGCCA
A-C_bactrianus : GCCCATACCCCGAAAATGTTGGTTTATACCCTTCCCGTACTAATAAATCCTCTAATTCTCGGCATCATTCTACTCACAATTATAGCAGGAACTTTAATTGTTATAATTAGCTCTCACTGACTGTTCATTTGAATCGGCTTCGAAATAAATATACTCGCCA
R-C_lupus_familiaris : GCCCATACCCCGAAAATGTTGGTTTATACCCTTCCCGTACTAATAAAACCCCCTATTCTCATTATCATCATAGCAACTATCATGACAGGAACCATAATCGTCATACTAAGCTCGCACTGATTACTGATCTGAATTGGATTCGAAATAAACATGCTAGCCA
A-C_lupus_familiaris : GCCCATACCCCGAAAATGTTGGTTTATACCCTTCCCGTACTAATAAAACCCCCTATTCTCATTATCATCATAGCAACTATCATGACAGGAACCATAATCGTCATACTAAGCTCGCACTGATTACTGATCTGAATTGGATTCGAAATAAACATGCTAGCCA
R-N_procyonoides : GCCCATACCCCGAAAATGTTGGTTTATACCCTTCCCATACTAATAAAGCCCCCAATCCTCATCACCATCTTGGCAACAGTATTAACCGGAACCGTGATTGTCATAATAAGCTCACACTGATTACTAATATGAATAGGATTCGAAATAAATATACTAGCTG
A-N_procyonoides : GCCCATACCCCGAAAATGTTGGTTTATACCCTTCCCATACTAATAAAGCCCCCAATCCTCATCACCATCTTGGCAACAGTATTAACCGGAACCGTGATTGTCATAATAAGCTCACACTGATTACTAATATGAATAGGATTCGAAATAAATATACTAGCTG
R-V_lagopus : GCCCATACCCCGAAAATGTTGGTTTATATCCTTCCCGTACTAATAAAACCTCCTATCCTCATTACAATTCTAACAACCGTCATGGCCGGAACTATGATCGTACTATTAAGCTCCCACTGATTGCTGATCTGGATCGGATTTGAAATAAATATGTTAGCCG
A-V_lagopus : GCCCATACCCCGAAAATGTTGGTTTATATCCTTCCCGTACTAATAAAACCTCCTATCCTCATTACAATTCTAACAACCGTCATGGCCGGAACTATGATCGTACTATTAAGCTCCCACTGATTGCTGATCTGGATCGGATTTGAAATAAATATGTTAGCCG
R-M_putorius : GCCCATACCCCGAAAATGTTGGTTTATCCCCTTCCCGTACTAATTAAACCCCCTATTCTCACCATTATCATATTTACTATTATCTCAGGGACTATCATAGTACTAATAAGTTCCCACTGATTAACAATTTGAATCGGATTTGAAATAAACATACTAGCCA
A-M_putorius : GCCCATACCCCGAAAATGTTGGTTTATCCCCTTCCCGTACTAATTAAACCCCCTATTCTCACCATTATCATATTTACTATTATCTCAGGGACTATCATAGTACTAATAAGTTCCCACTGATTAACAATTTGAATCGGATTTGAAATAAACATACTAGCCA
R-B_taurus : GCCCATACCCCGAAAATGTTGGTTTATATCCTTCCCGTACTAATAAACCCAATTATCTTTATTATTATTCTACTAACCATTATACTAGGAACTATTATTGTCATAATCAGTTCTCACTGACTACTTGTCTGAATCGGGTTTGAAATAAATATACTCGCCA
A-B_taurus : GCCCATACCCCGAAAATGTTGGTTTATATCCTTCCCGTACTAATAAACCCAATTATCTTTATTATTATTCTACTAACCATTATACTAGGAACTATTATTGTCATAATCAGTTCTCACTGACTACTTGTCTGAATCGGGTTTGAAATAAATATACTCGCCA
R-O_aries : GCCCATACCCCGAAAATGTTGGTTCATATCCTTCCCGTACTAATAAATCCAATTATCCTCATTATTATTCTAATAACCGTTATACTTGGAACCATTATCGTTATGATTAGCACCCACTGATTGCTCATCTGAATTGGATTTGAAATAAATATACTTGCTA
A-O_aries : GCCCATACCCCGAAAATGTTGGTTCATATCCTTCCCGTACTAATAAATCCAATTATCCTCATTATTATTCTAATAACCGTTATACTTGGAACCATTATCGTTATGATTAGCACCCACTGATTGCTCATCTGAATTGGATTTGAAATAAATATACTTGCTA
R-S_scrofa : GCCCATACCCCGAAAATGTTGGTTCATACCCTTCCCATACTAATAAATCCCATTATCTACACTACCCTTATCATAACAGTAATATCCGGAACCATACTAGTAATAATCAGCTCACACTGACTACTCATCTGAATCGGATTCGAAATAAACCTATTAGCAA
A-S_scrofa : GCCCATACCCCGAAAATGTTGGTTCATACCCTTCCCATACTAATAAATCCCATTATCTACACTACCCTTATCATAACAGTAATATCCGGAACCATACTAGTAATAATCAGCTCACACTGACTACTCATCTGAATCGGATTCGAAATAAACCTATTAGCAA
R-O_cuniculus : GCCCATACCCCGAAAATGTTGGTTTATATCCTTCCCGTACTAATTAACCCCCTAACTCTTATTATTATCATATTTACTCTATTTATAGGCACGATAATCACAGTATTTAGCTCCCACTGACTAACTATATGAATCGGATTAGAAATAAACATACTAGCAA
A-O_cuniculus : GCCCATACCCCGAAAATGTTGGTTTATATCCTTCCCGTACTAATTAACCCCCTAACTCTTATTATTATTATATTTACTCTATTTATAGGCACGATAATCACAGTATTTAGCTCCCACTGACTAACTATATGAATCGGATTAGAAATAAACATACTAGCAA
R-M_musculus : GCCCATACCCCGAAAACGTTGGTTTAAATCCTTCCCGTACTAATAAATCCTATCACCCTTGCCATCATCTACTTCACAATCTTCTTAGGTCCTGTAATCACAATATCCAGCACCAACCTAATACTAATATGAGTAGGCCTGGAATTCAGCCTACTAGCAA
A-M_musculus : GCCCATACCCCGAAAACGTTGGTTTAAATCCTTCCCGTACTAATAAATCCTATCACCCTTGCCATCATCTACTTCACAATCTTCTTAGGTCCTGTAATCACAATATCCAGCACCAACCTAATACTAATATGAGTAGGCCTGGAATTCAGCCTACTAGCAA
R-R_norvegicus : GCCCATACCCCGAAAATGTTGGTCTAAACCCTTCCCGTACTAATAAACCCAATCACCCTAATCATTATTTACTTTACTATCCTCATAGGGCCTGTAATCACTATATCTAGCTCCAACTTACTCCTAATATGAGTAGGATTGGAAATAAGCCTTTTAGCTA
A-R_norvegicus : GCCCATACCCCGAAAATGTTGGTCTAAACCCTTCCCGTACTAATAAACCCAATCACCCTAATCATTATTTACTTTACTATCCTCATAGGGCCTGTAATCACTATATCTAGCTCCAACTTACTCCTAATATGAGTAGGATTGGAAATAAGCCTTTTAGCTA
R-M_coypus : GCCCATACCCCAAAAATGTTGGATTAAACCCTTCCCGTACTAATTAACATCACAATAATAACCATTATTTATTCAACCCTTATTATAGGCACATTAATTACCTTAATTAGTTCCCATTGATTATTAATATGAGTAGGATTAGAATTGAGCATAATATCAA
A-M_coypus : GCCCATACCCCAAAAATGTTGGATTAAACCCTTCCCGTACTAATTAACATCACAATAATAACCATTATTTATTCAACCCTTATTATAGGCACATTAATTACCTTAATTAGTTCCCATTGATTATTAATATGAGTAGGATTAGAATTGAGCATAATATCAA
R-A_platyrhynchos : GCCCATACCCCGGAAATGATGGTTCAACCCCCTCCTCTACTAATGAACCCCCATGCAACCCCAGTCCTAGTCCTCAGTCTCGCATTAGGCACAACAATCACAATCTCTAGCAACCACTGAGTCCTAGCCTGAACCGGACTAGAAATTAACACACTAGCCA
A-A_platyrhynchos : GCCCATACCCCGGAAATGATGGTTCAACCCCCTCCTCTACTAATGAACCCCCATGCAACCCCAGTCCTAGTCCTCAGTCTCGCATTAGGCACAACAATCACAATCTCTAGCAACCACTGAGTCCTAGCCTGAACCGGACTAGAAATTAACACACTAGCCA
R-G_gallus : GCCCATACCCCGAAAATGATGGTTTAACCCCTTCCCCTACTAATGAACCCCCATGCAAAACTAATCTGCACAGTAAGCCTCATCATGGGAACCAGCATCACAATCTCCAGCAACCATTGAATCTTAGCCTGAACAGGCTTAGAGATCAACACCTTAGCCA
A-G_gallus : GCCCATACCCCGAAAATGATGGTTTAACCCCTTCCCCTACTAATGAACCCCCATGCAAAACTAATCTGCACAGTAAGCCTCATCATGGGAACCAGCATCACAATCTCCAGCAACCATTGAATCTTAGCCTGAACAGGCTTAGAGATCAACACCTTAGCCA
 GCCCATACCCCGAAAATGTTGGTTTATACCCTTCCCGTACTAATAAACCCCCTTATCCTCATYATYATTMTATTAACHATYATAATAGGAACTATAATCGTAATAATTAGCTCCCACTGAYTACTAATCTGAATCGGATTTGAAATAAACATACTAGCCA

 * 5620 * 5640 * 5660 * 5680 * 5700 * 5720 * 5740 * 5760
R-E_caballus : TTATCCCTATCCTAATAAAAAAGTACAATCCCCGAACCATAGAAGCCTCCACCAAATATTTTCTAACCCAAGCCACCGCATCAATACTCCTCATAATAGCGATCATCATTAACC-TCATACACTCAGGCCAATGAACAATCACAAAAG------TCTTCA
A-E_caballus : TTATCCCCATCCTAATAAAAAAGTACAATCCCCGAACCATAGAAGCCTCCACCAAATATTTTCTAACCCAAGCCACCGCATCAATACTCCTCATAATAGCGATCATCATTAACC-TCATACACTCAGGCCAATGAACAATCACAAAAG------TCTTCA
R-C_bactrianus : CCATCCCCATCCTAATAAAAAACTTCAGCCCCCGATCCATTGAGGCTTCTACCAAATATTTCCTAACCCAAGCCACCGCATCAATGCTACTTATGTTAGGAGTAATTATTAATC-TTCTATACTCAGGACAATGAACCACCACAAAAA------TATTCA
A-C_bactrianus : CCATCCCCATCCTAATAAAAAACTTCAGCCCCCGATCCATTGAGGCTTCTACCAAATATTTCCTAACCCAAGCCACCGCATCAATGCTACTTATGTTAGGAGTAATTATTAATC-TTCTATACTCAGGACAATGAACCACCACAAAAA------TATTCA
R-C_lupus_familiaris : TCATCCCTATTCTCATAAAAAAGTACAATCCACGAGCCATAGAGGCCTCTACAAAATATTTTCTTACACAAGCTACAGCCTCAATATTACTAATAATAGGAGTCACTATCAACC-TCCTTTACTCCGGCCAATGGGTAATCTCAAAAA------TCTCAA
A-C_lupus_familiaris : TCATCCCTATTCTCATAAAAAAGTACAATCCACGAGCCATAGAGGCCTCTACAAAATATTTTCTTACACAAGCTACAGCCTCAATATTACTAATAATAGGAGTCACTATCAACC-TCCTTTACTCCGGCCAATGGGTAATCTCAAAAA------TCTCAA
R-N_procyonoides : TCATACCTATCCTGATAAAAAAGTTTAACCCCCGGGCCGTGGAAGCATCCACAAAATATTTCCTTACACAAGCAACCGCTTCCATAGTATTTATAATAGGAGTCACCATTAACC-TTCTCTATTCCGGCCAATGGGTGGTATCAAAAG------TCTCAA
A-N_procyonoides : TCATACCTATCCTGATAAAAAAGTTTAACCCCCGGGCCGTGGAAGCATCCACAAAATATTTCCTTACACAAGCAACCGCTTCCATAGTATTTATAATAGGAGTCACCATTAACC-TTCTCTATTCCGGCCAATGGGTGGTATCAAAAG------TCTCAA
R-V_lagopus : TTATTCCTATTCTAATAAAAAAATTCAACCCACGGGCTATAGAAGCATCCACAAAATATTTCCTTACACAAGCAACAGCCTCAATGCTACTAATAATAGGAGTCACTATCAACC-TTCTCTACTCCGGTCAATGAGCAATCTCAAAAA------TCTCGA
A-V_lagopus : TTATTCCTATTCTAATAAAAAAATTCAACCCACGGGCTATAGAAGCATCCACAAAATATTTCCTTACACAAGCAACAGCCTCAATGCTACTAATAATAGGAGTCACTATCAACC-TTCTCTACTCCGGTCAATGAGCAATCTCAAAAA------TCTCGA
R-M_putorius : TTATTCCTATCCTAATAAAAAAATTCAGCCCACGAGCAATTGAAGCAGCCACAAAATATTTCCTCACCCAAGCCACTGCATCTATACTCCTAATACTAGGAATTATCATAAACC-TATTATTAACAGGACAATGAACAGCCCTAAACA------TCCTAA
A-M_putorius : TTATTCCTATCCTAATAAAAAAATTCAGCCCACGAGCAATTGAAGCAGCCACAAAATATTTCCTCACCCAAGCCACTGCATCTATACTCCTAATACTAGGAATTATCATAAACC-TATTATTAACAGGACAATGAACAGCCCTAAACA------TCCTAA
R-B_taurus : TCATCCCCATCATAATAAAAAATCACAACCCACGAGCTACAGAAGCATCAACTAAATATTTTTTGACTCAATCAACAGCCTCAATACTACTAATAATAGCCGTCATCATTAACG-TAATATTCTCAGGCCAATGAACCGTAATAAAAC------TATTTA
A-B_taurus : TCATCCCCATCATAATAAAAAATCACAACCCACGAGCTACAGAAGCATCAACTAAATATTTTTTGACTCAATCAACAGCCTCAATACTACTAATAATAGCCGTCATCATTAACC-TAATATTCTCAGGCCAATGAACCGTAATAAAAC------TATTTA
R-O_aries : TTATTCCCATTATAATAAAAAAGCACAACCCACGAGCCACAGAAGCATCAACCAAATATTTCCTAACTCAATCAACAGCCTCAATACTACTAATAATAGCCATTATCATTAACT-TAATATTCTCAGGCCAATGAACCGTAATAAAAC------TATTTA
A-O_aries : TTATTCCCATTATAATAAAAAAGCACAACCCACGAGCCACAGAAGCATCAACCAAATATTTCCTAACTCAATCAACAGCCTCAATACTACTAATAATAGCCATTATCATTAACT-TAATATTCTCAGGCCAATGAACCGTAATAAAAC------TATTTA
R-S_scrofa : TAATCCCAGTATTAATAAAAAATTTTAACCCACGAGCCACAGAAGCAGCCACAAAATATTTCCTAACACAAGCCACAGCCTCCATGATACTAATAATAGCCATCATCATCAACC-TCCTATATTCTGGCCAATGGACCATTACAAAAA------TATTTA
A-S_scrofa : TAATCCCAGTATTAATAAAAAATTTTAACCCACGAGCCACAGAAGCAGCCACAAAATATTTCCTAACACAAGCCACAGCCTCCATGATACTAATAATAGCCATCATCATCAACC-TCCTATATTCTGGCCAATGGACCATTACAAAAA------TATTTA
R-O_cuniculus : TTATCCCTATTCTAATCAACAAAGCCACCCCACGATCAACAGAAGCTGCAACCAAGTATTTTCTAACACAAGCCACAGCATCTATAATCTTAATAATAGCTATTACACTTAATA-TCCTTGACTCCGGACAATGAACCCTAATTAATC------CACAGA
A-O_cuniculus : TTATCCCTATTCTAATCAACAAAGCCACCCCACGATCAACAGAAGCTGCAACCAAATATTTTCTAACACAAGCCACAGCATCTATAATCTTAATAATAGCTATTACACTTAATA-TCCTTGACTCCGGACAATGAACCCTAATTAATC------CACAGA
R-M_musculus : TTATCCCCATACTAATCAACAAAAAAAACCCACGATCAACTGAAGCAGCAACAAAATACTTCGTCACACAAGCAACAGCCTCAATAATTATCCTCCTGGCCATCGTACTCAACTATAA-ACAACTAGG--AAC-ATGAATATTTCAACAACAAACAAACG
A-M_musculus : TTATCCCCATACTAATCAACAAAAAAAACCCACGATCAACTGAAGCAGCAACAAAATACTTCGTCACACAAGCAACAGCCTCAATAATTATCCTCCTGGCCATCGTACTCAACTATAA-ACAACTAGG--AAC-ATGAATATTTCAACAACAAACAAACG
R-R_norvegicus : TCATCCCACTTCTAGCCAACAAAAAAAGCCCACGATCAACTGAAGCAGCAACAAAATATTTTCTAACCCAAGCTACAGCCTCAATAATTATCCTACTAGTCATCATCCTCAACTACAA-ACAATCAGG--AAT-ATGAACCCTCCAACAACAAACCAATA
A-R_norvegicus : TCATCCCACTTCTAGCCAACAAAAAAAGCCCACGATCAACTGAAGCAGCAACAAAATATTTTCTAACCCAAGCTACAGCCTCAATAATTATCCTACTAGTCATCATCCTCAACTACAA-ACAATCAGG--AAT-ATGAACCCTCCAACAACAAACCAATA
R-M_coypus : TTATTCCCATTCTAATAAATAAATCTAATCCTCGATCAACAGAAGCTGCTACAAAATATTTCCTCACACAAGCAACAGCATCAATAATTTTACTATTCTCTATTATTAT-AACAATACTATACTCCGGTCAATGATCAATTTTCTACT------CCAGCA
A-M_coypus : TTATTCCCATTCTAATAAATAAATCTAATCCTCGATCAACAGAAGCTGCTACAAAATATTTCCTCACACAAGCAACAGCATCAATAATTTTACTATTCTCTATTATTAT-AACAATACTATACTCCGGTCAGTGATCAATTTTCTACT------CCAGCA
R-A_platyrhynchos : TCATCCCCCTAATCTCCAAATCCCACCACCCGCGAGCAGTAGAAGCCGCGACAAAATACTTCTTGACACAGGCAGCTGCCTCCGCCCTAGTACTATTCTCCAGCATAACCAACG-CCTGAGCCACCGGCCAGTGAGACATCACACAAC------TTAACC
A-A_platyrhynchos : TCATCCCCCTAATCTCCAAATCCCACCACCCGCGAGCAGTAGAAGCCGCGACAAAATACTTCTTGACACAGGCAGCTGCCTCCGCCCTAGTACTATTCTCCAGCATAACCAACG-CCTGAGCCACCGGCCAGTGAGACATCACACAAC------TTAACC
R-G_gallus : TCATCCCCCTCATCTCCAAGTCACACCACCCCCGAGCGATTGAAGCCACTATCAAATATTTCCTCACCCAATCAACTGCATCAGCCCTAATCCTCTTCTCGAGCATAACCAACG-CCTGATCCACCGGACAATGAGACATTACACAAC------TAAACC
A-G_gallus : TCATCCCCCTCATCTCCAAGTCACACCACCCCCGAGCGATTGAAGCCACTATCAAATATTTCCTCACCCAATCAACTGCATCAGCCCTAATCCTCTTCTCGAGCATAACCAACG-CCTGATCCACCGGACAATGAGACATTACACAAC------TAAACC
 TYATCCCCATYCTAATAAAAAAATACAACCCACGAGCMATAGAAGCAKCMACAAAATATTTCCTAACACAAGCAACAGCCTCAATACTACTAATAATAGCCATCATCATCAACCATMCTATACTCMGGCCAATGAACMATCACAAAACAACAAATMTTCA

 * 5780 * 5800 * 5820 * 5840 * 5860 * 5880 * 5900 * 5920
R-E_caballus : ACCCCACAGCGTCCATCATTATAACTTCAGCTCTCGCCATAAAACTTGGACTCACACCATTCCACTTCTGAGTACCCGAAGTCACACAGGGCATCTCATTAACATCAGGTCTCATCCTACTTACATGACAAAAACTAGCCCCAATATCAATCCTATATCA
A-E_caballus : ACCCCACAGCGTCCATCATTATAACTTCAGCTCTCGCCATAAAACTTGGACTCACACCATTCCACTTCTGAGTACCCGAAGTCACACAGGGCATCTCATTAACATCAGGTCTCATCCTACTTACATGACAAAAACTAGCCCCAATATCAATCCTATATCA
R-C_bactrianus : ACCAAACCTCATCTATCATCATTACCACAGCCCTAACCATAAAACTAGGACTAGCCCCATTTCACTTCTGAGTGCCAGAAGTCACACAAGGAATCCCCCTGATATCAGGACTAATCCTATTAACATGACAAAAACTAGCCCCCCTCTCCGTACTATACCA
A-C_bactrianus : ACCAAACCTCATCTATCATCATTACCACAGCCCTAACCATAAAACTAGGACTAGCCCCATTTCACTTCTGAGTGCCAGAAGTCACACAAGGAATCCCCCTGATATCAGGACTAATCCTATTAACATGACAAAAACTAGCCCCCCTCTCCGTACTATACCA
R-C_lupus_familiaris : ACCCCATCGCATCCATCATGATAACCACTGCCCTAACAATAAAACTAGGCCTATCTCCATTCCACTTCTGAGTTCCCGAAGTAACACAGGGAATTACGCTCATATCAGGAATAATCCTACTAACATGACAAAAAATCGCACCTATATCCATCCTATATCA
A-C_lupus_familiaris : ACCCCATCGCATCCATCATGATAACCACTGCCCTAACAATAAAACTAGGCCTATCTCCATTCCACTTCTGAGTTCCCGAAGTAACACAGGGAATTACGCTCATATCAGGAATAATCCTACTAACATGACAAAAAATCGCACCTATATCCATCCTATATCA
R-N_procyonoides : ACCCCGCCGCATCCGCAATAATAACCATCGCCCTAACAATAAAACTAGGTCTATCTCCGTTCCACTTCTGAGTCCCAGAAGTAACACAAGGAATTTCACTCACATCTGGCATGATTCTACTAACATGACAAAAAATCGCACCCATATCCGTTCTCTACCA
A-N_procyonoides : ACCCCGCCGCATCCGCAATAATAACCATCGCCCTAACAATAAAACTAGGTCTATCTCCGTTCCACTTCTGAGTCCCAGAAGTAACACAAGGAATTTCACTCACATCTGGCATGATTCTACTAACATGACAAAAAATCGCACCCATATCCGTTCTCTACCA
R-V_lagopus : ACCCCGCAGCATCCACTATAATAACTATCGCCCTGACAATAAAATTGGGTCTATCCCCATTTCACTTCTGAGTTCCCGAAGTAACTCAGGGCATTTCACTCTTATCAGGCATAATCCTACTAACATGACAAAAAATTGCACCAATATCCGTTCTCTATCA
A-V_lagopus : ACCCCGCAGCATCCACTATAATAACTATTGCCCTGACAATAAAATTGGGTCTATCCCCATTTCACTTCTGAGTTCCCGAAGTAACTCAGGGCATTTCACTCTTATCAGGCATAATCCTACTAACATGACAAAAAATTGCACCAATATCCGTTCTCTATCA
R-M_putorius : ACCCAATCGTATCTAACATAATAACAGTAGCCTTATCAATAAAACTAGGATTATCGCCTTTCCACTTCTGAGTACCAGAAGTAACCCAAGGAATCCCGTTAATGTCAGGAATAATCCTACTAACTTGGCAAAAAATTGCCCCCCTATCTGTCCTATACCA
A-M_putorius : ACCCAATCGTATCTAACATAATAACAGTAGCCTTATCAATAAAACTAGGATTATCGCCTTTCCACTTCTGAGTACCAGAAGTAACCCAAGGAATCCCGTTAATGTCAGGAATAATCCTACTAACTTGGCAAAAAATTGCCCCCCTATCTGTCCTATACCA
R-B_taurus : GCCCAATAGCCTCAATACTTATAACGATAGCCCTAGCTATAAAACTAGGAATAGCCCCATTTCACTTCTGAGTCCCAGAAGTAACACAGGGCATCCCCCTATCCTCAGGCCTTATCCTACTGACATGACAAAAACTAGCACCTATATCTGTACTTTACCA
A-B_taurus : ACCCAATAGCCTCAATACTTATAACGATAGCCCTAGCTATAAAACTAGGAATAGCCCCATTTCACTTCTGAGTCCCAGAAGTAACACAGGGCATCCCCCTATCCTCAGGCCTTATCCTACTGACATGACAAAAACTAGCACCTATATCTGTACTTTACCA
R-O_aries : ATCCAATAGCCTCCATACTCATAACAATAGCCCTCGCTATAAAACTAGGTATAGCCCCATTCCACTTCTGAGTCCCAGAAGTAACACAAGGCATTCCCCTATCCTCAGGCCTAATCTTACTCACATGACAAAAACTAGCACCCATGTCAGTACTTTACCA
A-O_aries : ACCCAATAGCCTCCATACTCATAACAATAGCCCTTGCTATAAAACTAGGTATAGCCCCATTCCACTTCTGAGTCCCAGAAGTAACACAAGGCATTCCCCTATCCTCAGGCCTAATCTTACTCACATGACAAAAACTAGCACCCATGTCAGTACTTTACCA
R-S_scrofa : ACCCAGTAGCAATAACAATAATAACCCTGGCCCTAGCCATAAAACTAGGACTCTCACCTTTCCACTTCTGAGTCCCAGAAGTAACCCAAGGCATTTCACTACAAGCAGGCCTACTATTACTAACATGACAAAAACTAGCCCCATTATCAGTACTATGCCA
A-S_scrofa : ACCCAGTAGCAATAACAATAATAACCCTGGCCCTAGCCATAAAACTAGGACTCTCACCTTTCCACTTCTGAGTCCCAGAAGTAACCCAAGGCATTTCACTACAAGCAGGCCTACTATTACTAACATGACAAAAACTAGCCCCATTATCAGTACTATGCCA
R-O_cuniculus : ATCAACTTACTCCAGTCCTAATTACACTGGCACTAATCATTAAACTAGGAATAGCCCCCTTCCACTTCTGAGTACCAGAGGTCACCCAAGGAGTTCCCCTAAAATCAGGATTAATCCTTCTCACGTGACAAAAATTAGCCCCCCTATCCATCCTTTACCA
A-O_cuniculus : ATCAACTTACTCCAGTCCTAATTACACTGGCACTAATCATTAAACTAGGAATAGCCCCCTTCCACTTCTGAGTACCAGAGGTCACCCAAGGAGTTCCCCTAAAATCAGGATTAATCCTTCTCACGTGACAAAAATTAGCCCCCCTATCCATCCTTTACCA
R-M_musculus : GTCTTATCCTTAACATAACATTAA---TAGCCCTATCCATAAAACTAGGCCTCGCCCCATTCCACTTCTGATTACCAGAAGTAACTCAAGGGATCCCACTGCACATAGGACTTATTCTTCTTACATGACAAAAAATTGCTCCCCTATCAATTTTAATTCA
A-M_musculus : GTCTTATCCTTAACATAACATTAA---TAGCCCTATCCATAAAACTAGGCCTCGCCCCATTCCACTTCTGATTACCAGAAGTAACTCAAGGGATCCCACTGCACATAGGACTTATTCTTCTTACATGACAAAAAATTGCTCCCCTATCAATTTTAATTCA
R-R_norvegicus : ACATACTACTCAACATAATACTCA---TTTCACTAGCCATAAAACTTGGACTAGCCCCCTTCCACTACTGACTACCCGAAGTCACCCAAGGAATTCCCCTACACATTGGATTAATCTTACTAACATGACAAAAAATTGCTCCACTATCAATTCTATACCA
A-R_norvegicus : ACATACTACTCAACATAATACTCA---TTTCACTAGCCATAAAACTTGGACTAGCCCCATTCCACTACTGACTACCCGAAGTCACCCAAGGAATTCCCCTACACATTGGATTAATCTTACTAACATGACAAAAAATTGCTCCACTATCAATTCTATACCA
R-M_coypus : ATCCCCTCATCTCCCTAGCACTAACCTTATCATTAATCATAAAATTAGGTCTTGCCCCTTTTCACTTCTGAGTGACAGAAGTAACACAAGGAACCCCTTTAATCCCAGGAATAATCTTACTAACATGACAAAAAATCGCCCCTCTATCAATCCTAATCCA
A-M_coypus : ATCCCCTCATCTCCCTAGCACTAACCTTATCATTAATCATAAAATTAGGTCTTGCCCCTTTTCACTTCTGAGTGACAGAAGTAACACAAGGAACCCCTTTAATCCCAGGAATAATCTTACTAACATGACAAAAAATCGCCCCTCTATCAATCCTAATCCA
R-A_platyrhynchos : ACCCAACCTCATGTCTACTGCTCACAGCAGCAATCGCAATTAAATTAGGCCTGGTCCCATTTCACTTCTGATTCCCAGAAGTCCTACAAGGATCCCCCCTAATAACGGCCCTCCTACTCTCAACCCTCATAAAATTCCCCCCACTGACCCTCCTCCTAAT
A-A_platyrhynchos : ACCCAACCTCATGTCTACTGCTCACAGCAGCAATCGCAATTAAATTAGGCCTGGTCCCATTTCACTTCTGATTCCCAGAAGTCCTACAAGGATCCCCCCTAATAACGGCCCTCCTACTCTCAACCCTCATAAAATTCCCCCCACTGACCCTCCTCCTAAT
R-G_gallus : ACCCGACATCATGCCTAATATTAACAATAGCAATCGCAATCAAATTAGGACTAGTCCCATTCCACTTCTGATTCCCAGAAGTACTCCAAGGCTCCTCCCTAATCACTGCCCTACTACTCTCCACCCTAATAAAACTCCCCCCAATCACACTCCTCCTCCT
A-G_gallus : ACCCGACATCATGCCTAATATTAACAATAGCAATCGCAATCAAATTAGGACTAGTCCCATTCCACTTCTGATTCCCAGAAGTACTCCAAGGCTCCTCCCTAATCACTGCCCTACTACTCTCCACCCTAATAAAACTCCCCCCAATCACACTCCTCCTCCT
 ACCCAATMGCATCCATAATAATAACMATAGCCCTAGCCATAAAACTAGGACTAGCCCCATTCCACTTCTGAGTCCCAGAAGTAACACAAGGAATCCCCCTAATATCAGGMCTAATCCTACTAACATGACAAAAAATAGCCCCMMTATCAGTCCTATACCA

 * 5940 * 5960 * 5980 * 6000 * 6020 * 6040 * 6060 * 6080
R-E_caballus : AATCTCACCCTCAATTAACCTAAATATCTTATTAACTATAGCCGTACTGTCAATCCTAGTAGGAGGCTGAGGCGGTCTCAACCAAACCCAACTACGAAAAATCATAGCATACTCGTCAATCGCGCATATAGGATGAATAACAGCTGTCCTAGTATATAAC
A-E_caballus : AATCTCACCCTCAATTAACCTAAATATCTTATTAACTATAGCCGTACTGTCAATCCTAGTAGGAGGCTGAGGCGGTCTCAACCAAACCCAACTACGAAAAATCATAGCATACTCGTCAATCGCGCATATAGGATGAATAACAGCTGTCCTAGTATATAAC
R-C_bactrianus : AATCGCCCCGTCAATCAATCTAAACATACTGTTAACCATATCCTTACTGTCAATTATAGTTGGAGGCTGAGGCGGACTTAATCAAACGCAACTACGAAAAATCATGGCCTACTCATCAATTGGTCACATGGGCTGAATAACTGCAATTATAGTGTACAAC
A-C_bactrianus : AATCGCCCCGTCAATCAATCTAAACATACTGTTAACCATATCCTTACTGTCAATTATAGTTGGAGGCTGAGGCGGACTTAATCAAACGCAACTACGAAAAATCATGGCCTACTCATCAATTGGTCACATGGGCTGAATAACTGCAATTATAGTGTACAAC
R-C_lupus_familiaris : AATCTCTCCATCAATTAACACTAACCTTCTTATACTAATAGCCCTTACATCCGTTCTAGTAGGAGGCTGAGGCGGACTAAATCAAACTCAACTACGAAAAATCATAGCATACTCCTCCATTGCCCACATAGGCTGAATAGCCGCTATCATTACTTATAAC
A-C_lupus_familiaris : AATCTCTCCATCAATTAACACTAACCTTCTTATACTAATAGCCCTTACATCCGTTCTAGTAGGAGGCTGAGGCGGACTAAATCAAACTCAACTACGAAAAATCATAGCATACTCCTCCATTGCCCACATAGGCTGAATAGCCGCTATCATTACTTATAAC
R-N_procyonoides : AATTTCACCATCAGTCAACACCAACCTCCTGACATTGATAGCCCTCACGTCAATCCTTGTAGGGGGCTGAGGAGGATTAAACCAGACTCAATTACGAAAAATCATAGCATACTCCTCCATTGCCCACATGGGCTGAATAACAGCAATTATCATCTATAAC
A-N_procyonoides : AATTTCACCATCAGTCAACACCAACCTCCTGACATTGATAGCCCTCACGTCAATCCTTGTAGGGGGCTGAGGAGGATTAAACCAGACTCAATTACGAAAAATCATAGCATACTCCTCCATTGCCCACATGGGCTGAATAACAGCAATTATCATCTATAAC
R-V_lagopus : AATTTCACCATCTATCAATACTAACCTCATAATACTAATAGCCCTTACATCTGTCCTAATTGGAGGATGAGGCGGACTTAATCAAACTCAACTACGAAAAATCATAGCTTACTCCTCTATCGCGCACATAGGCTGAATAACAGCAATCATTATTTATAGC
A-V_lagopus : AATTTCACCATCTATCAATACTAACCTCATAATACTAATAGCCCTTACATCTGTCCTAATTGGAGGATGAGGCGGACTTAATCAAACTCAACTACGAAAAATCATAGCTTACTCCTCTATCGCGCACATAGGCTGAATAACAGCAATCATTATTTATAGC
R-M_putorius : AATAGCCCCCTCCATAAACACACACTTATTAATAATTATAGCATTCATATCTGTCTTAATTGGAGGATGGGGAGGCCTTAACCAAACACAACTACGAAAAATTCTAGCCTACTCATCAATCGCACACATAGGATGAATGATCGCCGTAACAACATATAAC
A-M_putorius : AATAGCCCCCTCCATAAACACACACTTATTAATAATTATAGCATCCATATCTGTCTTAATTGGAGGATGGGGAGGCCTTAACCAAACACAACTACGAAAAATTCTAGCCTACTCATCAATCGCACACATAGGATGAATGATCGCCGTAACAACATATAAC
R-B_taurus : AATCTTCCCATCAATTAACCTAAACTTAATTCTAACCCTATCAGTTTTATCAATCCTAATTGGAGGCTGAGGGGGACTAAACCAAACACAACTCCGAAAAATCATAGCCTACTCATCAATCGCTCATATAGGCTGAATAACAGCAGTACTACCATATAAC
A-B_taurus : AATCTTCCCATCAATTAACCTAAACTTAATTCTAACCCTATCAGTTTTATCAATCCTAATTGGAGGCTGAGGGGGACTAAACCAAACACAACTCCGAAAAATCATAGCCTACTCATCAATCGCTCATATAGGCTGAATAACAGCAGTACTACCATATAAC
R-O_aries : AATCCTTCCATCCATCAACCTAGACCTGATCCTAACCCTATCAATTCTATCTATTACAATCGGAGGCTGAGGAGGACTGAACCAAACCCAACTACGAAAAATTATAGCCTATTCATCAATTGCCCACATAGGCTGAATAACAGCAGTTTTACTATATAAT
A-O_aries : AATCCTTCCATCCATCAACCTAGACCTGATCCTAACCCTATCAATTCTATCTATTACAATCGGAGGCTGAGGAGGACTGAACCAAACCCAACTACGAAAAATCATAGCCTATTCATCAATTGCCCACATAGGCTGAATAACAGCAGTTTTACTATATAAT
R-S_scrofa : AATCTCACAATCAATCAACCCAAACCTAATATTAACTATGGCCATATTATCAATTTTAATCGGAGGGTGAGGAGGACTAAACCAAACCCAACTTCGAAAAATCATAGCATATTCATCAATCGCACACATAGGATGAATGACAGCAGTATTACCATATAAC
A-S_scrofa : AATCTCACAATCAATCAACCCAAACCTAATATTAACTATGGCCATATTATCAATTTTAATCGGAGGGTGAGGAGGACTAAACCAAACCCAACTTCGAAAAATCATAGCATATTCATCAATCGCACACATAGGATGAATGACAGCAGTATTACCATATAAC
R-O_cuniculus : AATCTCCCCATCAATTAATCCAACTATAATAATATCAGTAGCCATTCTATCAATTATAGTTGGCGGTTGAGGTGGACTAAACCAAACCCAACTACGAAAAATCCTAGCATACTCTTCAATCGCCCACATAGGATGAATAGCAGCTATCATCACATTTAAC
A-O_cuniculus : AATCTCCCCATCAATTAATCCAACTATAATAATATCAGTAGCCATTCTATCAATTATAGTTGGCGGTTGAGGTGGACTAAACCAAACCCAACTACGAAAAATCCTAGCATACTCTTCAATCGCCCACATAGGATGAATAGCAGCTATCATCACATTTAAC
R-M_musculus : AATTTACCCGCTACTCAACTCTACTATCATTTTAATACTAGCAATTACTTCTATTTTCATAGGGGCATGAGGAGGACTTAACCAAACACAAATACGAAAAATTATAGCCTATTCATCAATTGCCCACATAGGATGAATATTAGCAATTCTTCCTTACAAC
A-M_musculus : AATTTACCCGCTACTCAACTCTACTATCATTTTAATACTAGCAATTACTTCTATTTTCATAGGGGCATGAGGAGGACTTAACCAAACACAAATACGAAAAATTATAGCCTATTCATCAATTGCCCACATAGGATGAATATTAGCAATTCTTCCTTACAAC
R-R_norvegicus : ATTTTATCAACTCCTAAACCCAACTATCACCACCATTCTCGCAATTTCATCAGTCTTTGTTGGCGCCTGAGGAGGACTTAACCAGACCCAAACACGAAAAATCATAGCATATTCATCAATTGCCCACATAGGATGAATAACAGCAATCCTTCCATACAAC
A-R_norvegicus : ATTTTATCAACTCCTAAGCCCAACTATCACCACCATTCTCGCAATTTCATCAGTCTTTGTTGGCGCCTGAGGAGGACTTAACCAGACCCAAACACGAAAAATCATAGCATATTCATCAATTGCCCACATAGGATGAATAACAGCAATCCTTCCATACAAC
R-M_coypus : AACCTCCTCAACAATTAATCAACCCTTAATTATTATTTCCGCATTACTATCTACCCTTTTAGGAGGCTGAGGGGGTCTAAACCAAACACAGCTACGAAAAATCTTAGCATATTCCTCTATCGCCCATATAGGCTGAATATTAGTAGTACTAACATATAAC
A-M_coypus : AACCTCCTCAACAATTAATCAACCCTTAATTATTATTTCCGCATTACTATCTACCCTTTTAGGAGGCTGAGGGGGTCTAAACCAAACACAGCTACGAAAAATCTTAGCATATTCCTCTATCGCCCATATAGGCTGAATATTAGTAGTACTAACATATAAC
R-A_platyrhynchos : GACATCTAAATCTCTCAACCCAGCCCTACTTACCGCAATAGCCCTGGCCTCAGCAGCATTGGGAGGCTGAATAGGACTAAATCAAACACAAACACGCAAAATCCTAGCCTTCTCATCCATCTCCCACCTAGGCTGAATCGCCATCATCCTAGTCTACAGC
A-A_platyrhynchos : GACATCTAAATCTCTCAACCCAGCCCTACTTACCGCAATAGCCCTGGCCTCAGCAGCATTGGGAGGCTGAATAGGACTAAATCAAACACAAACACGCAAAATCCTAGCCTTCTCATCCATCTCCCACCTAGGCTGAATCGCCATCATCCTAGTCTACAGC
R-G_gallus : AACATCACAGTCTCTTAATACCACCTTACTCACCCTCCTAGCAATCTCCTCCACCCTAATCGGAGGCTGAATGGGCCTAAACCAAACACAAACACGAAAAATCCTAGCCTTCTCATCCATCTCCCATTTAGGATGAATAATTATAATTATCTCCTATAAC
A-G_gallus : AACATCACAGTCTCTTAATACCACCTTACTCACCCTCCTAGCAATCTCCTCCACCCTAATCGGAGGCTGAATGGGCCTAAACCAAACACAAACACGAAAAATCCTAGCCTTCTCATCCATCTCCCATTTAGGATGAATAATTATAATTATCTCCTATAAC
 AATCTCCCCATCAATCAACCCAAACCTAATWATAATWATAGCCATTMTATCAATCCTAATTGGAGGCTGAGGAGGACTAAACCAAACACAACTACGAAAAATCATAGCMTACTCATCAATCGCCCACATAGGCTGAATAACAGCAATCATAACATATAAC

 * 6100 * 6120 * 6140 * 6160 * 6180 * 6200 * 6220 * 6240
R-E_caballus : CCAACACTAACAATACTAAACATATTAATTTACATTATAATAACACTCACAATATTCATACTATTTATCCACAGCT----CCTCTACTA--CAACACTATCACTCTCCCACA---CATGAAACAAAATACCTCTAACCACTACACTAATCTTAATTACCT
A-E_caballus : CCAACACTGACAATACTAAACATATTAATTTACATTATAATAACACTCACAATATTCATACTATTTATCCACAGCT----CCTCTACTA--CAACACTATCACTCTCCCACA---CATGAAACAAAACACCTCTAACCACTACACTAATCCTAATTACCT
R-C_bactrianus : CCCACTATAGCAATGCTATACCTATTAATATATTTAACAATAACACTCACAATATTTATACTATTCATAATCAACT----CCACTACAA--CCCTTCTCTCCCTATCACACA---CCTGAAACAAGACTCCCATCATTACAATAATTGTCCTCACCGTCA
A-C_bactrianus : CCCACTATAGCAATGCTATACCTATTAATATATTTAACAATAACACTCACAATATTTATACTATTCATAATCAACT----CCACTACAA--CCCTTCTCTCCCTATCACACA---CCTGAAACAAGACTCCCATCATTACAATAATTGTCCTCACCGTCA
R-C_lupus_familiaris : CCTACAATAATAGTTCTAAACTTAACTTTATATATTCTAATAACACTATCTACCTTCATACTATTTATATTAAACT----CATCCACCA--CGACCCTATCTTTATCCCACA---TATGAAACAAATTTCCCCTAATCACTTCCATAATCTTAATCTTAA
A-C_lupus_familiaris : CCTACAATAATAGTTCTAAACTTAACTTTATATATTCTAATAACACTATCTACCTTCATACTATTTATATTAAACT----CATCCACCA--CGACCCTATCTTTATCCCACA---TATGAAACAAATTTCCCCTAATCACTTCCATAATCTTAATCTTAA
R-N_procyonoides : CCAACAATAACAATCCTAAACCTAATCCTATATATCTTAATAACCCTATCAACATTTATACTATTTATACTAAACA----CATCCACCA--CAACCCTATCCCTCTCCCACG---TATGAAACAAATTCCCATTGATAACCTCCGTCATCCTGGCCCTAA
A-N_procyonoides : CCAACAATAACAATCCTAAACCTAATCCTATATATCTTAATAACCCTATCAACATTTATACTATTTATACTAAACA----CATCCACCA--CAACCCTATCCCTCTCCCACG---TATGAAACAAATTCCCATTGATAACCTCCGTCATCCTGGCCCTAA
R-V_lagopus : CCTACAATAATATTCCTAAACCTATCCCTATATATTTTTATGACCCTATCAACATTTATATTATTTATACTAAGCA----CATCCACCA--CAACCTTATCCCTCTCACACA---CATGAAATAAAGTCCCCTTAATCGCTTCCACCATTCTAACTCTAA
A-V_lagopus : CCTACAATAATATTCCTAAACCTATCCCTATATATTTTTATGACCCTATCAACATTTATATTATTTATACTAAGCA----CATCCACCA--CAACCTTATCCCTCTCACACA---CATGAAATAAAGTCCCCTTAATCGCTTCCACCATTCTAACTCTAA
R-M_putorius : CCAACCCTGATATTACTAAACCTCACAATTTACATTATAATAACACTAGGAACATTCATACTATTTACATTCAGCT----CATCTACAA--CTACACTATCACTATCCCTTA---CATGAAATAAACTCCCACTAATCACCTCACTAATCCTTATCATCA
A-M_putorius : CCAACCCTGATATTACTAAACCTCACAATTTACATTATAATAACACTAGGAACATTCATACTATTTACATTCAGCT----CATCTACAA--CTACACTATCACTATCCCTTA---CATGAAATAAACTCCCACTAATCACCTCACTAATCCTTATCATCA
R-B_taurus : CCCACCATAACATTGCTAAACTTAATTATCTATATCATTATAACTTCCACCATATTTACCATATTTATAGCCAATT----CCACCACCA--CTACCCTGTCATTATCACACA---CATGAAATAAAACACCCATTATAACCGTCCTAATTCTTGCCACTC
A-B_taurus : CCCACCATAACATTGCTAAACTTAATTATCTATATCATTATAACTTCCACCATATTTACCATATTTATAGCCAATT----CCACCACCA--CTACCCTGTCATTATCACACA---CATGAAATAAAACACCCATTATAACCGTCCTAATTCTTGCCACTC
R-O_aries : CCCACCATAACACTACTAAACCTAATTATTTATATCATTATAACCTCTACCATATTTACACTATTTATAGCCAACT----CAACCACAA--CCACCCTATCATTATCACACA---CATGAAATAAAGCACCCATCATAACAATTCTAGTCCTCATTACCC
A-O_aries : CCCACCATAACACTACTAAACCTAATTATTTATATCATTATAACCTCTACCATATTTACACTATTTATAGCCAACT----CAACCACAA--CCACCCTATCATTATCACACA---CATGAAATAAAGCACCCATCATAACAATTCTAGTCCTCATTACCC
R-S_scrofa : ACAACCATAACAATCTTAAACCTACTAATTTACATCACAACAACACTAGCAATATTCATACTATTAATCCACAGCT----CAGCAACCA--CAACTTTATCCCTATCCCATA---CATGAAACAAGATACCCATCATCACAAGCCTAATAATAGTAACCC
A-S_scrofa : ACAACCATAACAATCTTAAACCTACTAATTTACATCACAACAACACTAGCAATATTCATACTATTAATCCACAGCT----CAGCAACCA--CAACTTTATCCCTATCCCATA---CATGAAACAAGATACCCATCATCACAAGCCTAATAATAGTAACCC
R-O_cuniculus : CCAAATACTATAGTCCTAAACCTAATTATCTATATTCTCATGACAATCCCTATATTCATAATATTCATACAGCACT----CAAGTACGA--CCACTCTATCACTATCACAGA---TGTGAAACAAAAATCCACTAATAGTATCTACTATCCTAATCACCC
A-O_cuniculus : CCAAATACTATAGTCCTAAACCTAATTATCTATATTCTCATGACAATCCCTATATTCATAATATTCATACAGCACT----CAAGTACAA--CCACTCTATCACTATCACAAA---TGTGAAACAAAAATCCACTAATAGTATCTACTATCCTAATCACCC
R-M_musculus : CCATCCCTCACTCTACTCAACCTCATAATCTATATTATTCTTACAGCCCCTATATTCATAGCACTTATA-CTAAATAACTCTATAACCAT-CAACTCAATC----TCACTTC---TATGAAATAAAACTCCAGCAATACTAACTATAATCTCACTGATAT
A-M_musculus : CCATCCCTCACTCTACTCAACCTCATAATCTATATTATTCTTACAGCCCCTATATTCATAGCACTTATA-CTAAATAACTCTATAACCAT-CAACTCAATC----TCACTTC---TATGAAATAAAACTCCAGCAATACTAACTATAATCTCACTGATAT
R-R_norvegicus : CCTAACTTAACCCTCCTAAACTTAACAATTTACATCCTACTTACTGTTCCAATATTCATCACACTCATA-ACAAACTCAGCAACAACAAT-CAACACACTC----TCACTCG---CATGAAATAAAACTCCCATAATCCTAACCATAACATCCATCATCC
A-R_norvegicus : CCTAACTTAACCCTCCTAAACTTAACAATTTACATCCTACTTACTGTTCCAATATTCATCACACTCATA-ACAAACTCAGCAACAACAAT-CAACACACTC----TCACTCG---CATGAAATAAAACTCCCATAATCCTAACCATAGCATCCATCATCC
R-M_coypus : CCCTCAGCCACCCTATTTAACCTAATTATTTACATCATATTAACCATCTCATTATTTATTAC-CCTATATACAAA-----CAATAACCTTACCACACTATCCTTATCACACG---TATGAAGCACCGCACCCCCTACCATTATCATTATCTTAATAAATT
A-M_coypus : CCCTCAGCCACCCTATTTAACCTAATTATTTACATCATATTAACCATCTCATTATTTATTAC-CCTATATACAAA-----CAATAACCTTACCACACTATCCTTATCACACG---TATGAAGCACCGCACCCCCTACCATTATCATTATCTTAATAAATT
R-A_platyrhynchos : CCCAAGCTAGCACTACTCACCTTCTATCTCTACACAATCATGACATCAGCTGTATTCATGGC-CCTAAACAAGATT------AAAGCTC--TCAACCTGTCCATAATCCTAACCTCATGGACAAAGACCCCGGTACTAAACGCCACCCTAATGCTAGTGC
A-A_platyrhynchos : CCCAAGCTAGCACTACTCACCTTCTATCTCTACACAATCATGACATCAGCTGTATTCATGGC-CCTAAACAAGATT------AAAGCTC--TCAACCTGTCCATAATCCTAACCTCATGGACAAAGACCCCGGTACTAAACGCCACCCTAATGCTAGTGC
R-G_gallus : CCACAACTCACTATTCTCACCTTCATCCTCTACACAATTATGACCTCAACTGTATTCCTATC-CCTAGCCCAAATC------AAAGTCC--TAAAACTGTCAACACTACTCATCTCATGAACTAAAACCCCAATACTAAATGCAACTGTAATACTAACCC
A-G_gallus : CCACAACTCACTATTCTCACCTTCATCCTCTACACAATTATGACCTCAACTGTATTCCTATC-CCTAGCCCAAATC------AAAGTCC--TAAAACTGTCAACAATACTCATCTCATGAACTAAAACCCCAATACTAAATGCAACTGTAATACTAACCC
 CCAACMATAACAMTACTAAACCTAATWATTTATATYATAATAACACTAACAATATTCATACTATTTATACACAACTWMMKCAACAACCATACAACCCTATCMCTATCACACAYCTCATGAAAYAAAACHCCCATAATMACAACCATAATCCTAATCATCC

 * 6260 * 6280 * 6300 * 6320 * 6340 * 6360 * 6380 * 6400
R-E_caballus : TACTATCCATAGGAGGCCTCCCCCCACTATCAGGATTCATACCCAAATGAATAATCATTCAAGAGCTCACCAAAAATAGCAGCATCATCCTCCCCACACTAATAGCCATTATAGCACTACTCAACCTCTACTTCTACATACGACTAACCTATTCCACCTC
A-E_caballus : TACTATCCATAGGAGGCCTCCCCCCACTATCAGGATTCATACCCAAATGAATAATCATTCAAGAGCTCACCAAAAATAGCAGCATCATCCTCCCCACACTAATAGCCATTATAGCACTACTCAACCTCTACTTCTACATACGACTAACCTATTCCACCTC
R-C_bactrianus : TAATATCTATAGGAGGCCTCCCCCCACTATCTGGATTTATACCCAAATGAATAATCATCCAAGAGCTGACAAAAAATGATAACATTATTCTCCCCACTCTAATAGCTATAATAGCATTACTAAACCTATATTTTTATATGCGACTAGCATACTCCACGGC
A-C_bactrianus : TAATATCTATAGGAGGCCTCCCCCCACTATCTGGATTTATACCCAAATGAATAATCATCCAAGAGCTGACAAAAAATGATAACATTATTCTCCCCACTCTAATAGCTATAATAGCATTACTAAACCTATATTTTTATATGCGACTAGCATACTCCACAGC
R-C_lupus_familiaris : TACTATCCCTAGGAGGACTACCCCCATTATCTGGCTTCATCCCCAAATGAATAATTATTCAAGAATTAACGAAAAATAACATAATTATTATTCCAACACTAATGGCTATCACCGCTCTACTTAACTTATATTTCTACCTGCGACTCACATATAGCACCGC
A-C_lupus_familiaris : TACTATCCCTAGGAGGACTACCCCCATTATCTGGCTTCATCCCCAAATGAATAATTATTCAAGAATTAACGAAAAATAACATAATTATTATTCCAACACTAATGGCTATCACCGCTCTACTTAACTTATATTTCTACCTGCGACTCACATATAGCACCGC
R-N_procyonoides : TATTATCCCTAGGGGGTCTACCGCCACTATCCGGTTTCATCCCTAAATGAATAATCATCCTAGAATTAACAAAAAACAACATAATCATCACTCCAACACTCATAGCTATCACTGCCCTAATCAACTTATACTTCTACATACGACTCACATACAGCACTGC
A-N_procyonoides : TATTATCCCTAGGGGGTCTACCGCCACTATCCGGTTTCATCCCTAAATGAATAATCATCCTAGAATTAACAAAAAACAACATAATCATCACTCCAACACTCATAGCTATCACTGCCCTAATCAACTTATACTTCTACATACGACTCACATACAGCACTGC
R-V_lagopus : TACTATCCCTGGGAGGACTTCCACCACTATCCGGCTTCATCCCTAAATGAATAATCATCCAGGAACTAACAAAAAATGACATAATTATTATCCCAACACTTATAGCTATCACCGCACTACTCAACCTATACTTCTACATACGACTCACATACAGCACTGC
A-V_lagopus : TACTATCCCTGGGAGGACTTCCACCACTATCCGGCTTCATCCCTAAATGAATAATCATCCAGGAACTAACAAAAAATGACATAATTATTATCCCAACACTTATAGCTATCACCGCACTACTCAACCTATACTTCTACATACGACTCACATACAGCACTGC
R-M_putorius : TACTATCACTAGGAGGCTTACCACCACTTTCAGGCTTCATACCCAAATGAATAATCATCCACGAACTCACAAAAAACAACATAATTACTGCAGCAATATTCATAACAATCACAGCCCTACTAAACTTATACTTTTACATACGACTAACATACGCAACAGC
A-M_putorius : TACTATCACTAGGAGGCTTACCACCACTTTCAGGCTTCATACCCAAATGAATAATCATCCACGAACTCACAAAAAACAACATAATTACTGCAGCAATATTCATAACAATCACAGCCCTACTAAACTTATACTTTTACATACGACTAACATACGCAACAGC
R-B_taurus : TCCTATCCATAGGAGGACTCCCTCCCCTATCTGGGTTTATACCAAAATGAATAATCATCCAAGAGATAACAAAAAATAACAGCATCATTCTACCCACTTTCATAGCAATCACAGCTCTACTAAACTTATATTTTTATATACGACTCACGTATTCTACCAC
A-B_taurus : TCCTATCCATAGGAGGACTCCCTCCCCTATCTGGGTTTATACCAAAATGAATAATCATCCAAGAGATAACAAAAAATAACAGCATCATTCTACCCACTTTCATAGCAATCACAGCTCTACTAAACTTATATTTTTATATACGACTCACGTATTCTACCAC
R-O_aries : TCCTATCAATAGGAGGACTTCCCCCACTATCAGGATTTATACCAAAATGAATAATTATCCAAGAAATAACAAAAAATGACAGCATTATCTTACCCACCCTCATAGCAATTACAGCACTACTAAACCTATATTTTTATATACGACTTACATACTCCACTGC
A-O_aries : TCCTATCAATAGGAGGACTTCCCCCACTATCAGGATTTATACCAAAATGAATAATTATCCAAGAAATAACAAAAAATGACAGCATTATCTTACCCACCCTCATAGCAATTACAGCACTACTAAACCTATATTTTTATATACGACTTACATACTCCACTGC
R-S_scrofa : TACTCTCAATAGGAGGCCTGCCTCCACTATCAGGATTTATACCAAAATGAATAATCATTCAAGAAATAACAAAAAATGAAAGCATCATCATGCCAACACTCATAGCAATAACAGCACTGCTAAACCTCTATTTCTACATACGACTAGCCTACTCCTCCTC
A-S_scrofa : TACTCTCAATAGGAGGCCTGCCTCCACTATCAGGATTTATACCAAAATGAATAATCATTCAAGAAATAACAAAAAATGAAAGCATCATCATGCCAACACTCATAGCAATAACAGCACTGCTAAACCTCTATTTCTACATACGACTAGCCTACTCCTCCTC
R-O_cuniculus : TAATATCCCTAGGAGGCCTGCCCCCACTCACAGGTTTTATCCCTAAATGAATTATCATCCAGGAATTAACAAAAAATGGCAATATCATTTTACCCACCGCTATAGCCATGCTTGCCCTCCTAAATCTATATTTTTATATACGCCTTATCTATTCTTCCTC
A-O_cuniculus : TAATATCCCTAGGAGGCCTGCCCCCACTCACAGGTTTTATCCCTAAATGAATTATCATCCAGGAATTAACAAAAAATGGCAATATCATTTTACCCACCGCTATAGCCATGCTTGCCCTCCTAAATCTATATTTTTATATACGCCTTATCTATTCTTCCTC
R-M_musculus : TACTATCCCTAGGAGGCCTTCCACCACTAACAGGATTCTTACCAAAATGAATTATCATCACAGAACTTATAAAAAACAACTGTCTAATTATAGCAACACTCATAGCAATAATAGCTCTACTAAACCTATTCTTTTATACTCGCCTAATTTATTCCACTTC
A-M_musculus : TACTATCCCTAGGAGGCCTTCCACCACTAACAGGATTCTTACCAAAATGAATTATCATCACAGAACTTATAAAAAACAACTGTCTAATTATAGCAACACTCATAGCAATAATAGCTCTACTAAACCTATTCTTTTATACTCGCCTAATTTATTCCACTTC
R-R_norvegicus : TCCTATCACTAGGAGGACTCCCCCCTCTCACAGGATTTTTACCAAAATGAGCAATTATCTCCGAGCTTCTAAAAAACAACTGCTCAACCCTATCAACACTAATAGCTATCATAGCCCTATTAAGCCTATTCTTCTATACACGACTAATTTACTCTATATC
A-R_norvegicus : TCCTATCACTAGGAGGACTCCCCCCTCTCACAGGATTTTTACCAAAATGAACAATTATCTCCGAGCTTCTAAAAAACAACTGCTCAACCCTATCAACACTAATAGCTATCATAGCCCTATTAAGCCTATTCTTCTATACACGACTAATTTACTCTATATC
R-M_coypus : TATTATCCCTAGGAGGCCTTCCCCCTTTAACTGGATTTGCCCCTAAATGAATCATTATTCAAGAACTAATTAAAAATAATAATATCGTACTTCCCATACTCATAACAATAATAGCTTTACTAAGCCTATATTTCTATATACGATTAACATATTCAACAAC
A-M_coypus : TATTATCCCTAGGAGGCCTTCCCCCTTTAACTGGATTTGCCCCTAAATGAATCATTATTCAAGAACTAATTAAAAATAATAATATCGTACTTCCCATACTCATAACAATAATAGCTTTACTAAGCCTATATTTCTATATACGATTAACATATTCAACAAC
R-A_platyrhynchos : TGCTGTCCCTGGCAGGCCTCCCCCCACTGACAGGGTTTATACCAAAGTGACTCATCATCCAAGAGCTAACTAAGCAAGAGATAACACCCGCAGCCATAGCAATCGCCATGCTATCCCTACTTAGCCTATTCTTCTACCTACGCCTCGCATACCACTCAAC
A-A_platyrhynchos : TGCTGTCCCTGGCAGGCCTCCCCCCACTGACAGGGTTTATACCAAAGTGACTCATCATCCAAGAGCTAACTAAGCAAGAGATAACACCCGCAGCCATAGCAATCGCCATGCTATCCCTACTTAGCCTATTCTTCTACCTACGCCTCGCATACCACTCAAC
R-G_gallus : TCCTCTCCCTAGCTGGCCTCCCACCATTAACCGGCTTCATGCCAAAATGACTCATTATCCAAGAACTAACCAAACAAGAAATAACCCCAATAGCCACAATCATCACAATACTATCACTCCTAAGCCTATTCTTCTACCTCCGACTTGCATACCACTCAAC
A-G_gallus : TCCTCTCCCTAGCTGGCCTCCCACCATTAACCGGCTTCATGCCAAAATGACTCATTATCCAAGAACTAACCAAACAAGAAATAACCCCAATAGCCACAATCATCACAATACTATCACTCCTAAGCCTATTCTTCTACCTCCGACTTGCATACCACTCAAC
 TACTATCCCTAGGAGGCCTCCCCCCACTATCAGGATTTATACCAAAATGAATAATCATCCAAGAACTAACAAAAAATAACAKMATCATTATACCCACACTCATAGCAATCATAGCACTACTAAACCTATACTTCTACATACGACTAACATACTCCACAGC

 * 6420 * 6440 * 6460 * 6480 * 6500 * 6520 * 6540 * 6560
R-E_caballus : ACTGACCATATTCCCA---TCCACAAACAACATAAAAATAAAATGACAATTCGA-A---ACC--AAACGAATTACTCTCTTACCCCCGTTAATTGTTATATCCTCCCTACTCCTCCC-CCTAACCCCCATACTA-TCAATTTTGGACTA---------GG
A-E_caballus : ACTGACCATATTCCCA---TCCACAAACAACATAAAAATAAAATGACAATTCGA-A---ACC--AAACGAATTACTCTCTTACCCCCGTTAATTGTTATATCCTCCCTACTCCTCCC-CCTAACCCCCATACTA-TCAATTTTGGACTA---------GG
R-C_bactrianus : ACTCACTATATTTCCA---TCATCTAACAACATAAAAATAAAATGACAATTCGA-GGGCACG--AAACGAATGACATCCTTACCAATCATAATTGTTCTATCCACCATAATTCTACC-CCTAACCCCAATACTA-TCAGTACTATATTA---------GG
A-C_bactrianus : ACTCACTATATTTCCA---TCATCTAACAACATAAAAATAAAATGACAATTCGA-GGGCACG--AAACGAATGACATCCTTACCAATCATAATTGTTCTATCCACCATAATTCTACC-CCTAACCCCAATACTA-TCAGTACTATATTA---------GG
R-C_lupus_familiaris : ACTTACCATATTTCCA---TCCACAAACAACATAAAAATAAAATGACAGTTCGA-ATACACA--AAAAAGGCAACCCTATTACCCCCCTTAATTATTACCTCAACTATACTACTCCC-ACTAACACCTATATTA-TCAGTCTTGGACTA---------GG
A-C_lupus_familiaris : ACTTACCATATTTCCA---TCCACAAACAACATAAAAATAAAATGACAGTTCGA-ATACACA--AAAAAGGCAACCCTATTACCCCCCTTAATTATTACCTCAACTATACTACTCCC-ACTAACACCTATATTA-TCAGTCTTGGACTA---------GG
R-N_procyonoides : ACTTACCATATTTCCA---TCTGCAAACAACATAAAAATAAAATGACAATTCGA-ACATACA--AAAAAGATAACTCTACTACCCCCTCTCATTATCACCTCTACTATATTACTCCC-ACTATCACCCACACTA-TCAATCTTATACTA---------GA
A-N_procyonoides : ACTTACCATATTTCCA---TCTGCAAACAACATAAAAATAAAATGACAATTCGA-ACATACA--AAAAAGATAACTCTACTACCCCCTCTCATTATCACCTCTACTATATTACTCCC-ACTATCACCCACACTA-TCAATCTTATACTA---------GA
R-V_lagopus : ACTCACCATATTCCCA---TCCGCAAACAACATAAAAATAAAATGACAGTTCGA-GCACACA--AAAAAGACAACCCTACTGCCCCCTTTGATTATCATTTCAACTATATTACTCCCCATTA-CACCCATAATA-TCAATCTTGGACTA---------GA
A-V_lagopus : ACTCACCATATTCCCA---TCCGCAAACAACATAAAAATAAAATGACAGTTCGA-GCACACA--AAAAAGACAACCCTACTGCCCCCTTTGATTATCATTTCAACTATATTACTCCCCATTA-CACCCATAATA-TCAATCTTGGACTA---------GA
R-M_putorius : ACTAACCTTATTCCCC---TCAACAAATAACATAAAAATAAAATGGCAATTTGA-AAGCACA--AAATATACAACCCTATTACCCCCATTAATTGTGATATCAACTATACTTCTCCC-ACTCACCCCAATAATA-CCAACACTATTCTA---------GG
A-M_putorius : ACTAACCTTATTCCCC---TCAACAAATAACATAAAAATAAAATGGCAATTTGA-AAGCACA--AAATATACAACCCTATTACCCCCATTAATTGTGATATCAACTATACTTCTCCC-ACTCACCCCAATAATA-CCAACACTATTCTA---------GG
R-B_taurus : ACTAACAATATTTCCC---TCCACAAACAACATAAAAATAAAATGACAATTTCC-CCTTATG--AAAAAAATAACTTTTCTACCAACAATAGTCGTATTATCTACCATAATACTACC-ACTCACGCCAATACTA-TCAGTGTTAGAATA---------GG
A-B_taurus : ACTAACAATATTTCCC---TCCACAAACAACATAAAAATAAAATGACAATTTCC-CCTTATG--AAAAAAATAACTTTTCTACCAACAATAGTCGTATTATCTACCATAATACTACC-ACTCACGCCAATACTA-TCAGTGTTAGAATA---------GG
R-O_aries : ACTCACGATATTTCCC---TCCACAAACAACATAAAAATGAAATGACAATTCCC-AACCACA--AAACGAATAACCCTCCTACCAACAATAACTGTACTATCCACCATACTACTACC-ACTAACACCAATCCTC-TCAATTCTAGAATA---------GG
A-O_aries : ACTCACGATATTTCCC---TCCACAAACAATATAAAAATGAAATGACAATTCCC-AACCACA--AAACGAATAACCCTCCTACCAACAATAACTGTACTATCCACCATATTACTACC-ACTAACACCAATTCTC-TCAATTCTAGAATA---------GG
R-S_scrofa : ACTGACTATGTTCCCA---TCCACCAATAACATAAAAATAAAATGACAATTCGA-ACACACA--AAACAAATAAAACTACTTCCCACAATAATTGTATTATCGACACTAATCCTACC-TATAACACCAGCCCTC-TCGTCCCTAAACTA---------GG
A-S_scrofa : ACTGACTATGTTCCCA---TCCACCAATAACATAAAAATAAAATGACAATTCGA-ACACACA--AAACAAATAAAACTACTTCCCACAATAATTGTATTATCGACACTAATCCTACC-TATAACACCAGCCCTC-TCGTCCCTAAACTA---------GG
R-O_cuniculus : ACTAACTATATTCCCT---ACCACCAACAATCTAAAAATAAAATGACAATTTGA-ATCAACA--AAACGCATACCCCTCATCACTCCACTAATTATTTTATCAACTATACTCCTCCC-CCTCACACCAGCACTC-TCAGTATTAAATTAAGCTTTAAAGG
A-O_cuniculus : ACTAACTATATTCCCT---ACCACCAACAATCTAAAAATAAAATGACAATTTGA-ATCAACA--AAACGCATACCCCTCATCACTCCACTAATTATTTTATCAACCATACTCCTCCC-CCTCACACCAGCACTC-TCAGTATTAAATTA-GCTTTAAAGG
R-M_musculus : ACTAACAATATTTCCA---ACCAACAATAACTCAAAAATAATAACTCACCAA-ACAAAAACT--AAACCCAACCTAATATTTTCCACCCTAGCTATCATAAGCACAATAACCCTACC-CCTAGCCCCCCAACTAATTACCTAGAAGTTT--------AGG
A-M_musculus : ACTAACAATATTTCCA---ACCAACAATAACTCAAAAATAATAACTCACCAA-ACAAAAACT--AAACCCAACCTAATATTTTCCACCCTAGCTATCATAAGCACAATAACCCTACC-CCTAGCCCCCCAACTAATTACCTAGAAGTTT--------AGG
R-R_norvegicus : CCTCACCATATTCCCA---ACCAACAACAACTCCAAAATAATCTCCCACCACCACCAAAACCCAAAACATAATTTTATCCTCCCAACCCTCACAGTATTAAGTACCCTTACCCTACC-GCTTTCCTCCCAACTAATCACATAGAAGTTT--------AGG
A-R_norvegicus : CCTCACCATATTCCCA---ACCAACAACAACTCCAAAATAATCTCCCACCACCACCAAAACCCAAAACATAATTTTATCCTCCCAACCCTCACAGTATTAAGTACCCTTACCCTACC-GCTTTCCTCCCAACTAATCACATAGAAGTTT--------AGG
R-M_coypus : CCTAACACTATTTCCA---ACTACAAATAACACAAAAACCAAATGATACTTCAACAATAACA--AAACATTAA-TAATTCCACCCTCTTTAACCACATTATCTACTATAGCTCTTCC-ACTAACCCCTCTACTC-TCAATTTTAAGTTA--------AGG
A-M_coypus : CCTAACACTATTTCCA---ACTACAAATAACACAAAAACCAAATGATACTTCAACAATAACA--AAACATTAA-TAATTCCACCCTCTTTAACCACATTATCTACTATAGCTCTTCC-ACTAACCCCTCTACTC-TCAATTTTAAGTTA--------AGG
R-A_platyrhynchos : AATCACCCTCCCACCAAACTCGTCCAACCACATAAAACAG---TGGTACACTAGCAAACCCC-----CAAGCACGCCCACCG-CAATCCTAGCCTCACTATCAATCCTCCTACTCCC-CCTCTCCCCCATAGTCCACG-CTATTGTCTA---------GA
A-A_platyrhynchos : AATCACCCTCCCACCAAACTCGTCCAACCACATAAAACAG---TGGTACACTAGCAAACCCC-----CAAGCACGCCCACCG-CAATCCTAGCCTCACTATCAATCCTCCTACTCCC-CCTCTCCCCCATAGTCCACG-CTATTGTCTA---------GA
R-G_gallus : AATCACACTCCCCCCCAACTCATCAAACCACATAAAACTC---TGACGAACTAACAAAACCC-----TAAACACCCCCACCG-CCATTCTAACTGCGCTATCAACCACCCTATTGCC-CCTTTCCCCCCTAATTATTA-CCATACTATA---------GA
A-G_gallus : AATCACACTCCCCCCCAACTCATCAAACCACATAAAACTC---TGACGAACTAACAAAACCC-----TAAACACCCCCACCG-CCATTCTAACTGCGCTATCAACCACCCTATTGCC-CCTTTCCCCCCTAATTATTA-CCATACTATA---------GA
 ACTCACCATATTYCCAAACTCCACAAACAACATAAAAATAAAATGACAATTCGACAAACACACAAAACAAATAACCCTACTACCCACMCTAATTGTAATATCAACCATACTACTCCCCACTAACCCCCATACTAATCAATTTTAGACTAAGCTTTAAAGG

 * 6580 * 6600 * 6620 * 6640 * 6660 * 6680 * 6700 * 6720
R-E_caballus : AATTTAGG-TTAACAT---CCCAGACCAAGAGCCTTCAAAGCTCTAAGCAAGTG-AATC--CACTTAATTCCTG---CATA------CTAAGGACTGCGAGACTCTAT-CTCACATCAATTGAACGCAAATCAAACTCTTTTATTAAGCTAAGCCCTT-A
A-E_caballus : AATTTAGG-TTAACAT---CCCAGACCAAGAGCCTTCAAAGCTCTAAGCAAGTG-AATC--CACTTAATTCCTG---CATA------CTAAGGACTGCGAGACTCTAT-CTCACATCAATTGAACGCAAATCAAACACTTTTATTAAGCTAAGCCCTT-A
R-C_bactrianus : AATTTAGG-CTAAA------TCAGACCAAGAGCCTTCAAAGCTCTAAGTAAGTACACAA--TACTTAATTCCTGTT--TAA-------TAAGGACTGCAAGACTCTAT-CCTACATCAATTGAATGCAAACCAACTGCTTTAATTAAGCTAAGCCCTT-A
A-C_bactrianus : AATTTAGG-CTAAA------TCAGACCAAGAGCCTTCAAAGCTCTAAGTAAGTACACAA--TACTTAATTCCTGTT--TAA-------TAAGGACTGCAAGACTCTAT-CCTACATCAATTGAATGCAAACCAACTGCTTTAATTAAGCTAAGCCCTT-A
R-C_lupus_familiaris : AGTTTAGG-TTAGA------CCAGACCAAGAGCCTTCAAAGCTCTAAGCAAGTGCTACA--CACTTAACCCCTGAT-CAAATCACCTCTAAGGGCTGCAAGAATCTAT-CTTACATCAATTGAATGCAAATCAAACACTTTAATTAAGCTAAGCCCTC-C
A-C_lupus_familiaris : AGTTTAGG-TTAGA------CCAGACCAAGAGCCTTCAAAGCTCTAAGCAAGTGCTACA--CACTTAACCCCTGAT-CAAATCACCTCTAAGGGCTGCAAGAATCTAT-CTTACATCAATTGAATGCAAATCAAACACTTTAATTAAGCTAAGCCCTC-C
R-N_procyonoides : GGTTTAGG-TTAAA------CTAGACCAAGAGCCTTCAAAGCTCTAAGCAAGTGTTGTT--CACTTAACCCCTGAA-CCAATC--CCCTAAGGACTGCAGGAGCTCAC-CCCACATCAATTGAATGCAAATCAATCACTTTAATTAAGCTAAGTCCTT-G
A-N_procyonoides : GGTTTAGG-TTAAA------CTAGACCAAGAGCCTTCAAAGCTCTAAGCAAGTGTTGTT--CACTTAACCCCTGAA-CCAATC--CCCTAAGGACTGCAGGAGCTCAC-CCCACATCAATTGAATGCAAATCAATCACTTTAATTAAGCTAAGTCCTT-G
R-V_lagopus : GGTTTAGG-TTAGA------CAAGACCAAGAGCCTTCAAAGCTCTAAGCAAGTGCTATA--CACTTAACCCCTGAC-CAAAGCA-CTCTAAGGACTGCAGGAATCTAC-CCTACATCAATTGAATGCAAATCAAACACTTTAATTAAGCTAAGTCCTT-A
A-V_lagopus : GGTTTAGG-TTAGA------CAAGACCAAGAGCCTTCAAAGCTCTAAGCAAGTGCTATA--CACTTAACCCCTGAC-CAAAGCA-CTCTAAGGACTGCAGGAATCTAC-CCTACATCAATTGAATGCAAATCAAACACTTTAATTAAGCTAAGTCCTT-A
R-M_putorius : AGTTTAGG-TTAAA-------AAGACCAAGGACCTTCAAAGCCCTAAGTAAGTGACACT--CACTTAACTCCTGATTCCCATCA----TAAGGACTGCAAGGATATAT-CTCACATCTATTGAACGCAAATCAATCACTTTAATTAAGCTAAGCCCTT-C
A-M_putorius : AGTTTAGG-TTAAA-------AAGACCAAGGACCTTCAAAGCCCTAAGTAAGTGACACT--CACTTAACTCCTGATTCCCATCA----TAAGGACTGCAAGGATATAT-CTCACATCTATTGAACGCAAATCAATCACTTTAATTAAGCTAAGCCCTT-C
R-B_taurus : AATTTAGG-TTAA-------ACAGACCAAGAGCCTTCAAAGCCCTAAGCAAGTACAATT--TACTTAATTCCTGA-------------TAAGGATTGCAAGACTACAC-CTTACATCAATTGAATGCAAATCAACCACTTTAATTAAGCTAAATCCTC-A
A-B_taurus : AATTTAGG-TTAA-------ACAGACCAAGAGCCTTCAAAGCCCTAAGCAAGTACAATT--TACTTAATTCCTGA-------------TAAGGATTGCAAGACTACAC-CTTACATCAATTGAATGCAAATCAACCACTTTAATTAAGCTAAATCCTC-A
R-O_aries : AATTTAGG-TTAA-------ACAGACCAAGAGCCTTCAAAGCCCTAAGCAAGTATAATT--TACTTAATTCCTGA-------------TAAGGACTGCAAGACTACAT-CTTACATCAATTGAATGCAAATCAACCACTTTAATTAAGCTAAATCCTC-A
A-O_aries : AATTTAGG-TTAA-------ACAGACCAAGAGCCTTCAAAGCCCTAAGCAAGTATAATT--TACTTAATTCCTGA-------------TAAGGACTGCAAGACTACAT-CTTACATCAATTGAATGCAAATCAACCACTTTAATTAAGCTAAATCCTC-A
R-S_scrofa : AATTTAGG-TTAAC------ACAGACCAAGAGCCTTCAAAGCTCTAAGTAAGTACAAAG--TACTTAACTCCTGAA---AACC-----TAAGGACTGCAGGATT-CAT-CCTACATCAATTGAATGCAAATCAAACACTTTAATTAAGCTAAATCCTC-A
A-S_scrofa : AATTTAGG-TTAAC------ACAGACCAAGAGCCTTCAAAGCTCTAAGTAAGTACAAAG--TACTTAACTCCTGAA---AACC-----TAAGGACTGCAGGATT-CAT-CCTACATCAATTGAATGCAAATCAAACACTTTAATTAAGCTAAATCCTC-A
R-O_cuniculus : AGTTTAGG-TTACA-------CAGACCAAGAGCCTTCAAAGCTCTAAGCAAGTAGATCA--TACTTAACCCCTGCT------------TAAGGACTGCAAA--TTAAC-TTTACATCTCCTGAGTGCAAACCAGGTGCTTTAATTAAGCTAAATCCTC--
A-O_cuniculus : AGTTTAGG-TTACA-------CAGACCAAGAGCCTTCAAAGCTCTAAGCAAGTAGATCA--TACTTAACCCCTGCT------------TAAGGACTGCAAA--TTAAC-TTTACATCTCCTGAATGCAAACCAGGTGCTTTAATTAAGCTAAATCCTC--
R-M_musculus : ATAT--------AC-------TAGTCCGCGAGCCTTCAAAGCCCTAAGAAAACACACAA---GTTTAACTTCTGAT-------------AAGGACTGTAAGACTTCAT-CCTACATCTATTGAATGCAAATCAATTGCTTTAATTAAGCTAAGACCTCAA
A-M_musculus : ATAT--------AC-------TAGTCCGCGAGCCTTCAAAGCCCTAAGAAAACACACAA---GTTTAACTTCTGAT-------------AAGGACTGTAAGACTTCAT-CCTACATCTATTGAATGCAAATCAATTGCTTTAATTAAGCTAAGACCTCAA
R-R_norvegicus : ATAT--------AC--------AGTCCAAGAGCCTTCAAAGCCCTTAGAAAACAAACAA---GTTTAACTTCTGAT-------------AAGGACTGTAAGACTATAT-CTTACATCTGTTAAATGCAAATCAACTGCTTTAATTAAGCTAAATCCTCAA
A-R_norvegicus : ATAT--------AC--------AGTCCAAGAGCCTTCAAAGCCCTTAGAAAACAAACAA---GTTTAACTTCTGAT-------------AAGGACTGTAAGACTATAT-CTTACATCTGTTAAATGCAAATCAACTGCTTTAATTAAGCTAAATCCTCAA
R-M_coypus : AAATTAGG-TTAAC-------TAGACCAAGAGCCTTCAAAGCCCTAAGTAAATAATCCAC-TATTTATTTCCTGAT------------TAAAGGTTGCAAGCCTATAAACTTACATCACCTGAATGCAAAACAGATACTTTAATTAAGCTAAA-CCTTTA
A-M_coypus : AAATTAGG-TTAAC-------TAGACCAAGAGCCTTCAAAGCCCTAAGTAAATAATCCAC-TATTTATTTCCTGAT------------TAAAGGTTGCAAGCCTATAAACTTACATCACCTGAATGCAAAACAGATACTTTAATTAAGCTAAA-CCTTTA
R-A_platyrhynchos : AACTTAGGAT-AACACCCACCTAAACCGAAGGCCTTCAAAGCCTTAAATAAGAGTTAAACCCTCTTAGTTTCTGC-----------GCTAAGACCAACAGGACACTAA-CCTGTATCTCCTGGATGCAAACCAGACGCTTTAATTAAGCTAAAGCCTTTA
A-A_platyrhynchos : AACTTAGGAT-AACACCCACCTAAACCGAAGGCCTTCAAAGCCTTAAATAAGAGTTAAACCCTCTTAGTTTCTGC-----------GCTAAGACCAACAGGACACTAA-CCTGTATCTCCTGGATGCAAACCAGACGCTTTAATTAAGCTAAAGCCTTTA
R-G_gallus : AACTTAGGATTAACTGTCACC-AAACCAAAGGCCTTCAAAGCCTTAAATAAGAGTTAAACTCTCTTAGTTTCTGCC--------CAACTAAGACCAACAGGACATTAA-CCTGTATCTCCTGAATGCAAATCAGACGCTTTAATTAAGCTAAGGCCTCTA
A-G_gallus : AACTTAGGATTAACTGTCACC-AAACCAAAGGCCTTCAAAGCCTTAAATAAGAGTTAAACTCTCTTAGTTTCTGCC--------CAACTAAGACCAACAGGACATTAA-CCTGTATCTCCTGAATGCAAATCAGACGCTTTAATTAAGCTAAGGCCTCTA
 AATTTAGGATTAACABYCACCCAGACCAAGAGCCTTCAAAGCCCTAAGCAAGTACAAAACYCACTTAACTCCTGATTCAAATCACCTCTAAGGACTGCAAGACTMTATACTTACATCAATTGAATGCAAATCAAACACTTTAATTAAGCTAAGTCCTCTA

 * 6740 * 6760 * 6780 * 6800 * 6820 * 6840 * 6860 * 6880
R-E_caballus : C-TAGATTGGTGGGCTACCAT-CCCACGAAATTTT-AGTTAACAGCTAAATACCCTAATCAACTGGCTTCAATCTA-CTTCTCCCGCCGCCTAGA-AAAAA---AGGCGGGAGAAGCCCCGGCAGAAATT-GAAGCTGCTCCTTTGAATTTGCAATTCAA
A-E_caballus : C-TAGATTGGTGGGCTACCAT-CCCACGAAATTTT-AGTTAACAGCTAAATACCCTAATCAACTGGCTTCAATCTA-CTTCTCCCGCCGCCTAGA-AAAAA---AGGCGGGAGAAGCCCCGGCAGAA-TT-GAAGCTGCTCCTTTGAATTTGCAATTCAA
R-C_bactrianus : C-TAGATTGATGGGCCTTTAT-CCCACGAAATTTT-AGTTAACAGCTAAATACCCTAGTCAACTGGCTTCAATCTA-CTTCTCCCGCCGCGAGAAAAAAAA---AGGCGGGAGAAGCCCCGGCAGAGTTT-GAAGCTGCTTCTTTGAATTTGCAATTCAA
A-C_bactrianus : C-TAGATTGATGGGCCTTTAT-CCCACGAAATTTT-AGTTAACAGCTAAATACCCTAGTCAACTGGCTTCAATCTA-CTTCTCCCGCCGCGAGAAAAAAAA---AGGCGGGAGAAGCCCCGGCAGAGTTT-GAAGCTGCTTCTTTGAATTTGCAATTCAA
R-C_lupus_familiaris : C-TAGATTGGTGAGCTTCTAC-CTCACGAAATTTT-AGTTAACAGCTAAATACCCTAGT-AACTGGCTTCAATCTACCTTCTCCCGCCGCGTAGAAAAAAA---AGGCGGGAGAAGCCCCGGCGGCGTCT--AGGCTGCTTCTTTGAATTTGCAATTCAA
A-C_lupus_familiaris : C-TAGATTGGTGAGCTTCTAC-CTCACGAAATTTT-AGTTAACAGCTAAATACCCTAGT-AACTGGCTTCAATCTACCTTCTCCCGCCGCGTAGAAAAAAA---AGGCGGGAGAAGCCCCGGCGGCGTCT--AGGCTGCTTCTTTGAATTTGCAATTCAA
R-N_procyonoides : C-TAGATTGGTGGGCTTCCAT-CCCACGAAATTTT-AGTTAACAGCTAAATACCCTAAG-AACTGGCTTCAATCTACCTTCTCCCGCCGCGTAGAAAAAAA---AGGCGGGAGAAGCCCCGGCGGCGTCT--AGGCTGCTTCTTTGAATTTGCAATTCAA
A-N_procyonoides : C-TAGATTGGTGGGCTTCCAT-CCCACGAAATTTT-AGTTAACAGCTAAATACCCTAAG-AACTGGCTTCAATCTACCTTCTCCCGCCGCGTAGAAAAAAA---AGGCGGGAGAAGCCCCGGCGGCGTCT--AGGCTGCTTCTTTGAATTTGCAATTCAA
R-V_lagopus : C-TAGATTGGTGGGCTTTCAT-CCCACGAAATTTT-AGTTAACAGCTAAATACCCTAT--AACTGGCTTCAATCTAGCTTCTCCCGCCGTGTAGGGAAAAA---AGGCGGGAGAAGCCCCGGCGGCGTCT--AAGCTGCTTCTTTGAATTTGCAATTCAA
A-V_lagopus : C-TAGATTGGTGGGCTTTCAT-CCCACGAAATTTT-AGTTAACAGCTAAATACCCTAT--AACTGGCTTCAATCTAGCTTCTCCCGCCGTGTAGGGAAAAA---AGGCGGGAGAAGCCCCGGCGGCGTCT--AAGCTGCTTCTTTGAATTTGCAATTCAA
R-M_putorius : C-TAGATTGGTGGGCTACCAT-CCCACGAAACTTT-AGTTAACAGCTAAACACCCTAATCAACTGGCTTCAATCTA-CTTCTCCCGCCGCGAAGG--AAAA---AGGCGGGAGAAGCCCCGGCAGGGTTG--AAGCTGCTTCTTTGAATTTGCAATTCAA
A-M_putorius : C-TAGATTGGTGGGCTACCAT-CCCACGAAACTTT-AGTTAACAGCTAAACACCCTAATCAACTGGCTTCAATCTA-CTTCTCCCGCCGCGAAGG--AAAA---AGGCGGGAGAAGCCCCGGCAGGGTTG--AAGCTGCTTCTTTGAATTTGCAATTCAA
R-B_taurus : C-TAGACTGGTGGGCTC-CACCCCCACGAAACTTT-AGTTAACAGCTAAACACCCTAGCTAACTGGCTTCAATCTA-CTTCTCCCGCCGCA-AGAAAAAAA---AGGCGGGAGAAGCCCCGGCAGAATT--GAAGCTGCTTCTCTGAATTTGCAATTCAA
A-B_taurus : C-TAGACTGGTGGGCTC-CACCCCCACGAAACTTT-AGTTAACAGCTAAACACCCTAGCTAACTGGCTTCAATCTA-CTTCTCCCGCCGCA-AGAAAAAAA---AGGCGGGAGAAGCCCCGGCAGAATT--GAAGCTGCTTCTCTGAATTTGCAATTCAA
R-O_aries : C-TAGATTGGTGGGCTC-CACCCCCACGAAACTTT-AGTTAACAGCTAAACACCCTAAACAACTGGCTTCAATCTA-CTTCTCCCGCCGCG-AGAAAAAAA---AGGCGGGAGAAGCCCCGGCAGAGTTT-GAAGCTGCTTCTTTGAATTTGCAATTCAA
A-O_aries : C-TAGATTGGTGGGCTC-CACCCCCACGAAACTTT-AGTTAACAGCTAAACACCCTAAACAACTGGCTTCAATCTA-CTTCTCCCGCCGCG-AGAAAAAAA---AGGCGGGAGAAGCCCCGGCAGAGTTT-GAAGCTGCTTCTTTGAATTTGCAATTCAA
R-S_scrofa : C-TAGATTGGTGGGATTACATACCCACGAAACTTTTAGTTAACAGCTAAACACCCTAATCAACTGGCTTCAATCTA-CTTCTCCCGCCGCA-GGAAAAAAA---AGGCGGGAGAAGTCCCGGCAGAATT--GAAGCTGCTTCTTTGAATTTGCAATTCAA
A-S_scrofa : C-TAGATTGGTGGGATTACATACCCACGAAACTTTTAGTTAACAGCTAAACACCCTAATCAACTGGCTTCAATCTA-CTTCTCCCGCCGCA-GGAAAAAAA---AGGCGGGAGAAGTCCCGGCAGAATT--GAAGCTGCTTCTTTGAATTTGCAATTCAA
R-O_cuniculus : C-TAGATTGGTGGGCTCCAAC-CCCACGAAGCTTT-AGTTAACAGCTAAATACCCTAGTCAACTGGCTTCAACCTA-CTTCTCCCGCCGTAAGAAAAAAAA----GGCGGGAGAAGCCCCGGCAGAGTT--GAAGCTGCTTCTTTGAATTTGCAATTCAA
A-O_cuniculus : C-TAGATTGGTGGGCTCCAAC-CCCACGAAGCTTT-AGTTAACAGCTAAATACCCTAGTCAACTGGCTTCAACCTA-CTTCTCCCGCCGTAAGAAAAAAAA---AGGCGGGAGAAGCCCCGGCAGAGTT--GAAGCTGCTTCTTTGAATTTGCAATTCAA
R-M_musculus : C-TAGATTGGCAGGAATTAAA-CCTACGAAAATTT-AGTTAACAGCTAAATACCCTA--TTACTGGCTTCAATCTA-CTTCTACCGCCG--AAAAAAAAAA--ATGGCGGTAGAAGTCTTAGTAGAGAT--TTCTCTACACCTTCGAATTTGCAATTCGA
A-M_musculus : C-TAGATTGGCAGGAATTAAA-CCTACGAAAATTT-AGTTAACAGCTAAATACCCTA--TTACTGGCTTCAATCTA-CTTCTACCGCCG--AAAAAAAAAA--ATGGCGGTAGAAGTCTTAGTAGAGAT--TTCTCTACACCTTCGAATTTGCAATTCGA
R-R_norvegicus : C-TAGATTGGAAGGATTCAAA-CCTACGAAAATTT-AGTTAACAGCTAAATACCCTAC-TTACTGGCTTCAATCTA-CTTCTCCCGCCT--ATCAGAAAAA--GAGGCGGGAGAAGCCTTAGTAGAGGAGATTCTCTACACCTTCGAATTTGCAATTCGA
A-R_norvegicus : C-TAGATTGGAAGGATTCAAA-CCTACGAAAATTT-AGTTAACAGCTAAATACCCTAC-TTACTGGCTTCAATCTA-CTTCTCCCGCCT--ATCAGAAAA---GAGGCGGGAGAAGCCTTAGTAGAGGGGGTTCTCTACACCTTCGAATTTGCAATTCGA
R-M_coypus : C-TAGGTCGGTGGGACATTAA-CCCACGAAAAATT-AGTTAACAGCTAACTACCCTAAACAACTGGCTTCAACCTA-CTTCTCCCGCCCTAAAAAGAAAAAGGAGGGCGGGAGAAGCCCCGGCAGGTTTG--AAGCTGCTCCTTCGAATTTGCAATTCAA
A-M_coypus : C-TAGGTCGGTGGGACATTAA-CCCACGAAAAATT-AGTTAACAGCTAACTACCCTAAACAACTGGCTTCAACCTA-CTTCTCCCGCCCTAAAAAGAAAAAGGAGGGCGGGAGAAGCCCCGGCAGGTTTG--AAGCTGCTCCTTCGAATTTGCAATTCAA
R-A_platyrhynchos : C-TAGACAGACGGGCTTCGAT-CCCGCAAAATTTT-AGTTAACAGCTAAACGCCCAAACCTACTGGCCTCTGCCTA-------------------------------------AGGCCCCGGTACACTCT--CGTGCACATCGATGAGCTTGCAACTCAA
A-A_platyrhynchos : C-TAGACAGACGGGCTTCGAT-CCCGCAAAATTTT-AGTTAACAGCTAAACGCCCAAACCTACTGGCCTCTGCCTA-------------------------------------AGGCCCCGGTACACTCT--CGTGCACATCGATGAGCTTGCAACTCAA
R-G_gallus : CCTAGACAGATGGGCTTCGAT-CCCATACAATTTT-AGTTAACAGCTAAATGCCAACACCAATTGGCTTCTGCCTA------------------------------------CAGACCCCGGCACACTTT--AGTGTACATCAACGAGTTTGCAACTCAT
A-G_gallus : CCTAGACAGATGGGCTTCGAT-CCCATACAATTTT-AGTTAACAGCTAAATGCCAACACCAATTGGCTTCTGCCTA------------------------------------CAGACCCCGGCACACTTT--AGTGTACATCAACGAGTTTGCAACTCAT
 CCTAGATTGGTGGGCTTCCATCCCCACGAAATTTTTAGTTAACAGCTAAATACCCTAATCAACTGGCTTCAATCTACCTTCTCCCGCCGCGAAGAAAAAAAGGAAGGCGGGAGAAGCCCCGGCAGAGTTTRGAAGCTGCTTCTTTGAATTTGCAATTCAA

 * 6900 * 6920 * 6940 * 6960 * 6980 * 7000 * 7020 * 7040
R-E_caballus : TGTGAA--AATTCACCACGGGACTT-----GATAAGAAGAGGATTCCAACCCCTGTCTTTAGATTTACAGTCTAATGCTTAC-----TCAGCCATCTTACC------TATG---TTCATCAACCGCTGACTATTTTCAACTAACCACAAAGACATCGGCA
A-E_caballus : TGTGAA--A-TTCACCACGGGACTT-----GATAAGAAGAGGATTCCAACCCCTGTCTTTAGATTTACAGTCTAATGCTTAC-----TCAGCCATCTTACC------TATG---TTCATCAACCGCTGACTATTTTCAACTAACCACAAAGACATCGGCA
R-C_bactrianus : TATGTT---TTACACCACAAGGCTT-----GGTAAGAAGAGGGCTCTCACCTCTGTCTTTAGATTTACAGTCTAATGCCTAC-----TCGGCCATCTTACC------TATG---TTCATTACTCGCTGATTATTCTCAACCAACCACAAAGATATTGGAA
A-C_bactrianus : TATGTT---TTACACCACAAGGCTT-----GGTAAGAAGAGGGCTCTCACCTCTGTCTTTAGATTTACAGTCTAATGCCTAC-----TCGGCCATCTTACC------TATG---TTCATTACTCGCTGATTATTCTCAACCAACCACAAAGATATTGGAA
R-C_lupus_familiaris : TATGAA--AATTCACCACGGAGCTT-----GGCAAAAAGAGGACTTAAACCCCTATCTTTAGATTTACAGTCTAATGCTTTTA----TCAGCCATTTTACC------TATG---TTCATTAACCGATGACTGTTCTCCACTAATCACAAGGATATTGGTA
A-C_lupus_familiaris : TATGAA--AATTCACCACGGAGCTT-----GGCAAAAAGAGGACTTAAACCCCTATCTTTAGATTTACAGTCTAATGCTTTTA----TCAGCCATTTTACC------TATG---TTCATTAACCGATGATTGTTCTCCACTAATCACAAGGATATTGGTA
R-N_procyonoides : TATGAGA-AATTCACCACAGGGCTT-----GGTAAAAAGAGGACTAGAACCTCTATCTTTAGATTTACAGTCTAATGCTTATA----TCAGCCATTCTACC------TATG---TTCATTAACCGATGACTATTCTCTACTAACCACAAAGACATTGGCA
A-N_procyonoides : TATGAGA-AATTCACCACAGGGCTT-----GGTAAAAAGAGGACTAGAACCTCTATCTTTAGATTTACAGTCTAATGCTTATA----TCAGCCATTCTACC------TATG---TTCATTAACCGATGACTATTCTCTACTAACCACAAAGACATTGGCA
R-V_lagopus : TATGAAT-AATTCACCACAAGGCTT-----GGCAAAAAGAGGACTCACACCTCTATCTTTAGATTTACAGTCTAACGCTTTTA----TCAGCCATTTTACC------TATG---TTCATTAATCGATGATTATTCTCTACTAACCACAAAGACATTGGCA
A-V_lagopus : TATGAAT-AATTCACCACAAGGCTT-----GGCAAAAAGAGGACTCACACCTCTATCTTTAGATTTACAGTCTAACGCTTTTA----TCAGCCATTTTACC------TATG---TTCATTAATCGATGATTATTCTCTACTAACCACAAAGACATTGGCA
R-M_putorius : CGTGAT--ATTTCACCACAGAGCTTTTTTTGGCAAAAAGGGGACTTAAACCCCTATTCTTAGATTTACAGTCTAATGCCTTTA----TCAGCCATTTTACC------TATG---TTCATTAATCGATGATTATTCTCCACTAATCACAAAGACATCGGCA
A-M_putorius : CGTGAT--ATTTCACCACAGAGCTTTTTTTGGCAAAAAGGGGACTTAAACCCCTATTCTTAGATTTACAGTCTAATGCCTTTA----TCAGCCATTTTACC------TATG---TTCATTAATCGATGATTATTCTCCACTAATCACAAAGACATCGGCA
R-B_taurus : CGTGTA--AATTCACCACAGGGCTT-----GGTAAAAAGAGGAGTCAAACCTCTATCTTTAGATTTACAGTCTAATGCTTTGC----TCAGCCATTTTACC------CATG---TTCATTAACCGCTGACTATTCTCAACCAGCCATAAAGATATTGGTA
A-B_taurus : CGTGTA--AATTCACCACAGGGCTT-----GGTAAAAAGAGGAGTCAAACCTCTATCTTTAGATTTACAGTCTAATGCTTTGC----TCAGCCATTTTACC------CATG---TTCATTAACCGCTGACTATTCTCAACCAACCATAAAGATATTGGTA
R-O_aries : TATGTT--AATTCACTACAGGACCT-----GGTAAAAAGAGGAATTAAACCTCTGTTCTTAGATTTACAGTCTATTGCTTTAC----TCAGCCATTTTACC------CATG---TTCATCAACCGCTGATTATTTTCAACCAACCACAAAGATATCGGCA
A-O_aries : TATGTT--AATTCACTACAGGACCT-----GGTAAAAAGAGGAATTAAACCTCTGTTCTTAGATTTACAGTCTATTGCTTTAC----TCAGCCATTTTACC------CATG---TTCATCAACCGCTGATTATTTTCAACCAACCACAAAGATATCGGCA
R-S_scrofa : CATG-A--CATTCACCACGGAAC-T-----GGCAAAAAGAGGGCTTAA-CCTCTGTCTTTAGATTTACAGTCTAATGCTT-AC----TCAGCCATTTTACC------TATG---TTCGTAAATCGTTGACTATACTCAACAAACCACAAAGACATCGGCA
A-S_scrofa : CATG-A--CATTCACCACGGAAC-T-----GGCAAAAAGAGGGCTTAA-CCTCTGTCTTTAGATTTACAGTCTAATGCTT-AC----TCAGCCATTTTACC------TATG---TTCGTAAATCGTTGACTATACTCAACAAACCACAAAGACATCGGCA
R-O_cuniculus : TATGTTAGTACCCACCTCAGGGCTT-----GGTAAAAAGAGGGCTTAA-CCTCTGTCTTTAGATTTACAGTCTAATACCTAC-----TCGGCCATTTTACCCTTACTTATG---TTCGTCAATCGTTGACTTTTCTCTACCAACCACAAAGACATCGGCA
A-O_cuniculus : TATGTGAGTACCCACCTCAGGGCTT-----GGTAAAAAGAGGGCTTAA-CCTCTGTCTTTAGATTTACAGTCTAATACCTAC-----TCGGCCATTTTACCCTTACTTATG---TTCGTCAATCGTTGACTTTTCTCTACCAACCACAAAGACATCGGCA
R-M_musculus : CATGAA--TA-TCACCTTAAGACCTCT---GGTAAAAAGAGGATTTAAACCTCTGTGTTTAGATTTACAGTCTAATGCTTACT-----CAGCCATTTTACC------TATG---TTCATTAATCGTTGATTATTCTCAACCAATCACAAAGATATCGGAA
A-M_musculus : CATGAA--TA-TCACCTTAAGACCTCT---GGTAAAAAGAGGATTTAAACCTCTGTGTTTAGATTTACAGTCTAATGCTTACT-----CAGCCATTTTACC------TATG---TTCATTAATCGTTGATTATTCTCAACCAATCACAAAGATATCGGAA
R-R_norvegicus : CATGAT--AA-TCACCTTAAGGCTTTTT--GGTAAAAAGGGGGCTCAA-CCCCTGTCTTTAGATTTACAGTCTAATGCTTACT-----CAGCCATTTTACC------TATG---CTCGTAAACCGTTGACTCTTTTCAACTAACCACAAAGATATCGGAA
A-R_norvegicus : CATGAA--AA-TCACCTTAAGGCTTTTT--GGTAAAAAGGGGCCTCAA-CCCCTGTCTTTAGATTTACAGTCTAATGCTTACT-----CAGCCATTTTACC------TATG---TTCGTAAACCGTTGACTCTTTTCAACTAACCACAAAGATATCGGAA
R-M_coypus : CATGAA--AAATCACCTCAAGGCCCTT---GGTAGAAAAAGGAGTTAAACCTTTATCTTTAGATTTACAGTCTAATGCTTATT----TCAGCCATTCTACCACTACTTATG---TTAATTAATCGGTGATTATTCTCCACAAACCACAAAGACATTGGCA
A-M_coypus : CATGAA--AAATCACCTCAAGGCCCTT---GGTAGAAAAAGGAGTTAAACCTTTATCTTTAGATTTACAGTCTAATGCTTATT----TCAGCCATTCTACCACTACTTATG---TTAATTAATCGGTGATTATTCTCCACAAACCACAAAGACATTGGCA
R-A_platyrhynchos : CATGAAC---TTCACTACAGGGCC------GATAAGAAGAGGAATTGAACCTCTGTAAAAAGGACTACAGCCTAACGCTTTAAACACTCAGCCATCTTACC------CGTGACCTTCATCAATCGATGACTATTTTCTACCAATCACAAAGACATCGGTA
A-A_platyrhynchos : CATGAAC---TTCACTACAGGGCC------GATAAGAAGAGGAATTGAACCTCTGTAAAAAGGACTACAGCCTAACGCTTTAAACACTCAGCCATCTTACC------CGTGACCTTCATCAATCGATGACTATTTTCTACCAATCACAAAGACATCGGTA
R-G_gallus : TATGAAC---TTCACTACAGAGTC------GATAAGAAGAGGAATTGAACCTCTGTAAAAAGGACTACAGCCTAACGCTTCAA-CACTCAGCCATCTTACC------TGTGACCTTCATCAACCGATGATTATTCTCAACCAACCACAAAGACATTGGCA
A-G_gallus : TATGAAC---TTCACTACAGAGTC------GATAAGAAGAGGAATTGAACCTCTGTAAAAAGGACTACAGCCTAACGCTTCAA-CACTCAGCCATCTTACC------TGTGACCTTCATCAACCGATGATTATTCTCAACCAACCACAAAGACATTGGCA
 TATGAAMGAATTCACCACAGGGCTTTTTTTGGTAAAAAGAGGACTTAAACCTCTGTCTTTAGATTTACAGTCTAATGCTTAYAACACTCAGCCATTTTACCMYTACTTATGACCTTCATTAATCGATGAYTATTCTCAACCAACCACAAAGACATCGGCA

 * 7060 * 7080 * 7100 * 7120 * 7140 * 7160 * 7180 * 7200
R-E_caballus : CTCTGTACCTCCTATTCGGCGCTTGAGCTGGAATAGTAGGAACTGCCCTAAGCCTCCTAATCCGTGCTGAATTAGGCCAACCTGGGACCCTACTAGGAGATGATCAGATCTACAATGTCATTGTAACCGCCCATGCATTCGTAATAATTTTCTTTATGGT
A-E_caballus : CTCTGTACCTCCTATTCGGCGCTTGAGCTGGAATAGTAGGAACTGCCCTAAGCCTCCTAATCCGTGCTGAATTAGGCCAACCTGGGACCCTACTAGGAGATGATCAGATCTACAATGTTATTGTAACCGCCCATGCATTCGTAATAATTTTCTTTATGGT
R-C_bactrianus : CTCTCTACCTATTATTTGGAGCTTGGGCTGGAATAGTAGGAATAGGACTAAGCTTATTAATTCGCGCTGAATTGGGACAGCCCGGGACGTTGCTTGGAGACGACCAAATCTATAACGTAGTTGTAACAGCTCATGCTTTCGTCATGATCTTCTTTATGGT
A-C_bactrianus : CTCTCTACCTATTATTTGGAGCTTGGGCTGGAATAGTAGGAATAGGACTAAGCTTATTAATTCGCGCTGAATTGGGACAGCCCGGGACGTTGCTTGGAGACGACCAAATCTATAACGTAGTTGTAACAGCTCATGCTTTCGTCATGATCTTCTTTATGGT
R-C_lupus_familiaris : CTTTATACTTACTATTTGGAGCATGAGCCGGTATAGTAGGCACTGCTTTGAGCCTCCTCATCCGAGCCGAACTAGGTCAGCCCGGTACTTTACTAGGTGACGATCAAATTTATAATGTCATCGTAACCGCCCATGCTTTCGTAATAATCTTCTTCATAGT
A-C_lupus_familiaris : CTTTATACTTACTATTTGGAGCATGAGCCGGTATAGTAGGCACTGCCTTGAGCCTCCTCATCCGAGCCGAACTAGGTCAGCCCGGTACTTTACTAGGTGACGATCAAATTTATAATGTCATCGTAACCGCCCATGCTTTCGTAATAATCTTCTTCATAGT
R-N_procyonoides : CTTTATATTTACTATTTGGGGCATGGGCCGGCATAGTAGGCACTGCCTTGAGCCTCCTTATTCGAGCCGAATTAGGTCAGCCTGGCACCCTATTGGGAGACGACCAAATTTATAATGTTGTCGTAACTGCCCATGCTTTCGTGATAATCTTCTTCATGGT
A-N_procyonoides : CTTTATATTTACTATTTGGGGCATGGGCCGGCATAGTAGGCACTGCCTTGAGCCTCCTTATTCGAGCCGAATTAGGTCAGCCTGGCACCCTATTGGGAGACGACCAAATTTATAATGTTGTCGTAACTGCCCATGCTTTCGTGATAATCTTCTTCATGGT
R-V_lagopus : CTTTATACTTGCTGTTTGGAGCATGGGCCGGTATAGTAGGCACCGCCCTAAGCCTTCTGATTCGAGCCGAATTAGGCCAACCTGGCACCTTGTTAGGAGACGATCAGATCTACAACGTAATCGTAACCGCACACGCCTTTGTAATAATCTTCTTTATAGT
A-V_lagopus : CTTTATACTTGCTGTTTGGAGCATGGGCCGGTATAGTAGGCACCGCCCTAAGCCTTCTGATTCGAGCCGAATTAGGCCAACCTGGCACCTTGTTAGGAGACGATCAGATCTACAACGTAATCGTAACCGCACACGCCTTTGTAATAATCTTCTTTATAGT
R-M_putorius : CCCTCTACCTCTTATTTGGTGCATGGGCCGGAATGGTAGGGACCGCTCTCAGTCTACTGATCCGTGCTGAACTAGGTCAACCTGGCACTCTGCTAGGAGACGACCAGATTTATAATGTAATCGTAACTGCTCACGCATTTGTAATAATTTTCTTCATAGT
A-M_putorius : CCCTCTACCTCTTATTTGGTGCATGGGCCGGAATGGTAGGGACCGCTCTCAGTCTACTGATCCGTGCTGAACTAGGTCAACCTGGCACTCTGCTAGGAGACGACCAGATTTATAATGTAATCGTAACTGCTCACGCATTTGTAATAATTTTCTTCATAGT
R-B_taurus : CCCTTTATCTACTATTTGATGCTTGGGCCGGTATAGTAGGAACAGCTCTAAGCCTTCTAATTCGCGCTGAATTAGGCCAACCCGGAACTCTGCTCGGAGACGACCAAATCTACAACGCAGTTGTAACCGCACACGCATTTGTAATAATCTTCTTCATAGT
A-B_taurus : CCCTTTATCTACTATTTGGTGCTTGGGCCGGTATAGTAGGAACAGCTCTAAGCCTTCTAATTCGCGCTGAATTAGGCCAACCCGGAACTCTGCTCGGAGACGACCAAATCTACAACGTAGTTGTAACCGCACACGCATTTGTAATAATCTTCTTCATAGT
R-O_aries : CCCTTTACCTTCTATTTGGTGCCTGAGCTGGTATAGTAGGAACCGCCTTAAGCCTACTAATTCGCGCCGAACTAGGCCAACCCGGAACTCTACTCGGAGATGACCAAATCTACAACGTAATTGTAACCGCACATGCATTTGTAATAATTTTCTTTATAGT
A-O_aries : CCCTTTACCTTCTATTTGGTGCCTGAGCTGGTATAGTAGGAACCGCCTTAAGCCTACTAATTCGCGCCGAACTAGGCCAACCCGGAACTCTACTCGGAGATGACCAAATCTACAACGTAATTGTAACCGCACATGCATTTGTAATAATTTTCTTTATAGT
R-S_scrofa : CCCTGTACCTACTATTTGGTGCCTGAGCAGGAATAGTGGGCACTGCCTTGAGCCTACTAATTCGCGCTGAACTAGGTCAGCCCGGAACCCTACTTGGCGATGATCAAATCTATAATGTAATTGTTACAGCTCATGCCTTTGTAATAATCTTCTTTATAGT
A-S_scrofa : CCCTGTACCTACTATTTGGTGCCTGAGCAGGAATAGTGGGCACTGCCTTGAGCCTACTAATTCGCGCTGAACTAGGTCAGCCCGGAACCCTACTTGGCGATGATCAAATCTATAATGTAATTGTTACAGCTCATGCCTTTGTAATAATCTTCTTTATAGT
R-O_cuniculus : CTCTTTATCTCCTATTTGGAGCTTGAGCTGGGATGGTGGGAACAGCCCTCAGCCTGCTAATTCGAGCAGAATTAGGTCAGCCAGGGACTCTACTCGGGGATGATCAAATCTATAATGTAATCGTCACCGCACATGCCTTTGTAATAATCTTCTTTATAGT
A-O_cuniculus : CTCTTTATCTCCTATTTGGAGCTTGAGCTGGGATGGTGGGAACAGCCCTTAGCCTGCTAATTCGAGCAGAATTAGGTCAGCCAGGGACTCTACTCGGGGATGATCAAATCTATAATGTAATCGTCACCGCACATGCCTTTGTAATAATCTTCTTTATAGT
R-M_musculus : CCCTCTATCTACTATTCGGAGCCTGAGCGGGAATAGTGGGTACTGCACTAAGTATTTTAATTCGAGCAGAATTAGGTCAACCAGGTGCACTTTTAGGAGATGACCAAATTTACAATGTTATCGTAACTGCCCATGCTTTTGTTATAATTTTCTTCATAGT
A-M_musculus : CCCTCTATCTACTATTCGGAGCCTGAGCGGGAATAGTGGGTACTGCACTAAGTATTTTAATTCGAGCAGAATTAGGTCAACCAGGTGCACTTTTAGGAGATGACCAAATTTACAATGTTATCGTAACTGCCCATGCTTTTGTTATAATTTTCTTCATAGT
R-R_norvegicus : CCCTCTACCTATTATTTGGAGCCTGAGCAGGAATAGTAGGGACAGCTTTAAGTATTCTAATTCGAGCTGAACTAGGACAGCCAGGCGCACTCCTAGGAGATGACCAAATCTATAATGTCATCGTCACAGCCCATGCATTCGTAATAATTTTCTTTATAGT
A-R_norvegicus : CCCTCTACCTATTATTTGGAGCCTGAGCAGGAATAGTAGGGACAGCTTTAAGTATTCTAATTCGAGCTGAACTAGGACAGCCAGGCGCACTCCTAGGAGATGACCAAATCTATAATGTCATCGTCACAGCCCATGCATTCGTAATAATTTTCTTTATAGT
R-M_coypus : CCTTATATCTTTTATTTGGAGCCTGAGCTGGAATAGTAGGGACCGCTTTAAGTCTACTAATTCGAGCAGAACTAGGTCAACCAGGTGCATTGCTAGGAGATGACCAAATCTATAATGTAATTGTGACCGCCCACGCATTCGTCATAATTTTCTTCATAGT
A-M_coypus : CCTTATATCTTTTATTTGGAGCCTGAGCTGGAATAGTAGGGACCGCTTTAAGTCTACTAATTCGAGCAGAACTAGGTCAACCAGGTGCATTGCTAGGAGATGACCAAATCTATAATGTAATTGTGACCGCCCACGCATTCGTCATAATTTTCTTCATAGT
R-A_platyrhynchos : CTCTATACCTTATCTTCGGGGCATGAGCCGGAATAATTGGCACAGCACTCAGCCTACTGATCCGGGCAGAACTAGGCCAGCCAGGGACCCTCCTGGGCGACGACCAAATTTATAACGTGATCGTCACCGCTCACGCCTTCGTAATAATCTTCTTCATGGT
A-A_platyrhynchos : CTCTATACCTTATCTTCGGGGCATGAGCCGGAATAATTGGCACAGCACTCAGCCTACTGATCCGGGCAGAACTAGGCCAGCCAGGGACCCTCCTGGGCGACGACCAAATTTATAACGTGATCGTCACCGCTCACGCCTTCGTAATAATCTTCTTCATGGT
R-G_gallus : CTCTTTACCTAATTTTCGGCACATGGGCGGGCATAGCCGGCACAGCACTTAGCCTTCTAATTCGCGCAGAACTAGGACAGCCCGGAACTCTCTTAGGAGACGATCAAATTTACAATGTAATCGTCACAGCCCATGCTTTCGTCATAATCTTCTTTATAGT
A-G_gallus : CTCTTTACCTAATTTTCGGCACATGGGCGGGCATAGCCGGCACAGCACTTAGCCTTCTAATCCGCGCAGAACTAGGACAGCCCGGAACTCTCTTAGGAGACGACCAAATTTACAATGTAATCGTCACAGCCCATGCTTTCGTCATAATCTTCTTTATAGT
 CTCTATACCTACTATTTGGAGCATGAGCCGGAATAGTAGGCACAGCCCTAAGCCTACTAATTCGAGCTGAACTAGGTCAGCCCGGVACTCTACTAGGAGACGACCAAATCTATAATGTAATCGTAACCGCCCATGCATTCGTAATAATCTTCTTTATAGT

 * 7220 * 7240 * 7260 * 7280 * 7300 * 7320 * 7340 * 7360
R-E_caballus : CATACCCATTATAATCGGAGGATTCGGAAACTGATTAGTCCCCCTGATAATTGGAGCACCTGATATAGCTTTCCCCCGAATAAACAACATAAGCTTCTGATTACTTCCCCCATCATTCCTACTTCTTCTCGCTTCCTCAATAATTGAAGCAGGTGCCGGA
A-E_caballus : CATACCCATTATAATCGGAGGATTCGGAAACTGATTAGTCCCCCTGATAATTGGAGCACCTGATATAGCTTTCCCCCGAATAAACAACATAAGCTTCTGATTACTTCCCCCATCATTCCTACTTCTTCTCGCTTCCTCAATAATTGAAGCAGGTGCCGGA
R-C_bactrianus : AATGCCAATCATGATTGGAGGCTTCGGGAACTGACTGGTCCCCCTAATAATTGGAGCCCCGGATATGGCGTTCCCCCGCATGAATAATATGAGCTTCTGACTGCTGCCTCCCTCATTCTTGCTACTACTAGCATCATCTATAGTTGAAGCAGGAGCAGGT
A-C_bactrianus : AATGCCAATCATGATTGGAGGCTTCGGGAACTGACTGGTCCCCCTAATAATTGGAGCCCCGGATATGGCGTTCCCCCGCATGAATAATATGAGCTTCTGACTGCTGCCTCCCTCATTCTTGCTACTACTAGCATCATCTATAGTTGAAGCAGGAGCAGGT
R-C_lupus_familiaris : CATGCCCATCATAATTGGGGGCTTTGGAAACTGACTAGTGCCGTTAATAATTGGTGCTCCGGACATGGCATTCCCCCGAATAAATAACATGAGCTTCTGACTCCTTCCTCCATCCTTTCTTCTACTATTAGCATCTTCTATGGTAGAAGCAGGTGCAGGA
A-C_lupus_familiaris : CATGCCCATCATAATTGGGGGCTTTGGAAACTGACTAGTGCCGTTAATAATTGGTGCTCCGGACATGGCATTCCCCCGAATAAATAACATGAGCTTCTGACTCCTTCCTCCATCCTTTCTTCTACTATTAGCATCTTCTATGGTAGAAGCAGGTGCAGGA
R-N_procyonoides : TATACCCATTATAATTGGAGGGTTCGGAAATTGACTGGTTCCACTGATGATCGGTGCCCCAGACATAGCATTTCCCCGAATAAACAACATGAGCTTTTGATTACTCCCCCCATCCTTTCTTCTATTACTAGCATCCTCTATAGTAGAAGCAGGCGCAGGG
A-N_procyonoides : TATACCCATTATAATTGGAGGGTTCGGAAATTGACTGGTTCCACTGATGATCGGTGCCCCAGACATAGCATTTCCCCGAATAAACAACATGAGCTTTTGATTACTCCCCCCATCCTTTCTTCTATTACTAGCATCCTCTATAGTAGAAGCAGGCGCAGGG
R-V_lagopus : AATACCAATTATAATTGGAGGGTTCGGAAACTGATTAGTTCCCTTAATAATTGGTGCTCCTGACATAGCATTCCCTCGAATAAACAACATAAGCTTCTGACTACTTCCCCCATCCTTTCTTCTATTATTAGCATCTTCCATAGTAGAAGCGGGCGCGGGA
A-V_lagopus : AATACCAATTATAATTGGAGGGTTCGGAAACTGATTAGTTCCCTTAATAATTGGTGCTCCTGACATAGCATTCCCTCGAATAAACAACATAAGCTTCTGACTACTTCCCCCATCCTTTCTTCTATTATTAGCATCTTCCATAGTAGAAGCGGGCGCGGGA
R-M_putorius : AATACCCATCATGCTTGGGGGCTTTGGGAACTGGCTTATTCCTCTAATAATCGGCGCACCTGACATAGCATTCCCACGGATAAACAACATAAGCTTCTGGCTTCTTCCGCCCTCTTTTCTTCTCCTACTAGCTTCCTCTATGGTAGAAGCAGGTGCAGGG
A-M_putorius : AATACCCATCATGCTTGGGGGCTTTGGGAACTGGCTTATTCCTCTAATAATCGGCGCACCTGACATAGCATTCCCACGGATAAACAACATAAGCTTCTGGCTTCTTCCGCCCTCTTTTCTTCTCCTACTAGCTTCCTCTATGGTAGAAGCAGGTGCAGGG
R-B_taurus : AATACCAATCATAATTGGAGGATTCGGTAACTGACTTGTTCCCCTAATAATTGGTGCTCCCGATATAGCATTTCCCCGAATAAATAATATAAGCTTCTGACTCCTCCCTCCCTCATTCCTACTACTCCTCGCATCCTCTATAGTTGAAGCTGGGGCAGGA
A-B_taurus : AATACCAATCATAATTGGAGGATTCGGTAACTGACTTGTTCCCCTAATAATTGGTGCTCCCGATATAGCATTTCCCCGAATAAATAATATAAGCTTCTGACTCCTCCCTCCCTCATTCCTACTACTCCTCGCATCCTCTATAGTTGAAGCTGGGGCAGGA
R-O_aries : AATGCCTATTATAATCGGTGGATTCGGCAACTGACTAGTTCCTCTGATAATTGGAGCCCCTGATATAGCATTTCCTCGGATAAATAACATAAGCTTTTGACTTCTTCCCCCATCTTTCCTGTTACTCCTAGCATCCTCTATGGTTGAGGCCGGAGCAGGA
A-O_aries : AATGCCTATTATAATCGGTGGATTTGGCAACTGACTAGTTCCTCTGATAATTGGAGCCCCTGATATAGCATTTCCTCGGATAAATAACATAAGCTTTTGACTTCTTCCCCCATCTTTCCTGTTACTCCTAGCATCCTCTATGGTTGAGGCCGGAGCAGGA
R-S_scrofa : AATACCCATTATGATTGGGGGTTTTGGTAACTGACTCGTACCGCTAATAATCGGAGCTCCCGATATGGCCTTTCCACGTATAAACAACATAAGTTTCTGACTACTTCCACCATCCTTCCTATTACTACTGGCATCCTCAATAGTAGAAGCCGGGGCGGGT
A-S_scrofa : AATACCCATTATGATTGGGGGTTTTGGTAACTGACTCGTACCGCTAATAATCGGAGCTCCCGATATGGCCTTTCCACGTATAAACAACATAAGTTTCTGACTACTTCCACCATCCTTCCTATTACTACTGGCATCCTCAATAGTAGAAGCCGGGGCGGGT
R-O_cuniculus : CATGCCTATTATAATTGGAGGCTTCGGGAACTGGCTTGTCCCCCTGATAATTGGGGCTCCTGACATAGCCTTCCCCCGAATAAATAATATGAGCTTCTGACTTCTCCCCCCTTCATTCCTTCTTCTACTAGCCTCCTCAATAGTAGAAGCTGGGGCGGGG
A-O_cuniculus : CATACCTATTATAATTGGAGGCTTCGGGAACTGGCTTGTCCCCCTGATAATTGGGGCTCCTGACATAGCCTTCCCCCGAATAAATAATATGAGCTTCTGACTTCTCCCCCCTTCATTCCTTCTTCTACTAGCCTCCTCAATAGTAGAAGCTGGGGCGGGG
R-M_musculus : AATACCAATAATAATTGGAGGCTTTGGAAACTGACTTGTCCCACTAATAATCGGAGCCCCAGATATAGCATTCCCACGAATAAATAATATAAGTTTTTGACTCCTACCACCATCATTTCTCCTTCTCCTAGCATCATCAATAGTAGAAGCAGGAGCAGGA
A-M_musculus : TATACCAATAATAATTGGAGGCTTTGGAAACTGACTTGTCCCACTAATAATCGGAGCCCCAGATATAGCATTCCCACGAATAAATAATATAAGTTTTTGACTCCTACCACCATCATTTCTCCTTCTCCTAGCATCATCAATAGTAGAAGCAGGAGCAGGA
R-R_norvegicus : AATACCTATAATAATTGGAGGCTTCGGGAACTGACTTGTACCACTAATAATTGGAGCCCCTGATATAGCATTCCCACGAATAAATAACATAAGCTTTTGACTGCTTCCTCCATCATTTCTACTCCTTTTAGCATCCTCCATAGTAGAAGCTGGAGCTGGA
A-R_norvegicus : AATACCTATAATAATTGGAGGCTTCGGGAACTGACTTGTACCACTAATAATTGGAGCCCCTGATATAGCATTCCCACGAATAAATAACATAAGCTTTTGACTGCTTCCTCCATCATTTCTACTCCTTTTAGCATCCTCCATAGTAGAAGCTGGAGCTGGA
R-M_coypus : CATACCCATTATGATTGGAGGTTTCGGAAACTGACTTGTCCCTTTAATAATTGGAGCTCCTGATATAGCCTTTCCACGAATAAATAATATAAGCTTTTGGTTATTACCACCATCGTTTCTTTTACTTCTAGCTTCTTCAATAGTAGAAGCCGGAGCCGGG
A-M_coypus : CATACCCATTATGATTGGAGGTTTCGGAAACTGACTTGTCCCTTTAATAATTGGAGCTCCTGATATAGCCTTTCCACGAATAAATAATATAAGCTTTTGGTTATTACCACCATCGTTTCTTTTACTTCTAGCTTCTTCAATAGTAGAAGCCGGAGCCGGG
R-A_platyrhynchos : AATGCCCATCATAATTGGAGGGTTCGGCAACTGATTGGTCCCCCTGATAATCGGTGCCCCCGACATAGCATTCCCACGAATAAACAACATAAGCTTCTGACTCCTCCCACCATCATTCCTCCTTCTACTCGCCTCATCCACTGTAGAAGCTGGCGCTGGT
A-A_platyrhynchos : AATGCCCATCATAATTGGAGGGTTCGGCAACTGATTGGTCCCCCTGATAATCGGTGCCCCCGACATAGCATTCCCACGAATAAACAACATAAGCTTCTGACTCCTCCCACCATCATTCCTCCTTCTACTCGCCTCATCCACTGTAGAAGCTGGCGCTGGT
R-G_gallus : TATACCCATCATGATCGGTGGCTTCGGAAACTGACTAGTCCCACTTATAATCGGTGCCCCAGACATAGCATTCCCCCGCATAAATAACATAAGCTTCTGACTCCTCCCTCCCTCCTTCCTTCTCCTACTAGCCTCATCTACCGTAGAAGCTGGGGCCGGC
A-G_gallus : TATACCCATCATGATCGGTGGCTTCGGAAACTGACTAGTCCCGCTTATAATCGGTGCCCCAGACATAGCATTCCCCCGCATAAATAACATAAGCTTCTGACTCCTCCCTCCCTCCTTCCTTCTCCTACTAGCCTCATCTACCGTAGAAGCTGGGGCCGGC
 AATACCCATTATAATTGGAGGCTTCGGAAACTGACTTGTCCCCCTAATAATTGGAGCCCCTGATATAGCATTCCCCCGAATAAATAACATAAGCTTCTGACTMCTTCCYCCATCATTCCTTCTACTACTAGCATCCTCTATAGTAGAAGCAGGAGCAGGA

 * 7380 * 7400 * 7420 * 7440 * 7460 * 7480 * 7500 * 7520
R-E_caballus : ACAGGCTGAACCGTATATCCTCCTCTAGCTGGAAATCTGGCGCATGCAGGAGCCTCTGTTGACTTAACCATTTTCTCTCTCCACCTAGCTGGGGTGTCCTCGATTTTAGGTGCCATCAACTTTATTACCACAATCATTAACATAAAACCACCAGCCCTAT
A-E_caballus : ACAGGCTGAACCGTATATCCTCCTCTAGCTGGAAATCTGGCGCATGCAGGAGCCTCTGTTGACTTAACCATTTTCTCTCTCCACCTGGCTGGGGTGTCCTCGATTTTAGGTGCCATCAACTTTATTACCACAATCATTAACATAAAACCACCAGCTCTAT
R-C_bactrianus : ACAGGCTGAACCGTTTACCCTCCCCTAGCCGGAAACCTGGCACACGCAGGCGCCTCCGTCGATTTAACTATTTTCTCCCTGCATTTAGCGGGAGTATCTTCAATTCTAGGGGCTATTAACTTTATTACCACTATTATCAATATAAAACCACCTGCTATAT
A-C_bactrianus : ACAGGCTGAACCGTTTACCCTCCCCTAGCCGGAAACCTGGCACACGCAGGCGCCTCCGTCGATTTAACTATTTTCTCCCTGCATTTAGCGGGAGTATCTTCAATTCTAGGGGCTATTAACTTTATTACCACTATTATCAATATAAAACCACCTGCTATAT
R-C_lupus_familiaris : ACGGGATGAACCGTATACCCCCCACTGGCTGGCAATCTGGCCCATGCAGGAGCATCCGTTGACCTTACAATTTTCTCCTTACACTTAGCCGGAGTCTCTTCTATTTTAGGGGCAATTAATTTCATCACTACTATTATCAACATAAAACCCCCTGCAATAT
A-C_lupus_familiaris : ACGGGATGAACCGTATACCCCCCACTGGCTGGCAATCTGGCCCATGCAGGAGCATCCGTTGACCTTACAATTTTCTCCTTACACTTAGCCGGAGTCTCTTCTATTTTAGGGGCAATTAATTTCATCACTACTATTATCAACATAAAACCCCCTGCAATAT
R-N_procyonoides : ACAGGATGGACTGTGTACCCCCCATTAGCTGGCAACCTAGCCCATGCAGGAGCATCAGTAGACCTTACAATCTTCTCCTTACACCTTGCCGGGGTATCCTCAATTCTAGGGGCAATTAATTTTATTACCACAATTATCAATATAAAACCTCCTGCAATAT
A-N_procyonoides : ACAGGATGGACTGTGTACCCCCCATTAGCTGGCAACCTAGCCCATGCAGGAGCATCAGTAGACCTTACAATCTTCTCCTTACACCTTGCCGGGGTATCCTCAATTCTAGGGGCAATTAATTTTATTACCACAATTATCAATATAAAACCTCCTGCAATAT
R-V_lagopus : ACAGGGTGAACCGTGTATCCACCACTAGCTGGCAACCTGGCTCACGCCGGAGCATCAGTAGATCTTACAATTTTCTCTCTTCACCTGGCCGGAGTCTCTTCAATTCTAGGGGCCATTAATTTTATTACTACTATTATTAATATAAAACCTCCTGCCATAT
A-V_lagopus : ACAGGGTGAACCGTGTATCCACCACTAGCTGGCAACCTGGCTCACGCCGGAGCATCAGTAGATCTTACAATTTTCTCTCTTCACCTGGCCGGAGTCTCTTCAATTCTAGGGGCCATTAATTTTATTACTACTATTATTAATATAAAACCTCCTGCCATAT
R-M_putorius : ACTGGATGAACTGTATACCCCCCTTTAGCAGGAAATCTAGCACATGCTGGAGCATCCGTGGACCTGGTAATCTTTTCTCTACACTTAGCTGGTGTTTCATCTATCTTAGGGTCAATCAACTTTATTACTACTATTATCAACATGAAACCGCCTGCCATGT
A-M_putorius : ACTGGATGAACTGTATACCCCCCTTTAGCAGGAAATCTAGCACATGCTGGAGCATCCGTGGACCTGGTAATCTTTTCTCTACACTTAGCTGGTGTTTCATCTATCTTAGGGTCAATCAACTTTATTACTACTATTATCAACATGAAACCGCCTGCCATGT
R-B_taurus : ACAGGCTGAACCGTGTACCCTCCCTTAGCAGGCAACCTAGCCCATGCAGGAGCTTCAGTAGATCTAACCATTTTCTCTTTACACTTAGCAGGAGTTTCCTCAATTTTAGGAGCCATCAACTTCATTACAACAATTATCAACATAAAGCCCCCCGCAATGT
A-B_taurus : ACAGGCTGAACCGTGTACCCTCCCTTAGCAGGCAACCTAGCCCATGCAGGAGCTTCAGTAGATCTAACCATTTTCTCTTTACACTTAGCAGGAGTTTCCTCAATTTTAGGAGCCATCAACTTCATTACAACAATTATCAACATAAAGCCCCCCGCAATGT
R-O_aries : ACAGGTTGAACCGTATACCCTCCTCTAGCAGGCAACCTAGCCCATGCAGGAGCCTCAGTAGATCTAACTATTTTCTCCCTACATCTGGCAGGTGTCTCTTCAATTCTAGGAGCCATTAATTTTATTACAACTATTATTAATATAAAACCCCCTGCGATGT
A-O_aries : ACAGGTTGAACCGTATACCCTCCTCTAGCAGGCAACCTAGCCCATGCAGGAGCCTCAGTAGATCTAACTATTTTCTCCCTACACCTGGCAGGTGTCTCTTCAATTCTAGGAGCCATTAATTTTATTACAACTATTATTAATATAAAACCCCCTGCGATGT
R-S_scrofa : ACTGGATGAACCGTATACCCACCTTTAGCTGGAAACTTAGCCCATGCAGGAGCTTCAGTTGATCTAACAATTTTCTCCCTACACCTTGCAGGTGTATCATCAATCCTAGGGGCTATTAATTTCATTACCACAATTATTAACATAAAACCTCCCGCAATGT
A-S_scrofa : ACTGGATGAACCGTATACCCACCTTTAGCCGGAAACTTAGCCCATGCAGGAGCTTCAGTTGATCTAACAATTTTCTCCCTACACCTTGCAGGTGTATCATCAATCCTAGGGGCTATTAATTTCATTACCACAATTATTAACATAAAACCTCCCGCAATGT
R-O_cuniculus : ACTGGCTGAACTGTTTATCCACCTCTAGCCGGTAATCTTGCACATGCTGGAGCCTCAGTGGATCTTACTATTTTCTCCCTTCACTTAGCTGGAGTATCATCTATTTTAGGGGCTATTAACTTTATTACAACTATTATTAATATGAAAGCCCCTGCAATAT
A-O_cuniculus : ACTGGCTGAACTGTTTATCCACCTCTAGCCGGTAATCTTGCACATGCTGGAGCCTCAGTGGATCTTACTATTTTCTCCCTTCACTTAGCTGGAGTATCATCTATTTTAGGGGCTATTAACTTTATTACAACTATTATTAATATGAAACCCCCTGCAATAT
R-M_musculus : ACAGGATGAACAGTCTACCCACCTCTAGCCGGAAATCTAGCCCATGCAGGAGCATCAGTAGACCTAACAATTTTCTCCCTTCATTTAGCTGGAGTGTCATCTATTTTAGGTGCAATTAATTTTATTACCACTATTATCAACATGAAACCCCCAGCCATAA
A-M_musculus : ACAGGATGAACAGTCTACCCACCTCTAGCCGGAAATCTAGCCCATGCAGGAGCATCAGTAGACCTAACAATTTTCTCCCTTCATTTAGCTGGAGTGTCATCTATTTTAGGTGCAATTAATTTTATTACCACTATTATCAACATGAAACCCCCAGCCATAA
R-R_norvegicus : ACAGGATGAACAGTATATCCCCCCTTAGCCGGAAACCTAGCCCATGCTGGAGCATCCGTAGATTTAACTATTTTTTCCCTCCACCTAGCCGGGGTGTCTTCTATCTTAGGAGCTATCAACTTTATCACCACTATCATTAATATAAAACCCCCTGCTATAA
A-R_norvegicus : ACAGGATGAACAGTATACCCCCCCTTAGCCGGAAACCTAGCCCATGCTGGAGCATCCGTAGATTTAACTATTTTTTCCCTCCACCTAGCCGGGGTGTCTTCTATCTTAGGAGCTATCAACTTTATCACCACTATCATTAATATAAAACCCCCTGCTATAA
R-M_coypus : ACTGGATGAACCGTTTATCCCCCACTAGCAGGCAATATAGCCCATGCAGGGGCCTCCGTCGATCTAACTATCTTTTCTTTACATTTAGCAGGAGTTTCCTCAATTCTTGGTGCTATTAATTTTATTACTACTATCATCAATATAAAACCACCTGCTCTAA
A-M_coypus : ACTGGATGAACCGTTTATCCCCCACTAGCAGGCAATATAGCCCATGCAGGGGCCTCCGTCGATCTAACTATCTTTTCTTTACATTTAGCAGGAGTTTCCTCAATTCTTGGTGCTATTAATTTTATTACTACTATCATCAATATAAAACCACCTGCTCTAA
R-A_platyrhynchos : ACGGGTTGAACCGTATACCCACCTCTAGCAGGCAACCTAGCCCACGCCGGAGCCTCAGTGGACCTGGCTATCTTCTCACTTCACCTGGCTGGTGTCTCCTCCATCCTCGGAGCCATTAACTTCATTACCACAGCCATCAACATAAAACCCCCCGCACTCT
A-A_platyrhynchos : ACGGGTTGAACCGTATACCCACCTCTAGCAGGCAACCTAGCCCACGCCGGAGCCTCAGTGGACCTGGCTATCTTCTCACTTCACCTGGCTGGTGTCTCCTCCATCCTCGGAGCCATTAACTTCATTACCACAGCCATCAACATAAAACCCCCCGCACTCT
R-G_gallus : ACAGGATGGACAGTTTACCCCCCTTTAGCCGGCAACCTAGCCCACGCTGGCGCATCAGTAGACCTAGCCATCTTT-CATTAC--TTAGCAGGTGTTTCCTCCATTCTAGGAGCCATCAACTTTATCACTACCATCATCAACATAAAACCCCCCGCACTGT
A-G_gallus : ACAGGATGGACAGTTTACCCCCCTTTAGCCGGCAACCTAGCCCACGCTGGCGCATCAGTAGACCTAGCCATCTTTTCATTACACTTAGCAGGTGTTTCCTCCATTCTAGGAGCCATCAACTTTATCACTACCATCATCAACATAAAACCCCCCGCACTGT
 ACAGGATGAACCGTATACCCCCCTCTAGCCGGCAACCTAGCCCATGCAGGAGCATCAGTAGATCTAACWATTTTCTCCCTACACTTAGCWGGAGTHTCCTCAATTCTAGGGGCCATTAACTTTATTACCACTATTATCAACATAAAACCCCCTGCAATAT

 * 7540 * 7560 * 7580 * 7600 * 7620 * 7640 * 7660 * 7680
R-E_caballus : CCCAATATCAAACCCCCCTATTCGTTTGATCTGTCCTTATTACGGCAGTACTCCTTCTCCTAGCCCTCCCGGTCCTAGCAGCAGGCATTACCATGCTTCTCACAGACCGTAACCTGAACACTACTTTCTTCGACCCCGCAGGAGGAGGGGATCCAATCCT
A-E_caballus : CCCAATATCAAACCCCCCTATTCGTTTGATCTGTCCTTATTACGGCAGTACTCCTTCTCCTAGCCCTCCCGGTCCTAGCAGCAGGCATTACCATGCTTCTCACAGACCGTAACCTAAACACTACTTTCTTCGACCCCGCAGGAGGAGGGGATCCAATCCT
R-C_bactrianus : CCCAATACCAAACCCCTCTATTTGTCTGATCCGTTCTAATCACTGCCGTTCTCTTACTACTCTCCCTTCCGGTACTGGCTGCCGGAATCACAATACTATTAACAGATCGTAACCTAAATACGACTTTCTTTGATCCTGCAGGAGGAGGGGATCCCATCCT
A-C_bactrianus : CCCAATACCAAACCCCTCTATTTGTCTGATCCGTTCTAATCACTGCCGTTCTCTTACTACTCTCCCTTCCGGTACTGGCTGCCGGAATCACAATACTATTAACAGATCGTAACCTAAATACGACTTTCTTTGATCCTGCAGGAGGAGGGGATCCCATCCT
R-C_lupus_familiaris : CCCAGTATCAAACTCCCCTGTTTGTATGATCAGTACTAATTACAGCAGTTCTACTCTTACTATCCCTGCCTGTACTGGCTGCTGGAATTACAATACTTTTAACAGACCGGAATCTTAATACAACATTTTTTGATCCCGCTGGAGGAGGAGACCCTATCCT
A-C_lupus_familiaris : CCCAGTATCAAACTCCCCTGTTTGTATGATCAGTACTAATTACAGCAGTTCTACTCTTACTATCCCTGCCTGTACTGGCTGCTGGAATTACAATACTTTTAACAGACCGGAATCTTAATACAACATTTTTTGATCCCGCTGGAGGAGGAGACCCTATCCT
R-N_procyonoides : CCCAATATCAAACCCCTTTATTCGTATGATCAGTATTAATTACAGCAGTCCTATTACTACTATCACTACCCGTACTGGCCGCAGGAATCACAATACTCCTAACGGATCGAAACCTTAACACAACCTTTTTTGACCCTGCCGGAGGGGGAGACCCAATCTT
A-N_procyonoides : CCCAATATCAAACCCCTTTATTCGTATGATCAGTATTAATTACAGCAGTCCTATTACTACTATCACTACCCGTACTGGCCGCAGGAATCACAATACTCCTAACGGATCGAAACCTTAACACAACCTTTTTTGACCCTGCCGGAGGGGGAGACCCAATCTT
R-V_lagopus : CCCAATACCAAACCCCATTATTTGTATGATCAGTCCTAATTACAGCGGTTCTATTGCTATTATCGCTACCGGTACTAGCTGCTGGGATTACTATACTTCTAACGGATCGCAACCTTAACACAACATTTTTCGATCCTGCTGGAGGAGGGGATCCTATTTT
A-V_lagopus : CCCAATACCAAACCCCATTATTTGTATGATCAGTCCTAATTACAGCGGTTCTATTGCTATTATCGCTACCGGTACTAGCTGCTGGGATTACTATACTTCTAACGGATCGCAACCTTAACACAACATTTTTCGATCCTGCTGGAGGAGGGGATCCTATTTT
R-M_putorius : CACAATACCAAACTCCACTATTTGTTTGATCAGTTTTAATTACAGCCGTACTTCTTCTTCTGTCCCTGCCAGTTTTAGCAGCCGGCATTACTATATTACTTACAGACCGTAATCTAAATACTACTTTCTTTGACCCAGCCGGAGGGGGGGACCCTATCCT
A-M_putorius : CACAATACCAAACTCCACTATTTGTTTGATCAGTTTTAATTACAGCCGTACTTCTTCTTCTGTCCCTGCCAGTTTTAGCAGCCGGCATTACTATATTACTTACAGACCGTAATCTAAATACTACTTTCTTTGACCCAGCCGGAGGGGGGGACCCTATCCT
R-B_taurus : CACAATACCAAACCCCTCTGTTCGTATGATCCGTAATAATTACCGCCGTACTACTACTACTCTCGCTCCCTGTATTAGCAGCCGGCATCACAATGCTATTAACAGACCGGAACCTAAATACAACCTTCTTCGACCCGGCAGGAGGAGGAGACCCTATTCT
A-B_taurus : CACAATACCAAACCCCTCTGTTCGTATGATCCGTAATAATTACCGCCGTACTACTACTACTCTCGCTCCCTGTATTAGCAGCCGGCATCACAATGCTATTAACAGACCGGAACCTAAATACAACCTTCTTCGACCCGGCAGGAGGAGGAGACCCTATTCT
R-O_aries : CACAGTATCAAACCCCCTTGTTTGTATGATCTGTACTAATTACTGCCGTACTTCTCCTTCTCTCACTTCCTGTATTAGCAGCTGGTATCACAATACTACTAACGGACCGAAACCTGAATACAACCTTTTTTGACCCAGCAGGAGGAGGAGACCCTATCCT
A-O_aries : CACAATATCAAACCCCCTTGTTTGTATGATCTGTACTAATTACTGCCGTACTTCTCCTTCTCTCACTTCCTGTATTAGCAGCTGGTATCACAATACTACTAACGGACCGAAACCTGAATACAACCTTTTTTGACCCAGCAGGAGGAGGAGACCCTATCCT
R-S_scrofa : CTCAATACCAAACACCCCTGTTTGTCTGATCAGTACTAATCACAGCCGTACTACTTCTACTATCCCTGCCAGTTCTAGCAGCTGGCATTACTATACTACTGACAGACCGCAACCTGAACACAACCTTTTTTGATCCAGCAGGTGGTGGAGACCCTATCCT
A-S_scrofa : CTCAATACCAAACACCCCTGTTTGTCTGATCAGTACTAATCACAGCCGTACTACTTCTACTATCCCTGCCAGTTCTAGCAGCTGGCATTACTATACTACTGACAGACCGCAACCTGAACACAACCTTTTTTGATCCAGCAGGTGGTGGAGACCCTATCCT
R-O_cuniculus : CTCAATATCAAACCCCCTTATTCGTATGATCTGTTCTAATCACAGCCGTACTTCTTCTTCTCTCTTTACCAGTCCTAGCTGCTGGCATTACAATGCTTTTAACAGACCGAAACTTAAATACAACCTTCTTTGATCCTGCAGGAGGAGGAGACCCTATCCT
A-O_cuniculus : CTCAATATCAAACCCCCTTATTCGTATGATCTGTTCTAATCACAGCCGTACTTCTTCTTCTCTCTTTACCGGTCCTAGCTGCTGGCATTACAATGCTTTTAACAGACCGAAACTTAAATACAACCTTCTTTGATCCTGCAGGAGGAGGAGACCCTATCCT
R-M_musculus : CACAGTATCAAACTCCACTATTTGTCTGATCCGTACTTATTACAGCCGTACTGCTCCTATTATCACTACCAGTGCTAGCCGCAGGCATTACTATACTACTAACAGACCGCAACCTAAACACAACTTTCTTTGATCCCGCTGGAGGAGGGGACCCAATTCT
A-M_musculus : CACAGTATCAAACTCCACTATTTGTCTGATCCGTACTTATTACAGCCGTACTGCTCCTATTATCACTACCAGTGCTAGCCGCAGGCATTACTATACTACTAACAGACCGCAACCTAAACACAACTTTCTTTGATCCCGCTGGAGGAGGGGACCCAATTCT
R-R_norvegicus : CCCAATATCAGACACCTCTCTTTGTATGATCCGTACTAATTACAGCCGTCCTACTACTTCTCTCACTGCCAGTATTAGCAGCAGGTATCACTATACTCCTTACAGACCGAAATCTAAATACTACTTTCTTCGACCCCGCTGGAGGTGGAGACCCAATCCT
A-R_norvegicus : CCCAATATCAGACACCTCTCTTTGTATGATCCGTACTAATTACAGCCGTCCTACTACTTCTCTCACTGCCAGTATTAGCAGCAGGTATCACTATACTCCTTACAGACCGAAATCTAAATACTACTTTCTTCGACCCCGCTGGAGGTGGAGACCCAATTCT
R-M_coypus : CGCAATATCAAACCCCTCTGTTCGTATGATCAGTACTAATTACTGCTGTTCTTCTTCTCCTCTCACTTCCTGTTTTAGCAGCCGGGATTACTATATTATTAACAGACCGTAACTTAAACACAACTTTCTTTGACCCTGCTGGCGGAGGAGATCCCGTTTT
A-M_coypus : CGCAATATCAAACCCCTCTGTTCGTATGATCAGTACTAATTACTGCTGTTCTTCTTCTCCTCTCACTTCCTGTTTTGGCAGCCGGGATTACTATATTATTAACAGACCGTAACTTAAACACAACTTTCTTTGACCCTGCTGGCGGAGGAGATCCCGTTTT
R-A_platyrhynchos : CACAATACCAAACCCCACTTTTCGTCTGATCAGTCCTAATTACCGCCATCCTGCTCCTCCTATCACTCCCCGTCCTCGCCGCCGGCATCACAATGCTACTAACCGACCGAAACCTAAACACCACATTCTTTGATCCTGCCGGAGGGGGAGACCCAATCCT
A-A_platyrhynchos : CACAATACCAAACCCCACTTTTCGTCTGATCAGTCCTAATTACCGCCATCCTGCTCCTCCTATCACTCCCCGTCCTCGCCGCCGGCATCACAATGCTACTAACCGACCGAAACCTAAACACCACATTCTTTGATCCTGCCGGAGGGGGAGACCCAATCCT
R-G_gallus : CACAATACCAAACACCCCTATTCGTATGATCCGTCCTCATTACTGCCATCCTACTACTCCTCTCCTTACCCGTCCTAGCAGCTGGGATTACCATACTACTTACCGACCGCAACCTTAACACCACATTCTTCGACCCAGCTGGAGGAGGAGACCCAATCCT
A-G_gallus : CACAATACCAAACACCCCTATTCGTATGATCCGTCCTCATTACTGCCATCCTACTACTCCTCTCCTTACCCGTCCTAGCAGCTGGGATTACCATACTACTTACCGACCGCAACCTTAACACCACATTCTTCGACCCAGCTGGAGGAGGAGACCCAATCCT
 CMCAATATCAAACCCCCCTATTTGTATGATCAGTACTAATTACAGCCGTACTACTWCTACTMTCMCTACCAGTACTAGCAGCTGGCATTACAATACTACTAACAGACCGAAACCTAAACACAACTTTCTTTGACCCTGCWGGAGGAGGAGACCCTATCCT

 * 7700 * 7720 * 7740 * 7760 * 7780 * 7800 * 7820 * 7840
R-E_caballus : TTATCAACACCTATTCTGATTCTTCGGACACCCCGAAGTCTATATTCTTATCCTACCAGGCTTCGGTATAATCTCACACATCGTCACATACTACTCAGGTAAAAAGGAACCTTTTGGCTACATGGGTATAGTGTGAGCTATAATATCCATTGGCTTTCTA
A-E_caballus : TTATCAACACCTATTCTGATTCTTCGGACACCCCGAAGTCTATATTCTTATCCTACCAGGCTTCGGTATAATCTCACACATCGTCACATACTACTCAGGTAAAAAGGAACCTTTTGGCTACATGGGTATAGTGTGAGCTATAATATCCATTGGCTTTCTA
R-C_bactrianus : TTACCAACACCTATTTTGATTCTTCGGTCACCCAGAGGTTTACATCCTAATCTTACCTGGCTTTGGAATAATTTCTCACATTGTCACTTATTACTCTGGAAAAAAGGAGCCCTTTGGTTATATGGGAATAGTCTGGGCTATAATGTCTATCGGCTTCCTA
A-C_bactrianus : TTACCAACACCTATTTTGATTCTTCGGTCACCCAGAGGTTTACATCCTAATCTTACCTGGCTTTGGAATAATTTCTCACATTGTCACTTATTACTCTGGAAAAAAGGAGCCCTTTGGTTATATGGGAATAGTCTGGGCTATAATGTCTATCGGCTTCCTA
R-C_lupus_familiaris : ATATCAACACCTATTCTGATTCTTCGGACATCCTGAAGTTTACATTCTTATCCTGCCCGGATTCGGAATAATTTCTCACATTGTCACTTACTACTCAGGGAAAAAAGAGCCTTTCGGTTATATAGGAATAGTATGAGCAATAATATCTATTGGGTTTTTA
A-C_lupus_familiaris : ATATCAACACCTATTCTGATTCTTCGGGCATCCTGAAGTTTACATTCTTATCCTGCCCGGATTCGGAATAATTTCTCACATTGTCACTTACTACTCAGGGAAAAAAGAGCCTTTCGGTTATATAGGAATAGTATGAGCAATAATATCTATTGGGTTTTTA
R-N_procyonoides : ATATCAACACTTATTCTGATTCTTTGGACATCCTGAGGTTTATATCTTAATCTTGCCTGGGTTTGGAATAATTTCCCATATCGTTACATACTACTCCGGGAAAAAAGAACCTTTTGGTTACATAGGAATAGTCTGAGCAATAATATCTATTGGATTCTTA
A-N_procyonoides : ATATCAACACTTATTCTGATTCTTTGGACATCCTGAGGTTTATATCTTAATCTTGCCTGGGTTTGGAATAATTTCCCATATCGTTACATACTACTCCGGGAAAAAAGAACCTTTTGGTTACATAGGAATAGTCTGAGCAATAATATCTATTGGATTCTTA
R-V_lagopus : ATACCAACACTTGTTTTGATTCTTCGGACACCCCGAGGTCTATATTTTAATCTTGCCTGGGTTTGGTATAATCTCCCACATCGTCACTTACTATTCAGGGAAAAAAGAACCCTTTGGTTATATGGGAATGGTATGAGCAATAATGTCTATCGGGTTTTTA
A-V_lagopus : ATACCAACACTTGTTTTGATTCTTCGGACACCCCGAGGTCTATATTTTAATCTTGCCTGGGTTTGGTATAATCTCCCACATCGTCACTTACTATTCAGGGAAAAAAGAACCCTTTGGTTATATGGGAATGGTATGAGCAATAATGTCTATCGGGTTTTTA
R-M_putorius : GTACCAACACTTATTTTGATTCTTTGGGCACCCAGAAGTTTATATCCTGATTCTCCCAGGATTTGGTATCATTTCACACGTCGTAACTTACTACTCAGGAAAAAAAGAACCATTTGGTTATATGGGAATAGTATGGGCAATAATATCAATTGGTTTCCTA
A-M_putorius : GTACCAACACTTATTTTGATTCTTTGGGCACCCAGAAGTTTATATCCTGATTCTCCCAGGATTTGGTATCATTTCACACGTCGTAACTTACTACTCAGGAAAAAAAGAACCATTTGGTTATATGGGAATAGTATGGGCAATAATATCAATTGGTTTCCTA
R-B_taurus : ATATCAACACTTATTCTGATTCTTTGGACACCCCGAAGTCTATATTTTAATCTTACCTGGGTTTGGAATAATCTCTCATATCGTGACCTACTACTCAGGAAAAAAAGAACCATTCGGATATATGGGAATAGTTTGGGCTATAATGTCAATCGGATTTCTA
A-B_taurus : ATATCAACACTTATTCTGATTCTTTGGACACCCCGAAGTCTATATTTTAATCTTACCTGGGTTTGGAATAATCTCTCATATCGTGACCTACTACTCAGGAAAAAAAGAACCATTCGGATATATGGGAATAGTTTGGGCTATAATGTCAATCGGATTTCTA
R-O_aries : ATATCAACACCTATTCTGATTCTTTGGGCACCCTGAAGTATATATTCTTATTTTACCTGGGTTTGGGATAATCTCCCATATTGTGACCTACTATTCAGGAAAAAAAGAACCATTCGGATATATAGGAATAGTATGAGCCATAATATCAATTGGGTTCCTA
A-O_aries : ATATCAACACCTATTCTGATTCTTTGGGCACCCTGAAGTATATATTCTTATTTTACCTGGGTTTGGGATAATCTCCCATATTGTGACCTACTATTCAGGAAAAAAAGAACCATTCGGATATATAGGAATAGTATGAGCCATAATATCAATTGGGTTCCTA
R-S_scrofa : TTATCAACACTTGTTCTGATTTTTCGGACACCCAGAAGTATATATTCTCATCTTACCAGGGTTCGGAATAATCTCCCACATTGTAACCTACTATTCAGGTAAAAAAGAACCATTTGGATATATAGGCATAGTATGAGCCATAATGTCCATTGGATTCTTA
A-S_scrofa : TTATCAACACTTGTTCTGATTTTTCGGACACCCAGAAGTATATATTCTCATCTTACCAGGGTTCGGAATAATCTCCCACATTGTAACCTACTATTCAGGTAAAAAAGAACCATTTGGATATATAGGCATAGTATGAGCCATAATGTCCATTGGATTCTTA
R-O_cuniculus : CTACCAACACCTATTCTGATTTTTCGGGCACCCCGAAGTATATATTCTTATTCTTCCAGGATTTGGAATAATTTCGCACATTGTGACATACTATTCCGGGAAAAAAGAGCCATTTGGCTATATAGGAATAGTATGAGCTATAATATCAATTGGTTTCCTT
A-O_cuniculus : CTACCAACACCTATTCTGATTTTTCGGGCACCCCGAAGTATATATTCTTATTCTTCCAGGATTTGGAATAATTTCGCACATTGTGACATACTATTCCGGGAAAAAAGAGCCATTTGGCTATATAGGAATAGTATGAGCTATAATATCAATTGGTTTCCTT
R-M_musculus : CTACCAGCATCTGTTCTGATTCTTTGGGCACCCAGAAGTTTATATTCTTATCCTCCCAGGATTTGGAATTATTTCACATGTAGTTACTTACTACTCCGGAAAAAAAGAACCTTTCGGCTATATAGGAATAGTATGAGCAATAATGTCTATTGGCTTTCTA
A-M_musculus : CTACCAGCATCTGTTCTGATTCTTTGGGCACCCAGAAGTTTATATTCTTATCCTCCCAGGATTTGGAATTATTTCACATGTAGTTACTTACTACTCCGGAAAAAAAGAACCTTTCGGCTATATAGGAATAGTATGAGCAATAATGTCTATTGGCTTTCTA
R-R_norvegicus : TTATCAACACCTATTCTGATTCTTCGGCCACCCAGAAGTGTACATCTTAATTCTTCCAGGGTTTGGAATTATTTCACATGTAGTTACCTATTACTCTGGAAAAAAAGAACCCTTCGGATATATAGGTATGGTATGAGCCATAATATCTATTGGCTTCCTA
A-R_norvegicus : CTATCAACACCTATTCTGATTCTTCGGCCACCCAGAAGTGTACATCTTAATTCTTCCAGGGTTTGGAATTATTTCACATGTAGTTACCTATTACTCTGGAAAAAAAGAACCCTTCGGATATATAGGTATGGTATGAGCCATAATATCTATTGGCTTCCTA
R-M_coypus : ATATCAACACTTGTTTTGATTTTTTGGTCATCCTGAAGTATACATTCTAATCCTTCCGGGCTTTGGAATAATTTCTCACATTGTCACATACTATTCAGGAAAAAAAGAGCCTTTTGGTTATATAGGCATAGTTTGAGCTATAATATCAATTGGTTTTTTA
A-M_coypus : ATATCAACACTTGTTTTGATTTTTTGGTCATCCTGAAGTATACATTCTAATCCTTCCGGGCTTTGGAATAATTTCTCACATTGTCACATACTATTCAGGAAAAAAAGAGCCTTTTGGTTATATAGGCATAGTTTGAGCTATAATATCAATTGGTTTTTTA
R-A_platyrhynchos : GTACCAACACCTATTTTGATTCTTCGGCCACCCAGAAGTCTATATCTTAATCCTCCCAGGATTCGGAATTATCTCACACGTAGTCACATACTACTCGGGCAAAAAGGAACCCTTCGGCTACATAGGAATAGTCTGAGCCATGCTATCCATCGGCTTCCTA
A-A_platyrhynchos : GTACCAACACCTATTTTGATTCTTCGGCCACCCAGAAGTCTATATCTTAATCCTCCCAGGATTCGGAATTATCTCACACGTAGTCACATACTACTCGGGCAAAAAGGAACCCTTCGGCTACATAGGAATAGTCTGAGCCATGCTATCCATCGGCTTCCTA
R-G_gallus : ATACCAACACCTATTCTGATTCTTCGGTCACCCCGAAGTTTACATCCTCATCCTCCCAGGTTTCGGAATAATTTCCCACGTAGTAGCATACTATGCAGGAAAAAAAGAACCATTCGGATACATAGGAATAGTCTGAGCCATACTGTCAATCGGATTCCTT
A-G_gallus : ATACCAACACCTATTCTGATTCTTCGGTCACCCCGAAGTTTACATCCTCATCCTCCCAGGTTTCGGAATAATTTCCCACGTAGTAGCATACTATGCAGGAAAAAAAGAACCATTCGGATACATAGGAATAGTCTGAGCCATACTGTCAATCGGATTCCTT
 ATATCAACACCTATTCTGATTCTTCGGACACCCAGAAGTTTATATTCTAATCCTACCAGGGTTTGGAATAATTTCMCACATTGTCACATACTACTCAGGAAAAAAAGAACCATTTGGTTATATAGGAATAGTATGAGCHATAATATCWATTGGCTTCCTA

 * 7860 * 7880 * 7900 * 7920 * 7940 * 7960 * 7980 * 8000
R-E_caballus : GGCTTCATCGTATGGGCTCACCACATGTTTACAGTAGGGATAGACGTTGACACACGAGCATACTTCACATCAGCTACCATAATCATCGCTATCCCTACTGGTGTAAAAGTATTCAGCTGACTAGCCACCCTGCACGGAGGAAATATCAAATGATCTCCAG
A-E_caballus : GGCTTCATCGTATGGGCTCACCACATGTTTACAGTAGGGATAGACGTTGACACACGAGCATACTTCACATCAGCTACCATAATCATCGCTATCCCTACCGGTGTAAAAGTATTCAGCTGACTAGCCACCCTGCACGGAGGAAATATCAAATGATCTCCAG
R-C_bactrianus : GGCTTCATTGTATGAGCCCACCATATATTTACAGTAGGCATGGACGTAGACACACGTGCTTATTTCACATCTGCCACAATAATTATTGCTATTCCAACGGGAGTAAAAGTATTTAGCTGATTGGCAACACTCCATGGAGGCAACATCAAATGATCCCCTG
A-C_bactrianus : GGCTTCATTGTATGAGCCCACCATATATTTACAGTAGGCATGGACGTAGACACACGTGCTTATTTCACATCTGCCACAATAATTATTGCTATTCCAACGGGAGTAAAAGTATTTAGCTGATTGGCAACACTCCATGGAGGCAACATCAAATGATCCCCTG
R-C_lupus_familiaris : GGCTTTATCGTATGAGCTCACCATATGTTTACCGTAGGAATAGATGTAGACACACGAGCGTACTTTACGTCCGCCACTATAATTATCGCTATTCCAACGGGAGTAAAAGTATTTAGTTGACTGGCAACACTTCATGGAGGCAATATTAAATGATCTCCAG
A-C_lupus_familiaris : GGCTTTATCGTATGAGCTCACCATATGTTTACCGTAGGAATAGATGTAGACACACGAGCGTACTTTACGTCCGCCACTATAATTATCGCTATTCCAACGGGAGTAAAAGTATTTAGTTGACTGGCAACACTTCATGGAGGCAATATTAAATGATCTCCAG
R-N_procyonoides : GGCTTTATCGTGTGAGCCCACCACATATTTACTGTAGGAATGGACGTAGATACACGAGCATACTTCACATCCGCTACTATAATTATTGCCATCCCGACTGGGGTTAAGGTATTTAGCTGACTAGCGACGCTTCACGGAGGTAATATTAAATGATCCCCAG
A-N_procyonoides : GGCTTTATCGTGTGAGCCCACCACATATTTACTGTAGGAATGGACGTAGATACACGAGCATACTTCACATCCGCTACTATAATTATTGCCATCCCGACTGGGGTTAAGGTATTTAGCTGACTAGCGACGCTTCACGGAGGTAATATTAAATGATCCCCAG
R-V_lagopus : GGTTTTATCGTGTGAGCTCATCACATATTCACTGTAGGAATAGACGTGGATACACGAGCATACTTCACGTCCGCCACTATAATTATTGCCATTCCTACAGGGGTTAAAGTATTCAGCTGATTAGCAACACTTCACGGGGGAAATATTAAATGATCTCCAG
A-V_lagopus : GGTTTTATCGTGTGAGCTCATCACATATTCACTGTAGGAATAGACGTGGATACACGAGCATACTTCACGTCCGCCACTATAATTATTGCCATTCCTACAGGGGTTAAAGTATTCAGCTGATTAGCAACACTTCACGGGGGAAATATTAAATGATCTCCAG
R-M_putorius : GGATTTATCGTATGAGCCCACCATATATTTACTGTGGGCCTGGACGTCGACACACGAGCATATTTCACTTCAGCTACTATAATCATCGCTATCCCCACAGGAGTAAAAGTATTCAGCTGACTAGCCACTCTGCATGGAGGAAATATCAAATGAGCTCCTG
A-M_putorius : GGATTTATCGTATGAGCCCACCATATATTTACTGTGGGCCTGGACGTCGACACACGAGCATATTTCACTTCAGCTACTATAATCATCGCTATCCCCACAGGAGTAAAAGTATTCAGCTGACTAGCCACTCTGCATGGAGGAAATATCAAATGAGCTCCTG
R-B_taurus : GGTTTCATCGTATGAGCCCACCATATATTCACTGTCGGAATAGACGTCGACACACGAGCCTACTTCACATCAGCCACTATAATTATTGCTATTCCAACCGGGGTAAAAGTCTTCAGCTGATTGGCAACACTTCATGGAGGTAATATCAAATGGTCTCCTG
A-B_taurus : GGTTTCATCGTATGAGCCCACCATATATTCACTGTCGGAATAGACGTCGACACACGAGCCTACTTCACATCAGCCACTATAATTATTGCTATTCCAACCGGGGTAAAAGTCTTCAGCTGATTGGCAACACTTCATGGAGGTAATATCAAATGGTCTCCTG
R-O_aries : GGATTCATTGTATGAGCCCACCATATATTCACAGTCGGAATAGACGTCGATACACGGGCTTACTTCACGTCAGCTACTATAATTATCGCCATCCCAACAGGAGTAAAAGTATTCAGTTGACTAGCAACGCTTCATGGGGGTAATATCAAATGATCTCCTG
A-O_aries : GGATTCATTGTATGAGCCCACCATATATTCACAGTCGGAATAGACGTCGATACACGGGCTTACTTCACGTCAGCTACTATAATTATTGCCATCCCAACAGGAGTAAAAGTATTCAGTTGACTAGCAACGCTTCATGGGGGTAATATCAAATGATCTCCTG
R-S_scrofa : GGTTTTATCGTATGGGCTCACCACATATTCACCGTAGGAATAGACGTGGATACCCGAGCATACTTTACATCTGCCACAATAATCATTGCTATTCCCACTGGAGTAAAAGTATTTAGTTGATTAGCTACCCTGCACGGCGGCAATATTAAATGATCACCCG
A-S_scrofa : GGTTTTATCGTATGGGCTCACCACATATTCACCGTAGGAATAGACGTGGATACCCGAGCATACTTTACATCTGCCACAATAATCATTGCTATTCCCACTGGAGTAAAAGTATTTAGTTGATTAGCTACCCTGCACGGCGGCAATATTAAATGATCACCCG
R-O_cuniculus : GGATTTATCGTATGGGCCCATCATATATTTACAGTAGGAATAGATGTAGACACACGAGCCTACTTTACATCAGCTACCATAATCATCGCTATCCCTACTGGAGTCAAAGTATTTAGCTGGCTAGCAACACTGCATGGCGGCAACATCAAATGATCCCCCG
A-O_cuniculus : GGATTTATCGTATGGGCCCATCATATATTTACAGTAGGAATAGATGTAGACACACGAGCCTACTTTACATCAGCTACCATAATCATCGCTATCCCTACTGGAGTCAAAGTATTTAGCTGGCTAGCAACACTGCATGGCGGCAACATCAAATGATCCCCCG
R-M_musculus : GGCTTTATTGTATGAGCCCACCACATATTCACAGTAGGATTAGATGTAGACACACGAGCTTACTTTACATCAGCCACTATAATTATCGCAATTCCTACCGGTGTCAAAGTATTTAGCTGACTTGCAACCCTACACGGAGGTAATATTAAATGATCTCCAG
A-M_musculus : GGCTTTATTGTATGAGCCCACCACATATTCACAGTAGGATTAGATGTAGACACACGAGCTTACTTTACATCAGCCACTATAATTATCGCAATTCCTACCGGTGTCAAAGTATTTAGCTGACTTGCAACCCTACACGGAGGTAATATTAAATGATCTCCAG
R-R_norvegicus : GGATTTATTGTATGAGCACATCACATATTCACAGTAGGCCTAGATGTAGACACCCGAGCCTACTTTACATCTGCCACTATAATTATCGCAATTCCTACAGGCGTAAAAGTATTCAGCTGACTCGCTACACTACATGGAGGAAATATCAAATGATCCCCCG
A-R_norvegicus : GGGTTTATTGTATGAGCACATCACATATTCACAGTAGGCCTAGATGTAGACACCCGAGCCTACTTTACATCTGCCACTATAATTATCGCAATTCCTACAGGCGTAAAAGTATTCAGCTGACTCGCTACACTACATGGAGGGAATATCAAATGATCCCCCG
R-M_coypus : GGTTTTATTGTATGAGCTCACCATATATTTACAGTTGGAATAGACGTTGACACTCGAGCATATTTTACATCAGCCACAATAATTATTGCAATTCCTACAGGTGTAAAAGTATTTAGCTGATTAGCAACACTGCACGGCGGAAATATTAAATGGTCCCCCG
A-M_coypus : GGTTTTATTGTATGAGCTCATCATATATTTACAGTTGGAATAGACGTTGACACTCGAGCATATTTTACATCAGCCACAATAATTATTGCAATTCCTACAGGTGTAAAAGTATTTAGCTGATTAGCAACACTGCACGGCGGAAATATTAAATGGTCCCCCG
R-A_platyrhynchos : GGATTTATCGTCTGAGCCCACCACATATTCACCGTAGGAATAGACGTTGACACCCGGGCCTACTTCACATCCGCCACTATAATCATCGCCATCCCTACCGGAATCAAAGTCTTTAGCTGACTCGCCACCCTACACGGGGGAACAATCAAATGAGATCCCC
A-A_platyrhynchos : GGATTTATCGTCTGAGCCCACCACATATTCACCGTAGGAATAGACGTTGACACCCGGGCCTACTTCACATCCGCCACTATAATCATCGCCATCCCTACCGGAATCAAAGTCTTTAGCTGACTCGCCACCCTACACGGGGGAACAATCAAATGAGATCCCC
R-G_gallus : GGCTTCATTGTATGAGCCCACCATATATTCACAGTCCGAATGGACGTAGACACCCGAGCCTACTTTACATCAGCCACAATAATCATCGCCATCCCAACTGGTATTAAAGTCTTCAGCTGACTAGCAACCCTGCACGGAGGAACAATTAAATGAGACCCCC
A-G_gallus : GGCTTCATTGTATGAGCCCACCATATATTCACAGTCGGAATGGACGTAGACACCCGAGCCTACTTTACATCAGCCACAATAATCATCGCCATCCCAACTGGTATTAAAGTCTTCAGCTGACTAGCAACCCTGCACGGAGGAACAATTAAATGAGACCCCC
 GGCTTTATCGTATGAGCCCACCATATATTCACAGTAGGAATAGACGTAGACACACGAGCATACTTCACATCAGCCACTATAATTATCGCTATTCCTACAGGAGTAAAAGTATTTAGCTGACTAGCAACACTGCACGGAGGAAATATCAAATGATCTCCCG

 * 8020 * 8040 * 8060 * 8080 * 8100 * 8120 * 8140 * 8160
R-E_caballus : CTATACTCTGAGCTCTAGGCTTCATCTTCTTATTCACAGTAGGAGGTCTAACAGGAATCGTCCTAGCTAACTCATCCCTAGATATTGTTCTCCACGATACTTATTATGTAGTAGCACATTTCCATTATGTCCTGTCTATAGGAGCAGTCTTCGCCATTAT
A-E_caballus : CTATACTCTGAGCTCTAGGCTTCATCTTCTTATTCACAGTAGGAGGTCTAACAGGAATCGTCCTAGCTAACTCATCCCTAGATATTGTTCTCCACGATACTTATTATGTAGTAGCACATTTCCATTATGTCCTGTCTATAGGAGCAGTCTTCGCCATTAT
R-C_bactrianus : CCATACTGTGAGCCCTTGGCTTTATTTTCTTATTCACCGTAGGAGGTCTAACAGGAATTGTTCTAGCTAATTCATCACTAGACATTGTCCTTCACGATACTTATTATGTAGTTGCCCACTTCCACTATGTCTTGTCAATAGGGGCAGTCTTCGCTATCAT
A-C_bactrianus : CCATACTGTGAGCCCTTGGCTTTATTTTCTTATTCACCGTAGGAGGTCTAACAGGAATTGTTCTAGCTAATTCATCACTAGACATTGTCCTTCACGATACTTATTATGTAGTTGCCCACTTCCACTATGTCTTGTCAATAGGGGCAGTCTTCGCTATCAT
R-C_lupus_familiaris : CTATGCTATGAGCTTTAGGGTTTATTTTCTTATTTACAGTAGGCGGGTTAACAGGTATTGTCCTAGCTAATTCGTCCTTAGACATCGTTCTTCATGATACATATTATGTTGTGGCTCATTTTCACTATGTGCTTTCAATAGGAGCAGTTTTTGCCATTAT
A-C_lupus_familiaris : CTATGCTATGAGCTTTAGGGTTTATTTTCTTATTTACAGTAGGCGGGTTAACAGGTATTGTCCTAGCTAATTCGTCCTTAGACATCGTTCTTCATGATACATATTATGTTGTGGCTCATTTTCACTATGTGCTTTCAATAGGAGCAGTTTTTGCCATTAT
R-N_procyonoides : CCATACTATGAGCCCTAGGGTTTATCTTTCTTTTCACAGTAGGCGGACTGACAGGCATTGTCCTGGCTAACTCGTCCTTAGATATTGTCCTTCACGATACATACTATGTTGTAGCCCACTTCCATTATGTGCTTTCAATAGGAGCAGTATTTGCCATTAT
A-N_procyonoides : CCATACTATGAGCCCTAGGGTTTATCTTTCTTTTCACAGTAGGCGGACTGACAGGCATTGTCCTGGCTAACTCGTCCTTAGATATTGTCCTTCACGATACATACTATGTTGTAGCCCACTTCCATTATGTGCTTTCAATAGGAGCAGTATTTGCCATTAT
R-V_lagopus : CTATGCTGTGAGCTCTAGGCTTTATTTTCCTATTCACGGTAGGTGGCCTAACGGGCATCGTTTTAGCCAACTCGTCCCTGGACATCGTTCTCCATGACACGTACTACGTTGTAGCCCACTTCCACTACGTACTCTCAATGGGAGCAGTATTTGCTATTAT
A-V_lagopus : CTATGCTGTGAGCTCTAGGCTTTATTTTCCTATTCACGGTAGGTGGCCTAACGGGCATCGTTTTAGCCAACTCGTCCCTGGACATCGTTCTCCATGACACGTACTACGTTGTAGCCCACTTCCACTACGTACTCTCAATGGGAGCAGTATTTGCTATTAT
R-M_putorius : CTATACTATGAGCCTTAGGGTTTATTTTTCTATTTACAGTGGGGGGTCTAACGGGCATTGTACTATCTAACTCATCACTAGACATTGTCCTTCACGATACGTATTATGTAGTAGCACATTTCCACTACGTCCTTTCAATAGGGGCAGTATTTGCAATTAT
A-M_putorius : CTATACTATGAGCCTTAGGGTTTATTTTTCTATTTACAGTGGGGGGTCTAACGGGCATTGTACTATCTAACTCATCACTAGACATTGTCCTTCACGATACGTATTATGTAGTAGCACATTTCCACTACGTCCTTTCAATAGGGGCAGTATTTGCAATTAT
R-B_taurus : CTATAATGTGAGCCCTAGGCTTTATTTTCTTATTTACAGTAGGGGGTTTAACTGGAATTGTCTTAGCCAACTCTTCCCTCGATATTGTTCTTCACGACACATACTACGTTGTCGCACATTTCCACTATGTTTTATCAATAGGAGCTGTATTTGCTATTAT
A-B_taurus : CTATAATGTGAGCCCTAGGCTTTATTTTCTTATTTACAGTAGGGGGTTTAACTGGAATTGTCTTAGCCAACTCTTCCCTCGATATTGTTCTTCACGACACATACTACGTTGTCGCACATTTCCACTATGTTTTATCAATAGGAGCTGTATTTGCTATTAT
R-O_aries : CCATAATATGAGCCCTAGGTTTCATCTTTCTTTTCACAGTCGGAGGCTTAACTGGAATTGTTCTAGCCAACTCCTCCCTTGACATTGTCCTCCATGACACATATTATGTAGTAGCACATTTCCACTACGTATTATCAATAGGAGCTGTATTTGCTATTAT
A-O_aries : CCATAATATGAGCCCTAGGTTTCATCTTTCTTTTCACAGTCGGAGGCTTAACTGGAATTGTTCTAGCCAACTCCTCCCTTGACATTGTCCTCCATGACACATATTATGTAGTAGCACATTTCCACTACGTATTATCAATAGGAGCTGTATTTGCTATTAT
R-S_scrofa : CAATACTATGAGCTCTGGGCTTCATCTTCCTATTCACCGTAGGAGGTCTAACGGGCATTGTACTAGCTAACTCCTCCCTAGACATTGTATTACATGATACATATTATGTAGTCGCACACTTCCACTATGTCTTATCTATAGGAGCAGTGTTTGCCATTAT
A-S_scrofa : CAATACTATGAGCTCTGGGCTTCATCTTCCTATTCACCGTAGGAGGTCTAACGGGCATTGTACTAGCTAACTCCTCCCTAGACATTGTATTACATGATACATATTATGTAGTCGCACACTTCCACTATGTCTTATCTATAGGAGCAGTGTTTGCCATTAT
R-O_cuniculus : CTATGCTCTGAGCTCTAGGCTTTATTTTCCTATTTACAGTAGGCGGTCTTACAGGAATTGTGCTAGCCAACTCCTCTTTAGACATCGTACTACATGACACCTACTATGTAGTAGCTCACTTCCACTATGTCCTATCTATGGGGGCTGTATTTGCTATTAT
A-O_cuniculus : CTATGCTCTGAGCTCTAGGCTTTATTTTCCTATTTACAGTAGGCGGTCTTACAGGAATTGTGCTAGCCAACTCCTCTTTAGACATCGTACTACATGACACCTACTATGTAGTAGCTCACTTCCACTATGTCCTATCTATGGGGGCTGTATTTGCTATTAT
R-M_musculus : CTATACTATGAGCCTTAGGCTTTATTTTCTTATTTACAGTTGGTGGTCTAACCGGAATTGTTTTATCCAACTCATCCCTTGACATCGTGCTTCACGATACATACTATGTAGTAGCCCATTTCCACTATGTTCTATCAATGGGAGCAGTGTTTGCTATCAT
A-M_musculus : CTATACTATGAGCCTTAGGCTTTATTTTCTTATTTACAGTTGGTGGTCTAACCGGAATTGTTTTATCCAACTCATCCCTTGACATCGTGCTTCACGATACATACTATGTAGTAGCCCATTTCCACTATGTTCTATCAATGGGAGCAGTGTTTGCTATCAT
R-R_norvegicus : CCATATTATGAGCCTTAGGGTTTATCTTCTTATTCACAGTAGGGGGCCTAACAGGGATCGTACTATCTAACTCATCCCTTGACATTGTACTTCATGATACATACTATGTAGTAGCTCACTTCCACTATGTCTTATCTATAGGAGCAGTATTCGCCATCAT
A-R_norvegicus : CCATATTATGAGCCTTAGGGTTTATCTTCTTATTCACAGTAGGGGGCCTAACAGGGATCGTACTATCTAACTCATCCCTTGACATTGTACTTCATGATACATACTATGTAGTAGCTCACTTCCACTATGTCTTATCTATAGGAGCAGTATTCGCCATCAT
R-M_coypus : CTATACTATGAGCACTTGGCTTTATCTTCTTATTTACTGTGGGTGGCTTAACAGGCATTGTGCTAGCTAACTCATCTTTAGATATTGTTTTACACGATACATATTATGTTGTCGCACATTTCCACTATGTTTTATCAATAGGAGCAGTATTTGCTATTAT
A-M_coypus : CTATACTATGAGCACTTGGCTTTATCTTCTTATTTACTGTGGGTGGCTTAACAGGCATTGTGCTAGCTAACTCATCTTTAGATATTGTTTTACACGATACATATTATGTTGTCGCACATTTCCACTATGTTTTATCAATAGGAGCAGTATTTGCTATTAT
R-A_platyrhynchos : CAATACTCTGAGCTCTAGGGTTTATCTTCCTATTTACCATCGGAGGACTAACAGGGATCGTCCTTGCGAACTCCTCCCTAGATATCGCCCTGCATGACACGTACTACGTAGTCGCCCACTTCCACTACGTACTATCTATGGGCGCTGTCTTTGCCATCCT
A-A_platyrhynchos : CAATACTCTGAGCTCTAGGGTTTATCTTCCTATTTACCATCGGAGGACTAACAGGGATCGTCCTTGCGAACTCCTCCCTAGATATCGCCCTGCATGACACGTACTACGTAGTCGCCCACTTCCACTACGTACTATCTATGGGCGCTGTCTTTGCCATCCT
R-G_gallus : CTATGCTATGAGCCCTAGGATTCATCTTCCTCTTCACTATCGGAGGCCTAACGGGAATCGTCCTTGCTAACTCATCACTAGATATTGCCCTTCATGACACCTACTATGTAGTCGCCCACTTCCACTATGTCCTCTCAATGGGGGCAGTTTTTGCCATTCT
A-G_gallus : CTATGCTATGAGCCCTAGGATTCATCTTCCTCTTCACTATCGGAGGCCTAACGGGAATCGTCCTTGCTAACTCATCACTAGATATTGCCCTTCATGACACCTACTATGTAGTCGCCCACTTCCACTATGTCCTCTCAATGGGGGCAGTTTTTGCCATTCT
 CTATACTATGAGCCCTAGGCTTTATCTTCCTATTCACAGTAGGAGGTCTAACAGGAATTGTCCTAGCTAACTCATCCCTAGACATTGTCCTTCATGATACATACTATGTAGTAGCMCACTTCCACTATGTCCTATCAATAGGAGCAGTATTTGCYATTAT

 * 8180 * 8200 * 8220 * 8240 * 8260 * 8280 * 8300 * 8320
R-E_caballus : GGGGGGATTTGTACACTGATTCCCTCTATTCTCAGGATACACACTCAACCAAACCTGAGCAAAAATCCACTTTACAATTATATTCGTAGGGGTAAATATAACCTTCTTCCCACAACATTTCCTTGGCCTCTCAGGAATGCCACGACGCTATTCTGATTAT
A-E_caballus : GGGGGGATTTGTACACTGATTCCCTCTATTCTCAGGATACACACTCAACCAAACCTGAGCAAAAATCCACTTTACAATTATATTCGTAGGGGTAAATATAACCTTCTTCCCACAACATTTCCTTGGCCTCTCAGGAATGCCACGACGCTATTCTGATTAT
R-C_bactrianus : AGGGGGCTTTATGCATTGATTCCCTCTGTTCTCCGGATACACGATTGACGATACATGAGCAAAAATTCAATTCGCAATTATATTTGTAGGGGTAAACCTAACTTTCTTCCCGCAACATTTTCTAGGTCTCTCCGGAATGCCTCGACGCTACTCTGACTAT
A-C_bactrianus : AGGGGGCTTTATGCATTGATTCCCTCTGTTCTCCGGATACACGATTGACGATACATGAGCAAAAATTCAATTCGCAATTATATTTGTAGGGGTAAACCTAACTTTCTTCCCGCAACATTTTCTAGGTCTCTCCGGAATGCCTCGACGCTACTCTGACTAT
R-C_lupus_familiaris : GGGAGGATTTGCCCACTGATTCCCTTTATTCTCAGGTTATACTCTTAACGATACTTGAGCAAAGATTCACTTTACAATTATGTTTGTGGGAGTAAATATAACTTTCTTCCCTCAACATTTCCTAGGTTTATCTGGAATACCTCGTCGATACTCTGACTAC
A-C_lupus_familiaris : GGGAGGATTTGCCCACTGATTCCCTTTATTCTCAGGTTATACTCTTAACGATACTTGAGCAAAGATTCACTTTACAATTATGTTTGTGGGAGTAAATATAACTTTCTTCCCTCAACATTTCCTAGGTTTATCTGGAATACCTCGTCGATACTCTGACTAC
R-N_procyonoides : AGGCGGATTTGCTCACTGATTCCCCCTATTCTCAGGCTATACTCTCAATGATACTTGAGCAAAAATTCACTTTACAATTATATTTGTAGGGGTTAATATGACTTTCTTCCCTCAACACTTTCTAGGGCTATCAGGAATACCTCGTCGGTATTCAGACTAC
A-N_procyonoides : AGGCGGATTTGCTCACTGATTCCCCCTATTCTCAGGCTATACTCTCAATGATACTTGAGCAAAAATTCACTTTACAATTATATTTGTAGGGGTTAATATGACTTTCTTCCCTCAACACTTTCTAGGGCTATCAGGAATACCTCGTCGGTATTCAGACTAC
R-V_lagopus : AGGCGGATTTGCCCACTGATTCCCCTTATTCTCGGGCTACACCCTAAATGACACCTGAGCAAAAATCCATTTTACAATCATATTCGTAGGGGTGAACATGACCTTCTTCCCTCAACACTTCCTGGGACTATCGGGCATACCCCGTCGATACTCCGACTAC
A-V_lagopus : AGGCGGATTTGCCCACTGATTCCCCTTATTCTCGGGCTACACCCTAAATGACACCTGAGCAAAAATCCATTTTACAATCATATTCGTAGGGGTGAACATGACCTTCTTCCCTCAACACTTCCTGGGACTATCGGGCATACCCCGTCGATACTCCGACTAC
R-M_putorius : AGGTGGATTCGTCCACTGATTCCCACTATTCACAGGCTACACCCTAAATGATGTATGAGCAAAAATTCATTTCACGATCATATTTGTAGGAGTAAACATAACATTCTTTCCTCAACATTTCCTGGGCCTATCAGGCATACCTCGACGCTACTCTGATTAT
A-M_putorius : AGGTGGATTCGTCCACTGATTCCCACTATTCACAGGCTACACCCTAAATGATGTATGAGCAAAAATTCATTTCACGATCATATTTGTAGGAGTAAACATAACATTCTTTCCTCAACATTTCCTGGGCCTATCAGGCATACCTCGACGCTACTCTGATTAT
R-B_taurus : AGGGGGATTTGTTCATTGATTCCCACTATTCTCAGGTTATACTCTCAACGATACATGAGCCAAAATCCACTTCGCAATTATATTTGTAGGCGTCAATATAACCTTCTTCCCACAACACTTTCTAGGACTATCTGGCATGCCTCGACGATACTCCGACTAC
A-B_taurus : AGGGGGATTTGTTCATTGATTCCCACTATTCTCAGGTTATACTCTCAACGATACATGAGCCAAAATCCACTTCGCAATTATATTTGTAGGCGTCAATATAACCTTCTTCCCACAACACTTTCTAGGACTATCTGGCATGCCTCGACGATACTCCGACTAC
R-O_aries : AGGAGGATTTGTACATTGATTTCCCCTATTCTCAGGCTATACTCTCAATGATACATGAGCCAAAATCCACTTTGCAATTATATTTGTAGGTGTTAACATGACTTTCTTTCCACAGCATTTCCTAGGACTATCCGGTATACCACGACGATACTCTGATTAT
A-O_aries : AGGAGGATTCGTACATTGATTTCCCCTATTCTCAGGCTATACTCTCAATGATACGTGAGCCAAAATCCACTTTGCAATTATATTTGTAGGTGTTAACATGACTTTCTTTCCACAACATTTCCTAGGATTATCCGGTATACCACGACGATACTCTGATTAT
R-S_scrofa : AGGGGGCTTTGTTCACTGATTCCCCCTATTCTCCGGGTACACACTCAACCAAGCATGAGCAAAAATTCACTTTGTAATTATATTCGTAGGAGTAAATATAACATTCTTTCCACAACACTTTCTAGGACTATCCGGAATACCTCGACGATACTCCGATTAT
A-S_scrofa : AGGGGGCTTTGTTCACTGATTCCCCCTATTCTCCGGGTACACACTCAACCAAGCATGAGCAAAAATTCACTTTGTAATTATATTCGTAGGAGTAAATATAACATTCTTTCCACAACACTTTCTAGGACTATCCGGAATACCTCGACGATACTCCGATTAT
R-O_cuniculus : AGGAGGATTTGCCCATTGATTCCCCCTATTCTCAGGCTATACCCTTGACCCAACCTGAGCTAAAATTCACTTCACTGTGATATTTGTAGGAGTCAACTTAACTTTCTTCCCTCAACATTTCCTTGGCCTCTCAGGTATACCCCGACGGTACTCAGACTAC
A-O_cuniculus : AGGAGGATTTGCCCATTGATTCCCCCTATTCTCAGGCTATACCCTTGACCCAACCTGAGCTAAAATTCACTTCACTGTGATATTTGTAGGAGTCAACTTAACCTTCTTCCCTCAACATTTCCTTGGCCTCTCAGGTATACCCCGACGGTACTCAGACTAC
R-M_musculus : AGCAGGATTTGTTCACTGATTCCCATTATTTTCAGGCTTCACCCTAGATGACACATGAGCAAAAGCCCACTTCGCCATCATATTCGTAGGAGTAAACATAACATTCTTCCCTCAACATTTCCTGGGCCTTTCAGGAATACCACGACGCTACTCAGACTAC
A-M_musculus : AGCAGGATTTGTTCACTGATTCCCATTATTTTCAGGCTTCACCCTAGATGACACATGAGCAAAAGCCCACTTCGCCATCATATTCGTAGGAGTAAACATAACATTCTTCCCTCAACATTTCCTGGGCCTTTCAGGAATACCACGACGCTACTCAGACTAC
R-R_norvegicus : AGCTGGCTTCGTCCACTGATTCCCACTATTCTCAGGCTATACCCTAAATGACACATGAGCAAAAGCCCACTTTGCCATTATATTTGTAGGTGTAAACATAACATTCTTTCCTCAACACTTCCTAGGATTAGCAGGGATACCTCGTCGTTACTCTGATTAT
A-R_norvegicus : AGCTGGCTTCGTCCACTGATTCCCACTATTCTCAGGCTATACCCTAAATGACACATGAGCAAAAGCCCACTTTGCCATTATATTTGTAGGTGTAAACATAACATTTTTCCCTCAACACTTCCTAGGATTAGCGGGGATACCTCGTCGTTACTCTGATTAT
R-M_coypus : AGGCGGGTTTGTTCACTGATTCCCCTTATTTTCAGGATATACCTTAAATACAATATGAGCTAAAGTCCATTTTTTCATTATATTTACAGGAGTAAATATTACTTTCTTCCCTCAACACTTCCTGGGATTATCAGGTATACCTCGACGATACTCAGATTAT
A-M_coypus : AGGCGGGTTTGTTCACTGATTCCCCTTATTTTCAGGATATACCTTAAATACAATATGAGCTAAAGTCCATTTTTTCATTATATTTACAGGAGTAAATATTACTTTCTTCCCTCAACACTTCCTGGGATTATCAGGTATACCTCGACGATACTCAGATTAT
R-A_platyrhynchos : AGCTGGATTCACTCACTGATTCCCCCTTCTTACAGGATTCACTCTACACCAAACATGAGCAAAAGCCCACTTCGGAGTGATATTTACAGGGGTAAACCTAACATTCTTCCCCCAACACTTCCTAGGCCTGGCAGGAATGCCCCGACGATACTCGGACTAC
A-A_platyrhynchos : AGCTGGATTCACTCACTGATTCCCCCTTCTTACAGGATTCACTCTACACCAAACATGAGCAAAAGCCCACTTCGGAGTGATATTTACAGGGGTAAACCTAACATTCTTCCCCCAACACTTCCTAGGCCTGGCAGGAATGCCCCGACGATACTCGGACTAC
R-G_gallus : AGCAGGATTTACCCACTGATTTCCCCTCTTCACAGGCTTTACCCTACACCCATCATGAACCAAGGCACATTTCGGAGTAATATTTACCGGAGTTAACCTAACCTTTTTCCCCCAACATTTCCTGGGCCTAGCTGGAATACCCCGACGATACTCAGATTAC
A-G_gallus : AGCAGGATTTACCCACTGATTTCCCCTCTTCACAGGCTTTACCCTACACCCATCATGAACCAAGGCACATTTCGGAGTAATATTTACCGGAGTTAACCTAACCTTTTTCCCCCAACATTTCCTGGGCCTAGCTGGAATACCCCGACGATACTCAGATTAC
 AGGAGGATTTGTYCACTGATTCCCCCTATTCTCAGGCTATACCCTAAACGAWACATGAGCAAAAATCCACTTTGCAATTATATTTGTAGGAGTAAACATAACTTTCTTCCCTCAACATTTCCTAGGMCTATCAGGAATACCTCGACGATACTCTGACTAC

 * 8340 * 8360 * 8380 * 8400 * 8420 * 8440 * 8460 * 8480
R-E_caballus : CCAGACGCATATACAACATGAAATACCATCTCATCCATAGGATCTTTTATCTCACTTACAGCAGTGATACTAATAATTTTCATAATTTGAGAAGCGTTCGCATCCAAACGAGAAGTGTCTACAGTAGAATTAACCTCAACTAATCTGGAATGACTACACG
A-E_caballus : CCAGACGCATATACAACATGAAATACCATCTCATCCATAGGATCTTTTATCTCACTTACAGCAGTGATACTAATAATTTTCATAATTTGAGAAGCATTCGCATCCAAACGAGAAGTGTCTACAGTAGAATTAACCTCAACTAATCTGGAATGACTACACG
R-C_bactrianus : CCCGATGCCTATACTACATGAAACACTATTTCATCCGTGGGCTCTTTTATTTCCTTAACGGCAGTCGTGCTGATAGTGTTTATTGTGTGAGAAGCCTTCGCGTCAAAACGGGAGGTCACAACCGTAGAGCTAACGGCCACCAATCTAGAGTGACTGCACG
A-C_bactrianus : CCCGATGCCTATACTACATGAAACACTATTTCATCCGTGGGCTCTTTTATTTCCTTAACGGCAGTCGTGCTGATAGTGTTTATTGTGTGAGAAGCCTTCGCGTCAAAACGGGAGGTCACAACCGTAGAGCTAACGGCCACCAATCTAGAGTGACTGCACG
R-C_lupus_familiaris : CCAGATGCATATACTACCTGAAATACCGTCTCCTCTATAGGATCGTTTATCTCGCTTACAGCGGTGATGCTTATAATTTTTATGATCTGGGAAGCCTTTGCATCCAAACGAGAAGTTGCTATAGTAGAACTTACTACAACTAACATTGAGTGACTACATG
A-C_lupus_familiaris : CCAGATGCATATACTACCTGAAATACCGTCTCCTCTATAGGATCGTTTATCTCGCTTACAGCGGTGATGCTTATAATTTTTATGATCTGGGAAGCCTTTGCATCCAAACGAGAAGTTGCTATAGTAGAACTTACTACAACTAACATTGAGTGACTACATG
R-N_procyonoides : CCAGATGCGTATACGACATGAAATACCGTCTCCTCTATAGGCTCATTCATCTCACTCACTGCAGTGATATTAATAATTTTCATGATCTGAGAGGCATTCGCCTCCAAACGAGAAGTTGCAACAGTGGAGCTCACCACAACCAACATCGAGTGATTACATG
A-N_procyonoides : CCAGATGCGTATACGACATGAAATACCGTCTCCTCTATAGGCTCATTCATCTCACTCACTGCAGTGATATTAATAATTTTCATGATCTGAGAGGCATTCGCCTCCAAACGAGAAGTTGCAACAGTGGAGCTCACCACAACCAACATCGAGTGATTACATG
R-V_lagopus : CCAGATGCATACACTACTTGAAACACCGTCTCATCTATAGGCTCATTCATCTCACTTACAGCAGTAATACTTATAATCTTCATAATCTGGGAAGCTTTTGCTTCTAAACGGGAGGTTGCAATAGTAGAGCTCACTACAACTAACATTGAGTGACTACACG
A-V_lagopus : CCAGATGCATACACTACTTGAAACACCGTCTCATCTATAGGCTCATTCATCTCACTTACAGCAGTAATACTTATAATCTTCATAATCTGGGAAGCTTTTGCTTCTAAACGGGAGGTTGCAATAGTAGAGCTCACTACAACTAACATTGAGTGACTACACG
R-M_putorius : CCAGATGCTTATACAACATGAAATACAGTGTCCTCCATGGGCTCATTCATCTCATTAACAGCAGTCATACTAATGATCTTCATGATTTGAGAAGCTTTCGCATCCAAACGAGAAGTATTGACAGTTGAATTAACCTCAACTAACATTGAATGATTGCACG
A-M_putorius : CCAGATGCTTATACAACATGAAATACAGTGTCCTCCATGGGCTCATTCATCTCATTAACGGCAGTCATACTAATGATCTTCATGATTTGAGAAGCTTTCGCATCCAAACGAGAAGTATTGACAGTTGAATTAACCTCAACTAACATTGAATGATTGCACG
R-B_taurus : CCAGATGCATACACAATATGAAATACTATCTCATCAATAGGCTCATTCATTTCCCTAACAGCAGTTATACTAATAGTTTTCATCATCTGAGAAGCATTTGCATCTAAACGAGAAGTCTTGACTGTAGACTTAACCACGACAAATCTAGAATGATTAAACG
A-B_taurus : CCAGATGCATACACAATATGAAATACTATCTCATCAATAGGCTCATTCATTTCCCTAACAGCAGTTATACTAATAGTTTTCATCATCTGAGAAGCATTTGCATCTAAACGAGAAGTCTTGACTGTAGACTTAACCACGACAAATCTAGAATGATTAAACG
R-O_aries : CCAGACGCATATACAATATGAAATACTATCTCATCTATAGGCTCATTTATCTCACTAACAGCAGTAATACTAATAATCTTCATCATCTGAGAAGCATTTGCATCTAAACGAGAAGTCCTAACTGTAGACCTAACCACAACAAACCTAGAATGACTAAACG
A-O_aries : CCAGACGCATATACAATATGAAATACTATCTCATCTATAGGCTCATTTATCTCACTAACAGCAGTGATACTAATAATCTTCATCATCTGAGAAGCATTTGCATCTAAACGAGAAGTCCTAACTGTAGACCTAACCACAACAAACCTAGAATGACTAAACG
R-S_scrofa : CCTGACGCATACACAGCATGAAATACTATTTCCTCAATAGGCTCATTCATCTCACTAACAGCAGTGATATTAATAATCTTCATTATCTGAGAAGCATTTGCATCAAAACGAGAAGTATCTGCAGTAGAACTGACAAGCACAAACCTAGAATGACTACACG
A-S_scrofa : CCTGACGCATACACAGCATGAAATACTATTTCCTCAATAGGCTCATTCATCTCACTAACAGCAGTGATATTAATAATCTTCATTATCTGAGAAGCATTTGCATCAAAACGAGAAGTATCTGCAGTAGAACTGACAAGCACAAACCTAGAATGACTACACG
R-O_cuniculus : CCGGACGCCTATACAATATGAAATACTGTTTCATCAATAGGCTCATTCATTTCCCTAACTGCCGTAATAGTAATAATTTTTATAATCTGAGAAGCCTTCGCCTCAAAACGAGAAGTAGAGACTATTGAACTAACAACCACAAACCTAGAATGACTTCATG
A-O_cuniculus : CCGGACGCCTATACAATATGAAATACTGTTTCATCAATAGGCTCATTCATTTCCCTAACTGCCGTAATAGTAATAATTTTTATAATCTGAGAAGCCTTCGCCTCAAAACGAGAAGTAGAGACTATTGAACTAACCACCACAAACCTAGAATGACTTCATG
R-M_musculus : CCAGATGCTTACACCACATGAAACACTGTCTCTTCTATAGGATCATTTATTTCACTAACAGCTGTTCTCATCATGATCTTTATAATTTGAGAGGCCTTTGCTTCAAAACGAGAAGTAATATCAGTATCGTATGCTTCAACAAATTTAGAATGACTTCATG
A-M_musculus : CCAGATGCTTACACCACATGAAACACTGTCTCTTCTATAGGATCATTTATTTCACTAACAGCTGTTCTCATCATGATCTTTATAATTTGAGAGGCCTTTGCTTCAAAACGAGAAGTAATATCAGTATCGTATGCTTCAACAAATTTAGAATGACTTCATG
R-R_norvegicus : CCAGATGCTTACACCACATGAAATACAGTCTCCTCTATAGGCTCATTCATCTCACTTACGGCCGTCCTTGTAATGATCTTCATGATTTGAGAAGCCTTCGCATCAAAACGAGAAGTACTCTCAATTTCCTACTCCTCAACTAACCTAGAATGACTGCATG
A-R_norvegicus : CCAGATGCTTACACCACATGAAATACAGTCTCCTCTATAGGCTCATTCATCTCACTTACGGCCGTCCTTGTAATGATCTTCATGATTTGAGAAGCCTTCGCATCAAAACGAGAAGTACTCTCAATTTCCTACTCCTCAACTAACCTAGAATGACTGCATG
R-M_coypus : CCTGATGCTTATACATTTTGAAATACAGTCTCCTCTGTAGGCTCATTTATCTCTTTAACCGCTGTAATAGTAATAATCTTTATAATTTGAGAAGCTTTCGCCTCTAAACGAGAAGTAATAACAACAGAGCTAACCTCTGTAAATCTAGAATGACTTCACG
A-M_coypus : CCCGATGCTTATACATTTTGAAATACAGTCTCCTCTGTAGGCTCATTTATCTCTTTAACCGCTGTAATAGTAATAATCTTTATAATTTGAGAAGCTTTCGCCTCTAAACGAGAAGTAATAACAACAGAGCTAACCTCTGTAAATCTAGAATGACTTCACG
R-A_platyrhynchos : CCTGATGCCTACACACTGTGAAACACCGTCTCCTCTATTGGGTCCCTGATCTCAATAGTGGCCGTAATCATACTAATGTTCATCATCTGAGAAGCCTTCTCAGCCAAACGGAAAGTCCTCCAACCAGAATTAACCGCCACAAACATTGAGTGAATCCACG
A-A_platyrhynchos : CCTGATGCCTACACACTGTGAAACACCGTCTCCTCTATTGGGTCCCTGATCTCAATAGTGGCCGTAATCATACTAATGTTCATCATCTGAGAAGCCTTCTCAGCCAAACGGAAAGTCCTCCAACCAGAATTAACCGCCACAAACATTGAGTGAATCCACG
R-G_gallus : CCAGACGCCTACACACTATGAAACACACTATCCTCAATCGGCTCCTTAATTTCAATAACAGCCGTAATCATACTCATATTCATCGTCTGAGAAGCCTTCTCAGCAAAACGAAAAGTACTCCAACCCGAATTAACTGCCACTAATATCGAATGAATTCATG
A-G_gallus : CCAGACGCCTACACACTATGAAACACACTATCCTCAATCGGCTCCTTAATTTCAATAACAGCCGTAATCATACTCATATTCATCGTCTGAGAAGCCCTCTCAGCAAAACGAAAAGTACTCCAACCCGAATTAACTGCCACTAATATCGAATGAATTCATG
 CCAGATGCATATACAACATGAAATACTGTCTCCTCTATAGGCTCATTCATCTCACTAACAGCAGTAATACTAATAATCTTCATAATCTGAGAAGCCTTCGCATCAAAACGAGAAGTABTAACAGTAGAACTAACCACAACAAACCTAGAATGACTACACG

 * 8500 * 8520 * 8540 * 8560 * 8580 * 8600 * 8620 * 8640
R-E_caballus : GATGCCCCCCACCATACCACACATTTGAAGAACCCACCTACGTAAA--CCTAAAATAA----------GAAAGGAAGGAATCGAACCCCCTCTAACTGGTTTCAAGCCAATATCAT--AACCACT--ATG--TCTTTCTCCATCAAT-TGAGGTATTAGT
A-E_caballus : GATGCCCCCCACCATACCACACATTTGAAGAACCCACCTACGTAAA--CCTAAAATAA----------GAAAGGAAGGAATCGAACCCCCTCTAACTGGTTTCAAGCCAATATCAT--AACCACT--ATG--TCTTTCTCCATCAAT-TGAGGTATTAGT
R-C_bactrianus : GATGTCCTCCACCCTACCATACCTTTGAAGAACCGACCTATATTAA--CCTAAAATAG------ATAAGAAAGGAAGGAATCGAACCCTCTTTAATTGGTTTCAAGCCAACCCTAT--AGCCACT--ATA--ACTTTCTCGATCTA---GAGATATTAGT
A-C_bactrianus : GATGTCCTCCACCCTACCATACCTTTGAAGAACCGACCTATATTAA--CCTAAAATAG------ATAAGAAAGGAAGGAATCGAACCCTCTTTAATTGGTTTCAAGCCAACCCTAT--AGCCACT--ATA--ACTTTCTCGATCTA---GAGATATTAGT
R-C_lupus_familiaris : GATGTCCCCCTCCATACCACACGTTCGAAGAACCTACATATGTGAT--CCAAAAATAA----------GAAAGGAAGGAATCGAACCCCCTAAAATTGGTTTCAAGCCAATGTCAT--AACCATT--ATG--TCTTTCTCAATCAG---GAGGTATTAGT
A-C_lupus_familiaris : GATGTCCCCCTCCATACCACACGTTCGAAGAACCTACATATGTGAT--CCAAAAATAA----------GAAAGGAAGGAATCGAACCCCCTAAAATTGGTTTCAAGCCAATGTCAT--AACCATT--ATG--TCTTTCTCAATCAG---GAGGTATTAGT
R-N_procyonoides : GATGTCCCCCTCCATACCACACATTTGAGGAACCCACATACGTTAT--CCAAAAATAATATTAAACAAGAAAGGAAGGAATCGAACCCCCTAAAGCTGGTTTCAAGCCAGCACCAT--AACCATT--ATG--TCTTTCTTGATTAG---GAGATATTAGT
A-N_procyonoides : GATGTCCCCCTCCATACCACACATTTGAGGAACCCACATACGTTAT--CCAAAAATAATATTAAACAAGAAAGGAAGGAATCGAACCCCCTAAAGCTGGTTTCAAGCCAGCACCAT--AACCATT--ATG--TCTTTCTTGATTAG---GAGATATTAGT
R-V_lagopus : GATGTCCTCCTCCATACCATACATTTGAGGAGCCCACATATGTCAT--CCAAAAATAA----------GAAAGGAAGGAATCGAACCCCCTAAAACTGGTTTCAAGCCAGCACCAT--AACCACT--ATG--TCTTTCTCAATTAG---GAGGTATTAGT
A-V_lagopus : GATGTCCTCCTCCATACCATACATTTGAGGAGCCCACATATGTCAT--CCAAAAATAA----------GAAAGGAAGGAATCGAACCCCCTAAAACTGGTTTCAAGCCAGCACCAT--AACCACT--ATG--TCTTTCTCAATTAG---GAGGTATTAGT
R-M_putorius : GGTGTCCCCCTCCATACCACACATTCGAAGAACCAACCTACGTACT--ATCAAAATAA----------GAAAGGAAGGAATCGAACCCCCTAAGACTGGTTTCAAGCCAATATCAT--AACCACT--ATG--TCTTTCTCGAT-AG---GAGGTATTAGT
A-M_putorius : GGTGTCCCCCTCCATACCACACATTCGAAGAACCAACCTACGTACT--ATCAAAATAA----------GAAAGGAAGGAATCGAACCCCCTAAGACTGGTTTCAAGCCAATATCAT--AACCACT--ATG--TCTTTCTCGAT-AG---GAGGTATTAGT
R-B_taurus : GATGCCCTCCACCATATCACACATTTGAAGAACCCACCTATGTTAA--CCTAAAATAAG----------AAAGGAAGGAATCGAACCCCCTACTATTGGTTTCAAGCCAACATCAT--AACCTCT--ATG--TCTCTCTCAATAAAC--GAGGTGTTAGT
A-B_taurus : GATGCCCTCCACCATATCACACATTTGAAGAACCCACCTATGTTAA--CCTAAAATAAG----------AAAGGAAGGAATCGAACCCCCTACTATTGGTTTCAAGCCAACATCAT--AACCTCT--ATG--TCTCTCTCAATAAAC--GAGGTGTTAGT
R-O_aries : GATGTCCTCCACCATACCACACATTTGAAGAACCCACATATGTTAA--CCTAAAATAAG----------AAAGGAAGGAATCGAACCTCCTATTATTGGTTTCAAGCCAACACCAT--AGCCACT--ATG--ACTCTCTCAATAAAC--GAGATGTTAGT
A-O_aries : GATGTCCTCCACCATACCACACATTTGAAGAACCCACATATGTTAA--CCTAAAATAAG----------AAAGGAAGGAATCGAACCTCCTATTATTGGTTTCAAGCCAACACCAT--AGCCACT--ATG--ACTCTCTCAATAAAC--GAGATGTTAGT
R-S_scrofa : GATGTCCTCCTCCCTATCACACATTTGAAGAACCAACATATATCAA--CCTAAAATAAGC----ATAAGAAAGGAAGGAATCGAACCCTCTCCCACTGGTTTCAAGCCAACGTCAT--AACCACT--ATG--TCTTTCTCGATAATC--GAGGTATTAGT
A-S_scrofa : GATGTCCTCCTCCCTATCACACATTTGAAGAACCAACATATATCAA--CCTAAAATAAGC----ATAAGAAAGGAAGGAATCGAACCCTCTCCCACTGGTTTCAAGCCAACGTCAT--AACCACT--ATG--TCTTTCTCGATAATC--GAGGTATTAGT
R-O_cuniculus : GATGCCCTCCCCCATACCATACATTTGAAGAACCCGCTTTCGTAAAAGCTTAGTACAA----------GAAAGGAAGGAATCGAACCTCCTAAAACTGGTTTCAAGCCAGCCCCAT--AGCCACT--ATG--ACTTTCTTGAT------AAGATATTAGT
A-O_cuniculus : GATGCCCTCCCCCATACCATACATTTGAAGAACCCGCTTTCGTAAAAGCTTAGTACAA----------GAAAGGAAGGAATCGAACCTCCTAAAACTGGTTTCAAGCCAGCCCCAT--AGCCACT--ATG--ACTTTCTTGAT------AAGATATTAGT
R-M_musculus : GCTGCCCTCCACCATATCACACATTCGAGGAACCAACCTATGTAAAAGTAAAATA------------AGAAAGGAAGGAATCGAACCCCCTAAAATTGGTTTCAAGCCAATCTCAT--ATCCTAT--ATG--TCTTTCTCAA------TAAGATATTAGT
A-M_musculus : GCTGCCCTCCACCATATCACACATTCGAGGAACCAACCTATGTAAAAGTAAAATA------------AGAAAGGAAGGAATCGAACCCCCTAAAATTGGTTTCAAGCCAATCTCAT--ATCCTAT--ATG--TCTTTCTCAA------TAAGATATTAGT
R-R_norvegicus : GATGCCCCCCACCCTACCACACATTCGAAGAACCTTCCTATGTAAAAGTTAAATA------------AGAAAGGAAGGATTCGAACCCCCTACAACTGGTTTCAAGCCAATTTCAT--AACCATT--ATG--TCTTTCTCAA------TGAGATATTAGT
A-R_norvegicus : GATGCCCCCCACCCTACCACACATTCGAAGAACCTTCCTACGTAAAAGTTAAATA------------AGAAAGGAAGGATTCGAACCCCCTACAACTGGTTTCAAGCCAATTTCAT--AACCATT--ATG--TCTTTCTCAA------TGAGATATTAGT
R-M_coypus : GATGCCCTCCGTCATATCATACGTTTGAAGAACCTACATACATTAAAATTTAATATACA--------AGAAAGGAAGGAATTGAACCCCCAAAGACTAGTTTCAAGCCAGCCTCAT--AACCTTT--ATG--TCTTTCTTTACCGCGTTTAGATATTAGT
A-M_coypus : GATGCCCTCCGTCATATCATACGTTTGAAGAACCTACATACATTAAAATTTAATATACA--------AGAAAGGAAGGAATTGAACCCCCAAAGACTAGTTTCAAGCCAGCCTCAT--AACCTTT--ATG--TCTTTCTTTACCGCGTTTAGATATTAGT
R-A_platyrhynchos : GCTGCCCCCCTCCATACCACACCTTCGAGGAGCCAGCTTTCGTTCA-----AGTACAA----------GAAAGGAAGGAATCGAACCTCCATACACTGGTTTCAAGCCAGCTGCATTAA-CCACTCA-TGCTTCTTTCTCAT-------GAGATGTTAGT
A-A_platyrhynchos : GCTGCCCCCCTCCATACCACACCTTCGAGGAGCCAGCTTTCGTTCA-----AGTACAA----------GAAAGGAAGGAATCGAACCTCCATACACTGGTTTCAAGCCAGCTGCATTAA-CCACTCA-TGCTTCTTTCTCAT-------GAGATGTTAGT
R-G_gallus : GCTGCCCACCCCCATACCACACCTTCGAAGAACCAGCCTTTGTACA-----AGTGCAA----------GAAAGGAAGGAATCGAACCCTCACATGTTGGTTTCAAGCCAACCGCATCAAACCACTTAATGCTTCTTTCTTAT-------GAGACGTTAGT
A-G_gallus : GCTGCCCACCCCCATACCACACCTTCGAAGAACCAGCCTTTGTACA-----AGTGCAA----------GAAAGGAAGGAATCGAACCCTCACATGTTGGTTTCAAGCCAACCGCATCAAACCACTTAATGCTTCTTTCTTAT-------GAGACGTTAGT
 GATGCCCTCCWCCATACCACACATTTGAAGAACCCACCTATGTWAAAGCCTAAAATAAGMTTAAATAAGAAAGGAAGGAATCGAACCCCCTAAAACTGGTTTCAAGCCAACATCATYAAACCACTYAATGCTTCTTTCTCAATCARCTTGAGATATTAGT

 * 8660 * 8680 * 8700 * 8720 * 8740 * 8760 * 8780 * 8800
R-E_caballus : AAAA---ATTACATGACTTTGTCAAAGTTAAATTATAGGTTAAACCCCTATATACCTC-TATGGCCTACCCCTTCCAACTAGGATTCCAAGACGCAACATCCCCTATTATAGAAGAACTCCTACACTTCCACGACCACACACTAATAATCGTATTCCTAA
A-E_caballus : AAAA---ATTACATAACTTTGTCAAAGTTAAATTATAGGTTAAACCCCTATATACCTC-TATGGCCTACCCCTTCCAACTAGGATTCCAAGACGCAACATCCCCTATTATAGAAGAACTCCTACACTTCCACGACCACACACTAATAATCGTATTCCTAA
R-C_bactrianus : AAAAT---TTACATAGCCTTGTCAAGGCTAAATTACAGGTGAAACCCCTGTATATCTC-TATGCCGTATCCTTTTCAACTAGGCTTCCAAGACGCTACCTCCCCCATTATAGAAGAGTTACTATATTTTCATGATCATACCTTAATAATTGTATTTTTAA
A-C_bactrianus : AAAAT---TTACATAGCCTTGTCAAGGCTAAATTACAGGTGAAACCCCTGTATATCTC-TATGCCGTATCCTTTTCAACTAGGCTTCCAAGACGCTACCTCCCCCATTATAGAAGAGTTACTATATTTTCATGATCATACCTTAATAATTGTATTTTTAA
R-C_lupus_familiaris : AAAAC--ATTACATGACTTTGTCAAAGTTAAATTATAGGTGAAACCCCTATATATCTC-TATGGCGTACCCATTTCAACTCGGATTACAGGACGCAACCTCCCCTATTATAGAGGAGCTACTTCATTTTCATGACCATACACTAATAATTGTATTCTTAA
A-C_lupus_familiaris : AAAAC--ATTACATGACTTTGTCAAAGTTAAATTATAGGTGAAACCCCTATATATCTC-TATGGCGTACCCATTTCAACTCGGATTACAGGACGCAACCTCCCCTATTATAGAGGAGCTACTTCATTTTCATGACCATACACTAATAATTGTATTCTTAA
R-N_procyonoides : AAAAC--ATTACATGACCTTGTCGAGGTCAAGTTATGGGTAAAAACCCCATATATCTC-TATGGCGTACCCTTTTCAACTCGGATTACAGGACGCAACCTCCCCTATCATAGAGGAGCTACTTCATTTTCATGATCATACATTAATAATTGTATTCCTAA
A-N_procyonoides : AAAAC--ATTACATGACCTTGTCGAGGTCAAGTTATGGGTAAAAACCCCATATATCTC-TATGGCGTACCCTTTTCAACTCGGATTACAGGACGCAACCTCCCCTATCATAGAGGAGCTACTTCATTTTCATGATCATACATTAATAATTGTATTCCTAA
R-V_lagopus : AAAAC--ATTACATGACTTTGTCAAAGTCAAATTATAGGTGAAATTCCTATATATCTC-TATGGCGTACCCTTTTCAACTCGGATTACAGGACGCAACCTCCCCTATTATAGAGGAGTTACTTCATTTTCATGATCATACCCTAATAATTGTATTCTTAA
A-V_lagopus : AAAAC--ATTACATGACTTTGTCAAAGTCAAATTATAGGTGAAATTCCTATATATCTC-TATGGCGTACCCTTTTCAACTCGGATTACAGGACGCAACCTCCCCTATTATAGAGGAGTTACTTCATTTTCATGATCATACCCTAATAATTGTATTCTTAA
R-M_putorius : AAAA---ATTACATGACTTTGTCAAAGTCAAATTATAGGTGAAAGTCCTTTATATCTC-TATGGCATACCCTTTCCAAATAGGCCTCCAAGATGCAGCCTCTCCTATCATAGAGGAACTTCTACACTTTCACGATCATACACTAATAATTGTTTTTCTAA
A-M_putorius : AAAA---ATTACATGACTTTGTCAAAGTCAAATTATAGGTGAAAGTCCTTTATATCTC-TATGGCATACCCTTTCCAAATAGGCCTCCAAGATGCAGCCTCTCCTATCATAGAGGAACTTCTACACTTTCACGATCATACACTAATAATTGTTTTTCTAA
R-B_taurus : AAAAC--ATTATATAATTTTGTCAAAGTTAAGTTACAAGTGAAAGTCCTGTACACCTCATATGGCATATCCCATACAACTAGGATTCCAAGATGCAACATCACCAATCATAGAAGAACTACTTCACTTTCATGACCACACGCTAATAATTGTCTTCTTAA
A-B_taurus : AAAAC--ATTATATAATTTTGTCAAAGTTAAGTTACAAGTGAAAGTCCTGTACACCTCATATGGCATATCCCATACAACTAGGATTCCAAGATGCAACATCACCAATCATAGAAGAACTACTTCACTTTCATGACCACACGCTAATAATTGTCTTCTTAA
R-O_aries : AAAAC--ATTACATAACCTTGTCAAGATTAAATTACAGGTGAAAATCCCGTACATCTCATATGGCATATCCCATACAACTAGGCTTTCAAGACGCAACATCACCTATCATGGAAGAACTACTACACTTTCACGACCACACATTAATAATCGTTTTCCTAA
A-O_aries : AAAAC--ATTACATAACCTTGTCAAGATTAAATTACAGGTGAAAATCCCGTACATCTCATATGGCATATCCCATACAACTAGGCTTTCAAGACGCAACATCACCTATCATGGAAGAACTACTACACTTTCACGACCACACATTAATAATCGTTTTCCTAA
R-S_scrofa : AAAAT--ATTACATAACTTTGTCGAAGTTATATTATAGGTGAAAACCCTATATGCCTC-TATGGCTTACCCTTTCCAACTAGGCTTCCAAGACGCCACTTCACCCATCATAGAAGAACTCCTACACTTTCACGATCACACCTTAATAATTGTATTCTTAA
A-S_scrofa : AAAAT--ATTACATAACTTTGTCGAAGTTATATTATAGGTGAAAACCCTATATGCCTC-TATGGCTTACCCTTTCCAACTAGGCTTCCAAGACGCCACTTCACCCATCATAGAAGAACTCCTACACTTTCACGATCACACCTTAATAATTGTATTCTTAA
R-O_cuniculus : AAAAC-CATTACATAACTTTGTCGAAGTTAATTTATAGGTTCAACTCCTATATATCTT-TATGGCGTACCCCTTCCAACTGGGCTTTCAGGATGCCTCATCCCCTATTATAGAAGAATTACTTCACTTTCATGACCACACTCTCATAATCGTCTTTCTAA
A-O_cuniculus : AAAAC-CATTACATAACTTTGTCGAAGTTAATTTATAGGTTCAACTCCTATATATCTT-TATGGCGTACCCCTTCCAACTGGGCTTTCAGGATGCCTCATCCCCTATTATAGAAGAATTACTTCACTTTCATGACCACACTCTCATAATCGTCTTTCTAA
R-M_musculus : AAAATCAATTACATAACTTTGTCAAAGTTAAATTATAGATCAATAATCTATATATCTTATATGGCCTACCCATTCCAACTTGGTCTACAAGACGCCACATCCCCTATTATAGAAGAGCTAATAAATTTCCATGATCACACACTAATAATTGTTTTCCTAA
A-M_musculus : AAAATCAATTACATAACTTTGTCAAAGTTAAATTATAGATCAATAATCTATATATCTTATATGGCCTACCCATTCCAACTTGGTCTACAAGACGCCACATCCCCTATTATAGAAGAGCTAATAAATTTCCATGATCACACACTAATAATTGTTTTCCTAA
R-R_norvegicus : AAAAT-AATTACATAACCTTGTCAAGGTTAAGTTATAGACTTA-AATCTATATATCTTACATGGCTTACCCATTTCAACTTGGCTTACAAGACGCCACATCACCTATCATAGAAGAACTTACAAACTTTCATGACCACACCCTAATAATTGTATTCCTCA
A-R_norvegicus : AAAAT-AATTACATAACCTTGTCAAGGTTAAATTATAGACTTA-AATCTATATATCTTACATGGCTTACCCATTTCAACTTGGCTTACAAGACGCCACATCACCTATCATAGAAGAACTTACAAACTTTCATGACCACACCCTAATAATTGTATTCCTCA
R-M_coypus : AAAAC-TATTACATAACTTTGTCAAAGTTAAATTACTGGCTTATACCCTGTATATCTTAAATGGCATACCCTTATGAACTAGGCTTTCAAGACGCTACCTCACCCATTATAGAAGAACTCCTTCACTTTCATGACCATACGCTCATAATCGTTTTTTTAA
A-M_coypus : AAAAC-TATTACATAACTTTGTCAAAGTTAAATTACTGGTTTATACCCTGTATATCTTAAATGGCATACCCTTATGAACTAGGCTTTCAAGACGCTACCTCACCCATTATAGAAGAACTCCTTCACTTTCATGACCATACGCTCATAATCGTTTTTTTAA
R-A_platyrhynchos : AAACC-AATTACATAGCCTTGTCAAGGCTAAATCACAGGTGAAAGCCCTGTACATCTCACGTGGCCAACCACTCCCAACTAGGATTCCAAGACGCCTCATCACCCATTATAGAAGAACTCGTTGAATTCCACGACCACGCTCTGATTGTTGCCTTAGCTA
A-A_platyrhynchos : AAACC-AATTACATAGCCTTGTCAAGGCTAAATCACAGGTGAAAGCCCTGTACATCTCACGTGGCCAACCACTCCCAACTAGGATTCCAAGACGCCTCATCACCCATTATAGAAGAACTCGTTGAATTCCACGACCACGCTCTGATTGTTGCCTTAGCTA
R-G_gallus : AAACC-AATTACATAGACCTGTCAAGACTAAATCACAGGTGCAAACCCTGTACATCTCATATGGCCAACCACTCCCAACTAGGCTTTCAAGACGCCTCATCCCCCATCATAGAAGAGCTCGTTGAATTCCACGACCACGCCCTGATAGTCGCACTAGCAA
A-G_gallus : AAACC-AATTACATAGACCTGTCAAGACTAAATCACAGGTGCAAACCCTGTACATCTCACATGGCCAACCACTCCCAACTAGGCTTTCAAGACGCCTCATCCCCCATCATAGAAGAGCTCGTTGAATTCCACGACCACGCCCTGATAGTCGCACTAGCAA
 AAAACCAATTACATAACTTTGTCAAAGTTAAATTATAGGTGAAAACCCTATATATCTCATATGGCGTACCCYTTCCAACTAGGCTTCCAAGACGCAACATCCCCTATTATAGAAGAACTACTTCACTTTCATGACCACACACTAATAATTGTATTCCTAA

 * 8820 * 8840 * 8860 * 8880 * 8900 * 8920 * 8940 * 8960
R-E_caballus : TTAGCTCTCTAGTATTATATATTATCTCATCAATACTAACAACTAAATTAACCCATACCAGCACCATAGATGCTCAAGAAGTAGAGACAATTTGAACGATTTTACCAGCCATCATCCTTATTCTAATCGCCCTCCCATCCCTACGAATTCTATATATAAT
A-E_caballus : TTAGCTCTCTAGTATTATATATTATCTCATCAATACTAACAACTAAATTAACCCATACCAGCACCATAGATGCTCAAGAAGTAGAGACAATTTGAACGATTTTACCAGCCATCATCCTTATTCTAATCGCCCTCCCATCCCTACGAATTCTATATATAAT
R-C_bactrianus : TTAGCTCTCTGGTTTTGTATATCATTACTCTAATGCTAACAACTAAATTAACACACACAAGTACTATAGACGCACAAGAAGTCGAGACGATCTGAACCATCCTGCCTGCCATTATTTTAATTACAATTGCCCTTCCGTCACTACGAATCCTTTATATAAT
A-C_bactrianus : TTAGCTCTCTGGTTTTGTATATCATTACTCTAATGCTAACAACTAAATTAACACACACAAGTACTATAGACGCACAAGAAGTCGAGACGATCTGAACCATCCTACCTGCCATTATTTTAATTACAATTGCCCTTCCGTCACTACGAATCCTTTATATAAT
R-C_lupus_familiaris : TCAGTTCTTTAGTTCTCTATATCATTTCACTAATATTGACTACAAAATTAACCCATACAAGCACAATAGACGCACAAGAAGTGGAAACAGTATGAACCATTCTACCCGCCATTATCCTAATCCTAATCGCTCTACCTTCCCTCCGAATCCTTTATATAAT
A-C_lupus_familiaris : TCAGTTCTTTAGTTCTCTATATCATTTCACTAATATTGACTACAAAATTAACCCATACAAGCACAATAGACGCACAAGAAGTGGAAACAGTATGAACCATTCTACCCGCCATTATCCTAATCCTAATCGCTCTACCTTCCCTCCGAATCCTTTATATAAT
R-N_procyonoides : TCAGCTCATTAGTTCTTTACATTATCTCATTGATACTAACCACTAAACTAACACATACAAGTACTATAGACGCACAAGAGGTTGAAACGGTATGGACCATCTTACCAGCCATTATTCTCATCCTAATCGCCCTACCTTCTCTCCGAATTCTCTACATAAT
A-N_procyonoides : TCAGCTCATTAGTTCTTTACATTATCTCATTGATACTAACCACTAAACTAACACATACAAGTACTATAGACGCACAAGAGGTTGAAACGGTATGGACCATCTTACCAGCCATTATTCTCATCCTAATCGCCCTACCTTCTCTCCGAATTCTCTACATAAT
R-V_lagopus : TCAGCTCATTAGTTCTTTATATTATTACCTTAATATTAACCACCAAGCTAACTCATACAAGTACAATAGACGCACAAGAGGTAGAAACAGTCTGAACAATTCTACCAGCCATCATCCTAATCCTAATCGCTCTGCCCTCTCTACGGATTCTCTACATAAT
A-V_lagopus : TCAGCTCATTAGTTCTTTATATTATTACCTTAATATTAACCACCAAGCTAACTCATACAAGTACAATAGACGCACAAGAGGTAGAAACAGTCTGAACAATTCTACCAGCCATCATCCTAATCCTAATCGCTCTGCCCTCTCTACGGATTCTCTACATAAT
R-M_putorius : TTAGTTCTCTTGTACTTTACATTATTTCAGTAATATTAACTACCAAGCTTACGCATACAAGTACTATAGACGCCCAAGCAGTTGAAACGATCTGAACCATCCTACCAGCCATTATTTTGATCATAATCGCTCTACCCTCGCTACGAATTCTCTATATGAT
A-M_putorius : TTAGTTCTCTTGTACTTTACATTATTTCAGTAATATTAACTACCAAGCTTACGCATACAAGTACTATAGACGCCCAAGCAGTTGAAACGATCTGAACCATCCTACCAGCCATTATTTTGATCATAATCGCTCTACCCTCGCTACGAATTCTCTATATGAT
R-B_taurus : TTAGCTCATTAGTACTTTACATTATTTCACTAATACTAACGACAAAGCTGACCCATACAAGCACGATAGATGCACAAGAAGTAGAGACAATCTGAACCATTCTGCCCGCCATCATCTTAATTCTAATTGCTCTTCCTTCTTTACGAATTCTATACATAAT
A-B_taurus : TTAGCTCATTAGTACTTTACATTATTTCACTAATACTAACGACAAAGCTGACCCATACAAGCACGATAGATGCACAAGAAGTAGAGACAATCTGAACCATTCTGCCCGCCATCATCTTAATTCTAATTGCTCTTCCTTCTTTACGAATTCTATACATAAT
R-O_aries : TCAGCTCTCTAGTACTTTATATTATTTCACTAATACTAACAACAAAATTAACCCATACCAGTACCATAGACGCGCAAGAAGTAGAAACAATCTGAACCATTCTACCAGCCATTATCTTAATTATGATTGCTCTTCCATCCTTGCGAATCCTATACATAAT
A-O_aries : TCAGCTCTCTAGTACTTTATATCATTTCACTAATACTAACAACAAAATTAACCCATACCAGTACCATAGACGCGCAAGAAGTAGAAACAATCTGAACTATTCTACCAGCCATTATCTTAATTATGATTGCTCTTCCATCCTTACGAATCCTATACATAAT
R-S_scrofa : TCAGCTCTTTAGTGTTATATATCATTTCACTTATACTAACAACAAAACTGACACACACTAGCACAATGGATGCCCAAGAAGTAGAAACAATTTGAACAATCCTACCCGCTATTATTTTAATTCTTATTGCCCTTCCATCATTACGAATCCTTTATATAAT
A-S_scrofa : TCAGCTCTTTAGTGTTATATATCATTTCACTTATACTAACAACAAAACTGACACACACTAGCACAATGGATGCCCAAGAAGTAGAAACAATTTGAACAATCCTACCCGCTATTATTTTAATTCTTATTGCCCTTCCATCATTACGAATCCTTTATATAAT
R-O_cuniculus : TCAGCTCCCTAGTCCTTTATATTATTTCTCTTATATTAACTACAAAGCTCACTCACACAAGCACAATGGATGCTCAGGAGGTAGAGACAATCTGAACCATCCTCCCAGCCATTATTCTTATTTTAATTGCGCTGCCCTCCCTACGAATCCTATATATAAT
A-O_cuniculus : TCAGCTCCCTAGTCCTTTATATTATTTCTCTTATATTAACTACAAAGCTCACTCACACAAGCACAATGGACGCTCAAGAGGTAGAGACAATCTGAACCATCCTCCCAGCCATTATTCTTATTTTAATTGCGCTGCCCTCCCTACGAATCCTATATATAAT
R-M_musculus : TTAGCTCCTTAGTCCTCTATATCATCTCGCTAATATTAACAACAAAACTAACACATACAAGCACAATAGATGCACAAGAAGTTGAAACCATTTGAACTATTCTACCAGCTGTAATCCTTATCATAATTGCTCTCCCCTCTCTACGCATTCTATATATAAT
A-M_musculus : TTAGCTCCTTAGTCCTCTATATCATCTCGCTAATATTAACAACAAAACTAACACATACAAGCACAATAGATGCACAAGAAGTTGAAACCATTTGAACTATTCTACCAGCTGTAATCCTTATCATAATTGCTCTCCCCTCTCTACGCATTCTATATATAAT
R-R_norvegicus : TCAGCTCCCTAGTACTTTATATTATTTCACTAATACTAACAACAAAACTAACACACACAAGCACAATAGACGCCCAAGAAGTAGAAACAATTTGAACAATTCTCCCAGCTGTCATTCTTATTCTAATTGCCCTTCCCTCCCTACGAATTCTATACATAAT
A-R_norvegicus : TCAGCTCCCTAGTACTTTATATTATTTCACTAATACTAACAACAAAACTAACACACACAAGCACAATAGACGCCCAAGAAGTAGAAACAATTTGAACAATCCTCCCAGCTGTCATTCTTATCCTAATTGCCCTTCCCTCCCTACGAATTCTATACATAAT
R-M_coypus : TCAGTACATTAGTTCTATATCTCATCTCTCTTATATTAACAACAAAGTTAACACATACTAGTACTATAGATGCTCAAGAAATTGAAACTATTTGAACTATTCTTCCTGCCATTATTCTTATCATAATTGCATTACCATCATTACGAATTTTATATATAAT
A-M_coypus : TCAGTACATTAGTTCTATATCTCATCTCTCTTATATTAACAACAAAGTTAACACATACTAGTACTATAGATGCTCAAGAAATTGAAACTATTTGAACTATTCTTCCTGCCATTATTCTTATCATAATTGCATTACCATCATTACGAATTTTATATATAAT
R-A_platyrhynchos : TCTGCAGCCTAGTCCTATACCTCTTAGCCCACATGCTAATAGAAAAACTA--TCAT-CCAACGCAGTAGACGCCCAAGAAGTAGAACTAATCTGAACAATCCTACCCGCCATCGTCCTAGTACTCCTCGCCCTCCCATCCCTACAAATCCTGTACATAAT
A-A_platyrhynchos : TCTGCAGCCTAGTCCTATACCTCTTAGCCCACATGCTAATAGAAAAACTA--TCAT-CCAACGCAGTAGACGCCCAAGAAGTAGAACTAATCTGAACAATCCTACCCGCCATCGTCCTAGTACTCCTCGCCCTCCCATCCCTACAAATCCTGTACATAAT
R-G_gallus : TTTGCAGCTTAGTACTCTACCTTCTAACTCTTATACTTATAGAAAAACTA--TCAT-CAAACACCGTAGATGCCCAAGAAGTTGAACTAATCTGAACCATCCTACCCGCTATTGTCCTAGTCCTGCTTGCCCTCCCCTCCCTCCAAATCCTCTACATAAT
A-G_gallus : TTTGCAGCTTAGTACTCTACCTTCTAACTCTTACACTTATAGAAAAACTA--TCAT-CAAACACCGTAGATGCCCAAGAAGTTGAACTAATCTGAACCATCCTACCCGCTATTGTCCTAGTCCTGCTTGCCCTCCCCTCCCTCCAAATCCTCTACATAAT
 TCAGCTCTTTAGTACTTTATATTATTTCACTAATACTAACAACAAAACTAACACATACAAGCACAATAGACGCACAAGAAGTAGAAACAATCTGAACCATYCTACCAGCCATTATCCTAATCCTAATTGCCCTTCCCTCCCTACGAATTCTATATATAAT

 * 8980 * 9000 * 9020 * 9040 * 9060 * 9080 * 9100 * 9120
R-E_caballus : AGATGAAATCAATAATCCGTCCCTCACAGTCAAAACAATAGGCCACCAATGATACTGAAGCTACGAGTATACCGATTACGAAGACTTGACCTTTGACTCCTACATGATCCCCACATCAGACCTAAAACCAGGAGAATTACGTCTTCTAGAAGTCGACAAT
A-E_caballus : AGATGAAATCAATAATCCGTCCCTCACAGTCAAAACAATAGGCCACCAATGATACTGAAGCTACGAGTATACCGATTACGAAGACTTGACCTTTGACTCCTACATGATCCCCACATCAGACCTAAAACCAGGAGAATTACGTCTTCTAGAAGTCGACAAT
R-C_bactrianus : AGACGAGATTAATAATCCGGTCTTAACCGTCAAAACGATTGGTCATCAATGATATTGAAGCTACGAGTATACAGACTACGAAACCCTTAGTTTTGACTCCTATATAATCCCAACGTCAGACCTAAAACCAGGTGAGCTACGACTGCTTGAAGTAGATAAT
A-C_bactrianus : AGACGAGATTAATAATCCGGTCTTAACCGTCAAAACGATTGGTCATCAATGATATTGAAGCTACGAGTATACAGACTACGAAACCCTTAGTTTTGACTCCTATATAATCCCAACGTCAGACCTAAAACCAGGTGAGCTACGACTGCTTGAAGTAGATAAT
R-C_lupus_familiaris : GGACGAAATTAATAACCCCTCTTTAACCGTGAAAACAATAGGCCACCAATGATACTGAAGCTATGAATATACTGACTATGAAGACTTAAACTTTGACTCCTACATAATCCCAACACAAGAATTAAAGCCAGGAGAACTCCGACTATTAGAAGTAGACAAC
A-C_lupus_familiaris : GGACGAAATTAATAACCCCTCTTTAACCGTGAAAACAATAGGCCACCAATGATACTGAAGCTATGAATATACTGACTATGAAGACTTAAACTTTGACTCCTACATAATCCCAACACAAGAATTAAAGCCAGGAGAACTCCGACTATTAGAAGTAGACAAC
R-N_procyonoides : GGACGAAATCAATAACCCATCCTTAACAGTAAAAACGATAGGCCACCAATGGTACTGAAGCTATGAGTATACCGACTACGAAGACCTAAATTTTGATTCTTACATAATCCCAACACAGGAGTTAAAACCAGGAGAGCTTCGACTGCTAGAAGTCGACAAT
A-N_procyonoides : GGACGAAATCAATAACCCATCCTTAACAGTAAAAACGATAGGCCACCAATGGTACTGAAGCTATGAGTATACCGACTACGAAGACCTAAATTTTGATTCTTACATAATCCCAACACAGGAGTTAAAACCAGGAGAGCTTCGACTGCTAGAAGTCGACAAT
R-V_lagopus : GGATGAAATTAATAACCCATCACTAACTGTAAAAACAATAGGCCACCAATGATACTGAAGCTACGAATACACTGACTACGAAGACCTAAACTTTGACTCTTACATAATTCCCACACAAGAATTAAAACCAGGGGAGCTTCGACTATTAGAAGTCGATAAC
A-V_lagopus : GGATGAAATTAATAACCCATCACTAACTGTAAAAACAATAGGCCACCAATGATACTGAAGCTACGAATACACTGACTACGAAGACCTAAACTTTGACTCTTACATAATTCCCACACAAGAATTAAAACCAGGGGAGCTTCGACTATTAGAAGTCGATAAC
R-M_putorius : AGACGAGATCAATAACCCCTCTTTAACCGTAAAAACTATGGGTCACCAATGATACTGAAGTTATGAATATACAGACTACGAAGACTTAAACTTCGACTCCTACATAATCCCAACTCAAGAACTGAAACCAGGAGAACTACGACTTCTAGAAGTGGATAAT
A-M_putorius : AGACGAGATCAATAACCCCTCTTTAACCGTAAAAACTATGGGTCACCAATGATACTGAAGTTATGAATATACAGACTACGAAGACTTAAACTTCGACTCCTACATAATCCCAACTCAAGAACTGAAACCAGGAGAACTACGACTTCTAGAAGTGGATAAT
R-B_taurus : AGATGAAATCAATAACCCATCTCTTACAGTAAAAACCATAGGACATCAGTGATACTGAAGCTATGAGTATACAGATTATGAGGACTTAAGCTTCGACTCCTACATAATTCCAACATCAGAATTAAAGCCAGGGGAGCTACGACTATTAGAAGTCGATAAT
A-B_taurus : AGATGAAATCAATAACCCATCTCTTACAGTAAAAACCATAGGACATCAGTGATACTGAAGCTATGAGTATACAGATTATGAGGACTTAAGCTTCGACTCCTACATAATTCCAACATCAGAATTAAAGCCAGGGGAGCTACGACTATTAGAAGTCGATAAT
R-O_aries : AGATGAAATCAACAACCCATCTCTCACAGTAAAGACCATAGGGCATCAATGATACTGAAGCTATGAATATACAGATTATGAAGACCTAAGCTTCGATTCCTATATAATCCCAACATCAGAACTAAAACCAGGAGAACTGCGTTTACTAGAAGTAGACAAC
A-O_aries : AGATGAAATCAACAACCCATCTCTCACAGTAAAGACCATAGGGCATCAATGATACTGAAGCTATGAATATACAGATTATGAAGACCTAAGCTTCGATTCCTATATAATCCCAACATCAGAACTAAAACCAGGAGAACTGCGTTTACTAGAAGTAGATAAC
R-S_scrofa : AGACGAAATTAATAACCCAGCCTTAACCGTAAAAACCATAGGACATCAATGATACTGAAGCTATGAGTATACAGACTATGAAGACCTCACCTTTGACTCATATATAATCCCCACATCAGATCTTAAACCTGGAGAAATACGACTACTAGAAGTAGACAAT
A-S_scrofa : AGACGAAATTAATAACCCAGCCTTAACCGTAAAAACCATAGGACATCAATGATACTGAAGCTATGAGTATACAGACTATGAAGACCTCACCTTTGACTCATATATAATCCCCACATCAGATCTTAAACCTGGAGAAATACGACTACTAGAAGTAGACAAT
R-O_cuniculus : AGATGAAATCAACAACCCCTCTTTAACAGTAAAAACGATAGGCCACCAATGATATTGAAGCTATGAGTACACAGACTATGAGGATTTAAATTTTGATTCTTACATGATCCCAACATCAGACCTGAATCCAGGTGACCTACGACTACTAGAAGTTGACAAT
A-O_cuniculus : AGATGAAATCAACAACCCCTCTTTAACAGTAAAAACGATAGGCCACCAATGATATTGAAGCTATGAGTACACAGACTATGAGGATTTAAATTTTGATTCTTATATGATCCCAACATCAGACCTGAATCCAGGTGACCTACGACTACTAGAAGTTGACAAT
R-M_musculus : AGACGAAATCAACAACCCCGTATTAACCGTTAAAACCATAGGGCACCAATGATACTGAAGCTACGAATATACTGACTATGAAGACCTATGCTTTGATTCATATATAATCCCAACAAACGACCTAAAACCTGGTGAACTACGACTGCTAGAAGTTGATAAC
A-M_musculus : AGACGAAATCAACAACCCCGTATTAACCGTTAAAACCATAGGGCACCAATGATACTGAAGCTACGAATATACTGACTATGAAGACCTATGCTTTGATTCATATATAATCCCAACAAACGACCTAAAACCTGGTGAACTACGACTGCTAGAAGTTGATAAC
R-R_norvegicus : AGACGAGATTAATAACCCAGTTCTAACAGTAAAGACTATAGGACACCAATGATACTGAAGCTATGAATATACTGACTATGAAGACCTATGCTTTGACTCCTACATAATCCCAACCAATGACCTAAAACCAGGTGAACTTCGTCTATTAGAAGTTGATAAT
A-R_norvegicus : AGACGAGATTAATAACCCAGTTCTAACAGTAAAAACTATAGGACACCAATGATACTGAAGCTATGAATATACTGACTATGAAGACCTATGCTTTGACTCCTACATAATCCCAACCAATGACCTAAAACCAGGTGAGCTTCGTCTATTAGAAGTTGATAAT
R-M_coypus : AGATGAAGTTAATAACCCCTTGCTAACGATTAAAACAATAGGACATCAATGATATTGAAGCTATGAATACACAGATTATGAGGAATTGAATTTTGACTCATACATGATTCCCACTACTGATCTAAAACCAGGAGAACTTCGACTGCTTGAAGTTGACAAT
A-M_coypus : AGATGAAGTTAATAACCCCTTGCTAACGATTAAAACAATAGGACATCAATGATATTGAAGCTATGAATACACAGATTATGAAGAATTGAATTTTGACTCATACATGATTCCCACTACTGATCTAAAACCAGGAGAACTTCGACTGCTTGAAGTTGACAAT
R-A_platyrhynchos : AGACGAAATCGACGAGCCAGACCTCACACTAAAAGCCATTGGCCACCAGTGATACTGAAGCTACGAATACACAGACTTCAAGGACCTCTCATTCGACTCCTACATAATTCCCACCACAGACCTGCCAAATGGGCACTTCCGACTCCTAGAAGTTGACCAC
A-A_platyrhynchos : AGACGAAATCGACGAGCCAGACCTCACACTAAAAGCCATTGGCCACCAGTGATACTGAAGCTACGAATACACAGACTTCAAGGACCTCTCATTCGACTCCTACATAATTCCCACCACAGACCTGCCAAATGGGCACTTCCGACTCCTAGAAGTTGACCAC
R-G_gallus : AGACGAAATCGACGAACCTGATCTCACCCTAAAAGCCATCGGACACCAATGATACTGAACCTATGAATACACAGACTTCAAGGACCTCTCATTTGACTCCTACATAACCCCAACAACAGACCTCCCCCTAGGCCACTTCCGCCTACTAGAAGTCGACCAT
A-G_gallus : AGACGAAATCGACGAACCTGACCTCACCCTAAAAGCCATCGGACACCAATGATACTGAACCTATGAATACACAGACTTCAAGGACCTCTCATTTGACTCCTACATAACCCCAACAACAGACCTCCCCCTAGGCCACTTCCGCCTACTAGAAGTCGACCAT
 AGACGAAATCAATAACCCATCTCTAACAGTAAAAACCATAGGCCACCAATGATACTGAAGCTATGAATATACAGACTATGAAGACCTAAACTTTGACTCCTACATAATCCCAACATCAGACCTAAAACCAGGAGAACTACGACTACTAGAAGTYGACAAT

 * 9140 * 9160 * 9180 * 9200 * 9220 * 9240 * 9260 * 9280
R-E_caballus : CGAGTGGTTCTCCCCATAGAAATAACCATCCGAATGCTAATTTCATCCGAAGACGTCCTACACTCATGAGCTGTGCCCTCCCTAGGCCTAAAAACAGACGCTATCCCTGGGCGCCTAAATCAGACAACTCTCGTGGCCTCTCGACCAGGACTTTACTACG
A-E_caballus : CGAGTGGTTCTCCCCATAGAAATAACCATCCGAATGCTAATTTCATCCGAGGACGTCCTACACTCATGAGCTGTGCCCTCCCTAGGCCTAAAAACAGACGCTATCCCTGGGCGCCTAAATCAGACAACTCTCGTGGCCTCTCGACCAGGACTTTACTACG
R-C_bactrianus : CGGGTCGTCCTGCCAATAGAAATAACCATTCGGATACTGGTCACCTCCGAAGACGTACTACACTCGTGAGCAGTCCCCTCTCTAGGATTGAAAACAGACGCGGTCCCCGGGCGCCTAAATCAAATTACACTGATGTCAACACGACCTGGACTCTTCTATG
A-C_bactrianus : CGGGTCGTCCTGCCAATAGAAATAACCATTCGGATACTGGTCACCTCCGAAGACGTACTACACTCGTGAGCAGTCCCCTCTCTAGGATTGAAAACAGACGCGGTCCCCGGGCGCCTAAATCAAATTACACTGATGTCAACACGACCTGGACTCTTCTATG
R-C_lupus_familiaris : CGAGTTGTCCTCCCAATAGAAATAACCATCCGAATACTTATCTCTTCAGAAGACGTTTTGCATTCATGAGCCGTTCCATCACTAGGTCTAAAAACTGACGCTATTCCAGGACGACTAAACCAAACCACCCTTATAGCCATACGACCAGGACTGTACTATG
A-C_lupus_familiaris : CGAGTTGTCCTCCCAATAGAAATAACCATCCGAATACTTATCTCTTCAGAAGACGTTTTGCATTCATGAGCCGTTCCATCACTAGGTCTAAAAACTGACGCTATTCCAGGACGACTAAACCAAACCACCCTTATAGCCATACGACCAGGACTGTACTATG
R-N_procyonoides : CGAGTGATCCTCCCAATAGAAATAACCGTCCGAATATTAATCTCTTCAGAAGATGTACTACACTCATGAGCCGTCCCATCACTAGGCCTTAAAACAGACGCCATCCCAGGACGATTAAACCAGACCACCCTAATAGCCATACGACCAGGACTATACTACG
A-N_procyonoides : CGAGTGATCCTCCCAATAGAAATAACCGTCCGAATATTAATCTCTTCAGAAGATGTACTACACTCATGAGCCGTCCCATCACTAGGCCTTAAAACAGACGCCATCCCAGGACGATTAAACCAGACCACCCTAATAGCCATACGACCAGGACTATACTACG
R-V_lagopus : CGAGTTGTTCTCCCAATAGAAATAACCGTCCGAATACTTATCTCTTCAGAAGACGTATTACACTCATGAGCCGTTCCATCACTAGGCTTAAAAACTGATGCCATCCCAGGACGATTAAATCAAACTACCCTAATAGCGATACGACCAGGGCTATATTACG
A-V_lagopus : CGAGTTGTTCTCCCAATAGAAATAACCGTCCGAATACTTATCTCTTCAGAAGACGTATTACACTCATGAGCCGTTCCATCACTAGGCTTAAAAACTGATGCCATCCCAGGACGATTAAATCAAACTACCCTAATAGCGATACGACCAGGGCTATATTACG
R-M_putorius : CGAGTAGTGCTCCCAATAGAAATAACAATTCGTATACTAATTTCTTCCGAGGATGTATTACACTCATGAGCCGTCCCATCCCTAGGATTAAAAACCGATGCTATCCCAGGACGCCTTAACCAAACTACTATTATAGCCATGCGACCGGGACTATACTACG
A-M_putorius : CGAGTAGTGCTCCCAATAGAAATAACAATTCGTATACTAATTTCTTCCGAGGATGTATTACACTCATGAGCCGTCCCATCCCTAGGATTAAAAACCGATGCTATCCCAGGACGCCTTAACCAAACTACTATTATAGCCATGCGACCGGGACTATACTACG
R-B_taurus : CGAGTTGTACTACCAATAGAAATAACAATCCGAATGTTAGTCTCCTCTGAAGACGTATTACACTCATGAGCTGTGCCCTCTCTAGGACTAAAAACAGACGCAATCCCAGGCCGTCTAAACCAAACAACCCTTATATCGTCCCGTCCAGGCTTATATTACG
A-B_taurus : CGAGTTGTACTACCAATAGAAATAACAATCCGAATGTTAGTCTCCTCTGAAGACGTATTACACTCATGAGCTGTGCCCTCTCTAGGACTAAAAACAGACGCAATCCCAGGCCGTCTAAACCAAACAACCCTTATATCGTCCCGTCCAGGCTTATATTACG
R-O_aries : CGAGTTGTATTACCCATGGAAATAACAGTCCGAATACTAATCTCTTCCGAAGATGTCCTACCCTCATGAGCAGTCCCTTCTCTAGGACTAAAAACAGACGCAATTCCAGGTCGTTTAAATCAAACAACCCTTATGTCAACTCGTCCAGGCCTATTCTACG
A-O_aries : CGAGTTGTATTACCCATGGAAATAACAGTCCGAATACTAATCTCTTCCGAAGATGTCCTACACTCATGAGCAGTCCCTTCTCTAGGACTAAAAACAGACGCAATTCCAGGTCGTTTAAATCAAACAACCCTTATGTCAACTCGTCCAGGCCTATTCTACG
R-S_scrofa : CGAGTTGTTCTGCCAATAGAAATAACAATCCGAATATTAGTGTCCTCTGAAGACGTACTACACTCATGAGCTGTCCCATCCCTCGGTTTAAAAACAGATGCTATCCCAGGACGACTAAACCAAACAACTCTAATATCCACACGACCTGGCCTTTATTACG
A-S_scrofa : CGAGTTGTTCTGCCAATAGAAATAACAATCCGAATATTAGTGTCCTCTGAAGACGTACTACACTCATGAGCTGTCCCATCCCTCGGTTTAAAAACAGATGCTATCCCAGGACGACTAAACCAAACAACTCTAATATCCACACGACCTGGCCTTTATTACG
R-O_cuniculus : CGAGTTGTACTTCCCATAGAACTCCCAATCCGCATGCTAATCTCCTCGGAAGACGTACTTCACTCATGAGCCGTACCATCACTAGGACTAAAAACAGATGCCATTCCCGGACGCTTAAATCAAGCCACACTTATCTCAACTCGACCAGGACTTTTCTATG
A-O_cuniculus : CGAGTTGTACTTCCCATAGAACTCCCAATCCGCATGCTAATCTCCTCGGAAGACGTACTTCACTCATGAGCCGTACCATCACTAGGACTAAAAACAGATGCCATTCCCGGACGCTTAAATCAAGCCACACTTATCTCAACTCGACCAGGACTTTTCTATG
R-M_musculus : CGAGTCGTTCTGCCAATAGAACTTCCAATCCGTATATTAATTTCATCTGAAGACGTCCTCCACTCATGAGCAGTCCCCTCCCTAGGACTTAAAACTGATGCCATCCCAGGCCGACTAAATCAAGCAACAGTAACATCAAACCGACCAGGGTTATTCTATG
A-M_musculus : CGAGTCGTTCTGCCAATAGAACTTCCAATCCGTATATTAATTTCATCTGAAGACGTCCTCCACTCATGAGCAGTCCCCTCCCTAGGACTTAAAACTGATGCCATCCCAGGCCGACTAAATCAAGCAACAGTAACATCAAACCGACCAGGGTTATTCTATG
R-R_norvegicus : CGGGTAGTCTTACCAATAGAACTTCCAATCCGTATACTAATCTCATCCGAAGACGTCCTGCACTCATGAGCCGTCCCTTCACTAGGGTTAAAAACCGACGCAATCCCCGGCCGCCTAAACCAAGCTACAGTGACATCAAACCGACCAGGTCTATTCTATG
A-R_norvegicus : CGGGTAGTCTTACCAATAGAACTTCCAATTCGTATACTAATCTCATCCGAAGACGTCCTGCACTCATGAGCCGTCCCTTCACTAGGGTTAAAAACCGACGCAATCCCCGGCCGCCTAAACCAAGCTACAGTGACATCAAACCGACCAGGTCTGTTCTATG
R-M_coypus : CGAATTGTATTTCCAATAGAAATACCAGTACGTATGTTAATCTCCTCAGAAGACGTATTACACTCATGAGCTGTTCCATCCCTAGGAGTTAAAACAGACGCAATTCCAGGACGACTAAATCAAACAATCCTTACATCGTCCCGCCCAGGTTTGTTTTACG
A-M_coypus : CGAATTGTATTTCCAATAGAAATACCAGTACGTATGTTAATCTCCTCAGAAGACGTATTACACTCATGAGCTGTTCCATCCCTAGGAGTTAAAACAGACGCAATTCCAGGACGACTAAATCAAACAATCCTTACATCGTCCCGCCCAGGTTTGTTTTACG
R-A_platyrhynchos : CGCGTAGTCGTACCCATAGAATCACCGATCCGCGTAATTATTACTGCCGGAGACGTACTTCACTCATGAGCAGTTCCAACGCTCGGAGTTAAAACAGATGCAATCCCAGGCCGACTAAACCAAACCTCATTCATTACCACCCGGCCTGGGATTTTCTACG
A-A_platyrhynchos : CGCGTAGTCGTACCCATAGAATCACCGATCCGCGTAATTATTACTGCCGGAGACGTACTTCACTCATGAGCAGTTCCAACGCTCGGAGTTAAAACAGATGCAATCCCAGGCCGACTAAACCAAACCTCATTCATTACCACCCGGCCTGGGATTTTCTACG
R-G_gallus : CGCATTGTAATCCCCATAGAATCCCCCATTCGAGTAATCATCACCGCTGATGACGTCCTCCACTCATGAGCCGTACCCGCCCTCGGGGTAAAAACAGACGCAATCCCTGGACGACTAAATCAAACCTCCTTCATCACCACTCGACCAGGAGTGTTTTACG
A-G_gallus : CGCATTGTAATCCCCATAGAATCCCCCATTCGAGTAATCATCACCGCTGATGACGTCCTCCACTCATGAGCCGTACCCGCCCTCGGGGTAAAAACAGACGCAATCCCTGGACGACTAAATCAAACCTCCTTCATCACCACTCGACCAGGAGTGTTTTACG
 CGAGTTGTMCTCCCAATAGAAATAACAATCCGAATACTAATCTCYTCCGAAGACGTACTACACTCATGAGCCGTCCCATCCCTAGGACTAAAAACAGACGCAATCCCAGGACGACTAAATCAAACAACCCTTATATCCACMCGACCAGGACTATTCTACG

 * 9300 * 9320 * 9340 * 9360 * 9380 * 9400 * 9420 * 9440
R-E_caballus : GTCAATGCTCAGAGATCTGCGGATCAAACCACAGCTTTATACCAATTGTCCTTGAACTAGTTCCACTGAAACACTTCGAAGAATGATCTGCATCAATATTA-TAA--------------AGTCACTAAGAAGCTATT--ATAGCATTAACCTTTTAAGTT
A-E_caballus : GTCAATGCTCAGAGATCTGCGGATCAAACCACAGCTTTATGCCAATTGTCCTTGAACTAGTTCCACTGAAACACTTCGAAGAATGATCTGCATCAATATTA-TAA--------------AGTCACTAAGAAGCTATT--ATAGCATTAACCTTTTAAGTT
R-C_bactrianus : GTCAATGTTCAGAAATTTGTGGCTCAAACCATAGCTTTATGCCCATTGTCCTTGAGATAGTACCACTAAAATACTTTGAGGAGTGATCTGCCTCTATATTA-TAA--------------GCTCACTAAGAAGCTAG---CCAGCGTTAACCTTTTAAGTT
A-C_bactrianus : GTCAATGTTCAGAAATTTGTGGCTCAAACCATAGCTTTATGCCCATTGTCCTTGAGATAGTACCACTAAAATACTTTGAGGAGTGATCTGCCTCTATATTA-TAA--------------GCTCACTAAGAAGCTAG---CCAGCGTTAACCTTTTAAGTT
R-C_lupus_familiaris : GCCAGTGCTCTGAAATCTGCGGATCTAACCACAGCTTTATACCCATTGTTCTTGAAATAGTCCCCCTATCTTACTTTGAGACCTGATCTGCCTTAATAGTA-TAACCTAGACTAGATCTACTCATTAAGAAGCTAT---AAAGCATTAACCTTTTAAGTT
A-C_lupus_familiaris : GCCAGTGCTCTGAAATCTGCGGATCTAACCACAGCTTTATACCCATTGTTCTTGAAATAGTCCCCCTATCTTACTTTGAGACCTGATCTGCCTTAATAGTA-TAACCTAGACTAGATCTACTCATTAAGAAGCTAT---AAAGCATTAACCTTTTAAGTT
R-N_procyonoides : GCCAATGCTCTGAAATTTGCGGATCTAACCACAGCTTCATGCCCATCGTTCTTGAAATAGTCCCCCTATCTTATTTCGAAACCTGATCTGCCCTTATAGTA-TAACATAGACTAGCGTAACTCATTGAGAAGCTAT---AAAGCGTTAACCTTTTAAGTT
A-N_procyonoides : GCCAATGCTCTGAAATTTGCGGATCTAACCACAGCTTCATGCCCATCGTTCTTGAAATAGTCCCCCTATCTTATTTCGAAACCTGATCTGCCCTTATAGTA-TAACATAGACTAGCGTAACTCATTGAGAAGCTAT---AAAGCGTTAACCTTTTAAGTT
R-V_lagopus : GCCAATGCTCTGAAATTTGCGGATCTAACCACAGCTTCATACCAATTGTTCTTGAAATAGTCCCACTGTCCTACTTCGAAACCTGATCTGCCTTAATGGTT-TAATATAGACAAGACCTATTCATTGAGAAGCTAT---AAAGCGTTAACCTTTTAAGTT
A-V_lagopus : GCCAATGCTCTGAAATTTGCGGATCTAACCACAGCTTCATACCAATTGTTCTTGAAATAGTCCCACTGTCCTACTTCGAAACCTGATCTGCCTTAATGGTT-TAATATAGACAAGACCTATTCATTGAGAAGCTAT---AAAGCGTTAACCTTTTAAGTT
R-M_putorius : GCCAATGCTCTGAAATCTGCGGCTCTAATCACAGCTTCATACCTATTGTCCTTGAGCTAGTACCTTTATCATACTTCGAAAAATGATCTGCCTCAATACTA-TAA--------------ATTCACCGAGAAGCTAA---ATAGCATTAACCTTTTAAGTT
A-M_putorius : GCCAATGCTCTGAAATCTGCGGCTCTAATCACAGCTTCATACCTATTGTCCTTGAGCTAGTACCTTTATCATACTTCGAAAAATGATCTGCCTCAATACTA-TAA--------------ATTCACCGAGAAGCTAA---ATAGCATTAACCTTTTAAGTT
R-B_taurus : GTCAATGCTCAGAAATTTGCGGGTCAAACCACAGTTTCATGCCCATTGTCCTTGAGTTAGTCCCACTAAAGTACTTTGAAAAATGATCTGCGTCAATATTA-TAA--------------AATCACTAAGAAGCTATA--T-AGCACTAACCTTTTAAGTT
A-B_taurus : GTCAATGCTCAGAAATTTGCGGGTCAAACCACAGTTTCATACCCATTGTCCTTGAGTTAGTCCCACTAAAGTACTTTGAAAAATGATCTGCGTCAATATTA-TAA--------------AATCACTAAGAAGCTATA--T-AGCACTAACCTTTTAAGTT
R-O_aries : GTCAATGCTCAGAAATTTGCGGATCAAATCACAGTTTTATGCCAATTGTTCTTGAACTAGTCCCATTAAAATACTTTGAAAAATGATCCGCATCAATACTA-TAA--------------AATCATCAAGAAGCTATC--CCAGCGTTAACCTTTTAAGTT
A-O_aries : GTCAATGCTCAGAAATTTGCGGATCAAATCACAGTTTTATGCCAATTGTTCTTGAACTAGTCCCATTAAAATACTTTGAAAAATGATCCGCATCAATACTA-TAA--------------AATCATCAAGAAGCTAGC--CCAGCGTTAACCTTTTAAGTT
R-S_scrofa : GACAGTGCTCAGAAATCTGTGGATCAAACCACAGCTTCATGCCCATTGTACTTGAACTTGTCCCATTAAAGTACTTCGAAAAATGGTCAACATCAATATTAACAG--------------GTTCATTGAGAAGCTAGT--C-AGCACTAACCTTTTAAGTT
A-S_scrofa : GACAGTGCTCAGAAATCTGTGGATCAAACCACAGCTTCATGCCCATTGTACTTGAACTTGTCCCATTAAAGTACTTCGAAAAATGGTCAACATCAATATTAACAG--------------GTTCATTGAGAAGCTAGT--C-AGCACTAACCTTTTAAGTT
R-O_cuniculus : GTCAGTGCTCAGAAATTTGTGGCTCAAACCATAGCTTTATACCTATTGTCCTCGAAATAGTTCCACTTAAACACTTCGAAAACTGATCCCTATCTATGATT-TAG--------------ACTCGCTATGAAGCTAA---ACAGCGCTAGCCTTTTAAGCT
A-O_cuniculus : GTCAGTGCTCAGAAATTTGTGGCTCAAACCATAGCTTTATACCTATTGTCCTCGAAATAGTTCCACTTAAACACTTCGAAAACTGATCCCTATCTATGATT-TAG--------------ACTCGCTATGAAGCTAA---ACAGCGCTAGCCTTTTAAGCT
R-M_musculus : GCCAATGCTCTGAAATTTGTGGATCTAACCATAGCTTTATGCCCATTGTCCTAGAAATGGTTCCACTAAAATATTTCGAAAACTGATCTGCTTCAATAATT-TAA--------------TTTCACTATGAAGCTAA---G-AGCGTTAACCTTTTAAGTT
A-M_musculus : GCCAATGCTCTGAAATTTGTGGATCTAACCATAGCTTTATGCCCATTGTCCTAGAAATGGTTCCACTAAAATATTTCGAAAACTGATCTGCTTCAATAATT-TAA--------------TTTCACTATGAAGCTAA---G-AGCGTTAACCTTTTAAGTT
R-R_norvegicus : GCCAATGCTCTGAAATTTGCGGCTCAAATCACAGCTTCATACCCATTGTACTAGAAATAGTCCCTCTAAAATATTTCGAAAACTGATCAGCTTCTATAATT-TAA--------------ACTCATTGCGAAGCTTA---G-AGCGTTAACCTTTTAAGTT
A-R_norvegicus : GCCAATGCTCTGAAATTTGCGGCTCAAATCACAGCTTCATACCCATTGTACTAGAAATAGTGCCTCTAAAATATTTCGAAAACTGATCAGCTTCTATAATT-TAA--------------ACTCATTGCGAAGCTTA---G-AGCGTTAACCTTTTAAGTT
R-M_coypus : GACAATGCTCAGAAATCTGTGGTTCTAACCATAGTTTTATACCTATTGTTATTGAAGTAGTTACTTTAAAAGCATTTGAAAACTGATGTTCATCTATATTA-TAA--------------GC-CGTTATGAAGCTAA---GTAGCATTAACCTTTTAAGTT
A-M_coypus : GACAATGCTCAGAAATCTGTGGTTCTAACCATAGTTTTATACCTATTGTTATTGAAGTAGTTACTTTAAAAGCATTTGAAAACTGATGTTCATCTATATTA-TAA--------------GC-CGTTATGAAGCTAA---GTAGCATTAACCTTTTAAGTT
R-A_platyrhynchos : GCCAGTGCTCAGAAATCTGCGGGGCTAACCACAGCTACATGCCTATTGTAGTAGAATCTACCCCACTCCCATACTTTGAAGCCTGATCATCCCTCCTATCGTCATC-----------CTAATCATTAAGAAGCTATGCAACAGCACTAGCCTTTTAAGCT
A-A_platyrhynchos : GCCAGTGCTCAGAAATCTGCGGGGCTAACCACAGCTACATGCCTATTGTAGTAGAATCTACCCCACTCCCATACTTTGAAGCCTGATCATCCCTCCTATCGTCATC-----------CTAATCATTAAGAAGCTATGCAACAGCACTAGCCTTTTAAGCT
R-G_gallus : GACAATGCTCAGAAATCTGCGGAGCTAACCACAGCTACATACCCATTGTAGTAGAGTCTACCCCCCTAAAACACTTTGAAGCCTGA---TCCTCACTACTGTCATC-----------TTAACCATTAAGAAGCTATGCACCAGCACTAGCCTTTTAAGCT
A-G_gallus : GACAATGCTCAGAAATCTGCGGAGCTAACCACAGCTACATACCCATTGTAGTAGAGTCTACCCCCCTAAAACACTTTGAAGCCTGA---TCCTCACTACTGTCATC-----------TTAACCATTAAGAAGCTATGCACCAGCACTAGCCTTTTAAGCT
 GCCAATGCTCAGAAATTTGCGGATCTAACCACAGCTTCATACCCATTGTCCTTGAAATAGTCCCACTAAAATACTTCGAAAACTGATCTGCCTCAATATTATTAACATAGACTAGABCTACTCATTAAGAAGCTATKCAACAGCATTAACCTTTTAAGTT

 * 9460 * 9480 * 9500 * 9520 * 9540 * 9560 * 9580 * 9600
R-E_caballus : AA--AGATTGAGGGTTC--AACCCCCTCCCTAGTGATATGCCACAGTTGGATACATCAACATGATTTATTAATATCGTCTCAATAATCCTAACTCTATTTATTGTATTTCAACTAAAAATCTCAAAGC---ACTCCTATCCGACACACCCAGAAGTAAAG
A-E_caballus : AA--AGATTGAGGGTTC--AACCCCCTCCCTAGTGATATGCCACAGTTGGATACATCAACATGATTTATTAATATCGTCTCAATAATCCTAACTCTATTTATTGTATTTCAACTAAAAATCTCAAAGC---ACTCCTACCCGACACACCCAGAAGTAAAG
R-C_bactrianus : AA--AGAACGAGAGCCA--TGATCCCTCCTTAGTGACATGCCACAGCTGGATACATCAACATGATTTATTACCATTCTATCTATACTTATAACCCTCTTTGTACTATTTCAGCTAAAAATTTCCAAGC---ATATATACCTCTCAGCCCCCAGCCCTAAA
A-C_bactrianus : AA--AGAACGAGAGCCA--TGATCCCTCCTTAGTGACATGCCACAGCTGGATACATCAACATGATTTATTACCATTCTATCTATACTTATAACCCTCTTTGTACTATTTCAGCTAAAAATTTCCAAGC---ATATATACCTCTCAGCCCCCAGCCCTAAA
R-C_lupus_familiaris : AA--AGACTGGGAGTTT--TAACCTCTCCTTAATGAAATGCCACAGCTAGATACATCCACCTGATTTATTATAATCTTTTCAATATTTCTCACCCTCTTCATCCTATTTCAACTAAAAATTTCAAATC---ACTACTACCCAGAAAACCCGATAACCAAA
A-C_lupus_familiaris : AA--AGACTGGGAGTTT--TAACCTCTCCTTAATGAAATGCCACAGCTAGATACATCCACCTGATTTATTATAATCTTTTCAATATTTCTCACCCTCTTCATCCTATTTCAACTAAAAATTTCAAATC---ACTACTACCCAGAAAACCCGATAACCAAA
R-N_procyonoides : AA--AGACTGAGAGTCC--TACCCTCTCCTTAATGAAATGCCACAATTAGATACATCCACTTGATTTACTATAATTATCTCAATAATCCTAACTTTATTTATTCTGTTCCAACTAAAAATTTCAATGC---ACTATTACCCAGAAAACCCAGGACCTAAG
A-N_procyonoides : AA--AGACTGAGAGTCC--TACCCTCTCCTTAATGAAATGCCACAATTAGATACATCCACTTGATTTACTATAATTATCTCAATAATCCTAACTTTATTTATTCTGTTCCAACTAAAAATTTCAATGC---ACTATTACCCAGAAAACCCAGGACCTAAG
R-V_lagopus : AA--AGACTGAGAGTTT--TAACCTCTCCTTAATGAAATGCCACAGCTAGACACATCTACTTGATTCACCATAATTCTCTCAATAACCCTAACCCTGTTTATCTTATTTCAACTAAAAGTATCAAAAC---ACTACTACCCGGAGAATCCAGGACCTAAG
A-V_lagopus : AA--AGACTGAGAGTTT--TAACCTCTCCTTAATGAAATGCCACAGCTAGACACATCTACTTGATTCACCATAATTCTCTCAATAACCCTAACCCTGTTTATCTTATTTCAACTAAAAGTATCAAAAC---ACTACTACCCGGAGAATCCAGGACCTAAG
R-M_putorius : AA--AGATTGAGAGCAT--AAATCTCTCCTCAGTGATATGCCACAATTAGACACTTCAACATGATTTATCACTATTTTATCAATAATTGTAACCCTATTTTTTATATTTCAACTAAAACTATCAAAAT---ACAACTTTCCAGAAAACCCTGAACCAAAA
A-M_putorius : AA--AGATTGAGAGCAT--AAATCTCTCCTCAGTGATATGCCACAATTAGACACTTCAACATGATTTATCACTATTTTATCAATAATTGTAACCCTATTTTTTATATTTCAACTAAAACTATCAAAAT---ACAACTTTCCAGAAAACCCTGAACCAAAA
R-B_taurus : AG--AGATTGAGAGCCA--TATACTCTCCTTGGTGACATGCCGCAACTAGACACGTCAACATGACTGACAATGATCTTATCAATATTCTTGACCCTTTTTATCATCTTTCAACTAAAAGTTTCAAAACACAACTTTTATC--ACAA-TCCAGAACTGACA
A-B_taurus : AG--AGATTGAGAGCCA--TATACTCTCCTTGGTGACATGCCGCAACTAGACACGTCAACATGACTGACAATGATCTTATCAATATTCTTGACCCTTTTTATCATCTTTCAACTAAAAGTTTCAAAACACAACTTTTATC--ACAA-TCCAGAACTGACA
R-O_aries : AA--AGACTGAGAATAT--TATATTCTCCTTGATGATATGCCACAACTAGACACATCAACGTGACTTACAATAATTCTATCAATATTTTTAGTCCTCTTCATTATTTTTCAACTAAAAATCTCAAAACACAACTTCTACC--ACAA-CCCAGAATTAATA
A-O_aries : AA--AGACTGAGAATAT--TATATTCTCCTTGATGATATGCCACAACTAGACACATCAACGTGACTTACAATAATTCTATCAATATTTTTAGTCCTCTTCATTATTTTTCAACTAAAAATCTCAAAACACAACTTCTACC--ACAA-CCCAGAATTAATA
R-S_scrofa : AG--AGATCGGGAGCCT--AAATCTCCCCTCAATGGTATGCCACAACTAGATACATCTACATGATTCATTACAATTACATCAATAATTATAACATTATTTATTTTATTCCAACTAAAAATCTCAAA---CTACTCATACCCAGCAAGCCCAGAATCAACC
A-S_scrofa : AG--AGATCGGGAGCCT--AAATCTCCCCTCAATGGTATGCCACAACTAGATACATCTACATGATTCATTACAATTACATCAATAATTATAACATTATTTATTTTATTCCAACTAAAAATCTCAAA---CTACTCATACCCAGCAAGCCCAGAATCAACC
R-O_cuniculus : AG--AGAGTGAGAGTTAAATAGTCTCTCCATAGTGAAATGCCACAACTTGACACATCCACATGATTTACTACCATTGTCGCCATAATTCTTTCACTATTTATCCTAATACAACTCAAATTCCACAAAT---ACACATACCCTATGAACCCAGTACTAAAA
A-O_cuniculus : AG--AGAGTGAGAGTTAAATGGTCTCTCCATAGTGAAATGCCACAACTTGACACATCCACATGATTTACTACCATTGTCGCCATAATTCTTTCACTATTTATCCTAATACAACTCAAATTCCACAAAT---ACACATACCCTATGAACCCAGTACTAAAA
R-M_musculus : AA--AGTTAGAGACCTT---AAAATCTCCATAGTGATATGCCACAACTAGATACATCAACATGATTTATCACAATTATCTCATCAATAATTACCCTATTTATCTTATTTCAACTAAAAGTCTCATCAC---AAACATTCCCACTGGCACCTTCACCAAAA
A-M_musculus : AA--AGTTAGAGACCTT---AAAATCTCCATAGTGATATGCCACAACTAGATACATCAACATGATTTATCACAATTATCTCATCAATAATTACCCTATTTATCTTATTTCAACTAAAAGTCTCATCAC---AAACATTCCCACTGGCACCTTCACCAAAA
R-R_norvegicus : AA--AGTTAGAGACAAC---AAA-TCTCCACAATGACATGCCACAACTAGACACATCCACATGATTTATTACAATCATCTCCTCAATAGCCACACTATTTATTTTATTTCAATTAAAAATTTCTTCCC---AAACCTTTCCTGCACCTCCCTCCCCCAAA
A-R_norvegicus : AA--AGTTAGAGACAAC---AAA-TCTCCACAATGACATGCCACAACTAGACACATCCACATGATTTATTACAATCATCTCCTCAATAGCCACACTATTTATTTTATTTCAATTAAAAATTTCTTCCC---AAACCTTTCCTGCACCTCCCTCACCCAAA
R-M_coypus : AA--AGATCGAGATTT-----AATTCTCCATAACGAAATGCCACAACTAGACACATCCACATGATTTACAGTAATTTCCTCTATAATAATCACGCTATTTATTATCTTTCAACTAAAAATCTTAACTC---ACCAGATCCTTATCAAACCTCAAACAACC
A-M_coypus : AA--AGATCGAGATTT-----AATTCTCCATAACGAAATGCCACAACTAGACACATCCACATGATTTACAGTAATTTCCTCTATAATAATCACGCTATTTATTATCTTTCAACTAAAAATCTTAACTC---ACCAGATCCTTATCAAACCTCAAACAACC
R-A_platyrhynchos : AGCTAAAGAGGAATTAT----CC-CCTCCTTAATGGCATGCCTCAACTCAACCCTGCACCATGATTCTCAATCATAGTCATAACCTGACTAACCCTCGCACTCCTAATCCAGCCAAAACTGCTAACCT------TCACCACAACAAATCCCCCATCAAAA
A-A_platyrhynchos : AGCTAAAGAGGAATTAT----CC-CCTCCTTAATGGCATGCCTCAACTCAACCCTGCACCATGATTCTCAATCATAGTCATAACCTGACTAACCCTCGCACTCCTAATCCAGCCAAAACTGCTAACCT------TCACCACAACAAATCCCCCATCAAAA
R-G_gallus : AG--AGAGAGGGGACAC----CCTCCCCCTTAATGACATGCCCCAATTAAACCCAAACCCATGATTCTCCATCATACTCCTAACTTGATTCACCTTCTCTCTGCTTATCCAACCCAAACTTCTTTCAT------TCACTCTAACAAACAACCCTGCAAAC
A-G_gallus : AG--AGAGAGGGGACAC----CCTCCCCCTTAATGACATGCCCCAATTAAACCCAAACCCATGATTCTCCATCATACTCCTAACTTGATTCACCTTCTCTCTGCTTATCCAACCCAAACTTCTTTCAT------TCACTCTAACAAACAACCCTGCAAAC
 AACTAGATTGAGAGTTTAATAACCTCTCCTTAATGAHATGCCACAACTAGACACATCAACATGATTTACTATAATTHTCTCAATAATTCTAACCCTATTTATTCTATTTCAACTAAAAATYTCAAAACACAACTHCTACCCAACAAACCCAGAACCAAAA

 * 9620 * 9640 * 9660 * 9680 * 9700 * 9720 * 9740 * 9760
R-E_caballus : ACAA---CCAAAAT-AACAAAACACTCTGCCCCTTGAGAATCAAAATGAACGAAAATCTATTCGCCTCTTTCGCTA-CCCCAACAATAGTAGGCCTCCCTATTGTAATTCTGATCATCATATTTCCCAGCATCCTATTCCCCTCACCC---AACCGACTA
A-E_caballus : ACAA---CCAAAAT-AACAAAACACTCTGCCCCTTGAGAATCAAAATGAACGAAAATCTATTCGCCTCTTTCGCTA-CCCCAACAATAATAGGCCTCCCTATTGTAATTCTGATCATCATATTTCCCAGCATCCTATTCCCTTCACCC---AACCGACTA
R-C_bactrianus : TCCA---ATAAAAC-ACGCAAACAAAAGACCCCTTGAGAAACAAAATGAACGAAAATTTATTTGCCTCTTTCATTA-CCCCAACAGTAATAGGACTTCCTATTGTAATCCTCATTATTATGTTCCCAAGCATACTATTCCCGGCCCCA---TTTCGACTA
A-C_bactrianus : TCCA---ATAAAAC-ACGCAAACAAAAGACCCCTTGAGAAACAAAATGAACGAAAATTTATTTGCCTCTTTCATTA-CCCCAACAGTAATAGGACTTCCTATTGTAATCCTCATTATTATATTCCCAAGCATACTATTCCCGGCCCCA---TTTCGACTA
R-C_lupus_familiaris : TCTG---CTAAAAT-TGCTGGTCAACATAATCCTTGAGAAAACAAATGAACGAAAATCTATTCGCTTCTTTCGCTG-CCCCCTCAATAATAGGTCTCCCTATTGTGGTACTGATCGTCATATTCCCTTCCATTTTATTCCCAACACCC---AGTCGCCTA
A-C_lupus_familiaris : TCTG---CTAAAAT-TGCTGGTCAACATAATCCTTGAGAAAACAAATGAACGAAAATCTATTCGCTTCTTTCGCTG-CCCCCTCAATAATAGGTCTCCCTATTGTGGTACTGATCGTCATATTCCCTTCCATTTTATTCCCAACACCC---AGTCGCCTA
R-N_procyonoides : CCCA---CCAAAAT-AATTAATCAACACACCCCTTGAGAAAACAAATGAACGAAAATTTATTCGCCTCTTTCACTA-CCCCCTCAATAATAGGTCTTCCAATCGCAGTACTTATTGTCATATTCCCATCTATTCTATTCCCCTCACCT---GGCCGCCTA
A-N_procyonoides : CCCA---CCAAAAT-AATTAATCAACACACCCCTTGAGAAAACAAATGAACGAAAATTTATTCGCCTCTTTCACTA-CCCCCTCAATAATAGGTCTTCCAATCGCAGTACTTATTGTCATATTCCCATCTATTCTATTCCCCTCACCT---GGCCGCCTA
R-V_lagopus : TCCG---TTAAATC-TACTAGCAAACACATCCCTTGAGAAGATAAATGAACGAAAATCTATTCGCCTCTTTCGCTA-CCCCCACAATAATGGGCCTACCAATCGCTGTATTAATTGTAATATTTCCATCTATTCTATTCCCATCACCT---AACCGACTA
A-V_lagopus : TCCG---TTAAATC-TACTAGCAAACACATCCCTTGAGAAGATAAATGAACGAAAATCTATTCGCCTCTTTCGCTA-CCCCCACAATAATGGGCCTACCAATCGCTGTATTAATTGTAATATTTCCATCTATTCTATTCCCATCACCT---AACCGACTA
R-M_putorius : TTAGTGGCTACATC-AA--AATCTAC-TACACCTTGAGAAAAGAAATGAACGAAAATCTATTTTCCTCATTCACTA-CCCCTACAATAATAGGATTGCCTATCGTCATCCTCATCACCATATTCCCAAGTATTATATTCCCCTCACCC---AACCGACTG
A-M_putorius : TTAGTGGCTACATC-AA--AATCTAC-TACACCTTGAGAAAAGAAATGAACGAAAATCTATTTTCCTCATTCACTA-CCCCTACAATAATAGGATTGCCTATCGTCATCCTCATCACCATATTCCCAAGTATTATATTCCCCTCACCC---AACCGACTG
R-B_taurus : CCAA---CAAAAAT-ATTAAAACAAAACACCCCTTGAGAAACAAAATGAACGAAAATTTATTTACCTCTTTTATTA-CCCCTGTAATTTTAGGTCTCCCTCTCGTAACCCTTATCGTACTATTCCCAAGCCTACTATTCCCAACATCA---AACCGACTA
A-B_taurus : CCAA---CAAAAAT-ATTAAAACAAAACACCCCTTGAGAAACAAAATGAACGAAAATTTATTTACCTCTTTTATTA-CCCCTGTAATTTTAGGTCTCCCTCTCGTAACCCTTATCGTACTATTCCCAAGCCTACTATTCCCAACATCA---AACCGACTA
R-O_aries : ACAA---CAAAAAC-ACCGAAACAAAATACTCCTTGAGAAACAAAATGAACGAAAATCTATTTGCCTCTTTCATTA-CCCCTATAATATTTGGTCTCCCCCTCGTTACCCTCATTGTTTTATTCCCTAGCCTATTATTTCCCACATCA---AACCGACTA
A-O_aries : ACAA---CAAAAAC-ACCGAAACAAAATACTCCTTGAGAAACAAAATGAACGAAAATCTATTTGCCTCTTTCATTA-CCCCTATAATATTTGGCCTCCCCCTCATTACCCTCATTGTTTTATTCCCTAGCCTATTATTTCCCACATCA---AGCCGACTA
R-S_scrofa : GAAC---TCAAAAC-TCAAAAACATAGCACCCCTTGAGAAATAAAATGAACGAAAATCTATTTGCCTCTTTCATTG-CCCCTACGATAATAGGACTACCTATTGCCACCTTAATTATTATATTCCCAAGCTTACTATTCCCAACACCC---AAACGACTC
A-S_scrofa : GAAC---TCAAAAC-TCAAAAACATAGCACCCCTTGAGAAATAAAATGAACGAAAATCTATTTGCCTCTTTCATTG-CCCCTACGATAATAGGACTACCTATTGTCACCTTAATTATTATATTCCCAAGCTTACTATTCCCAACACCC---AAACGACTC
R-O_cuniculus : GCAC---TTGAGTC-TACTTCATTCCCTTGCCCATGAGAAACAAAATGAACGAAAATTTATTCTCCTCTTTCGCTA-CCCCAACACTAATAGGGCTCCCTATTGTAGCCTTAATTATCATATTCCCAACTTTACTATTTCCCTCCCCT---AGCCGACTA
A-O_cuniculus : TCAC---TTGAGTT-TACTTCATTCCCTTGCCCATGAGAAACAAAATGAACGAAAATTTATTCTCCTCTTTCGCTA-CCCCAACACTAATAGGGCTCCCTATTGTAGCCTTAATTATCATATTCCCAACTTTACTATTTCCCTCCCCT---AGCCGACTA
R-M_musculus : TCAC---TAACAACCATAAAAGTA-AAAACCCCTTGAGAATTAAAATGAACGAAAATCTATTTGCCTCATTCATTA-CCCCAACAATAATAGGATTCCCAATCGTTGTAGCCATCATTATATTTCCTTCAATCCTATTCCCATCCTCA---AAACGCCTA
A-M_musculus : TCAC---TAACAACCATAAAAGTA-AAAACCCCTTGAGAATTAAAATGAACGAAAATCTATTTGCCTCATTCATTA-CCCCAACAATAATAGGATTCCCAATCGTTGTAGCCATCATTATATTTCCTTCAATCCTATTCCCATCCTCA---AAACGCCTA
R-R_norvegicus : ACTA---TAGCCACAGAAAAAACG-AATAACCCTTGAGAATCAAAATGAACGAAAACCTATTTGCCTCTTTCATTA-CCCCCACAATAATAGGTCTACCAATTGTTGTAACCATTATTATGTTCCCATCAATTCTATTCCCATCATCA---GAACGCCTA
A-R_norvegicus : ACTA---TAGCTACAGAAAAAACG-AATAACCCTTGAGAATCAAAATGAACGAAAATCTATTTGCCTCTTTCATTA-CCCCCACAATAATAGGTCTACCAATTGTTGTAACCATTATTATGTTCCCATCAATTCTATTCCCATCATCA---AAACGCCTA
R-M_coypus : TTTC---TAG-AGCAAACAAAATATAATACGCCTTGAGAAGAAAAATGAACGAAAACTTATTTACCCCTTTCATAA-CACCAACACTAATAGGTATACCTATTGTTACTTTTATCATTTTATTCCCAACTATCTTATACCCTAATCCA---AGCCGACTG
A-M_coypus : TTTC---TAG-AGCAAACAAAATATAATACGCCTTGAGAAGAAAAATGAACGAAAACTTATTTACCCCTTTCATAA-CACCAACACTAATAGGTATACCTATTGTTACTTTTATCATTTTATTCCCAACTATCTTATACCCTAATCCA---AGCCGACTG
R-A_platyrhynchos : AAAC----CATCACTCATCACCAAACCCACACCATGAGCCTGACCATGAACCTAAGTTTCTTTGACCAATTCTCAAGCCCCCAC-CTACTTGGCATCCCCCTGATCCTACTATCCCTGCTCTTCCCAGCCCTATTGTTCCCATCCCCAGGCAACCGATGA
A-A_platyrhynchos : AAAC----CATCACTCATCACCAAACCCACACCATGAGCCTGACCATGAACCTAAGTTTCTTTGACCAATTCTCAAGCCCCCAC-CTACTTGGCATCCCCCTGATCCTACTATCCCTGCTCTTCCCAGCCCTATTGTTCCCATCCCCAGGCAACCGATGA
R-G_gallus : AAA------ATTACA-ACAACTAAACCCACCCCCTGAACCTGACCATGAACCTAAGCTTCTTCGACCAATTCTCAAGCCCCTGC-CTACTAGGAATCCCTCTAATCCTCCCATCACTCCTTCTTCCAGCCCTCCTACTTCCATCACCAGGAAACCGATGG
A-G_gallus : AAA------ATTACA-ACAACTAAACCCACCCCCTGAACCTGACCATGAACCTAAGCTTCTTCGACCAATTCTCAAGCCCCTGC-CTACTAGGAATCCCTCTAATCCTCCCATCACTCCTTCTTCCAGCCCTCCTACTTCCATCACCAGGAAACCGATGG
 TCAATGGYWAAAACAAACAAAACAAMATACCCCTTGAGAAACAAAATGAACGAAAATCTATTTGCCTCTTTCACTAGCCCCHACAATAATAGGTCTCCCTATTGTWATCCTMATYATYATATTCCCAACCATACTATTCCCATCACCAGGMAACCGACTA

 * 9780 * 9800 * 9820 * 9840 * 9860 * 9880 * 9900 * 9920
R-E_caballus : ATCAACAATCGCCTAATCTCAATTCAACAATGGCTAGTCCAACTTA-CATCAAAACAAATAATAGCTATCCATAACAGCAAAGGACAAACCTGAACTCTTATACTCATATCACTGATCCTATTCATTGGCTCAACAAACTTATTAGGCCTACTACCTCAC
A-E_caballus : ATCAACAATCGCCTAATCTCAATTCAACAATGGCTAGTCCAACTTA-CATCAAAACAAATAATAGCTATCCATAACAGCAAAGGACAAACCTGAACTCTTATACTCATATCACTGATCCTATTCATTGGCTCAACAAACTTATTAGGCCTACTACCTCAC
R-C_bactrianus : ATTAACAACCGTCTAATCTCTCTACAATATTGATTAATCCGACTCA-CATCTAAACAAATGATGACTATCCACAACCACAAGGGACAAACCTGATCCTTGATGCTAATATCTCTAATCATGTTTATCGGGACTACCAATCTCCTAGGACTCCTCCCACAT
A-C_bactrianus : ATTAACAACCGTCTAATCTCTCTACAATATTGATTAATCCGACTCA-CATCTAAACAAATGATGACTATCCATAACCACAAGGGACAAACCTGATCCTTGATGCTAATATCTCTAATCATGTTTATCGGGACTACCAATCTCCTAGGACTCCTCCCACAT
R-C_lupus_familiaris : ATCAATAATCGGTTAATCTCCATTCAGCAATGACTAATTCAACTAA-CATCAAAACAAATACTAGCAATTCATAACCAAAAGGGACGAACCTGAGCTCTCATACTTATATCACTAATTCTATTTATTGGCTCAACTAATCTACTTGGACTATTACCTCAC
A-C_lupus_familiaris : ATCAATAATCGGTTAATCTCCATTCAGCAATGACTAATTCAACTAA-CATCAAAACAAATACTAGCAATTCATAACCAAAAGGGACGAACCTGAGCTCTCATACTTATATCACTAATTCTATTTATTGGCTCAACTAATCTACTTGGACTATTACCTCAC
R-N_procyonoides : ATTAACAACCGCCTAATCTCCATTCAACAATGACTCATTCAATTAG-TATCCAAACAAATATTATCAATCCACAACCAAAAAGGACGAACTTGAGCTCTTATGTTAGTATCATTAATCCTATTTATTGCCTCGACCAATCTACTCGGACTGTTACCCCAC
A-N_procyonoides : ATTAACAACCGCCTAATCTCCATTCAACAATGACTCATTCAATTAG-TATCCAAACAAATATTATCAATCCACAACCAAAAAGGACGAACTTGAGCTCTTATGTTAGTATCATTAATCCTATTTATTGCCTCGACCAATCTACTCGGACTGTTACCCCAC
R-V_lagopus : ATTAATAATCGACTAATCTCTATTCAACAGTGATTAATTCAACTTA-CATCTAAACAAATACTAATAATCCACAATCAAAAAGGACGAACCTGGGCCCTTATGCTAATATCACTAATTATATTTATTGGCTCAACCAATCTCCTCGGGCTATTACCCCAC
A-V_lagopus : ATTAATAATCGACTAATCTCTATTCAACAGTGATTAATTCAACTTA-CATCTAAACAAATACTAATAATCCACAATCAAAAAGGACGAACCTGGGCCCTTATGCTAATATCACTAATTATATTTATTGGCTCAACCAATCTCCTCGGGCTATTACCCCAC
R-M_putorius : ATTAACAACCGACTCATTTCTATCCAACAATGATTGGTTCAATTAA-CATCAAAACAAATGCTGTCCATTCACAACCAAAAAGGACAAACTTGAGCATTAATACTAATATCCCTAATCCTATTTATTGGGTCTACTAACCTGCTAGGTCTCTTACCTCAC
A-M_putorius : ATTAACAACCGACTCATTTCTATCCAACAATGATTGGTTCAATTAA-CATCAAAACAAATGCTGTCCATTCACAACCAAAAAGGACAAACTTGAGCATTAATACTAATATCCCTAATCCTATTTATTGGGTCTACTAACCTGCTAGGTCTCTTACCTCAC
R-B_taurus : GTAAGCAATCGCTTTGTAACCCTCCAACAATGAATACTTCAACTTG-TATCAAAACAAATAATGAGTATCCACAATTCTAAAGGACAAACATGAACATTAATATTAATATCTCTGATCCTATTTATTGGATCAACAAACCTACTAGGCCTATTACCCCAT
A-B_taurus : GTAAGCAATCGCTTTGTAACCCTCCAACAATGAATACTTCAACTTG-TATCAAAACAAATAATGAGTATCCACAATTCTAAAGGACAAACATGAACATTAATATTAATATCTCTGATCCTATTTATTGGATCAACAAACCTACTAGGCCTATTACCCCAT
R-O_aries : GTCAACAACCGCCTCATCTCCCTCCAACAGTGAATACTTCAATTAG-TATCAAAACAAATAATGAGCATTCATAATACCAAAGGACAGACATGAGCATTAATGCTAATGTCCCTAATTTTATTTATTGGATCTACAAACCTACTAGGCCTCCTACCCCAC
A-O_aries : GTCAACAACCGCCTCATCTCCCTCCAACAGTGAATACTTCAATTAG-TATCAAAACAAATAATGAGCATTCATAACACCAAAGGACAGACATGAGCATTAATACTAATGTCCCTAATTTTATTTATTGGATCTACAAACCTACTAGGCCTCCTACCCCAC
R-S_scrofa : ATTAATAACCGCACAATCTCGATCCAACAATGATTAATCCAACTAA-CATCCAAACAAATAATGGCTATTCACAACCAAAAAGGCCAAACCTGATCACTAATACTTATATCTCTAATTATATTCATTGGCTCAACAAACATCCTAGGCCTACTACCACAC
A-S_scrofa : ATTAATAACCGCACAATCTCGATCCAACAATGATTAATCCAACTAA-CATCCAAACAAATAATGGCTATTCACAACCAAAAAGGCCAAACCTGATCACTAATACTTATATCTCTAATTATATTCATTGGCTCAACAAACATCCTAGGCCTACTACCACAC
R-O_cuniculus : ATTAACAACCGACTAGTCTCAACCCAACAATGATTAGCCCAACTTA-TCTTAAAGCAAATAATATTAATACATTCCCCCAAAGGACGAACCTGATCTCTAATACTAATTTCCCTAATCATATTTATTGGCTCAACCAACCTCCTAGGCCTCCTGCCTCAC
A-O_cuniculus : ATTAACAACCGACTAGTCTCAACCCAACAATGATTAGCCCAACTTA-TCTTAAAGCAAATAATATTAATACATTCCCCCAAAGGACGAACCTGATCTCTAATATTAATTTCCCTAATCATATTTATTGGCTCAACCAACCTCCTAGGCCTCCTGCCTCAC
R-M_musculus : ATCAACAACCGTCTCCATTCTTTCCAACACTGACTAGTTAAACTTATTATCAAA-CAAATAATGCTAATCCACACACCAAAAGGACGAACATGAACCCTAATAATTGTTTCCCTAATCATATTTATTGGATCAACAAATCTCCTAGGCCTTTTACCACAT
A-M_musculus : ATCAACAACCGTCTCCATTCTTTCCAACACTGACTAGTTAAACTTATTATCAAA-CAAATAATGCTAATCCACACACCAAAAGGACGAACATGAACCCTAATAATTGTTTCCCTAATCATATTTATTGGATCAACAAATCTCCTAGGCCTTTTACCACAT
R-R_norvegicus : ATCAGCAACCGACTACACTCATTTCAACACTGACTAATCAAACTTATCATCAAA-CAAATAATGTTAATCCACACACCAAAAGGACGAACCTGAGCCCTAATAATTGTATCCCTAATTATATTTATTGGCTCAACCAACCTTCTAGGGCTTCTTCCCCAT
A-R_norvegicus : ATCAGCAACCGACTACACTCATTTCAACACTGACTAATCAAACTTATCATCAAA-CAAATAATGTTAATCCACACACCAAAAGGACGAACCTGAGCCCTAATAATTGTATCCCTAATTATATTTATTGGCTCAACCAACCTTCTAGGGCTTCTTCCCCAT
R-M_coypus : ATTAATAACCGTATTATTACTATCCAGCAATGATTAATTAAA-TTAGTATTAAAACAAATAATACCAACCCATAACTCCAAAGGACGCTCCTGATCTCTTATGCTAGTTACACTAATCTTATTTATTGGAACAACCAATTTATTAGGGCTCCTTCCCCAC
A-M_coypus : ATTAATAACCGTATTATTACTATCCAGCAATGATTAATTAAA-TTAGTATTAAAGCAAATAATACCAATCCATAACTCCAAAGGACGCTCCTGATCTCTTATGCTAGTTACACTAATCTTATTTATTGGAACAACCAATTTATTAGGGCTCCTTCCCCAC
R-A_platyrhynchos : ATCAACAACCGACTATCCACCATCCAACTGTGACTCCTACACCTAATCA-CAAAACAACTAATAATCCCATTAAACAAAAACGGCCACAAATGAGCCCTGATGCTAACATCACTAATAACCATACTCCTAACAATCAACCTTCTAGGACTTCTCCCATAT
A-A_platyrhynchos : ATCAACAACCGACTATCCACCATCCAACTGTGACTCCTACACCTAATCA-CAAAACAACTAATAATCCCATTAAACAAAAACGGCCACAAATGAGCCCTGATGCTAACATCACTAATAACCATACTCCTAACAATCAACCTTCTAGGACTTCTCCCATAT
R-G_gallus : ATCAACAACCGCCTCTCCACCATCCAACTCTGATTCACCCACCTAATCA-CAAAACAACTAATAACCCCCCTAAACAAGGCAGGTCACAAATGAGCCCTCCTACTCACCTCACTTATCCTAATACTCCTCTCCATTAACCTCCTAGGCCTCCTCCCCTAC
A-G_gallus : ATCAACAACCGCCTCTCCACCATCCAACTCTGATTCACCCACCTAATCA-CAAAACAACTAATAACCCCCCTAAACAAGGCAGGTCACAAATGAGCCCTCCTACTCACCTCACTTATCCTAATACTCCTCTCCATTAACCTCCTAGGCCTCCTCCCCTAC
 ATYAACAACCGCCTAATCTCCATCCAACAATGATTAATTCAACTWATCATCAAAACAAATAATAACAATCCACAACCAAAAAGGACAAACCTGAGCCCTAATACTAATATCACTAATCATATTTATTGGCTCAACCAACCTMCTAGGCCTCCTACCCCAC

 * 9940 * 9960 * 9980 * 10000 * 10020 * 10040 * 10060 * 10080
R-E_caballus : TCATTTACACCAACAACACAACTATCAATAAACCTAGGCATAGCTATTCCCCTATGGGCAGGGACAGTATTCATAGGCTTTCGTCACAAAACAAAAGCAGCCCTAGCCCACTTTCTACCTCAAGGGACGCCCATTTTCCTCATCCCCATACTAGTAATTA
A-E_caballus : TCATTTACACCAACAACACAACTATCAATAAACCTAGGCATAGCTATTCCCCTATGGGCAGGGACAGTGTTCATAGGCTTTCGTCATAAAACAAAAGCAGCCCTAGCCCACTTTCTACCTCAAGGAACGCCCATTTTCCTCATCCCCATACTAGTAATTA
R-C_bactrianus : TCATTTACCCCTACCACACAACTATCGATAAATCTAGGAATAGCAATTCCTTTATGAGCCGGAACAGTGGTCACTGGTTTTCGCAATAAAACAAAGGCATCACTAGCACATTTCCTCCCCCAGGGAACACCTACACCTCTAATCCCAATACTAGTAATCA
A-C_bactrianus : TCATTTACCCCTACCACACAACTATCGATAAATCTAGGAATAGCAATTCCTTTATGAGCCGGAACAGTGGTCACTGGTTTTCGCAATAAAACAAAGGCATCACTAGCACATTTCCTCCCCCAGGGAACACCTACACCTCTAATCCCAATACTAGTAATCA
R-C_lupus_familiaris : TCATTTACGCCCACAACACAACTCTCTATAAACCTCGGAATAGCAATTCCCCTATGAGCAGGGACAGTAATTACCGGTTTCCGCTATAAAACCAAAGCATCCTTAGCACACTTTCTACCCCAAGGCACCCCTCTCCCCCTAATTCCAATACTAGTAGTCA
A-C_lupus_familiaris : TCATTTACGCCCACAACACAACTCTCTATAAACCTCGGAATAGCAATTCCCCTATGAGCAGGGACAGTAATTACCGGTTTCCGCTATAAAACCAAAGCATCCTTAGCACACTTTCTACCCCAAGGCACCCCTCTTCCCCTAATTCCAATACTAGTAGTCA
R-N_procyonoides : TCATTCACCCCTACAACTCAACTGTCCATGAATCTAGGAATAGCTATCCCCCTGTGAGCAGGAGCAGTAATTACTGGTTTTCGCTATAAAACTAAAGCATCCCTGGCACACTTCCTACCCCAAGGCACACCTATCCCCCTGATCCCAATACTAGTCATTA
A-N_procyonoides : TCATTCACCCCTACAACTCAACTGTCCATGAATCTAGGAATAGCTATCCCCCTGTGAGCAGGAGCAGTAATTACTGGTTTTCGCTATAAAACTAAAGCATCCCTGGCACACTTCCTACCCCAAGGCACACCTATCCCCCTGATCCCAATACTAGTCATTA
R-V_lagopus : TCGTTTACACCCACAACCCAGTTATCTATAAATCTAGGGATAGCAATTCCCCTGTGAGCAGGGACAGTAATTACCGGGTTCCGCCACAAAACTAAAGCTTCTTTAGCACATTTCTTACCTCAAGGCACACCCCTCCCCCTAATCCCCATGCTAGTAATTA
A-V_lagopus : TCGTTTACACCCACAACCCAGTTATCTATAAATCTAGGGATAGCAATTCCCCTGTGAGCAGGGACAGTAATTACCGGGTTCCGCCACAAAACTAAAGCTTCTTTAGCACATTTCTTACCTCAAGGCACACCCCTCCCCCTAATCCCCATGCTAGTAATTA
R-M_putorius : TCATTTACCCCTACCACACAACTGTCCTTGAACCTAGGAATAGCTATCCCCCTATGAGCAGGCACAGTAATTACTGGATTTCGACACAAAACAAAAGCCTCTTTAGCCCACTTTCTACCACAAGGAACCCCACTACCCCTAATCCCCATGCTCATTATCA
A-M_putorius : TCATTTACCCCTACCACACAACTGTCCTTGAACCTAGGAATAGCTATCCCCCTATGAGCAGGCACAGTAATTACTGGATTTCGACACAAAACAAAAGCCTCTTTAGCCCACTTTCTACCACAAGGAACCCCACTACCCCTAATCCCCATGCTCATTATCA
R-B_taurus : TCATTCACACCAACAACACAACTATCAATAAACCTAGGCATAGCCATCCCCCTGTGAGCAGGAGCCGTAATTACAGGATTCCGCAATAAAACTAAAGCATCACTTGCCCATTTCTTACCACAAGGAACACCCACTCCACTAATCCCAATACTAGTAATTA
A-B_taurus : TCATTCACACCAACAACACAACTATCAATAAACCTAGGCATAGCCATCCCCCTGTGAGCAGGAGCCGTAATTACAGGATTCCGCAATAAAACTAAAGCATCACTTGCCCATTTCTTACCACAAGGAACACCCACTCCACTAATCCCAATACTAGTAATTA
R-O_aries : TCATTTACACCAACTACACAACTATCAATAAACCTAGGCATGGCCATTCCTTTATGAGGAGGAGCTGTAATTACAGGCTTCCGCAACAAAACTAAAGCTTCACTCGCCCATTTCCTACCACAAGGGACACCCACCCCACTGATCCCAATACTAGTAATTA
A-O_aries : TCATTTACACCAACTACACAACTATCAATAAACCTAGGCATGGCCATTCCCTTATGAGCAGGAGCTGTAATTACAGGCTTCCGCAACAAAACTAAAGCTTCACTCGCCCATTTCCTACCACAAGGGACACCCACCCCACTGATCCCAATACTAGTAATTA
R-S_scrofa : TCATTCACACCCACCACACAACTATCAATAAACCTGGGTATAGCAATCCCCCTATGATCAGCAACCGTATTCACAGGATTCCGCCATAAAACCAAAACATCACTAGCCCACTTTCTACCACAAGGAACACCCGCCCCATTAATTCCTATGCTCGTAATTA
A-S_scrofa : TCATTCACACCCACCACACAACTATCAATAAACCTGGGTATAGCAATCCCCCTATGATCAGCAACCGTATTCACAGGATTCCGCCATAAAACCAAAACATCACTAGCCCACTTTCTACCACAAGGAACACCCGCCCCATTAATTCCTATGCTCGTAATTA
R-O_cuniculus : TCATTCACACCAACAACTCAATTATCAATAAATTTAGGGATAGCAATCCCCCTATGAGCAGGAGCCGTAATCACCGGGTTTCGATACAAAACTAAGGCGTCACTAGCCCATTTCCTCCCACAAGGAACCCCTATTCCTCTTATTCCTATGCTAATTGTTA
A-O_cuniculus : TCATTCACACCAACAACTCAATTATCAATAAATTTAGGGATAGCAATCCCCCTATGAGCAGGAGCCGTAATCACCGGGTTTCGATACAAAACTAAGGCGTCACTAGCCCATTTCCTCCCACAAGGAACCCCTATTCCTCTTATTCCTATGCTAATTGTTA
R-M_musculus : ACATTTACACCTACTACCCAACTATCCATAAATCTAAGTATAGCCATTCCACTATGAGCTGGAGCCGTAATTACAGGCTTCCGACACAAACTAAAAAGCTCACTTGCCCACTTCCTTCCACAAGGAACTCCAATTTCACTAATTCCAATACTTATTATTA
A-M_musculus : ACATTTACACCTACTACCCAACTATCCATAAATCTAAGTATAGCCATTCCACTATGAGCTGGAGCCGTAATTACAGGCTTCCGACACAAACTAAAAAGCTCACTTGCCCACTTCCTTCCACAAGGAACTCCAATTTCACTAATTCCAATACTTATTATTA
R-R_norvegicus : ACATTTACCCCTACCACTCAGCTATCTATAAACCTAAGCATAGCCATCCCCCTATGAGCAGGAGCCGTAATTCTAGGCTTCCGACACAAACTAAAAAATTCTTTAGCCCACTTCTTACCGCAAGGAACCCCCATCTCCCTAATTCCCATACTAATTATCA
A-R_norvegicus : ACATTTACCCCTACCACTCAGCTATCTATAAACCTAAGCATAGCCATCCCCCTATGAGCAGGAGCCGTAATTCTAGGCTTCCGACACAAACTAAAAAATTCTTTAGCCCACTTCTTACCGCAAGGAACCCCCATCTCACTAATTCCCATACTAATCATCA
R-M_coypus : TCCTTCACCCCTACAACACAACTATCTATAAACCTAGCTATAGCTATCCCCCTTTGAGCAGGAGCCGTATTATTAGGTTTCCGTTACAAGACAAAAGCATCTCTAGCCCACTTTCTCCCTCAAGGCACACCAGTTATCCTAATTCCCATATTAGTAATGA
A-M_coypus : TCCTTCACCCCTACAACACAACTATCTATAAACCTAGCTATAGCTATCCCCCTTTGAGCAGGAGCCGTATTATTAGGTTTCCGTTACAAGACAAAAGCATCTCTAGCCCACTTTCTCCCTCAAGGCACACCAGTTATCCTAATTCCCATATTAGTAATGA
R-A_platyrhynchos : ACATTCACCCCAACCACCCAGCTATCCATAAACATGGCCCTAGCCTTCCCCCTGTGGCTTGCTACCCTACTAACAGGCCTGCGAAACAAACCATCAGCCTCCTTGGCTCACTTACTGCCAGAAGGAACCCCAACACCCCTGATCCCCGCACTAATCCTGA
A-A_platyrhynchos : ACATTCACCCCAACCACCCAGCTATCCATAAACATGGCCCTAGCCTTCCCCCTGTGGCTTGCTACCCTACTAACAGGCCTGCGAAACAAACCATCAGCCTCCTTGGCTCACTTACTGCCAGAAGGAACCCCAACACCCCTGATCCCCGCACTAATCCTGA
R-G_gallus : ACCTTCACCCCAACTACCCAACTATCAATAAACATGGCCTTAGCCCTGCCACTATGACTAGCCACCTTACTAACAGGCCTGCGAAACCAACCCTCCGCCTCCTTAGGACACCTACTCCCTGAAGGCACCCCCACCCCACTGATTCCAGCCCTAATCATAA
A-G_gallus : ACCTTCACCCCAACTACCCAGCTATCAATAAACATGGCCTTAGCCCTGCCACTATGACTAGCCACCTTACTAACAGGCCTACGAAACCAACCCTCCGCCTCCTTAGGACACCTACTCCCTGAAGGCACCCCCACCCCACTGATTCCAGCCCTAATCATAA
 TCATTTACMCCWACAACACAACTATCAATAAACCTAGGCATAGCCATCCCCCTATGAGCAGGAACCGTAATTACAGGCTTCCGCCACAAAACAAAAGCATCACTAGCCCACTTCCTACCACAAGGAACACCCATCCCCCTAATCCCAATACTAGTAATTA

 * 10100 * 10120 * 10140 * 10160 * 10180 * 10200 * 10220 * 10240
R-E_caballus : TCGAGACTATCAGCCTATTTATTCAACCTGTAGCCCTAGCCGTGCGGCTAACCGCTAACATTACCGCCGGACACCTCCTAATACACCTCATCGGAGGGGCAACACTAGCCCTCATAAGCATCAGCCCCTCAACAGCCCTTATTACGTTTATCATCCTAAT
A-E_caballus : TCGAGACTATCAGCCTATTTATTCAACCTGTAGCCCTAGCCGTGCGGCTAACCGCTAACATTACCGCCGGACACCTCCTAATACACCTCATCGGAGGGGCAACACTAGCCCTCATAAGCATCAGCCCCTCAACAGCCCTTATTACGTTTATCATCCTAAT
R-C_bactrianus : TCGAGACTATTAGCCTATTCATTCAACCCGTGGCCCTGGCCGTTCGACTAACAGCCAATATCACAGCAGGCCACCTATTAATACACTTAATTGGGGGAGCCACTTTGGCACTAATAAGCATTAATACACCAACAGCCCTTATTACATTTATTGTCCTAAT
A-C_bactrianus : TCGAGACTATTAGCCTATTCATTCAACCCGTGGCCCTGGCCGTTCGACTAACAGCCAATATCACAGCAGGCCACCTATTAATACACTTAATTGGGGGAGCCACTTTGGCACTAATAAGCATTAATACACCAACAGCCCTTATTACATTTATTGTCCTAAT
R-C_lupus_familiaris : TCGAAACTATTAGTCTATTTATTCAACCCATGGCTCTAGCCGTTCGATTAACCGCCAATATTACTGCAGGACACCTCCTAATCCATTTGATTGGAGGGGCTACCTTAGCTCTTATCAATATTAGCGCGACCACAGCTTTTATCACTTTTATTATTCTAAT
A-C_lupus_familiaris : TCGAAACTATTAGTCTATTTATTCAACCCATGGCTCTAGCCGTTCGATTAACCGCCAATATTACTGCAGGACACCTCCTAATCCATTTGATTGGAGGGGCTACCTTAGCTCTTATCAATATTAGCGCGACCACAGCTTTTATCACTTTTATTATTCTAAT
R-N_procyonoides : TTGAGACTATTAGTCTATTCATTCAACCCATGGCCCTAGCCGTTCGGTTAACAGCCAATATTACCGCAGGACACCTTCTAATTCACCTAATCGGAGGCGCCACTCTAGCCCTAATCGACATCAGCACTACCACGGCCTTTATTACTTTTATTATCCTAAT
A-N_procyonoides : TTGAGACTATTAGTCTATTCATTCAACCCATGGCCCTAGCCGTTCGGTTAACAGCCAATATTACCGCAGGACACCTTCTAATTCACCTAATCGGAGGCGCCACTCTAGCCCTAATCGACATCAGCACTACCACGGCCTTTATTACTTTTATTATCCTAAT
R-V_lagopus : TCGAAACAATTAGCCTATTCATCCAGCCTATGGCCTTGGCCGTCCGATTAACAGCTAACATCACCGCAGGGCACCTATTAATCCATCTGATCGGAGGAGCCACTCTAGCCCTAATTAATATCAGTGCTACCACGGCCCTTATTACCTTTACAATCCTGGT
A-V_lagopus : TCGAAACAATTAGCCTATTCATCCAGCCTATGGCCTTGGCCGTCCGATTAACAGCTAACATCACCGCAGGGCACCTATTAATCCATCTGATCGGAGGAGCCACTCTAGCCCTAATTAATATCAGTGCTACCACGGCCCTTATTACCTTTACAATCCTGGT
R-M_putorius : TCGAAACTATCAGCCTATTTATTCAACCCATGGCCCTGGCCGTGCGACTAACAGCTAACATCACAGCGGGCCACCTATTAATTCACTTAATTGGAGGAGCCACCCTAGCCCTAATAAACATTAGTACTGTTACAGCAATAATTACCTTTTCCATCCTTGT
A-M_putorius : TCGAAACTATCAGCCTATTTATTCAACCCATGGCCCTGGCCGTGCGACTAACAGCTAACATCACAGCGGGCCACCTATTAATTCACTTAATTGGAGGAGCCACCCTAGCCCTAATAAACATTAGTACTGTTACAGCAATAATTACCTTTTCCATCCTTGT
R-B_taurus : TTGAAACTATCAGCCTTTTTATTCAACCTATAGCCCTCGCCGTGCGGTTAACAGCTAACATCACTGCAGGACACCTATTAATTCACCTAATCGGAGGAGCTACACTTGCACTAATAAGCATTAGCACTACAACAGCTCTAATTACATTCACCATTCTAAT
A-B_taurus : TTGAAACTATCAGCCTTTTTATTCAACCTATAGCCCTCGCCGTGCGGTTAACAGCTAACATCACTGCAGGACACCTATTAATTCACCTAATCGGAGGAGCTACACTTGCACTAATAAGCATTAGCACTACAACAGCTCTAATTACATTCACCATTCTAAT
R-O_aries : TTGAAACCATCAGCCTATTTATTCAACCAGTAGCCCTTGCCGTACGATTAACAGCTAATATCACGGCAGGACACTTACTAATTCACCTAATTGGAGGAGCCACCCTTGCACTAATAAGCATTAATACCACAACAGCACTCATCACATTCATTATCCTAAT
A-O_aries : TTGAAACCATTAGCCTATTTATTCAACCAGTAGCCCTTGCCGTACGATTAACAGCTAATATCACGGCAGGACACTTACTAATTCACCTAATTGGAGGAGCCACCCTTGCACTAATAAGCATTAATACCACAACAGCACTCATCACATTCATTATCCTAAT
R-S_scrofa : TTGAAACTATTAGCCTATTTATTCAACCAGTAGCCCTAGCCGTACGACTGACAGCCAACATTACAGCAGGGCACCTATTAATTCATCTAATTGGAGGGGCCACATTAGCACTACTCAACATCAGCACTATAACAGCTTTTATCACATTTACTATCCTCAT
A-S_scrofa : TTGAAACTATTAGCCTATTTATTCAACCAGTAGCCCTAGCCGTACGACTGACAGCCAACATTACAGCAGGGCACCTATTAATTCATCTAATTGGAGGGGCCACATTAGCACTACTCAACATCAGCACTATAACAGCTTTTATCACATTTACTATCCTCAT
R-O_cuniculus : TTGAAACTATTAGTCTCTTCATTCAACCTATAGCCCTAGCCGTACGGCTTACAGCCAATATCACAGCAGGCCACCTTCTCATACATCTTATTGGAGGCGCTGCACTAGCTTTAGTCTCAATTAGTCCAACAACAGCCCTAATTACCTTTATTATCCTTAT
A-O_cuniculus : TTGAAACTATTAGTCTCTTCATTCAACCTATAGCCCTAGCCGTACGGCTTACAGCCAATATCACAGCAGGCCACCTTCTCATACACCTTATTGGAGGCGCTGCACTAGCTTTAGTCTCAATTAGTCCAACAACAGCCCTAATTACCTTTATTATCCTTAT
R-M_musculus : TTGAAACAATTAGCCTATTTATTCAACCAATGGCATTAGCAGTCCGGCTTACAGCTAACATTACTGCAGGACACTTATTAATACACCTAATCGGAGGAGCTACTCTAGTATTAATAAATATTAGCCCACCAACAGCTACCATTACATTTATTATTTTACT
A-M_musculus : TTGAAACAATTAGCCTATTTATTCAACCAATGGCATTAGCAGTCCGGCTTACAGCTAACATTACTGCAGGACACTTATTAATACACCTAATCGGAGGAGCTACTCTAGTATTAATAAATATTAGCCCACCAACAGCTACCATTACATTTATTATTTTACT
R-R_norvegicus : TCGAAACTATCAGCCTATTTATTCAACCGATAGCACTAGCAGTACGACTAACAGCAAACATTACAGCAGGCCATCTATTAATGCATCTAATCGGAGGAGCTACTCTAGTACTTATAGACATCAGCCCACCAACCGCTACAATTACATTTATTATTCTACT
A-R_norvegicus : TCGAAACTATCAGCCTATTTATTCAACCGATAGCACTAGCAGTACGACTAACAGCAAACATTACAGCAGGCCATCTATTAATGCATCTAATCGGAGGAGCTACTCTAGTACTTATAGACATCAGCCCACCAACCGCTACAATTACATTTATTATTCTACT
R-M_coypus : TTGAAACCATTAGCTTATTCATCCAACCTATAGCCCTAGCAGTACGACTTACCGCTAACATTACTGCTGGTCACCTTCTAATTCACTTAATTGGAAGTGCAACTTCAGCTTTAATTTCTATTAGCATGCCAATCGCAACTATTACATTTATTATTCTTCT
A-M_coypus : TTGAAACCATTAGCTTATTCATCCAACCTATAGCCCTGGCAGTACGACTTACCGCTAACATTACTGCTGGTCACCTTCTAATTCACTTAATTGGAAGTGCAACTTCAGCTTTAATTTCTATTAGCATGTCAATCGCAACTATTACATTTATTATTCTTCT
R-A_platyrhynchos : TCGAAACAACCAGCCTGCTGATCCGGCCCTTAGCTCTAGGAGTCCGCCTCACAGCTAACCTCACAGCAGGCCACCTACTTATTCAACTCATCTCCACAGCCTCCATCGCACTCATGCCCATCCTTCCCACAGTATCAATCCTAACAATAGCCATCCTACT
A-A_platyrhynchos : TCGAAACAACCAGCCTGCTGATCCGGCCCTTAGCTCTAGGAGTCCGCCTCACAGCTAACCTCACAGCAGGCCACCTACTTATTCAACTCATCTCCACAGCCTCCATCGCACTCATGCCCATCCTTCCCACAGTATCAATCCTAACAATAGCCATCCTACT
R-G_gallus : TCGAAACAACCAGCCTACTTATTCGGCCATTAGCCCTAGGAGTACGCCTAACAGCAAACCTCACAGCTGGTCACCTACTTATCCAACTTATCTCTACAGCCACAATCGCCCTACTACCAATAATGCCATCAATCTCCGCCCTAACGGCACTCATCCTATT
A-G_gallus : TCGAAACAACCAGCCTACTTATTCGGCCATTAGCCCTAGGAGTACGCCTAACAGCAAACCTCACAGCTGGTCACCTACTTATCCAACTTATCTCTACAGCCACAATCGCCCTACTACCAATAATACCATCAATCTCCGCCCTAACGGCACTCATCCTATT
 TCGAAACTATTAGCCTATTTATTCAACCYATAGCCCTAGCCGTACGACTAACAGCTAACATCACAGCAGGACACCTACTAATTCACCTAATCGGAGGAGCCACTCTAGCACTAATAAACATTAGCACWACAACAGCCCTTATTACATTTATTATCCTAAT

 * 10260 * 10280 * 10300 * 10320 * 10340 * 10360 * 10380 * 10400
R-E_caballus : TCTACTAACTATCCTCGAATTCGCAGTAGCTATAATCCAAGCCTACGTATTCACTCTCCTGGTAAGCCTTTACTTACACGACAACACCTAATGACCCACCAAACCCACGCTTACCACATAGTAAACCCCAGCCCATGACCACTTACAGGAGCCCTATCAG
A-E_caballus : TCTACTAACTATTCTCGAATTCGCAGTAGCTATAATCCAAGCCTACGTATTCACTCTCCTGGTAAGCCTTTACTTACACGACAACACCTAATGACCCACCAAACCCACGCTTACCACATAGTAAACCCCAGCCCATGACCACTTACAGGAGCCCTATCAG
R-C_bactrianus : TTTACTCACGATCCTTGAATTTGCCGTGGCTATAATCCAAGCCTATGTGTTCACCCTATTAGTAAGCCTATACTTACATGACAATACCTAATGACCCACCAGACTCACGCATATCACATAGTGAATCCTAGCCCTTGGCCCCTCACTGGGGCCTTATCCG
A-C_bactrianus : TTTACTCACGATCCTTGAATTTGCCGTGGCTATAATCCAAGCCTATGTGTTCACCCTATTAGTAAGCCTATACTTACATGACAATACCTAATGACCCACCAGACTCACGCATATCACATAGTGAATCCTAGCCCTTGGCCCCTCACTGGGGCCTTATCCG
R-C_lupus_familiaris : CCTACTTACGATCCTAGAATTTGCTGTTGCCTTAATTCAAGCCTATGTTTTTACCTTACTAGTGAGTCTATACTTACATGACAACACCTAATGACCCACCAAACTCACGCTTACCACATAGTCAACCCAAGCCCATGACCGCTGACAGGGGCCCTTTCTG
A-C_lupus_familiaris : CCTACTTACGATCCTAGAATTTGCTGTTGCCTTAATTCAAGCCTATGTTTTTACCTTACTAGTGAGTCTATACTTACATGACAACACCTAATGACCCACCAAACTCACGCTTACCACATAGTCAACCCAAGCCCATGACCGCTGACAGGGGCCCTTTCTG
R-N_procyonoides : TCTACTCACTATTCTCGAATTCGCCGTTGCCCTTATCCAAGCCTACGTCTTTACTCTACTAGTAAGTCTGTACTTACATGACAACACCTAATGACCCACCAAACCCATGCCTATCATATAGTTAATCCAAGCCCATGACCACTAACTGGAGCTCTCTCCG
A-N_procyonoides : TCTACTCACTATTCTCGAATTCGCCGTTGCCCTTATCCAAGCCTACGTCTTTACTCTACTAGTAAGTCTGTACTTACATGACAACACCTAATGACCCACCAAACCCATGCCTATCATATAGTTAATCCAAGCCCATGACCACTAACTGGAGCTCTCTCCG
R-V_lagopus : TTTACTTACCATCCTCGAATTCGCCGTCGCTCTTATCCAAGCTTATGTCTTTACACTACTAGTAAGTCTATACTTACATGACAACACCTAATGACCCACCAAACTCATGCATACCACATAGTCAATCCAAGCCCATGACCACTAACAGGGGCCCTATCTG
A-V_lagopus : TTTACTTACCATCCTCGAATTCGCCGTCGCTCTTATCCAAGCTTATGTCTTTACACTACTAGTAAGTCTATACTTACATGACAACACCTAATGACCCACCAAACTCATGCATACCACATAGTCAATCCAAGCCCATGACCACTAACAGGGGCCCTATCTG
R-M_putorius : CTTATTAACTATCTTAGAATTTGCAGTAGCCCTTATTCAAGCTTACGTCTTTACCCTACTAGTAAGCCTATATTTACATGACAACACCTAATGACCCACCAAACACACTCATATCACATAGTCAATCCAAGCCCATGACCCCTGACAGGAGCTCTTTCCG
A-M_putorius : CTTATTAACTATCTTAGAATTTGCAGTAGCCCTTATTCAAGCTTACGTCTTTACCCTACTAGTAAGCCTATATTTACATGACAACACCTAATGACCCACCAAACACACTCATATCACATAGTCAATCCAAGCCCATGACCCCTGACAGGAGCTCTTTCCG
R-B_taurus : CCTACTAACAATTCTAGAGTTTGCAGTAGCTATAATCCAAGCCTATGTATTCACTCTCCTAGTCAGCCTATATCTGCATGACAACACATAATGACACACCAAACTCATGCTTATCATATAGTAAACCCAAGCCCTTGACCTCTTACAGGAGCTTTGTCTG
A-B_taurus : CCTACTAACAATTCTAGAGTTTGCAGTAGCTATAATCCAAGCCTATGTATTCACTCTCCTAGTCAGCCTATATCTGCATGACAACACATAATGACACACCAAACTCATGCTTATCATATAGTAAACCCAAGCCCTTGACCTCTTACAGGAGCTTTGTCTG
R-O_aries : TTTACTAACAGTTCTCGAATTCGCAGTGGCTATAATTCAAGCCTATGTATTTACCCTTCTAGTTAGCTTATACCTGCATGATAACACATAATGACACACCAAACCCACGCTTATCACATAGTAAACCCAAGCCCCTGACCTCTCACAGGAGCACTATCTG
A-O_aries : TTTACTAACAGTTCTCGAATTCGCAGTGGCTATAATTCAAGCCTATGTATTTACCCTTCTAGTTAGCTTATACCTGCATGATAACACATAATGACACACCAAACCCACGCTTATCACATAGTAAATCCAAGCCCCTGACCTCTCACAGGAGCACTATCTG
R-S_scrofa : CCTATTAACTATTCTTGAATTTGCAGTAGCTCTGATCCAAGCTTATGTGTTTACACTGCTAGTAAGCTTATACCTACACGACAATACATAATGACCCACCAAACACATGCATACCACATAGTAAACCCAAGCCCATGACCACTTACCGGAGCCCTATCAG
A-S_scrofa : CCTATTAACTATTCTTGAATTTGCAGTAGCTCTGATCCAAGCTTATGTGTTTACACTGCTAGTAAGCTTATACCTACACGACAATACATAATGACCCACCAAACACATGCATACCACATAGTAAACCCAAGCCCATGACCACTTACCGGAGCCCTATCAG
R-O_cuniculus : TCTACTAACAATTCTAGAATTCGCCGTAGCCTTGATCCAAGCTTACGTCTTTACTCTCCTTGTAAGCCTATACCTACATGACAATACCTAATGACACACCAAACTCACGCTTACCATATAGTCAACCCAAGCCCCTGACCACTCACCGGAGCCCTATCTG
A-O_cuniculus : TCTACTAACAATTCTAGAATTCGCCGTAGCCTTGATCCAAGCTTACGTCTTTACTCTCCTTGTAAGCCTATACCTACATGACAATACCTAATGACACACCAAACTCACGCTTACCATATAGTCAACCCAAGCCCCTGACCACTCACCGGAGCCCTATCTG
R-M_musculus : TCTACTCACAATTCTAGAATTTGCAGTAGCATTAATTCAAGCCTACGTATTCACCCTCCTAGTAAGCCTATATCTACATGATAATACATAATGACCCACCAAACTCATGCATATCACATAGTTAATCCAAGTCCATGACCATTAACTGGAGCCTTTTCAG
A-M_musculus : TCTACTCACAATTCTAGAATTTGCAGTAGCATTAATTCAAGCCTACGTATTCACCCTCCTAGTAAGCCTATATCTACATGATAATACATAATGACCCACCAAACTCATGCATATCACATAGTTAATCCAAGTCCATGACCATTAACTGGAGCCTTTTCAG
R-R_norvegicus : TCTACTTACAGTACTTGAATTTGCCGTAGCCTTAATTCAAGCCTATGTATTCACCCTTCTAGTAAGCCTGTACCTACATGATAACACATAATGACCCACCAAACCCATGCATACCATATAGTAAACCCAAGCCCATGACCACTAACAGGAGCCCTATCAG
A-R_norvegicus : TCTACTTACAGTACTTGAATTTGCCGTAGCCTTAATTCAAGCCTATGTATTCACCCTTCTAGTAAGCCTGTACCTACATGATAACACATAATGACCCACCAAACCCATGCATACCATATAGTAAACCCAAGCCCATGACCACTAACAGGAGCCCTATCAG
R-M_coypus : GCTCCTAACTTTACTTGAGTTCGCAGTAGCATTAATTCAAGCATATGTATTTACCTTATTAGTTAGTCTTTATTTACATGACAATACCTAATGACCCACCAAACACATGCCTACCATATAGTTAATCCTAGCCCTTGACCTCTTACAGGAGCCCTATCCG
A-M_coypus : GCTCCTAACTTTACTTGAGTTCGCAGTAGCATTAATTCAAGCATATGTATTTACCTTATTAGTTAGTCTTTATTTACATGACAATACCTAATGACCCACCAAACACATGCCTACCATATAGTTAATCCTAGCCCTTGACCTCTTACAGGAGCCCTATCCG
R-A_platyrhynchos : ACTCCTCACCATCCTAGAAGTAGCAGTGGCCATAATCCAGGCCTACGTTTTCGTCCTCCTCCTAAGCCTGTACTTACAAGAAAACATCTAATGGCACACCAAGCACACTCCTACCACATAGTCGACCCCAGCCCCTGACCAATCTTTGGAGCTGCCGCCG
A-A_platyrhynchos : ACTCCTCACCATCCTAGAAGTAGCAGTGGCCATAATCCAGGCCTACGTTTTCGTCCTCCTCCTAAGCCTGTACTTACAAGAAAACATCTAATGGCACACCAAGCACACTCCTACCACATAGTCGACCCCAGCCCCTGACCAATCTTTGGAGCTGCCGCCG
R-G_gallus : CCTACTAACCATCCTAGAAGTGGCAGTAGCAATAATCCAAGCCTACGTCTTCGTCCTCCTCCTAAGCCTCTACTTACAAGAAAATATTTAATGGCACACCAAGCACACTCCTACCACATAGTTGACCCAAGCCCATGACCAATCTTCGGCGCAGCCGCAG
A-G_gallus : CCTACTAACCATCCTAGAAGTGGCAGTAGCAATAATCCAAGCCTACGTCTTCGTCCTCCTCCTAAGCCTCTACTTACAAGAAAATATTTAATGGCACACCAAGCACACTCCTACCACATAGTTGACCCAAGCCCATGACCAATCTTCGGCGCAGCCGCAG
 TCTACTAACWATYCTAGAATTTGCAGTAGCYATAATCCAAGCCTATGTATTTACCCTMCTAGTAAGCCTATACTTACATGACAACACCTAATGACCCACCAAACTCACGCATACCACATAGTMAACCCAAGCCCATGACCACTCACAGGAGCCCTATCHG

 * 10420 * 10440 * 10460 * 10480 * 10500 * 10520 * 10540 * 10560
R-E_caballus : CCCTCCTGATAACATCAGGACTAGCCATGTGATTTCACTTTAACTCAACCTTACTTCTAGCTATAGGGCTATTAACTAACATCCTTACCATATATCAATGATGACGAGACATCATCCGAGAAAGCACATTCCAAGGCCATCACACATCAATCGTTCAAAA
A-E_caballus : CCCTCCTGATAACATCAGGACTAGCCATGTGATTTCACTTTAACTCAACCTTACTTCTAGCTATAGGGCTATTAACTAACATCCTTACCATATATCAATGATGACGAGACATCATCCGAGAAAGCACATTCCAAGGCCATCACACATCAATCGTTCAAAA
R-C_bactrianus : CCCTCCTAATAACGTCAGGCCTGACTATATGATTCCACTTCAATTCAAGTGTCCTGCTACTACTAGGTCTAGTTACAAATATACTGACTATATATCAATGGTGACGAGACGTTGTCCGAGAAAGCACATTCCAAGGGCATCACACGCCTGCCGTCCAAAA
A-C_bactrianus : CCCTCCTAATAACGTCAGGCCTGACTATATGATTCCACTTCAATTCAAGTGTCCTGCTACTACTAGGTCTAGTTACAAATATACTGACTATATATCAATGGTGACGAGACGTTGTCCGAGAAAGCACATTCCAAGGGCATCACACGCCTGCCGTCCAAAA
R-C_lupus_familiaris : CCCTCCTTATAACATCGGGTCTTATCATATGATTTCACTATAACTCAATAGCCCTACTTACATTAGGATTCACAACCAACCTGTTAACCATATGCCAGTGATGACGAGATGTGATCCGAGAAGGCACATTCCAAGGACATCATACCCCTATTGTACAAAA
A-C_lupus_familiaris : CCCTCCTTATAACATCGGGTCTTATCATATGATTTCACTATAACTCAATAGCCCTACTTACATTAGGATTCACAACCAACCTGTTAACCATATACCAGTGATGACGAGATGTGATCCGAGAAGGCACATTCCAAGGACATCATACCCCTATTGTACAAAA
R-N_procyonoides : CCCTACTTATAACGTCTGGTCTTATCATATGATTCCACTATAACTCAATATCCCTACTTATGCTAGGACTCACAACTAATATATTAACCATATTCCAATGGTGACGGGACGTGATCCGAGAGGGCACATTCCAAGGACACCATACCCCAATCGTACAAAA
A-N_procyonoides : CCCTACTTATAACGTCTGGTCTTATCATATGATTCCACTATAACTCAATATCCCTACTTATGCTAGGACTCACAACTAATATATTAACCATATTCCAATGGTGACGGGACGTGATCCGAGAGGGCACATTCCAAGGACACCATACCCCAATCGTACAAAA
R-V_lagopus : CCCTTCTTATAACATCGGGCCTTATTATATGATTTCATTACAACTCAATATCTCTACTTACTCTAGGACTCACAACTAATATGCTGACTATATACCAGTGGTGACGAGACGTAGTTCGAGAAGGCACATTTCAAGGACATCACACCTCTATTGTACAAAA
A-V_lagopus : CCCTTCTTATAACATCGGGCCTTATTATATGATTTCATTACAACTCAATATCTCTACTTACTCTAGGACTCACAACTAATATGCTGACTATATACCAGTGGTGACGAGACGTAGTTCGAGAAGGCACATTTCAAGGACATCACACCTCTATTGTACAAAA
R-M_putorius : CCCTACTTACAACATCAGGACTAGCAATATGATTCCACTACAATTCATTGTCTCTTCTAACCCTAGGAACTACAGCTAATGTACTAACCATATATCAATGGTGACGAGATGTGGTCCGAGAAGGAACATTTCAAGGCCACCATACCCCCACTGTTCAAAA
A-M_putorius : CCCTACTTACAACATCAGGACTAGCAATATGATTCCACTACAATTCATTGTCTCTTCTAACCCTAGGAACTACAGCTAATGTACTAACCATATATCAATGGTGACGAGATGTGGTCCGAGAAGGAACATTTCAAGGCCACCATACCCCCACTGTTCAAAA
R-B_taurus : CCCTCTTAATAACATCCGGCCTAACCATGTGATTTCACTTTAACTCAATGACCCTGCTAATAATTGGCCTAACAACAAATATACTAACAATATACCAATGATGACGAGATGTTATCCGAGAAAGCACCTTCCAAGGGCACCATACCCCAGCTGTCCAAAA
A-B_taurus : CCCTCTTAATAACATCCGGCCTAACCATGTGATTTCACTTTAACTCAATGACCCTGCTAATAATTGGCCTAACAACAAATATACTAACAATATACCAATGATGACGAGATGTTATCCGAGAAAGCACCTTCCAAGGGCACCATACCCCAGCTGTCCAAAA
R-O_aries : CCCTCCTAATAACATCTGGTCTCATCATATGATTTCACTTCAACTCAACAGCTCTACTAACTCTGGGCCTAACAACAAATATACTTACAATATACCAGTGATGACGAGATGTGATTCGAGAAAGCACCTTCCAAGGCCACCATACTCCGGCTGTCCAAAA
A-O_aries : CCCTCCTAATAACATCTGGTCTCATCATATGATTTCACTTCAACTCAACAGCTCTACTAACTCTGGGCCTAACAACAAATATACTTACAATATACCAGTGATGACGAGATGTGATTCGAGAAAGCACCTTCCAAGGCCACCATACTCCGGCTGTCCAAAA
R-S_scrofa : CCCTTTTAATAACATCAGGCCTAATTATATGATTCCACTTTAACTCTATACTCTTACTATCTCTAGGACTATTAACCAATACTTTGACAATATACCAATGGTGACGAGACATTATTCGAGAAAGCACTTTCCAAGGCCACCACACATCAGTCGTCCAAAA
A-S_scrofa : CCCTTTTAATAACATCAGGCCTAATTATATGATTCCACTTTAACTCTATACTCTTACTATCTCTAGGACTATTAACCAATACTTTGACAATATACCAATGGTGACGAGACATTATTCGAGAAAGCACTTTCCAAGGCCACCACACATCAGTCGTCCAAAA
R-O_cuniculus : CCCTTCTTATGACATCAGGCCTAGCCATATGATTTCATTTTAACTCCCCCTCACTTCTATTAATCGGCTTAGTAACCAACACTCTCACCATATACCAATGATGACGAGACATTGTACGAGAAGGTACATTCCAAGGCCACCATACTCCTATTGTACAAAA
A-O_cuniculus : CCCTTCTTATGACATCAGGCCTAGCCATATGATTTCATTTTAACTCCCCCTCACTTCTATTAATCGGCTTAGTGACCAACACTCTCACCATATACCAATGATGACGAGACATCGTACGAGAAGGTACATTCCAAGGCCACCATACTCCTATTGTACAAAA
R-M_musculus : CCCTCCTTCTAACATCAGGTCTAGTAATATGATTTCACTATAATTCAATTACACTATTAACCCTTGGCCTACTCACCAATATCCTCACAATATATCAATGATGACGAGACGTAATTCGTGAAGGAACCTACCAAGGCCACCACACTCCTATTGTACAAAA
A-M_musculus : CCCTCCTTCTAACATCAGGTCTAGTAATATGATTTCACTATAATTCAATTACACTATTAACCCTTGGCCTACTCACCAATATCCTCACAATATATCAATGATGACGAGACGTAATTCGTGAAGGAACCTACCAAGGCCACCACACTCCTATTGTACAAAA
R-R_norvegicus : CTCTTCTACTTACATCCGGCTTAGTAATATGATTCCATTACAACTCCACAATTCTCCTATCATTAGGCCTCCTGACAAACATCCTAACTATATATCAATGATGACGAGATATCATCCGTGAAGGAACATACCAAGGCCACCACACCCCTATTGTACAAAA
A-R_norvegicus : CTCTTCTACTCACATCCGGCTTAGTAATATGATTCCATTACAACTCCACAATTCTCCTATCATTAGGCCTCCTGACAAACATCCTAACTATATATCAATGATGACGAGATATCATCCGTGAAGGAACATACCAAGGCCACCATACCCCTATTGTACAAAA
R-M_coypus : CTCTTCTACTTACCTCTGGCTTAATTATATGATTTCACTTTGCTTCTACAACCCTCCTAACCCTAAGCATGCTAACTAATATAATAACCATATACCAATGATGACGTGATGTAGTACGAGAAGGTACATATCAAGGCCACCACACATCAACAGTACAAAA
A-M_coypus : CTCTTCTACTTACCTCTGGCTTAATTATATGATTTCACTTTGCTTCTACAACCCTCCTAACCCTAAGCATGCTAACTAATATAATAACCATATACCAATGATGACGTGATGTAGTACGAGAAGGTACATATCAAGGCCACCACACATCAACAGTACAAAA
R-A_platyrhynchos : CCTTACTCACAACCTCAGGGCTAGTCATGTGATTCCACTACAACTCATCTATCCTGCTAGCCGCCGGCCTCTTATCAATGCTCCTAGTGATACTCCAATGATGACGGGACATTGTCCGAGAGAGCACCTTCCAAGGCCACCACACACCTACAGTCCAAAA
A-A_platyrhynchos : CCTTACTCACAACCTCAGGGCTAGTCATGTGATTCCACTACAACTCATCTATCCTGCTAGCCGCCGGCCTCTTATCAATGCTCCTAGTGATACTCCAATGATGACGGGACATTGTCCGAGAGAGCACCTTCCAAGGCCACCACACACCTACAGTCCAAAA
R-G_gallus : CACTACTAACCACCTCTGGCCTAATCATATGGTTCCACTACAGCTCGACCACCCTACTGACAATAGGCCTCCTCTCTATACTTCTAGTCATGCTGCAATGATGACGAGACGTAGTCCGAGAAAGCACCTTCCAGGGCCACCACACCCCAACTGTCCAAAA
A-G_gallus : CACTACTAACCACCTCTGGCCTAATCATATGGTTCCACTACAGCTCGACCACCCTACTGACAATAGGCCTCCTCTCTATGCTTCTAGTCATGCTGCAATGATGACGAGACGTAGTCCGAGAAAGCACCTTCCAGGGCCACCACACCCCAACTGTCCAAAA
 CCCTCCTAATAACATCAGGCCTAATCATATGATTTCACTATAACTCAAYAACCCTACTAACACTAGGCCTAATAACTAATATACTAACCATATACCAATGATGACGAGACGTTATCCGAGAAGGCACATTCCAAGGCCACCACACCCCTATTGTACAAAA

 * 10580 * 10600 * 10620 * 10640 * 10660 * 10680 * 10700 * 10720
R-E_caballus : GGGACTCCGATATGGCATAATCCTTTTTATTATCTCAGAAGTCTTCTTCTTCTCTGGCTTCTTCTGAGCCTTTTACCACTCAAGCCTAGCCCCCACACCCGAACTAGGCGGCTGCTGACCACCCACAGGTATCCACCCCTTAAACCCCCTAGAAGTCCCC
A-E_caballus : GGGACTCCGATATGGCATAATCCTTTTTATTATCTCAGAAGTCTTCTTCTTCTCTGGCTTCTTCTGAGCCTTTTACCACTCAAGCCTAGCCCCCACACCCGAACTAGGCGGCTGCTGACCACCCACAGGTATCCACCCCTTAAACCCCCTAGAAGTCCCC
R-C_bactrianus : AGGCTTGCGATACGGAATAATCCTATTTATTGTGTCGGAGGTTTTATTTTTTACCGGATTTTTCTGAGCCTTTTATCACTCAAGCCTAGCCCCCACTCCCGAACTGGGAGGATGCTGACCTCCTACCGGCATCCACCCCTTAAACCCGCTAGAAGTTCCT
A-C_bactrianus : AGGCTTGCGATACGGAATAATCCTATTTATTGTGTCGGAGGTTTTATTTTTTACCGGATTTTTCTGAGCCTTTTATCACTCAAGCCTAGCCCCCACTCCCGAACTGGGAGGATGCTGACCTCCTACCGGCATCCACCCCTTAAACCCGCTAGAAGTTCCT
R-C_lupus_familiaris : AGGACTACGATACGGAATAGTTCTTTTTATCGTATCAGAAGTATTTTTCTTTGCAGGCTTCTTCTGAGCCTTTTACCACTCCAGCCTAGCCCCTACTCCTGAACTTGGGGGTTGCTGACCTCCTACCGGCATTATTCCTCTTAACCCATTAGAAGTGCCT
A-C_lupus_familiaris : AGGACTACGATACGGAATAGTTCTTTTTATCGTATCAGAAGTATTTTTCTTTGCAGGCTTCTTCTGAGCCTTTTACCACTCCAGCCTAGCCCCTACTCCTGAACTTGGGGGTTGCTGACCTCCTACCGGCATTATTCCTCTTAACCCATTAGAAGTGCCT
R-N_procyonoides : GGGCCTACGATACGGAATAATTCTATTCATCGTTTCGGAAGTATTTTTCTTCGCAGGGTTCTTCTGAGCCTTTTACCACTCTAGTTTAGCTCCAACCCCCGAATTAGGGGGATGCTGACCACCCACCGGCGTCATTCCTCTAAACCCACTGGAAGTCCCT
A-N_procyonoides : GGGCCTACGATACGGAATAATTCTATTCATCGTTTCGGAAGTATTTTTCTTCGCAGGGTTCTTCTGAGCCTTTTACCACTCTAGTTTAGCTCCAACCCCCGAATTAGGGGGATGCTGACCACCCACCGGCGTCATTCCTCTAAACCCACTGGAAGTCCCT
R-V_lagopus : GGGATTACGATATGGGATAATCCTATTCATCGTCTCAGAAGTTTTCTTTTTCGCTGGGTTTTTCTGAGCCTTTTATCACTCTAGCCTAGCCCCAACCCCCGAACTCGGAGGTTGTTGACCCCCTACCGGTATCACCCCCCTAAACCCGCTTGATGTTCCT
A-V_lagopus : GGGATTACGATATGGGATAATCCTATTCATCGTCTCAGAAGTTTTCTTTTTCGCTGGGTTTTTCTGAGCCTTTTATCACTCTAGCCTAGCCCCAACCCCCGAACTCGGAGGTTGTTGACCCCCTACCGGTATCACCCCCCTAAACCCGCTTGATGTTCCT
R-M_putorius : AGGTTTACGATACGGAATAATCCTCTTCATCACATCCGAAGTCTTTTTCTTTGCAGGCTTCTTCTGGGCTTTTTACCATTCAAGCCTAGCCCCAACACCCGAACTTGGAGGGTGCTGACCACCCACAGGTATTACACCCCTAAACCCCTTAGAAGTACCA
A-M_putorius : AGGTTTACGATACGGAATAATCCTCTTCATCACATCCGAAGTCTTTTTCTTTGCAGGCTTCTTCTGGGCTTTTTACCATTCAAGCCTAGCCCCAACACCCGAACTTGGAGGGTGCTGACCACCCACAGGTATTACACCCCTAAACCCCTTAGAAGTACCA
R-B_taurus : AGGCCTCCGTTATGGAATAATTCTTTTTATTATCTCCGAAGTACTATTCTTTACCGGATTTTTCTGAGCTTTCTACCACTCAAGCCTCGCCCCCACCCCTGAACTAGGCGGCTGCTGACCCCCAACAGGCATTCACCCACTAAACCCCCTAGAAGTCCCA
A-B_taurus : AGGCCTCCGTTATGGAATAATTCTTTTTATTATCTCCGAAGTACTATTCTTTACCGGATTTTTCTGAGCTTTCTACCACTCAAGCCTCGCCCCCACCCCTGAACTAGGCGGCTGCTGACCCCCAACAGGCATTCACCCACTAAACCCCCTAGAAGTCCCA
R-O_aries : GGGCCTTCGTTACGGAATGATTCTTTTCATTATCTCCGAAGTTCTATTCTTTACTGGATTTTTCTGAGCCTTCTACCACTCAAGCCTTGCCCCCACACCCGAACTAGGCGGCTGCTGACCTCCAACAGGCATTCACCCACTTAATCCCTTAGAAGTCCCA
A-O_aries : GGGCCTTCGTTACGGAATGATTCTTTTCATTATCTCCGAAGTTCTATTCTTTACTGGATTTTTCTGAGCCTTCTACCACTCAAGCCTTGCCCCCACACCCGAACTAGGCGGCTGCTGACCTCCAACAGGCATTCACCCACTTAATCCCTTAGAAGTCCCA
R-S_scrofa : AGGCTTACGATACGGTATAATTTTATTTATTATTTCCGAGGTTCTGTTCTTCACTGGATTCTTTTGAGCTTTCTACCACTCAAGCCTAGCACCAACACCCGAATTAGGAGGTTGCTGACCACCAACAGGAATTCACCCACTAAACCCCCTAGAAGTACCC
A-S_scrofa : AGGCTTACGATACGGTATAATTTTATTTATTATTTCCGAGGTTCTGTTCTTCACTGGATTCTTTTGAGCTTTCTACCACTCAAGCCTAGCACCAACACCCGAATTAGGAGGTTGCTGACCACCAACAGGAATTCACCCACTAAACCCCCTAGAAGTACCC
R-O_cuniculus : AGGTCTACGATATGGCATAATTCTCTTTATCATCTCAGAAGTATTCTTTTTCGCGGGCTTCTTCTGAGCCTTCTACCACTCAAGCCTAGCTCCTACCCCAGAACTAGGAGGATGCTGACCTCCAACAGGCATCAACCCCCTTAATCCCCTTGAAGTGCCG
A-O_cuniculus : AGGCCTACGATATGGCATAATTCTCTTTATCATCTCAGAAGTATTCTTTTTCGCGGGCTTCTTCTGAGCCTTCTACCACTCAAGCCTAGCTCCTACCCCAGAACTAGGAGGATGCTGACCTCCAACAGGCATCAACCCCCTTAATCCCCTTGAAGTGCCG
R-M_musculus : AGGACTACGATATGGTATAATTCTATTCATCGTCTCGGAAGTATTTTTCTTTGCAGGATTCTTCTGAGCGTTCTATCATTCTAGCCTCGTACCAACACATGATCTAGGAGGCTGCTGACCTCCAACAGGAATTTCACCACTTAACCCTCTAGAAGTCCCA
A-M_musculus : AGGACTACGATATGGTATAATTCTATTCATCGTCTCGGAAGTATTTTTCTTTGCAGGATTCTTCTGAGCGTTCTATCATTCTAGCCTCGTACCAACACATGATCTAGGAGGCTGCTGACCTCCAACAGGAATTTCACCACTTAACCCTCTAGAAGTCCCA
R-R_norvegicus : AGGCCTCCGATACGGAATAATCCTGTTTATTGTCTCCGAAGTATTCTTCTTTGCCGGATTTTTCTGAGCATTTTATCATTCCAGCCTAGTTCCTACCCACGACCTAGGCGGTTGCTGACCCCCAACAGGAATTACCCCTTTAAATCCCCTAGAAGTACCC
A-R_norvegicus : AGGCCTCCGATACGGAATAATCCTGTTTATTGTCTCCGAAGTATTCTTCTTTGCCGGATTTTTCTGAGCATTTTATCATTCCAGCCTAGTTCCTACCCACGACCTAGGCGGTTGCTGACCCCCAACAGGAATTACCCCTTTAAATCCCCTAGAAGTACCC
R-M_coypus : AGGCCTTCGATATGGTATAATTTTATTTATTATCTCAGAAGTGTTTTTCTTCTCAGGTTTCTTCTGAGCTTTCTATCACTCAAGCTTAGCCCCTACTCCAGAACTAGGAGGTTATTGACCTCCCACAGGAGTAAACCCTCTTAACCCCTTAGAAGTACCT
A-M_coypus : AGGCCTTCGATATGGTATAATTTTATTTATTATCTCAGAAGTGTTTTTCTTCTCAGGTTTCTTCTGAGCTTTCTATCACTCAAGCTTAGCCCCTACTCCAGAACTAGGAGGTTATTGACCTCCCACAGGAGTAAACCCTCTTAACCCCTTAGAAGTACCT
R-A_platyrhynchos : AGGCCTACGATACGGCATAATCCTCTTCATCACATCCGAAGCTTTCTTCTTCCTAGGATTTTTCTGGGCATTCTTCCACTCAAGCCTAGTACCAACCCCCGAACTAGGCGGCCAATGACCCCCAGCGGGCATCAAACCGCTCAACCCCATAGAAGTCCCG
A-A_platyrhynchos : AGGCCTACGATACGGCATAATCCTCTTCATCACATCCGAAGCTTTCTTCTTCCTAGGATTTTTCTGGTCATTCTTCCACTCAAGCCTAGTACCAACCCCCGAACTAGGCGGCCAATGACCCCCAGCGGGCATCAAACCGCTCAACCCCATAGAAGTCCCG
R-G_gallus : GGGCCTACGATACGGAATAATCCTTTTCATTACATCAGAGGCCTTCTTCTTCCTAGGATTCTTCTGAGCCTTCTTCCACTCAAGCCTAGCCCCAACACCAGAACTGGGGGGCCAATGGCCCCCAACAGGAGTCAAACCCCTAAACCCCCTTGAAGTACCC
A-G_gallus : GGGCCTACGATACGGAATAATCCTTTTCATTACATCAGAGGCCTTCTTCTTCCTAGGATTCTTCTGAGCCTTCTTCCACTCAAGCCTAGCCCCAACACCAGAACTGGGGGGCCAATGGCCCCCAACAGGAGTCAAACCCCTAAACCCCCTTGAAGTACCC
 AGGCCTACGATACGGAATAATTCTATTTATTATCTCMGAAGTATTCTTCTTCGCAGGATTCTTCTGAGCCTTCTACCACTCAAGCCTAGCCCCAACMCCCGAACTAGGAGGCTGCTGACCTCCAACAGGCATYAACCCCCTAAACCCCCTAGAAGTCCCT

 * 10740 * 10760 * 10780 * 10800 * 10820 * 10840 * 10860 * 10880
R-E_caballus : TTACTCAACACCTCAGTGCTCCTAGCATCTGGAGTCTCTATCACCTGAGCCCACCATAGCCTAATAGAAGGAAACCGTAAAAATATGCTCCAAGGCCTATTCATCACAATTTCACTAGGCGTATACTTCACCCTTCTCCAAGCCTCAGAATACTATGAAG
A-E_caballus : TTACTCAACACCTCAGTGCTCCTAGCATCTGGAGTCTCTATCACCTGAGCCCACCATAGCCTAATAGAAGGAAACCGTAAAAATATGCTCCAAGGCCTATTCATCACAATTTCACTAGGCGTATACTTCACCCTTCTCCAAGCCTCAGAATACTATGAAG
R-C_bactrianus : CTTCTCAACACCTCAGTCCTATTAGCCTCCGGAGTCTCAATCACCTGGGCCCATCACAGCCTGATGGAAGGCAACCGTGCCCATATACTCCAAGCCCTATTTATTACGATTGCCCTAGGACTATATTTCACGCTACTCCAGGCATCAGAATATTACGAAG
A-C_bactrianus : CTTCTCAACACCTCAGTCCTATTAGCCTCCGGAGTCTCAATCACCTGGGCCCATCACAGCCTGATGGAAGGCAACCGTGCCCATATACTCCAAGCCCTATTTATTACGATTGCCCTAGGACTATATTTCACGCTACTCCAGGCATCAGAATATTACGAAG
R-C_lupus_familiaris : CTACTCAACACCTCAGTCCTCCTAGCCTCCGGAGTATCTATTACTTGAGCCCATCATAGTTTAATAGAAGGTAATCGCAAACATATACTTCAAGCCTTATTCATTACAATCTCCTTAGGCGTATATTTTACGCTATTACAGGCCTCCGAATACTATGAGA
A-C_lupus_familiaris : CTACTCAACACCTCAGTCCTCCTAGCCTCCGGAGTATCTATTACTTGAGCCCATCATAGTTTAATAGAAGGTAATCGCAAACATATACTTCAAGCCTTATTCATTACAATCTCCTTAGGCGTATATTTTACGCTATTACAGGCCTCCGAATACTATGAGA
R-N_procyonoides : CTACTTAACACCTCCGTCCTCCTAGCTTCCGGAGTCTCAATCACTTGAGCCCACCATAGCTTAATAGAAGGAAATCGTAAACACATACTTCAAGCCCTATTTATCACTATCTCCCTAGGTGTATACTTTACATTACTTCAAGCATCCGAATATTACGAAA
A-N_procyonoides : CTACTTAACACCTCCGTCCTCCTAGCTTCCGGAGTCTCAATCACTTGAGCCCACCATAGCTTAATAGAAGGAAATCGTAAACACATACTTCAAGCCCTATTTATCACTATCTCCCTAGGTGTATACTTTACATTACTTCAAGCATCCGAATATTACGAAA
R-V_lagopus : TTACTCAACACCTCTGTTCTCCTAGCCTCAGGAGTCTCAATCACCTGGGCCCACCATAGCCTAATAGAAGGTAATCGTAAACACATACTTCAAGCCCTATTTATTACCATTTCCCTAGGCGTATACTTTACACTACTACAAGCATCTGAGTATTACGAGA
A-V_lagopus : TTACTCAACACCTCTGTTCTCCTAGCCTCAGGAGTCTCAATCACCTGGGCCCACCATAGCCTAATAGAAGGTAATCGTAAACACATACTTCAAGCCCTATTTATTACCATTTCCCTAGGCGTATACTTTACACTACTACAAGCATCTGAGTATTACGAGA
R-M_putorius : TTACTAAATACCTCTGTCCTCCTAGCCTCCGGAGTCTCTATTACTTGGGCCCACCACAGCCTCATAGAAGGGGACCGCAAACACATACTCCAAGCCCTATTTATCACAATCTCCCTAGGCCTGTATTTTACTGTCCTACAAGCCTCCGAATACTACGAAG
A-M_putorius : TTACTAAATACCTCTGTCCTCCTAGCCTCCGGAGTCTCTATTACTTGGGCCCACCACAGCCTCATAGAAGGGGACCGCAAACACATACTCCAAGCCCTATTTATCACAATCTCCCTAGGCCTGTATTTTACTGTCCTACAAGCCTCCGAATACTACGAAG
R-B_taurus : CTGCTCAACACCTCTGTCCTATTGGCTTCCGGAGTTTCTATTACCTGAGCCCATCATAGTTTAATAGAAGGGGACCGAAAGCATATATTACAAGCCCTATTTATCACCATCACATTAGGAGTCTACTTCACACTACTACAAGCCTCAGAATACTATGAAG
A-B_taurus : CTGCTCAACACCTCTGTCCTATTGGCTTCCGGAGTTTCTATTACCTGAGCCCATCATAGTTTAATAGAAGGGGACCGAAAGCATATATTACAAGCCCTATTTATCACCATCACATTAGGAGTCTACTTCACACTACTACAAGCCTCAGAATACTATGAAG
R-O_aries : CTACTCAACACCTCTGTCCTTCTAGCCTCAGGAGTATCCATTACTTGAGCTCACCATAGCCTCATAGAAGGGAACCGTTACCACATGTTACAAGCCCTATTCATTACCATCGCACTAGGCGTGTACTTTACACTGTTACAGGCATCAGAGTATTATGAAG
A-O_aries : CTACTCAACACCTCTGTCCTTCTAGCCTCAGGAGTATCCATTACTTGAGCTCACCATAGCCTCATAGAAGGGAACCGTTACCACATGTTACAAGCCCTATTCATTACCATCGCACTAGGCGTATACTTTACACTGTTACAGGCATCAGAGTATTATGAAG
R-S_scrofa : CTACTAAACACCTCAATCCTCCTCGCCTCAGGAGTATCCATTACCTGAGCCCATCACAGCCTAATAGAAGGGGACCGAAAACACATAATCCAAGCACTATCCATCACCATTGCACTAGGCGTATACTTCACCCTCCTCCAAGCCTCAGAATATTACGAAG
A-S_scrofa : CTACTAAACACCTCAATCCTCCTCGCCTCAGGAGTATCCATTACCTGAGCCCATCACAGCCTAATAGAAGGGGACCGAAAACACATAATCCAAGCACTATCCATCACCATTGCACTAGGCGTATACTTCACCCTCCTCCAAGCCTCAGAATATTACGAAG
R-O_cuniculus : CTACTAAACACTTCAGTCCTCCTGGCTTCAGGAGTCTCAATCACCTGGGCCCACCACAGTTTAATAGAAGGCAATCGCAAAAACATACAGCAAGCTCTAGCCATTACCATTCTCCTAGGTATCTATTTTACCCTACTTCAAGCATCGGAGTATTACGAAA
A-O_cuniculus : CTACTAAACACTTCAGTCCTCCTGGCTTCAGGAGTCTCAATCACCTGGGCCCACCACAGTTTAATAGAAGGCAATCGCAAAAACATACAGCAAGCTCTAGCCATTACCATTCTCCTAGGTATCTATTTTACCCTACTTCAAGCATCGGAGTATTACGAAA
R-M_musculus : CTACTTAATACTTCAGTACTTCTAGCATCAGGTGTTTCAATTACATGAGCTCATCATAGCCTTATAGAAGGTAAACGAAACCACATAAATCAAGCCCTACTAATTACCATTATACTAGGACTTTACTTCACCATCCTCCAAGCTTCAGAATACTTTGAAA
A-M_musculus : CTACTTAATACTTCAGTACTTCTAGCATCAGGTGTTTCAATTACATGAGCTCATCATAGCCTTATAGAAGGTAAACGAAACCACATAAATCAAGCCCTACTAATTACCATTATACTAGGACTTTACTTCACCATCCTCCAAGCTTCAGAATACTTTGAAA
R-R_norvegicus : CTTCTAAATACATCAGTCCTCTTAGCATCAGGAGTCTCAATTACATGAGCCCATCACAGCCTAATAGAAGGCAACCGAAACCATATAAACCAAGCCCTACTAATCACCATTCTCTTAGGATTATATTTCACTATCTTACAAGCCTCAGAGTATTTCGAAA
A-R_norvegicus : CTTCTAAATACATCAGTCCTCTTAGCATCAGGAGTCTCAATTACATGAGCCCATCACAGCCTAATAGAAGGCAACCGAAACCATATAAACCAAGCCCTACTAATCACCATTCTCTTAGGATTATATTTCACGATCTTACAAGCCTCAGAGTATTTCGAAA
R-M_coypus : TTACTAAATACATCTATTCTATTGGCTTCAGGAGTCTCAATCACCTGAGCTCATCACAGTCTGATAGAAGGGAATCGAAAACAAATAACTCAAGCCCTCACAATTACTATCGCCCTAGGAATTTACTTCACACTACTACAAGCATCAGAATACTTTGAAG
A-M_coypus : TTACTAAATACATCTATTCTATTGGCTTCAGGAGTCTCAATCACCTGAGCTCATCACAGTCTGATAGAAGGGAATCGAAAACAAATAACTCAAGCCCTCACAATTACTATCGCCCTAGGAATTTACTTCACACTACTACAAGCATCAGAATACTTTGAAG
R-A_platyrhynchos : CTACTAAACACAGCCATCCTCCTAGCCTCAGGCGTAACTGTCACATGAGCCCACCACAGCATCACAGAAGGAAACCGAAAACATGCCATCCACGCCCTAACATTGACGATTCTCCTAGGATTCTACTTCACCGCCCTACAAGCAATAGAGTACCATGAAG
A-A_platyrhynchos : CTACTAAACACAGCCATCCTCCTAGCCTCAGGCGTAACTGTCACATGAGCCCACCACAGCATCACAGAAGGAAACCGAAAACATGCCATCCACGCCCTAACATTGACGATTCTCCTAGGATTCTACTTCACCGCCCTACAAGCAATAGAGTACCATGAAG
R-G_gallus : CTACTAAATACAGCAATCCTCCTAGCCTCAGGAGTCACCGTTACATGGGCTCACCACAGCATCACAGAAGGAAACCGAAAACAAGCCATCCACGCACTAACTCTCACAATCCTCCTAGGATTCTATTTCACAGCCCTACAAGCAATAGAGTACCATGAAG
A-G_gallus : CTACTAAATACAGCAATCCTCCTAGCCTCAGGAGTCACCGTTACATGGGCTCACCACAGCATCACAGAAGGAAACCGAAAACAAGCCATCCACGCACTAACTCTCACAATCCTCCTAGGATTCTACTTCACAGCCCTACAAGCAATAGAGTACCATGAAG
 CTACTAAACACCTCAGTCCTCCTAGCCTCAGGAGTCTCAATTACCTGAGCCCACCACAGCCTAATAGAAGGGAACCGAAAACACATACTCCAAGCCCTATTTATYACCATTTCCCTAGGAGTATACTTCACACTACTACAAGCATCAGAATACTATGAAG

 * 10900 * 10920 * 10940 * 10960 * 10980 * 11000 * 11020 * 11040
R-E_caballus : CCTCATTTACTATTTCAGATGGAGTATACGGATCAACATTTTTCGTAGCAACAGGGTTCCACGGACTACACGTAATTATCGGATCTACCTTCCTCATTGTATGTTTCCTACGCCAACTAAAATTCCACTTTACATCCAGCCACCACTTCGGATTCGAAGC
A-E_caballus : CCTCATTTACTATTTCAGATGGAGTATACGGATCAACATTTTTCGTGGCAACAGGGTTCCACGGACTACACGTAATTATCGGATCTACCTTCCTCATTGTATGTTTCCTACGCCAACTAAAATTCCACTTTACATCCAGCCACCACTTCGGATTCGAAGC
R-C_bactrianus : CACCCTTCACAATCTCGGACGGTGTTTATGGGTCTACCTTCTTTGTAGCTACCGGATTCCACGGACTACATGTTATTATTGGCTCCACTTTCCTAACAGTGTGCTTTCTACGACAATTGAAATTCCACTTCACATCTAGTCACCATTTCGGATTTGAAGC
A-C_bactrianus : CACCCTTCACAATCTCGGACGGTGTTTATGGGTCTACCTTCTTTGTAGCTACCGGATTCCACGGACTACATGTTATTATTGGCTCCACTTTCCTAACAGTGTGCTTTCTACGACAATTGAAATTCCACTTCACATCTAGTCACCATTTCGGATTTGAAGC
R-C_lupus_familiaris : CATCTTTTACAATCTCCGATGGGGTATACGGATCTACCTTTTTTATAGCCACTGGATTTCACGGATTACACGTAATTATTGGCTCTACATTCCTCATCGTGTGCTTCCTCCGACAGCTATACTACCACTTCACATCAAACCACCACTTCGGATTTGAAGC
A-C_lupus_familiaris : CATCTTTTACAATCTCCGATGGGGTATACGGATCTACCTTTTTTATAGCCACTGGATTTCACGGATTACACGTAATTATTGGCTCTACATTCCTCATCGTGTGCTTCCTCCGACAGCTATACTACCACTTCACATCAAACCACCACTTCGGATTTGAAGC
R-N_procyonoides : CATCCTTTACAATCTCCGACGGAGTGTACGGATCCACTTTTTTCATAGCCACCGGATTCCACGGTCTACATGTAATTATTGGCTCCACATTCCTTATCGTATGTTTCCTCCGGCAACTATACTATCACTTTACATCTAATCACCACTTTGGATTTGAAGC
A-N_procyonoides : CATCCTTTACAATCTCCGACGGAGTGTACGGATCCACTTTTTTCATAGCCACCGGATTCCACGGTCTACATGTAATTATTGGCTCCACATTCCTTATCGTATGTTTCCTCCGGCAACTATACTATCACTTTACATCTAATCACCACTTTGGATTTGAAGC
R-V_lagopus : CATCCTTCACAATCTCCGACGGAGTCTATGGGTCCACATTCTTCATAGCCACCGGATTCCACGGACTACACGTAATTATCGGCTCCACATTTCTTATTGTTTGCTTTATGCGACAACTGCACTACCATTTCACATCTAATCACCACTTCGGATTTGAAGC
A-V_lagopus : CATCCTTCACAATCTCCGACGGAGTCTATGGGTCCACATTCTTCATAGCCACCGGATTCCACGGACTACACGTAATTATCGGCTCCACATTTCTTATTGTTTGCTTTATGCGACAACTGCACTACCATTTCACATCTAATCACCACTTCGGATTTGAAGC
R-M_putorius : CTCCATTCACAATCTCAGACGGAGTCTACGGCTCTACATTTTTTATAGCCACAGGATTCCACGGCCTCCATGTCATTATCGGATCTACATTTCTTATCGTATGTTTCCTACGACAACTAAGCTACCATTTTACATCTAATCACCATTTTGGATTCGAAGC
A-M_putorius : CTCCATTCACAATCTCAGACGGAGTCTACGGCTCTACATTTTTTATAGCCACAGGATTCCACGGCCTCCATGTCATTATCGGATCTACATTTCTTATCGTATGTTTCCTACGACAACTAAGCTACCATTTTACATCTAATCACCATTTTGGATTCGAAGC
R-B_taurus : CACCTTTTACTATCTCCGACGGAGTTTACGGCTCAACTTTTTTTGTAGCCACAGGCTTCCACGGCCTCCACGTCATCATTGGGTCCACCTTCTTAATTGTCTGCTTCTTCCGCCAATTAAAATTTCATTTTACTTCTAACCACCACTTCGGCTTTGAAGC
A-B_taurus : CACCTTTTACTATCTCCGACGGAGTTTACGGCTCAACTTTTTTTGTAGCCACAGGCTTCCACGGCCTCCACGTCATCATTGGGTCCACCTTCTTAATTGTCTGCTTCTTCCGCCAATTAAAATTTCATTTTACTTCTAACCACCACTTCGGCTTTGAAGC
R-O_aries : CACCCTTTACAATCTCAGACGGAGTTTACGGTTCAACTTTCTTCGTAGCTACAGGATTTCACGGCCTCCATGTCATCATCGGATCCACCTTCCTAATTGTCTGCTTCTTCCGCCAATTAAAATTTCATTTCACCTCTAGTCACCATTTCGGTTTCGAAGC
A-O_aries : CACCCTTTACAATCTCAGACGGGGTTTACGGTTCAACTTTCTTCGTAGCTACAGGATTTCACGGCCTCCATGTCATCATCGGATCCACCTTCCTAATTGTCTGCTTCTTCCGCCAATTGAAATTTCATTTCACCTCTAGTCACCATTTCGGTTTCGAAGC
R-S_scrofa : CACCATTCACAATCTCCGACGGAGTGTATGGATCCACTTTCTTTGTGGCTACAGGATTTCACGGGTTGCACGTAATCATCGGATCTACTTTCCTAGCAGTGTGCTTACTACGACAACTAAAATTCCACTTCACATCCAACCACCACTTCGGCTTTGAAGC
A-S_scrofa : CACCATTCACAATCTCCGACGGAGTGTATGGATCCACTTTCTTTGTGGCTACAGGATTTCACGGGTTGCACGTAATCATCGGATCTACTTTCCTAGCAGTGTGCTTACTACGACAACTAAAATTCCACTTCACATCCAACCACCACTTCGGCTTTGAAGC
R-O_cuniculus : CATCTTTTACTATTTCAGATGGAGTCTACGGATCAACATTCTTCATAGCCACAGGATTTCACGGTCTTCACGTCATCATTGGGTCCACTTTTCTTACGGTCTGCCTTCTACGACAATTTAACTTTCACTTTACATCGAACCACCACTTTGGTTTTGAAGC
A-O_cuniculus : CATCTTTTACTATTTCAGATGGAGTCTACGGATCAACATTCTTCATAGCCACAGGATTTCACGGTCTTCACGTCATCATTGGGTCCACTTTTCTTACGGTCTGCCTTCTACGACAATTTAACTTTCACTTTACATCGAACCACCACTTTGGTTTTGAAGC
R-M_musculus : CATCATTCTCCATTTCAGATGGTATCTATGGTTCTACATTCTTCATGGCTACTGGATTCCATGGACTCCATGTAATTATTGGATCAACATTCCTTATTGTTTGCCTACTACGACAACTAAAATTTCACTTCACATCAAAACATCACTTCGGATTTGAAGC
A-M_musculus : CATCATTCTCCATTTCAGATGGTATCTATGGTTCTACATTCTTCATGGCTACTGGATTCCATGGACTCCATGTAATTATTGGATCAACATTCCTTATTGTTTGCCTACTACGACAACTAAAATTTCACTTCACATCAAAACATCACTTCGGATTTGAAGC
R-R_norvegicus : CATCATTTTCTATCTCAGACGGAATTTACGGCTCAACATTCTTCATAGCAACGGGATTTCATGGCCTCCACGTAATTATTGGCTCAACTTTCCTAATTGTCTGTCTACTACGACAACTAAAATTCCACTTCACATCAAAACATCATTTCGGATTTGAAGC
A-R_norvegicus : CATCATTTTCTATCTCAGACGGAATTTACGGCTCAACATTCTTCATAGCAACGGGATTTCATGGCCTCCACGTAATTATTGGCTCAACTTTCCTAATTGTCTGTCTACTACGACAACTAAAATTCCACTTCACATCAAAACATCATTTCGGATTTGAAGC
R-M_coypus : CTCCTTTCACGATCTCAGACGGAATTTATGGTTCAACATTCTTCGTAGCTACCGGCTTCCACGGTCTCCATGTAATTATTGGATCAACATTCTTATTAACCTGTCTGCTACGACAACTCTTTTATCATTTCACTTCAAAACACCACTTCGGTTTCGAAGC
A-M_coypus : CTCCTTTCACGATCTCAGACGGAATTTATGGTTCAACATTCTTCGTAGCTACCGGCTTCCACGGTCTCCATGTAATTATTGGATCAACATTCTTATTAACCTGTCTGCTACGACAACTCTTTTATCATTTCACTTCAAAACACCACTTCGGTTTCGAAGC
R-A_platyrhynchos : CCCCATTCTCAATCGCCGACAGCGTCTACGGCTCCACTTTCTTTGTTGCCACCGGATTCCACGGACTCCACGTGATCATCGGATCCACCTTCCTAACCGTCTGCCTCCTCCGACTAATCAAATTCCACTTCACATCAGACCACCACTTCGGATTTGAAGC
A-A_platyrhynchos : CCCCATTCTCAATCGCCGACAGCGTCTACGGCTCCACTTTCTTTGTTGCCACCGGATTCCACGGACTCCACGTGATCATCGGATCCACCTTCCTAACCGTCTGCCTCCTCCGACTAATCAAATTCCACTTCACATCAGACCACCACTTCGGATTTGAAGC
R-G_gallus : CCTCCTTCTCAATCGCTGACAGCGTCTACGGCTCCACCTTCTTCGTCGCTACAGGGTTCCACGGACTACATGTAATCATTGGATCATCCTTTTTAACAGTTTGCCTCCTACGACTAATCAAATTCCACTTCACACCAAACCACCACTTCGGATTTGAAGC
A-G_gallus : CCTCCTTCTCAATCGCTGACAGCGTCTACGGCTCCACCTTCTTCGTCGCTACAGGGTTCCACGGACTACATGTAATCATTGGATCATCCTTTTTAACAGTTTGCCTCCTACGACTAATCAAATTCCACTTCACACCAAACCACCACTTCGGATTTGAAGC
 CATCATTCACAATCTCAGACGGAGTCTACGGMTCAACATTCTTCGTAGCCACAGGATTCCACGGACTCCACGTAATTATTGGATCCACATTCCTAATTGTCTGCTTCCTACGACAACTAAAATTCCACTTCACATCWAACCACCACTTCGGATTTGAAGC

 * 11060 * 11080 * 11100 * 11120 * 11140 * 11160 * 11180 * 11200
R-E_caballus : AGCCGCTTGATACTGACACTTCGTCGACGTAGTCTGACTATTCTTGTACGTCTCTATTTATTGATGAGGATCCTATTCTTTTAGTATTGACCAGTACAATTGACTTCCAATCAATCAGCTTCGG-TATAACCCGAAAAAGAA-TAATAAAC---CTCATA
A-E_caballus : AGCCGCTTGATACTGACACTTCGTCGACGTAGTCTGACTATTCTTGTACGTCTCTATTTATTGATGAGGATCCTATTCTTTTAGTATTGACCAGTACAATTGACTTCCAATCAATCAGCTTCGG-TATAACCCGAAAAAGAA-TAATAAAC---CTCATA
R-C_bactrianus : TGCCGCCTGATATTGACATTTCGTAGATGTCGTCTGGCTGTTCCTCTATGTCTCTATCTATTGATGAGGTTCATGTCCTTTTAGTATCAATTAGTACAACTGACTTCCAATCAGTTAGCTTCGG-ATAGCCCCCGAAAAAGGATAATTAAT---CTTATA
A-C_bactrianus : TGCCGCCTGATATTGACATTTCGTAGATGTCGTCTGGCTGTTCCTCTATGTCTCTATCTATTGATGAGGTTCATGTCCTTTTAGTATCAATTAGTACAACTGACTTCCAATCAGTTAGCTTCGG-ATAGCCCCCGAAAAAGGATAATTAAT---CTTATA
R-C_lupus_familiaris : CGCTGCATGATATTGACACTTTGTTGATGTAGTCTGGCTATTCTTGTATGTATCTATTTATTGATGAGGATCCTATTTCTTTAGTAT-AACTAGTACAATTGACTTCCAATCAGTTAGCTCCAG-ATCAACCTGGAAAGAAG-TAATAAAC---GTTATA
A-C_lupus_familiaris : CGCTGCATGATATTGACACTTTGTTGATGTAGTCTGGCTATTCTTGTATGTATCTATTTATTGATGAGGATCCTATTTCTTTAGTAT-AACTAGTACAATTGACTTCCAATCAGTTAGCTCCAG-ATCAACCTGGAAAGAAG-TAATAAAC---GTTATA
R-N_procyonoides : CGCCGCATGGTACTGACATTTCGTCGACGTAGTCTGACTGTTCTTATACGTCTCTATTTATTGATGAGGATCTTACTTCTTTAGTAT-ATCAAGTACAACTGACTTCCAATCAGTTAGCTTCAG-TTCAACCTGAAAAGAAG-TAATAAAC---ATACTA
A-N_procyonoides : CGCCGCATGGTACTGACATTTCGTCGACGTAGTCTGACTGTTCTTATACGTCTCTATTTATTGATGAGGATCTTACTTCTTTAGTAT-ATCAAGTACAACTGACTTCCAATCAGTTAGCTTCAG-TTCAACCTGAAAAGAAG-TAATAAAC---ATACTA
R-V_lagopus : TGCCGCATGATACTGACACTTCGTCGACGTAGTCTGACTATTCCTATATGTTTCCATCTATTGATGAGGATCTTACTTCTTTAGTAT-AATTAGTACAATTGACTTCCAATCAATTAGCTTCAG-ACAGATCTGGAAAGAAG-TAATAAAC---ATTATA
A-V_lagopus : TGCCGCATGATACTGACACTTCGTCGACGTAGTCTGACTATTCCTATATGTTTCCATCTATTGATGAGGATCTTACTTCTTTAGTAT-AATTAGTACAATTGACTTCCAATCAATTAGCTTCAG-ACAGATCTGGAAAGAAG-TAATAAAC---ATTATA
R-M_putorius : AGCTGCCTGATATTGGCACTTTGTAGATGTCGTATGACTATTCCTATATGTATCTATCTATTGATGAGGATCCTATTTCTCTAGTATCAACAAGTACAGTTGACTTCCAATTAACTAGTTCTGG-TCTAACCCAGAGAGAAA-TAATAAAT---ATAATA
A-M_putorius : AGCTGCCTGATATTGGCACTTTGTAGATGTCGTATGACTATTCCTATATGTATCTATCTATTGATGAGGATCCTATTTCTCTAGTATCAACAAGTACAGTTGACTTCCAATTAACTAGTTCTGG-TCTAACCCAGAGAGAAA-TAATAAAT---ATAATA
R-B_taurus : CGCTGCCTGATACTGACATTTCGTAGACGTAGTCTGACTTTTCCTCTATGTTTCTATCTATTGATGAGGCTCCTATTCTTTTAGTATTAACTAGTACAGCTGACTTCCAATCAGCTAGTTTCGG-TCTAGTCCGAAAAAGAA-TAATAAAT---TTAATA
A-B_taurus : CGCTGCCTGATACTGACATTTCGTAGACGTAGTCTGACTTTTCCTCTATGTTTCTATCTATTGATGAGGCTCCTATTCTTTTAGTATTAACTAGTACAGCTGACTTCCAATCAGCTAGTTTCGG-TCTAGTCCGAAAAAGAA-TAATAAAT---TTAATA
R-O_aries : CGCTGCCTGATACTGACACTTCGTAGATGTAGTATGACTTTTCCTCTATATATCCATCTACTGATGAGGCTCATGTCCTTTTAGTATTAATTAGTACAACTGACTTCCAATCAGTTAGTTTCGG-TCTAATCCGAAAAAGAA-CAATAAAC---CTTATA
A-O_aries : CGCTGCCTGATACTGACACTTCGTAGATGTAGTATGACTTTTCCTCTATGTATCCATCTACTGATGAGGCTCATGTCCTTTTAGTATTAATTAGTACAACTGACTTCCAATCAGTTAGTTTCGG-TCTAATCCGAAAAAGAA-CAATAAAC---CTTATA
R-S_scrofa : CGCAGCCTGATACTGACACTTCGTAGATGTAGTTTGACTATTCCTTTACGTATCAATCTATTGATGAGGATCCTACTCTTTTAGTATTAAGCAGTACAATTGACTTCCAATCAATCAGTTTCGG-TAAACTCCGAAAAAGAG-TAATAAAT---ATTATA
A-S_scrofa : CGCAGCCTGATACTGACACTTCGTAGATGTAGTTTGACTATTCCTTTACGTATCAATCTATTGATGAGGATCCTACTCTTTTAGTATTAAGCAGTACAATTGACTTCCAATCAATCAGTTTCGG-TAAACTCCGAAAAAGAG-TAATAAAT---ATTATA
R-O_cuniculus : AGCCGCATGATACTGACACTTTGTAGATGTAGTCTGACTATTCCTATATGTATCAATCTATTGATGAGGGTCATACTCTTTTAGTATCAACTAGTACAGCTGACTTCCAATCAGTTAGTTTTGGCTCCAACCCAAAAAAGAG-TAATAAAC---CTGATA
A-O_cuniculus : AGCCGCATGATACTGACACTTTGTAGATGTAGTCTGACTATTCCTATATGTATCAATCTATTGATGAGGGTCATACTCTTTTAGTATCAACTAGTACAGCTGACTTCCAATCAGTTAGTTTTGGCTCCAACCCAAAAAAGAG-TAATAAAC---CTAATA
R-M_musculus : CGCAGCATGATACTGACATTTTGTAGACGTAGTCTGACTTTTCCTATACGTCTCCATTTATTGATGAGGATCTTACTCCCTTAGTAT-AATTAATATAACTGACTTCCAATTAGTAGATTCTGA-ATAAACCCAGAAGAGAG-TAATTAAC---CTGTAC
A-M_musculus : CGCAGCATGATACTGACATTTTGTAGACGTAGTCTGACTTTTCCTATACGTCTCCATTTATTGATGAGGATCTTACTCCCTTAGTAT-AATTAATATAACTGACTTCCAATTAGTAGATTCTGA-ATAAACCCAGAAGAGAG-TAATCAAC---CTGTAC
R-R_norvegicus : CGCAGCATGATACTGACACTTCGTAGATGTAGTTTGACTATTCCTATACGTTTCTATCTATTGATGAGGATCCTACTCCCTTAGTAT-AAACAATACAACTGACTTCCAATCAGTTAATTCTGA-AAAAACTCAGAAGAGAG-TAATTAAC---CTACTT
A-R_norvegicus : CGCAGCATGATACTGACACTTCGTAGATGTAGTTTGACTATTCCTATACGTTTCTATCTATTGATGAGGATCCTACTCCCTTAGTAT-AAACAATACAACTGACTTCCAATCAGTTAATTCTGA-AAAAACTCAGAAGAGAG-TAATTAAC---CTACTT
R-M_coypus : TGCAGCCTGATACTGACATTTCGTGGACGTAGTTTGACTCTTCTTATACGTCTCAATCTACTGATGAGGCTCATACTTTCTTAGTAT-ACTTAGTACTACTGACTTCCAATCAGTAAGCTCCGA-ATAACTCCGGAAGAGAG-TAATAAAT---ATTGTT
A-M_coypus : TGCAGCCTGATATTGACATTTCGTGGACGTAGTTTGACTCTTCTTATACGTCTCAATCTACTGATGAGGCTCATACTTTCTTAGTAT-ACTTAGTACTACTGACTTCCAATCAGTAAGCTCCGA-ATAACTCCGGAAGAGAG-TAATAAAT---ATTGTT
R-A_platyrhynchos : CGCAGCCTGATACTGACACTTCGTAGACGTTATCTGACTATTCCTCTATATAACCATCTACTGATGAGGATCTTGCTCTTCTAGTAT-ATTAATTACAATTGACTTCCAATCTCTAAAATCTGGTGCAAGCCCAGAGAAGAG-CAATGAACATACTCACA
A-A_platyrhynchos : CGCAGCCTGATACTGACACTTCGTAGACGTTATCTGACTATTCCTCTATATAACCATCTACTGATGAGGATCTTGCTCTTCTAGTAT-ATTAATTACAATTGACTTCCAATCTCTAAAATCTGGTGCAAGCCCAGAGAAGAG-CAATGAACATACTCACA
R-G_gallus : AGCAGCCTGATACTGACACTTCGTAGACATCATCTGACTCTTCCTCTACATATCCATATACTGATGAGGATCTTGCTCTTCTAGTAT-ACTCATTACAACTGACTTCCAATCTTTAAAATCTGGTATCAACCCAGAGAAGAG-CAATGAACACACTTACA
A-G_gallus : AGCAGCCTGATACTGACACTTCGTAGACATCATCTGACTCTTCCTCTACATATCCATATACTGATGAGGATCTTGCTCTTCTAGTAT-ACTCATTACAACTGACTTCCAATCTTTAAAATCTGGTATCAACCCAGAGAAGAG-CAATGAACACACTTACA
 CGCAGCCTGATACTGACACTTCGTAGACGTAGTCTGACTATTCCTATATGTATCTATCTATTGATGAGGATCCTACTCTTTTAGTATTAATTAGTACAACTGACTTCCAATCAGTTAGTTTCGGTWYAAACCCGGAAAAGAGATAATAAACAYACTTATA

 * 11220 * 11240 * 11260 * 11280 * 11300 * 11320 * 11340 * 11360
R-E_caballus : CTGACACTCCTCACTAACACAT-TACTAGCCTCGCTACTCGTACTCATCGCATTCTGACTACCACAACTAAACATCTATGCAGAAAAAACCAGCCCATATGAATGCGGATTTGACCCTATAGGGTCAGCACGCCTCCCCTTCTCAATAAAATTTTTCTT-
A-E_caballus : CTGACACTCCTCACTAACACAT-TACTAGCCTCGCTACTCGTACTCATCGCATTCTGACTACCACAACTAAACATCTATGCAGAAAAAACCAGCCCATATGAATGCGGATTTGACCCTATAGGGTCAGCACGCCTCCCCTTCTCAATAAAATTTTTCTT-
R-C_bactrianus : CTGGCCTTATTTACAAATACTG-CCTTAGCATCCCTTCTTGTACTAATTGCATTCTGACTCCCCCAACTATATATTTATGCAGAAAAGACGGGCCCTTACGAATGTGGCTTCGACCCCATAGGCTCCGCCCGTTTACCCTTTTCCATAAAATTTTTCTT-
A-C_bactrianus : CTGGCCTTATTTACAAATACTG-CCTTAGCATCCCTTCTTGTACTAATTGCATTCTGACTCCCCCAACTATATATTTATGCAGAAAAGACGGGCCCTTACGAATGTGGCTTCGACCCCATAGGCTCCGCCCGTTTACCCTTTTCCATAAAATTTTTCTT-
R-C_lupus_familiaris : TTAACTTTGATAACTAATGTAA-CCCTAGCATCCTTACTTGTACTAATCGCATTCTGACTTCCCCAGCTAAATATCTATACAGACAAGACAAGCCCCTACGAATGTGGTTTTGACCCCATGGGATCTGCTCGCCTACCTTTCTCTATAAAATTTTTCCT-
A-C_lupus_familiaris : TTAACTTTGATAACTAATGTAA-CCCTAGCATCCTTACTTGTACTAATCGCATTCTGACTTCCCCAGCTAAATATCTATACAGACAAGACAAGCCCCTACGAATGTGGTTTTGACCCCATGGGATCTGCTCGCCTACCTTTCTCTATAAAATTTTTCCT-
R-N_procyonoides : CTGACTTTAACAACTAATATCG-CCCTAGCATCACTACTCGTAATAATCGCATTCTGACTCCCTCAACTAAACATCTACGCGGATAAGGCGAGCCCGTACGAATGCGGTTTTGACCCTATGGGGTCCGCTCGCCTGCCTTTCTCCATAAAATTTTTCCT-
A-N_procyonoides : CTGACTTTAACAACTAATATCG-CCCTAGCATCACTACTCGTAATAATCGCATTCTGACTCCCTCAACTAAACATCTACGCGGATAAGGCGAGCCCGTACGAATGCGGTTTTGACCCTATGGGGTCCGCTCGCCTGCCTTTCTCCATAAAATTTTTCCT-
R-V_lagopus : CTTACTCTAGTAACCAATGTTA-CCCTGGCTTCCCTACTCGTAATAATCGCATTCTGACTCCCCCAACTAAATATTTATGCCGACAAGACGAGTCCTTACGAATGCGGCTTCGACCCCATGGGATCGGCGCGCCTGCCGTTTTCCATAAAATTTTTCCT-
A-V_lagopus : CTTACTCTAGTAACCAATGTCA-CCCTGGCTTCCCTACTCGTAATAATCGCATTCTGACTCCCCCAACTAAATATTTATGCCGACAAGACGAGTCCTTACGAATGCGGCTTCGACCCCATGGGATCGGCGCGCCTGCCGTTTTCCATAAAATTTTTCCT-
R-M_putorius : TTAACCATACTTATCAATGTAT-CCCTAGCATCTCTACTTATTCTAATCGCATTCTGACTACCTCAACTAAATGTCTACACAGAAAAAGCAAGCCCATATGAATGTGGTTTTGATCCCTTAGGATCAGCACGCTTACCATTCTCCATAAAATTTTTCCT-
A-M_putorius : TTAACCATACTTATCAATGTAT-CCCTAGCATCTCTACTTATTCTAATCGCATTCTGACTACCTCAACTAAATGTCTACACAGAAAAAGCAAGCCCATATGAATGTGGTTTTGATCCCTTAGGATCAGCACGCTTACCATTCTCCATAAAATTTTTCCT-
R-B_taurus : CTAGCCCTCCTGACCAATTTTA-CACTAGCCACCCTACTCGTCATCATCGCATTCTGACTTCCCCAACTAAATGTATACTCTGAGAAAACAAGCCCATACGAATGTGGATTTGACCCCATAGGATCAGCCCGCCTTCCCTTCTCTATAAAATTCTTTCT-
A-B_taurus : CTAGCCCTCCTGACCAATTTTA-CACTAGCCACCCTACTCGTCATCATCGCATTCTGACTTCCCCAACTAAATGTATACTCTGAGAAAACAAGCCCATACGAATGTGGATTTGACCCCATAGGATCAGCCCGCCTTCCCTTCTCTATAAAATTCTTTCT-
R-O_aries : ATTACTCTCCTAACTAACTTCA-CGCTAGCTACATTACTCGTAACCATCGCATTCTGACTTCCCCAACTGAACGTGTATTCAGAAAAAACAAGCCCATACGAATGTGGATTTGACCCCATAGGGTCTGCTCGCCTCCCCTTCTCTATAAAATTCTTCCT-
A-O_aries : ATTACTCTCCTAACTAACTTCA-CGCTAGCTACATTACTCGTAACCATCGCATTCTGACTTCCCCAACTGAACGTGTATTCAGAAAAAACAAGCCCATACGAATGTGGATTTGACCCCATAGGGTCTGCTCGCCTCCCCTTCTCTATAAAATTCTTCCT-
R-S_scrofa : CTAACACTATTCACAAACGTAA-CCCTAGCCTCCCTACTCGTACTAATCGCATTCTGACTACCCCAACTAAACACATATTCAGAAAAAACAAGCCCATATGAATGTGGATTTGACCCCATAGGATCAGCACGCCTCCCATTCTCAATAAAATTTTTCCT-
A-S_scrofa : CTAACACTATTCACAAACGTAA-CCCTAGCCTCCCTACTCGTACTAATCGCATTCTGACTACCCCAACTAAACACATATTCAGAAAAAACAAGCCCATATGAATGTGGATTTGACCCCATAGGATCAGCACGCCTCCCATTCTCAATAAAATTTTTCCT-
R-O_cuniculus : CTAGTTCTATTAATTAACACCA-CCATTTCCTTAGTCCTAGTTACAATTGCATTCTGACTCCCTCAATTAAATATCTATTCAGAAAAATCAAGCCCTTATGAATGCGGATTCGATCCCATAGGATCAGCACGACTCCCTTTCTCTATAAAATTTTTTCT-
A-O_cuniculus : CTAGTACTATTAATTAACACCA-CCATTTCCTTAGTCCTAGTTACAATTGCATTCTGACTCCCTCAATTAAATATCTATTCAGAAAAATCAAGCCCTTATGAATGCGGATTCGATCCCATAGGATCAGCACGACTCCCTTTCTCTATAAAATTTTTTCT-
R-M_musculus : ACTGTTATCTTCATTAATATTT-TATTATCCCTAACGCTAATTCTAGTTGCATTCTGACTCCCCCAAATAAATCTGTACTCAGAAAAAGCAAATCCATATGAATGCGGATTCGACCCTACAAGCTCTGCACGTCTACCATTCTCAATAAAATTTTTCTT-
A-M_musculus : ACTGTTATCTTCATTAATATTT-TATTATCCCTAACGCTAATTCTAGTTGCATTCTGACTCCCCCAAATAAATCTGTACTCAGAAAAAGCAAATCCATATGAATGCGGATTCGACCCTACAAGCTCTGCACGTCTACCATTCTCAATAAAATTTTTCTT-
R-R_norvegicus : ATTATCATCACAATTAACATCA-CCTTATCTTTTATCCTCATTTCAATTGCATTCTGATTGCCTCAAATAAACTTATACTCCGAAAAAGCAAACCCATATGAATGTGGCTTCGACCCAACAAGTTCTGCACGCCTTCCTTTTTCAATAAAATTTTTCTT-
A-R_norvegicus : ATTATCATCACAATTAACATCA-CCTTATCTTTTATCCTCATTTCAATTGCATTCTGATTGCCTCAAATAAATTTATACTCCGAAAAAGCAAACCCATATGAATGTGGCTTCGACCCAACAAGTTCTGCACGCCTTCCTTTTTCAATAAAATTTTTCTT-
R-M_coypus : GTATCAATCTTCACTAATTATC-TATTAGCAATAGCCCTCATTATAATCGCATTTTGATTCCCTCAACTCAACATTTACACAGAAAAAATCAGCCCTTATGAATGTGGATTTGACCCCACAGAAATCACACGTCTTCCCTTCACCATAAAATTTTTCCT-
A-M_coypus : GTATCAATCTTCACTAATTATC-TATTAGCAATAGCCCTCATTATAATCGCATTTTGATTCCCTCAACTCAACATTTACACAGAAAAAATCAGCCCTTATGAATGTGGATTTGACCCCACAGAAATCACACGTCTTCCCTTCACCATAAAATTTTTCCT-
R-A_platyrhynchos : TTTATGTT-CTCCCTATCACTGGTCCTAAGTGCCATCCTAACCGCACTAAACTTCTGACTCGCCCAAATGACCCCCGACTCAGAAAAACTCTCACCATACGAATGCGGATTCGACCCCCTCGGGTCTGCACGCCTACCATTCTCAATCCGATTCTTCCTC
A-A_platyrhynchos : TTTATGTT-CTCCCTATCACTGGTCCTAAGTGCCATCCTAACCGCACTAAACTTCTGACTCGCCCAAATGACCCCCGACTCAGAAAAACTCTCACCATACGAATGCGGATTCGACCCCCTCGGGTCTGCACGCCTACCATTCTCAATCCGATTCTTCCTC
R-G_gallus : TTTATACT-CTCACTATCCTTTCTACTAAGCGCTGCACTAACTACTATAAACTTTTGACTAGCCCAAATAGCCCCAGACACAGAAAAACTGTCACCGTACGAATGCGGATTTGACCCACTAGGATCAGCCCGACTCCCATTCTCAATCCGATTCTTCCT-
A-G_gallus : TTTATACT-CTCACTATCCTTTCTACTAAGCGCTGCACTAACTACTATAAACTTTTGACTAGCCCAAATAGCCCCAGACACAGAAAAACTGTCACCGTACGAATGCGGATTTGACCCACTAGGATCAGCCCGACTCCCATTCTCAATCCGATTCTTCCTC
 CTWACTCTMCTMACTAATATTASCCCTAGCCTCCCTACTCGTAATAATCGCATTCTGACTCCCCCAACTAAAYATCTACTCAGAAAAAACAAGCCCATACGAATGTGGATTTGACCCCATAGGATCAGCACGCCTMCCMTTCTCAATAAAATTTTTCCTC

 * 11380 * 11400 * 11420 * 11440 * 11460 * 11480 * 11500 * 11520
R-E_caballus : AGTGGCCATTACATTTCTGCTATTCGACTTAGAAATTGCCCTCCTATTACCCCTTCCATGAGCATCCCAAACAACTA--ACCTAAACACTATACTTATCATAGCACTAGTCCTAATCTCTCTTCTAGCCATCAGCCTAGCCTACGAATGAACCCAAAAAG
A-E_caballus : AGTGGCCATTACATTTCTGCTATTCGACTTAGAAATTGCCCTCCTATTACCCCTTCCATGAGCATCCCAAACAACTA--ACCTAAACACTATACTTATCATAGCACTAGTCCTAATCTCTCTTCTAGCCATCAGCCTAGCCTACGAATGAACCCAAAAAG
R-C_bactrianus : GATTGCCATTACATTCCTCCTATTTGACCTGGAAATTGCCCTCCTCTTGCCCTTACCTTGAGCAACCCAAACGAACT--ACCTATATATCATATTAACCATAGCACTTCTTCTCATTTTACTACTGGCAGCTAGCCTCGCCTACGAATGAACCCAAGGGG
A-C_bactrianus : GATTGCCATTACATTCCTCCTATTTGACCTGGAAATTGCCCTCCTCTTGCCCTTACCTTGAGCAACCCAAACGAACT--ACCTATATATCATATTAACCATAGCACTTCTTCTCATTTTACTACTGGCAGCTAGCCTCGCCTACGAATGAACCCAAGGGG
R-C_lupus_familiaris : AGTTGCCATCACATTTCTGCTTTTCGACCTAGAAATTGCACTCCTACTCCCACTTCCCTGAGCGTCACAAACCAACA--AGCTAACAACAATACTTATCATAGCACTCCTACTAATCTCCCTCCTAGCTGCGAGCCTAGCGTATGAATGGACCGAAAAGG
[truncated: 277,793 more chars]
